# Electrochemical C−H Amidation of Heteroarenes with *N*‐Alkyl Sulfonamides in Aqueous Medium

**DOI:** 10.1002/chem.202004229

**Published:** 2020-11-26

**Authors:** Yan Zhang, Zhipeng Lin, Lutz Ackermann

**Affiliations:** ^1^ Institut für Organische und Biomolekulare Chemie Georg-August-Universität Göttingen Tammannstrasse 2 37077 Göttingen Germany; ^2^ Key Laboratory of the Ministry of Education for Advanced, Catalysis Materials Zhejiang Normal University Yingbin Road 688 321004 Jinhua P. R. China

**Keywords:** amidation, electrochemistry, heterocycles, radicals, sulfonamides

## Abstract

The construction of C−N bonds by free radical reactions represents a powerful synthetic approach for direct C−H amidations of arenes or heteroarenes. Developing efficient and more environmentally friendly synthetic methods for C−H amidation reactions remains highly desirable. Herein, metal‐free electrochemical oxidative dehydrogenative C−H amidations of heteroarenes with *N*‐alkylsulfonamides have been accomplished. The catalyst‐ and chemical‐oxidant‐free C−H amidation features an ample scope and employs electricity as the green and sole oxidant. A variety of heteroarenes, including indoles, pyrroles, benzofuran and benzothiophene, thereby underwent this C(sp^2^)−H nitrogenation. Cyclic voltammetry studies and control experiments provided evidence for nitrogen‐centered radicals being directly generated under metal‐free electrocatalysis.

## Graphical abstract

**Heterocycles**: Metal‐free electrochemical dehydrogenative C−H amidation of heteroarenes with *N*‐alkylsulfonamides have been accomplished. The catalyst‐ and chemical oxidant‐free C−H amidation featured ample substrate scope and employed electricity as the sole oxidant (see scheme).

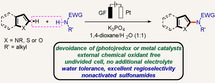

The construction of C−N bonds is one of the most vibrant areas in organic synthesis. Among several approaches, the development of methods for direct C−H amidations of arenes or heteroarenes is critically important, due to the prevalence of aromatic amines in both pharmaceuticals and materials.[^1^, ^2^] Although copper‐catalyzed Ullman couplings^[3]^ and palladium‐catalyzed Buchwald–Hartwig amidations^[4]^ with aryl halides have proven to be powerful, direct amidations of aromatic C−H bonds without pre‐functionalization have been pursued as more step‐economical routes. Thus, significant contributions have been reported on transition‐metal‐catalyzed C−H amidations of arenes (Scheme 1 a).^[5]^ However, the need for nonremovable directing groups, high reaction temperatures and waste‐generating stoichiometric oxidants continue to be their insurmountable limitation.[^5g^, ^6^, ^7^, ^8^]

**Scheme 1:**
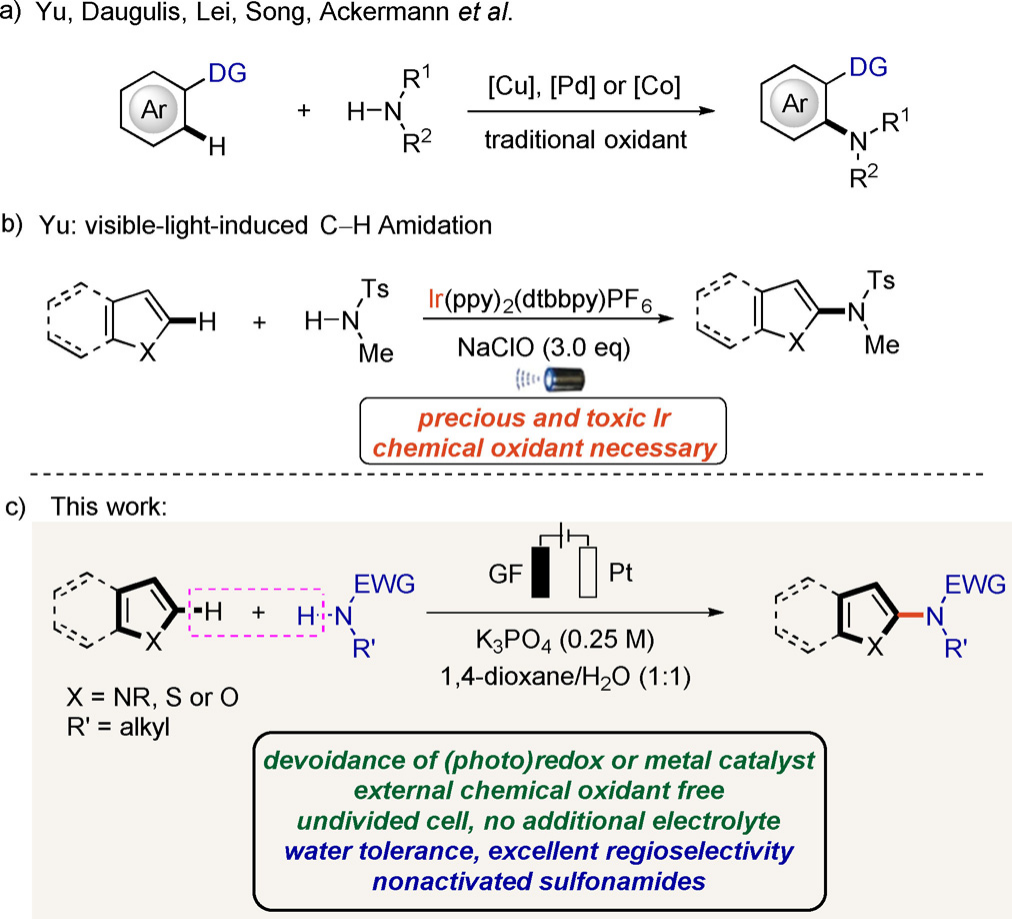
Strategies for direct C−H amidation of heteroarenes.

In contrast, the construction of C−N bonds by free radical reactions represents a powerful class of chemical transformations and has attracted considerable attention.^[9]^ In particular, nitrogen‐centered radicals have received increasing attention.^[10]^ The well‐established synthetic approaches to form *N*‐radicals include the proton‐coupled electron transfer (PCET) or oxidative deprotonation electron transfer (ODET) developed by Knowles^[11]^ and Xiao^[12]^ in the presence of iridium or ruthenium photocatalysts. Inspired by these work, Yu recently developed a visible‐light‐promoted direct oxidative C−H amidation of heteroarenes with nonactivated sulfonamides (Scheme 1 b).^[13]^ At the beginning of this reaction, stoichiometric NaClO is needed to oxidatively quench the iridium^III^ catalyst to generate iridium^IV^. Thus, the development of efficient and more environmentally friendly synthetic methods is still highly desirable and valuable,^[9d]^ particularly by means of electrosynthesis. In connection with our continued interest in electrochemical syntheses[^14^, ^15^] and C−N bond formation using electricity as the sole oxidant,^[16]^ we questioned whether the efficiency and mildness noted in the *N*‐radical formation could be translated into an electro‐oxidative heteroarene amidation strategy. We have now devised an unprecedented C−H amidation of heteroarenes that can be performed with commercially available, inexpensive reagents under green and operationally‐simple electrochemical conditions (Scheme 1 c). Compared to previously disclosed methods, salient features of our strategy comprise (a) the absence of external chemical oxidants, (b) (photo)redox and metal catalyst‐free condition, (c) the use of a user‐friendly undivided cell setup without additional electrolyte, (d) high regioselectivity, and (e) full water tolerance.

We initiated our studies by probing various reaction conditions for the envisioned metal‐free electrochemical C−H amidation reaction of indole **1 a** in a most user‐friendly undivided cell set‐up with a platinum plate cathode and graphite felt (GF) anode (Table 1 and Table S2 in the Supporting Information). After considerable preliminary experimentation, we observed that the desired amidation with amide **2 a** was best accomplished with a mixed solvent consisting of 1,4‐dioxane/H_2_O (1:1) and K_3_PO_4_ as both the key base and electrolyte (entries 1–12). The efficacy of the electro‐oxidation was reflected by efficient C−H amidations occurring at a temperature of 80 °C (entries 13 and 14). Attempts to reduce the amount of K_3_PO_4_ or coupling partner **2 a** loading led to a decreased yields (entry 15). A significant decline of yield was observed when a Pt anode or Fe cathode was used. It is noteworthy that when Ni‐foam was used as the cathode, the amidation product **3 aa** was isolated in 64 % yield (entry 16). Control experiments confirmed the essential role of electricity for the electrooxidative amidation (entries 17).

**Table 1:** Electrochemical C−H amidation of heteroarenes with sulfonamides.^[a]^

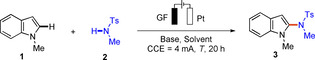				
Entry	Solvent	Base	*T* [°C]	Yield [%]
1	MeCN/H_2_O (3:1)	Na_2_CO_3_	50	15
2	*t*‐AmylOH/H_2_O (3:1)	Na_2_CO_3_	50	n.r.
3	*i*‐PrOH/H_2_O (1:1)	Na_2_CO_3_	50	n.r.
4	DMSO/H_2_O (2:1)	Na_2_CO_3_	50	13
5	1,4‐dioxane/H_2_O (1:1)	Na_2_CO_3_	50	27
6	1,4‐dioxane/H_2_O (1:1)	–	50	n.r.
7	1,4‐dioxane/H_2_O (1:1)	K_2_CO_3_	50	24
8	1,4‐dioxane/H_2_O (1:1)	NaOH	50	37
9	1,4‐dioxane/H_2_O (1:1)	NaOPiv	50	20
10	1,4‐dioxane/H_2_O (1:1)	NaOAc	50	32
11	1,4‐dioxane/H_2_O (1:1)	K_3_PO_4_	50	55
12	1,4‐dioxane/H_2_O (2:1)	K_3_PO_4_	50	50
13	1,4‐dioxane/H_2_O (1:1)	K_3_PO_4_	30	21
**14**	**1,4‐dioxane/H_2_O (1:1)**	**K_3_PO_4_**	**80**	**68**
15^[b]^	1,4‐dioxane/H_2_O (1:1)	K_3_PO_4_	80	54
16^[c]^	1,4‐dioxane/H_2_O (1:1)	K_3_PO_4_	80	64
17^[d]^	1,4‐dioxane/H_2_O (1:1)	K_3_PO_4_	80	n.r.

[a] Undivided cell, GF anode, Pt cathode, constant current=4 mA, **1** (0.5 mmol), **2 a** (1.0 mmol, 2.0 equiv), base (0.25 m, 1.0 mmol, 2.0 equiv), solvent (4.0 mL), under air, 20 h. Yield of isolated products. [b] With K_3_PO_4_ 1.0 equiv. [c] GF(+)|Ni(−) instead of GF(+)|Pt(−). [d] No electricity.

With the optimized reaction conditions in hand, we explored the scope of the electrochemical C−H amidation with a variety of heteroarenes **1** (Scheme 2). Several substituents on the indole N were explored. Methyl (Me), benzyl (Bn), allyl and *para*‐methylphenyl (Tol) indoles performed well to give the desired products **3 aa**–**3 da** in moderate to good yields. However, we found that 1*H*‐indole and *N*‐Ac indole could not be amidated in this transformation. We were pleased to find that C3‐substituted indoles underwent this transformation efficiently despite a possible existing steric hindrance. 3‐methyl and 3‐methoxymethyl (MOM) indole derivatives furnished the 2‐aminated products **3 ea** and **3 fa**. The more sensitive hydroxymethyl group was likewise tolerated, albeit with lower yield. We then examined various substituents at the C4‐C7 positions and 2‐aminated indole derivatives **3 ha**–**3 va** were obtained in reasonable yields (50–78 %). The structure of the product **3 pa** was unambiguously confirmed by single‐crystal X‐ray analysis.^[17]^ Additionally, other heteroarenes, such as pyrrole, benzofuran and thiophene derivatives, were also accessible to site‐selectively give the corresponding products **3 wa**–**3 za**.

**Scheme 2:**
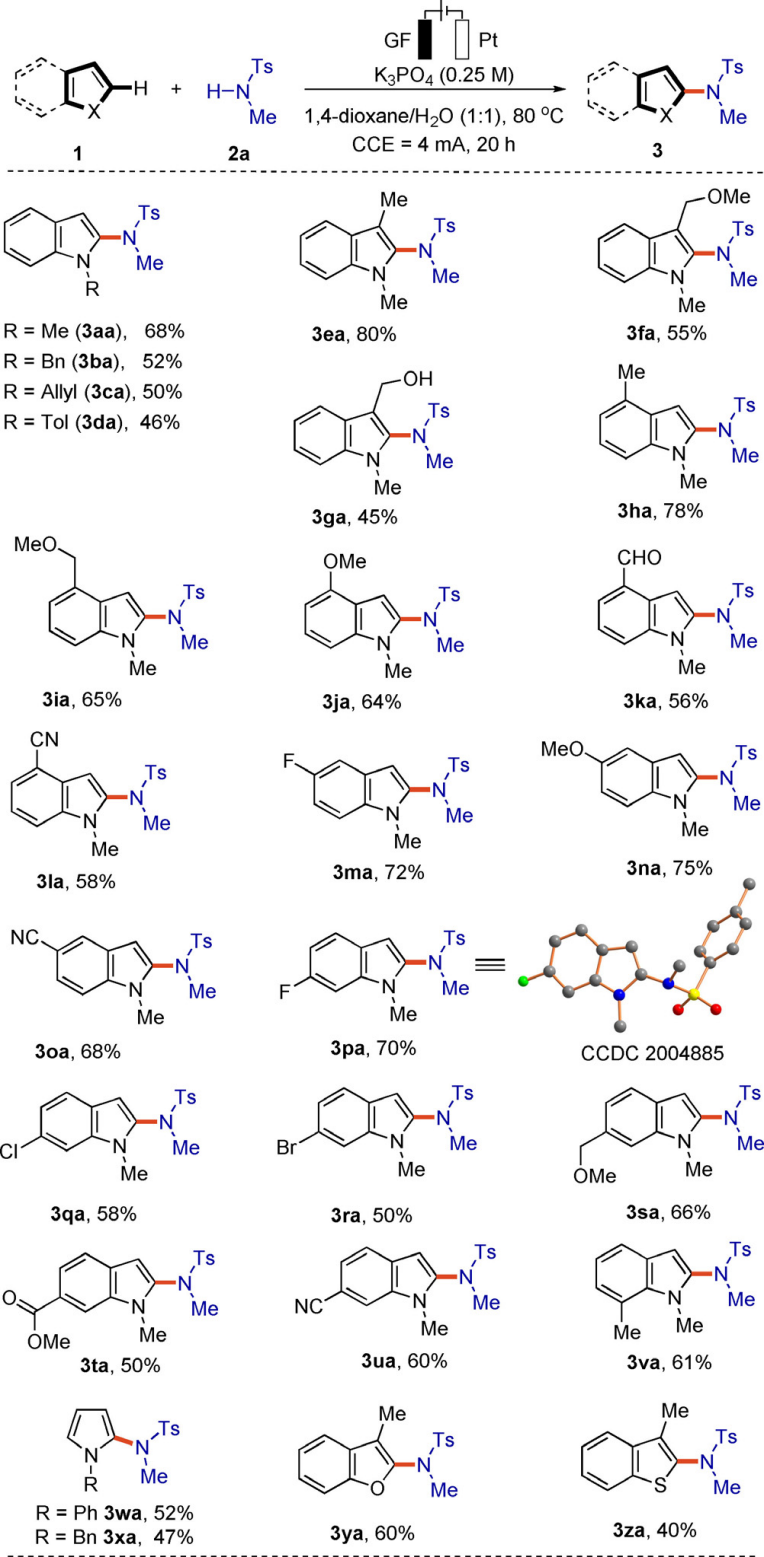
Electrochemical C−H amidation of different heteroarenes **1**.

The scope of the amidation reaction was further examined with various substituted sulfonamides **2** (Scheme 3). Generally, the alkyl part of the sulfonamide **2** did not significantly alter the reaction efficiency and the amidated products **3** were smoothly obtained in low to moderate yields (**3 ab**–**3 ao**). As depicted in Schemes 2 and 3, amidation approach was compatible with several sensitive functional groups, such as chloro, bromo, nitrile, hydroxyl, formyl, ester and amino. It is noteworthy that naphthalene **1 a“** underwent the electro‐oxidative C−H amidation with *N*‐methyl‐*para*‐toluenesulfonamide **2 a**, giving a minor amount of product **3 a′a**.

**Scheme 3:**
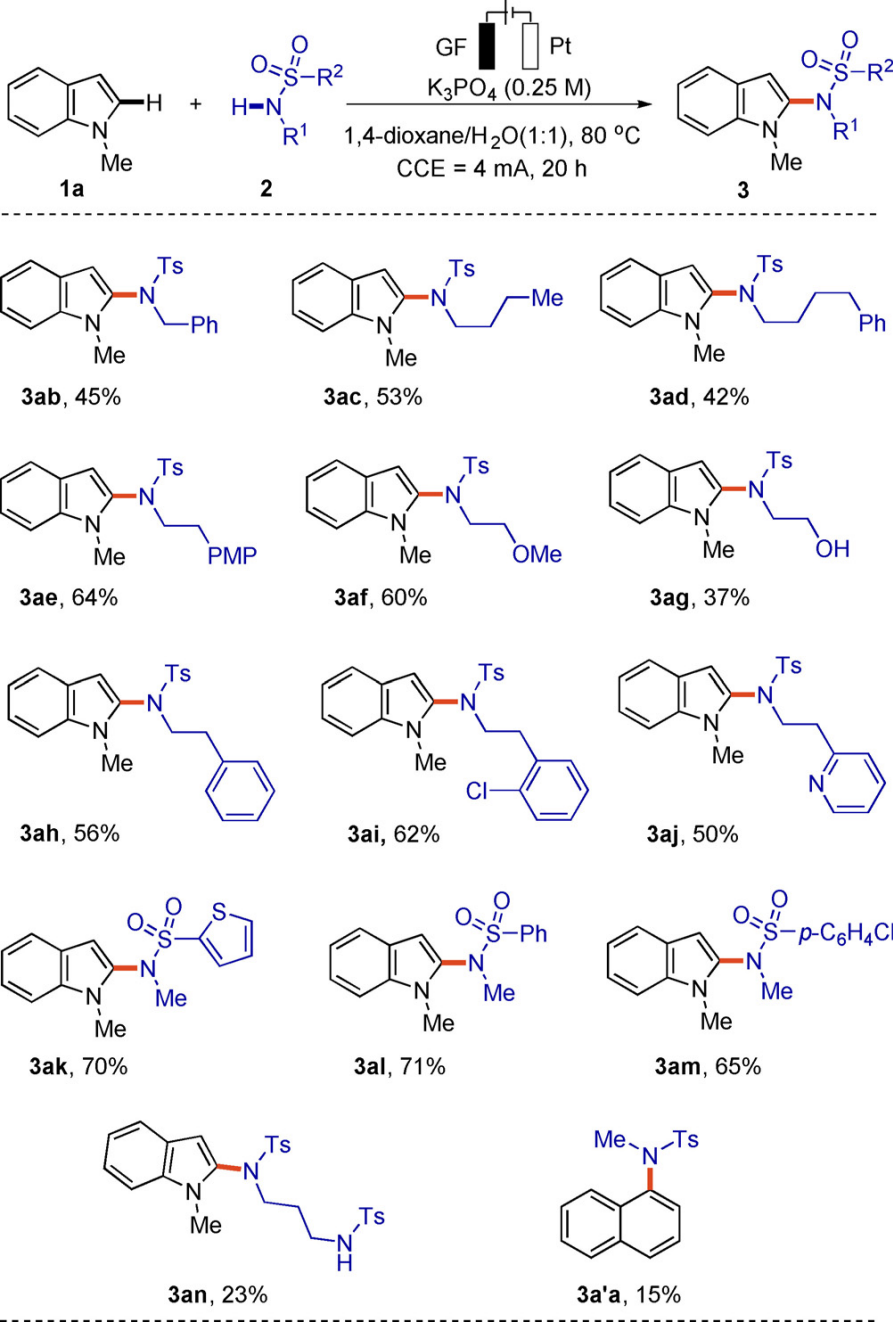
Electrochemical C−H amidation with *N*‐alkyl sulfonamides **2**.

The scalability of the electrochemical C−H amidation was probed next and a 10 mmol scale reaction of **1 a** and **2 a** yielded 1.8 g of product **3 aa** (Scheme 4 a). Derivation of this product was performed to access the privileged indole derivative **4** with a diverse range of pharmacological activities^[18]^ (Scheme 4 b).

**Scheme 4:**
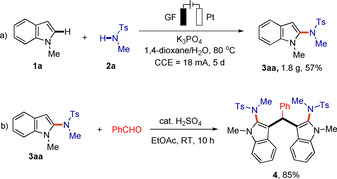
a) Gram‐scale reaction and b) derivatization of compound **3 aa**.

To investigate the interaction between base and sulfonamide **2**, thus understanding the pathway for the generation of the *N*‐centered amide radical, cyclic voltammetry studies were performed (Figure 1 and Figure S1 in the Supporting Information). We found that the oxidation potential of sulfonamide alone is relatively high with *E*_1/2_=+2.5 V vs. Ag/AgCl in acetonitrile. We then carried out cyclic voltammetry studies of sulfonamide **2 a** in acetonitrile/water (10:1) containing 0.2 m K_3_PO_4_, which acts as a base as well as an electrolyte. The mixture of the sulfonamide and K_3_PO_4_ resulted in a new oxidation wave at around +1.0 V vs. Ag/AgCl. Compared to sulfonamide, the oxidation potential and onset potential of the new peak were shifted to lower potentials. This outcome is consistent with the formation of a *N*‐centered amide anion from sulfonamide and K_3_PO_4_, and the *N*‐anion is electron‐rich and much more easily oxidized to a *N*‐centered amide radical.

**Figure 1:**
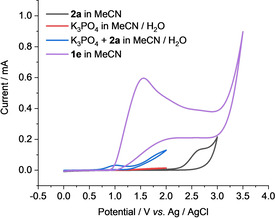
Cyclic voltammetry studies. Conditions: Cyclic voltammograms of sulphonamide **2 a** (5.0 mm) in MeCN containing 0.1 m *n*Bu_4_NPF_6_ (black), K_3_PO_4_ (0.2 m) (red), and the mixture of **2 a** (0.2 m) and K_3_PO_4_ (0.2 m) (blue) in acetonitrile/H_2_O (10:1), cyclic voltammogram of indole **1 e** (5.0 mm) in MeCN containing 0.1 m *n*Bu_4_NBF_4_ (purple), at ambient temperature. The scan rates were 100 mV s^−1^.

Considering that this conversion process includes a radical addition step, a number of control experiments was thereafter conducted. *N*‐centered radical **A** could be trapped by 2,6‐di‐*tert*‐butyl‐4‐methylphenol (BHT). When a mixture of sulfonamide **2 a** and BHT was subjected to the standard electrochemical conditions, and the trapping product **5** was isolated through an intramolecular radical transfer,^[19]^ independent of the presence of indole **1 a** (Scheme 5 a). The radical clock reaction with 3‐(1‐cyclopropylvinyl)‐1‐methyl‐1*H*‐indole **1 b“** as a radical trapping reagent provided the radical cyclization product **6** in 11 % yield, along with the otherwise typical product **3 b′a** (Scheme 5 b). Furthermore, we synthesized deuterated substrate [D_1_]‐**1 a** (95 % D) and an kinetic isotope effect (KIE) experiment was undertaken by parallel independent reactions, giving a KIE value of *k*_H_/*k*_D_≈1.2 (Scheme 5 c). These results suggest a fast and facile C−H cleavage.

**Scheme 5:**
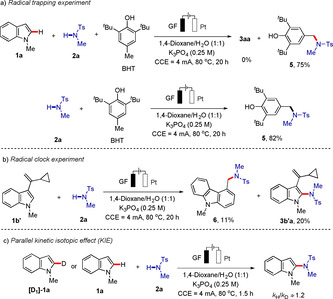
Summary of key mechanistic findings.

Based on our mechanistic studies, a radical mechanism is proposed in Scheme 6 for this electrocatalyzed C(sp^2^)‐H amidation reaction. First, *N*‐alkylsulfonamide **2 a** undergoes a concerted proton‐coupled electron transfer event at the anode to give the key *N*‐radical **A**.^[11]^ The subsequent radical addition to indole **1 a** furnishes the new C−N bond and the thermodynamically more stable carbon‐centered radical **B**. Then, radical **B** undergoes further SET oxidation to form the cation intermediate **C**. Finally, **C** generates the final amidation products **3 aa** through proton elimination/aromatization, generating molecular hydrogen as a byproduct at the cathode.

**Scheme 6:**
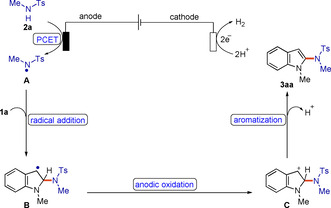
Proposed mechanism.

In conclusion, we have developed an efficient approach for electrochemical C−H amidations of unactivated heteroarenes by oxidative cleavage of N−H bonds under metal catalyst‐ and chemical oxidant‐free conditions. A variety of heteroarenes, including indoles, pyrroles, benzofurans and benzothiophenes, proved applicable to the electro‐C−H‐amidation. The robust catalyst‐free C−H amidation was broadly applicable to *N*‐alkylsulfonamides under green and mild reaction conditions. Control experiments and cyclic voltammetry studies were indicative of the generation of a nitrogen radical via an anodic oxidation.

## Conflict of interest

The authors declare no conflict of interest.

## Supporting information

As a service to our authors and readers, this journal provides supporting information supplied by the authors. Such materials are peer reviewed and may be re‐organized for online delivery, but are not copy‐edited or typeset. Technical support issues arising from supporting information (other than missing files) should be addressed to the authors.

Supplementary

## References

[1a] D. G. Brown , J. Boström , J. Med. Chem. 2016, 59, 4443–4458; 26571338 10.1021/acs.jmedchem.5b01409

[1b] R. Gianatassio , J. M. Lopchuk , J. Wang , C.-M. Pan , L. R. Malins , L. Prieto , T. A. Brandt , M. R. Collins , G. M. Gallego , N. W. Sach , J. E. Spangler , H. Zhu , J. Zhu , P. S. Baran , Science 2016, 351, 241–246; 26816372 10.1126/science.aad6252 PMC4730898

[1c] G. Dequirez , V. Pons , P. Dauban , Angew. Chem. Int. Ed. 2012, 51, 7384–7395; 10.1002/anie.201201945 22730346

[1d] J. Yamaguchi , A. D. Yamaguchi , K. Itami , Angew. Chem. Int. Ed. 2012, 51, 8960–9009; 10.1002/anie.201201666 22887739

[1e] J. Alvarez-Builla , J. J. Vaquero , J. Barluenga , Modern Heterocyclic Chemistry, Wiley-VCH, Weinheim, 2011;

[1f] A. Ricci , Amino Group Chemistry: From Synthesis to the Life Sciences, Wiley, Hoboken, 2008;

[1g] A. Ricci , Modern Amidation Methods, Wiley-VCH, Weinheim, 2000.

[2a] L.-L. Li , E. W.-G. Diau , Chem. Soc. Rev. 2013, 42, 291–304; 23023240 10.1039/c2cs35257e

[2b] A. Yella , H.-W. Lee , H. N. Tsao , C. Yi , A. K. Chandiran , M. K. Nazeeruddin , E. W.-G. Diau , C.-Y. Yeh , S. M. Zakeeruddin , M. Grätzel , Science 2011, 334, 629–634. 22053043 10.1126/science.1209688

[3a] J. Lindley , Tetrahedron 1984, 40, 1433–1456;

[3b] F. Ullmann , Ber. Dtsch. Chem. Ges. 1903, 36, 2382–2384.

[4a] P. Ruiz-Castillo , S. L. Buchwald , Chem. Rev. 2016, 116, 12564–12649; 27689804 10.1021/acs.chemrev.6b00512 PMC5070552

[4b] J. Bariwal , E. Van der Eycken , Chem. Soc. Rev. 2013, 42, 9283–9303; 24077333 10.1039/c3cs60228a

[4c] J. F. Hartwig , Acc. Chem. Res. 2008, 41, 1534–1544; 18681463 10.1021/ar800098p PMC2819174

[4d] F. Y. Kwong , A. Klapars , S. L. Buchwald , Org. Lett. 2002, 4, 581–584; 11843596 10.1021/ol0171867

[4e] A. Klapars , J. C. Antilla , X. Huang , S. L. Buchwald , J. Am. Chem. Soc. 2001, 123, 7727–7729. 11481007 10.1021/ja016226z

[5] For reviews on transition-metal-catalyzed C−H amidations, see:

[5a] Y. Park , Y. Kim , S. Chang , Chem. Rev. 2017, 117, 9247–9301; 28051855 10.1021/acs.chemrev.6b00644

[5b] H. Kim , S. Chang , ACS Catal. 2016, 6, 2341–2351;

[5c] J. Jiao , K. Murakami , K. Itami , ACS Catal. 2016, 6, 610–633;

[5d] Q.-Z. Zheng , N. Jiao , Chem. Soc. Rev. 2016, 45, 4590–4627; 27056573 10.1039/c6cs00107f

[5e] M.-L. Louillat , F. W. Patureau , Chem. Soc. Rev. 2014, 43, 901–910; 24217419 10.1039/c3cs60318k

[5f] V. S. Thirunavukkarasu , S. I. Kozhushkov , L. Ackermann , Chem. Commun. 2014, 50, 29–39; 10.1039/c3cc47028h 24212194

[5g] S. H. Cho , J. Y. Kim , J. Kwak , S. Chang , Chem. Soc. Rev. 2011, 40, 5068–5083; 21643614 10.1039/c1cs15082k

[5h] F. Collet , C. Lescot , P. Dauban , Chem. Soc. Rev. 2011, 40, 1926–1936. 21234469 10.1039/c0cs00095g

[6] For copper-catalyzed amidations, see:

[6a] J. Roane , O. Dangulis , J. Am. Chem. Soc. 2016, 138, 4601–4607; 26990413 10.1021/jacs.6b01117 PMC4829344

[6b] M. Shang , S.-Z. Sun , H.-X. Dai , J.-Q. Yu , J. Am. Chem. Soc. 2014, 136, 3354–3357; 24527701 10.1021/ja412880r

[6c] X. Wang , Y. Jin , Y. Zhao , L. Zhu , H. Fu , Org. Lett. 2012, 14, 452–455; 22206482 10.1021/ol202884z

[6d] A. E. Wendlandt , A. M. Suess , S. S. Stahl , Angew. Chem. Int. Ed. 2011, 50, 11062–11087; 10.1002/anie.201103945 22034061

[7] For cobalt-catalyzed amidations, see:

[7a] S. S. Bera , M. S. Maji , Org. Lett. 2020, 22, 2615–2620; 32207626 10.1021/acs.orglett.0c00589

[7b] N. Sauermann , R. Mei , L. Ackermann , Angew. Chem. Int. Ed. 2018, 57, 5090–5094; 10.1002/anie.201802206 29509336

[7c] C. Du , P.-X. Li , X. Zhu , J.-N. Han , J.-L. Niu , M. P. Song , Acs Catal. 2017, 7, 2810–2814;

[7d] L.-B. Zhang , S.-K. Zhang , D. Wei , X. Zhu , X.-Q. Hao , J.-H. Su , J.-L. Niu , M.-P. Song , Org. Lett. 2016, 18, 1318–1321; 26928948 10.1021/acs.orglett.6b00241

[7e] Q. Yan , T. Xiao , Z. Liu , Y. Zhang , Adv. Synth. Catal. 2016, 358, 2707–2711;

[7f] B. Sun , T. Yoshino , S. Matsunaga , M. Kanai , Adv. Synth. Catal. 2014, 356, 1491–1495, and cited references.

[8] Selected other metal-catalyzed amidations, see:

[8a] Q.-K. Kang , Y. Lin , Y. Li , H. Shi , J. Am. Chem. Soc. 2020, 142, 3706–3711; 32039590 10.1021/jacs.9b13684

[8b] T. Zhang , X. Hu , Z. Wang , T. Yang , H. Sun , G. Li , H. Lu , Chem. Eur. J. 2016, 22, 2920–2924; 26712274 10.1002/chem.201504880

[8c] V. S. Thirunavukkarasu , K. Raghuvanshi , L. Ackermann , Org. Lett. 2013, 15, 3286–3289; 23767780 10.1021/ol401321q

[8d] T. W. Lyons , M. S. Sanford , Chem. Rev. 2010, 110, 1147–1169, and references therein. 20078038 10.1021/cr900184e PMC2836499

[9a] D. Kurandina , D. Yadagiri , M. Rivas , A. Kavun , P. Chuentragool , K. Hayama , V. Gevorgyan , J. Am. Chem. Soc. 2019, 141, 8104–8109; 31046256 10.1021/jacs.9b04189 PMC6873700

[9b] A. Ruffoni , F. Juliá , T. D. Svejstrup , A. J. Mcmillan , J. J. Douglas , D. Leonori , Nat. Chem. 2019, 11, 426–433; 31011173 10.1038/s41557-019-0254-5

[9c] H. Jiang , G. Seidler , A. Studer , Angew. Chem. Int. Ed. 2019, 58, 16528–16532; 10.1002/anie.201910926 PMC6900080 31529676

[9d] X. Hu , G. Zhang , L. Nie , T. Kong , A. Lei , Nat. Commun. 2019, 10, 5467–5476; 31784522 10.1038/s41467-019-13524-4 PMC6884519

[9e] X. Wang , D. Xia , W. Qin , R. Zhou , X. Zhou , Q. Zhou , W. Liu , X. Dai , H. Wang , S. Wang , L. Tan , D. Zhang , H. Song , X.-Y. Liu , Y. Qin , Chem 2017, 2, 803–816;

[9f] E. Ito , T. Fukushima , T. Kawakami , K. Murakami , K. Itami , Chem 2017, 2, 383–392;

[9g] X.-Q. Hu , X. Qi , J.-R. Chen , Q.-Q. Zhao , Q. Wei , Y. Lan , W.-J. Xiao , Nat. Commun. 2016, 7, 11188–11199; 27048886 10.1038/ncomms11188 PMC4823831

[9h] H. Kim , T. Kim , D. G. Lee , S. W. Roh , C. Lee , Chem. Commun. 2014, 50, 9273–9276; 10.1039/c4cc03905j 25007122

[9i] B. Giese , J. Dupuis , Angew. Chem. Int. Ed. Engl. 1983, 22, 622–623;

[9j] B. Giese , Angew. Chem. Int. Ed. Engl. 1983, 22, 753–764;

[10] For reviews on *N*-centered radicals, see:

[10a] H. Jiang , A. Studer , Chem. Soc. Rev. 2020, 49, 1790–1811; 32055811 10.1039/c9cs00692c

[10b] H. Jiang , A. Studer , CCS. Chem. 2019, 1, 38–49;

[10c] H. Yi , G. Zhang , H. Wang , Z. Huang , J. Wang , A. K. Singh , A. Lei , Chem. Rev. 2017, 117, 9016–9085; 28639787 10.1021/acs.chemrev.6b00620

[10d] M. D. Kärkäs , Acs Catal. 2017, 7, 4999–5022;

[10e] T. Xiong , Q. Zhang , Chem. Soc. Rev. 2016, 45, 3069–3087; 27116936 10.1039/c5cs00852b

[10f] A. Studer , D. P. Curran , Angew. Chem. Int. Ed. 2016, 55, 58–102; 10.1002/anie.201505090 26459814

[10g] J.-R. Chen , X.-Q. Hu , L.-Q. Lu , W.-J. Xiao , Chem. Soc. Rev. 2016, 45, 2044–2056. 26839142 10.1039/c5cs00655d

[11a] D. C. Miller , G. J. Choi , H. S. Orbe , R. R. Knowles , J. Am. Chem. Soc. 2015, 137, 13492–13495; 26439818 10.1021/jacs.5b09671 PMC4629195

[11b] G. J. Choi , R. R. Knowles , J. Am. Chem. Soc. 2015, 137, 9226–9229. 26166022 10.1021/jacs.5b05377 PMC4643263

[12] X.-Q. Hu , J.-R. Chen , Q. Wei , F.-L. Liu , Q.-H. Deng , A. M. Beauchemin , W.-J. Xiao , Angew. Chem. Int. Ed. 2014, 53, 12163–12167; 10.1002/anie.201406491 25513705

[13] K. Tong , X. Liu , Y. Zhang , S. Yu , Chem. Eur. J. 2016, 22, 15669–15673. 27599166 10.1002/chem.201604014

[14] For reviews on electrochemical C−H functionalization, see:

[14a] T. H. Meyer , I. Choi , C. Tian , L. Ackermann , Chem. 2020, 6, 2484–2496;

[14b] L. Ackermann , Acc. Chem. Res. 2020, 53, 84–104; 31854967 10.1021/acs.accounts.9b00510

[14c] Q.-L. Yang , P. Fang , T.-S. Mei , Chin. J. Chem. 2018, 36, 338–352;

[14d] M. D. Kärkäs , Chem. Soc. Rev. 2018, 47, 5786–5865; 29911724 10.1039/c7cs00619e

[14e] N. Sauermann , T. H. Meyer , L. Ackermann , Chem. Eur. J. 2018, 24, 16209–16217; 29920808 10.1002/chem.201802706

[14f] N. Sauermann , T. H. Meyer , Y. Qiu , L. Ackermann , ACS Catal. 2018, 8, 7086–7103.

[15] For selected recent examples of electrochemical C−H amidations, see:

[15a] Y. Kawamata , J. C. Vantourout , D. P. Hickey , P. Bai , L. Chen , Q. Hou , W. Qiao , K. Barman , M. A. Edwards , A. F. Garrido-Castro , J. N. deGruyter , H. Nakamura , K. Knouse , C. Qin , K. J. Clay , D. Bao , C. Li , J. T. Starr , C. Garcia-Irizarry , N. Sach , H. S. White , M. Neurock , S. D. Minteer , P. S. Baran , J. Am. Chem. Soc. 2019, 141, 6392–6402; 30905151 10.1021/jacs.9b01886 PMC6996791

[15b] Y. Adeli , K. Huang , Y. Liang , Y. Jiang , J. Liu , S. Song , C.-C. Zeng , N. Jiao , ACS Catal. 2019, 9, 2063–2067;

[15c] J. H. Wang , T. Lei , X. L. Nan , H. L. Wu , X. B. Li , B. Chen , C. H. Tung , L. Z. Wu , Org. Lett. 2019, 21, 5581–5585; 31276420 10.1021/acs.orglett.9b01910

[15d] S. Tang , S. Wang , Y. Liu , H. Cong , A. Lei , Angew. Chem. Int. Ed. 2018, 57, 4737–4741; 10.1002/anie.201800240 29498166

[15e] Z.-W. Hou , Z.-Y. Mao , Y. Y. Melcamu , X. Lu , H.-C. Xu , Angew. Chem. Int. Ed. 2018, 57, 1636–1639; 10.1002/anie.201711876 29266679

[15f] X. Gao , P. Wang , L. Zeng , S. Tang , A. Lei , J. Am. Chem. Soc. 2018, 140, 4195–4199; 29522680 10.1021/jacs.7b13049

[15g] Q.-L. Yang , X.-Y. Wang , J.-Y. Lu , L.-P. Zhang , P. Fang , T.-S. Mei , J. Am. Chem. Soc. 2018, 140, 11487–11494; 30165030 10.1021/jacs.8b07380

[15h] F. Xu , Y.-J. Li , C. Huang , H.-C. Xu , ACS Catal. 2018, 8, 3820–3824;

[15i] T. Gieshoff , A. Kehl , D. Schollmeyer , K. D. Moeller , S. R. Waldvogel , J. Am. Chem. Soc. 2017, 139, 12317–12324; 28792218 10.1021/jacs.7b07488

[15j] P. Xiong , H.-H. Xu , H.-C. Xu , J. Am. Chem. Soc. 2017, 139, 2956–2959; 28199102 10.1021/jacs.7b01016

[15k] J. Wu , Y. Zhou , Y. Zhou , C.-W. Chiang , A. Lei , ACS Catal. 2017, 7, 8320–8323;

[15l] C. Li , Y. Kawamata , H. Nakamura , J. C. Vantourout , Z. Liu , Q. Hou , D. Bao , J. T. Starr , J. Chen , M. Yan , P. S. Baran , Angew. Chem. Int. Ed. 2017, 56, 13088–13093; 10.1002/anie.201707906 PMC5792186 28834098

[15m] T. Morofuji , A. Shimizu , J. Yoshida , J. Am. Chem. Soc. 2015, 137, 9816–9819; 26225441 10.1021/jacs.5b06526

[15n] B. R. Rosen , E. W. Werner , A. G. O'Brien , P. S. Baran , J. Am. Chem. Soc. 2014, 136, 5571–5574; 24697810 10.1021/ja5013323 PMC4004216

[15o] W. J. Gao , W. C. Li , C. C. Zeng , H. Y. Tian , L. M. Hu , R. D. Little , J. Org. Chem. 2014, 79, 9613–9618. For reviews, see:25255384 10.1021/jo501736w

[15p] R. Francke , R. D. Little , Chem. Soc. Rev. 2014, 43, 2492–2521; 24500279 10.1039/c3cs60464k

[15q] A. Jutand , Chem. Rev. 2008, 108, 2300–2347. 18605756 10.1021/cr068072h

[16] Y. Qiu , J. Struwe , T. H. Meyer , J. C. A. Oliveira , L. Ackermann , Chem. Eur. J. 2018, 24, 12784–12789. 29901828 10.1002/chem.201802832

[17] Deposition Numbers 2004885 contains the supplementary crystallographic data for this paper. These data are provided free of charge by the joint Cambridge Crystallographic Data Centre and Fachinformationszentrum Karlsruhe Access Structures service www.ccdc.cam.ac.uk/structures.

[18a] J. Deng , T. Sanchez , N. Neamati , J. M. Briggs , J. Med. Chem. 2006, 49, 1684–1692; 16509584 10.1021/jm0510629

[18b] K. M. Dalessandri , G. L. Firestone , M. D. Fitch , H. L. Bradlow , L. F. Bjeldanes , Nutr. Cancer. 2004, 50, 161–167; 15623462 10.1207/s15327914nc5002_5

[18c] R. Bell , S. Carmeli , N. Sar , J. Nat. Prod. 1994, 57, 1587–1590. 7853008 10.1021/np50113a022

[19a] Y. Gao , G. Lu , P. Zhang , L. Zhang , G. Tang , Y. Zhao , Org. Lett. 2016, 18, 1242–1245; 26925953 10.1021/acs.orglett.6b00056

[19b] H. Egami , T. Ide , Y. Kawato , Y. Hamashima , Chem. Commun. 2015, 51, 16675–16678. 10.1039/c5cc07011b 26431449

