# Supplementary material for: Electrochemical C−H Amidation of Heteroarenes with N‐Alkyl Sulfonamides in Aqueous Medium
Source: Chemistry. 2020 Nov 26;27(1):242–6. doi: 10.1002/chem.202004229 (PMC7898600; doi:10.1002/chem.202004229)
Supplement: Supplementary file 1 — Supplementary [file CHEM-27-242-s001.pdf]

Chemistry–A European Journal

Supporting Information

Electrochemical C–H Amidation of Heteroarenes with *N*-Alkyl Sulfonamides in Aqueous Medium

Yan Zhang,*[a, b] Zhipeng Lin,[a] and Lutz Ackermann*[a]

Table of Contents

1. General Remarks S2

2. Synthesis and Characterization of **2** S5

3. General Procedure for Electrochemical C–H Amidation of Heteroarenes S7

4. Characterization Data of Products **3** S8

5. Gram-Scale Reaction S32

6. Mechanistic Studies S34

6.1 Preparation of [D_1]-**1a** S34

6.2 KIE Studies S35

6.3 Cyclic Voltammetry S36

7. References S37

8. NMR Spectra S38

1. General Remarks

Catalytic reactions were carried out in undivided electrochemical cells using pre-dried glassware under air, if not noted otherwise. As mentioned in the manuscript, hydrogen gas is generated during the reaction and can lead to an explosive gaseous mixture with atmospheric oxygen. Therefore, the reactions should be performed in a well-ventilated fume-hood and specialized reaction equipment should be used. NMR spectra were recorded on BRUKER AVANCE III 400 or Avance III HD 500 spectrometer. $CDCl_3$ was used as the solvent. Chemical shifts were referenced relative to residual solvent signal ($CDCl_3$: ^{1}H NMR: δ 7.26 ppm, ^{13}C NMR: δ 77.16 ppm). The following abbreviations are used to describe peak patterns where appropriate: br = broad, s = singlet, d = doublet, t = triplet, q = quartet, m = multiplet. Coupling constants (*J*) are reported in Hertz (Hz). All IR spectra were recorded on a Bruker FT-IR Alpha-P device. Analytical thin layer chromatography (TLC) was performed on TLC Silica gel 60 F_{254} from Merck with detection at 254 nm or 360 nm. Preparative chromatographic separations were carried out on Merck Geduran SI 60 (40–63 µm, 70–230 mesh ASTM) silica gel. Electrospray-ionization (ESI) mass spectra were obtained on Bruker micrOTOF and maXis instruments. All systems are equipped with time-of-flight (TOF) analyzers. Melting points were measured with micro melting point apparatus. Electrocatalysis was conducted using an AXIOMET AX-3003P potentiostat in constant current mode. Platinum electrodes (10 mm × 15 mm × 0.25 mm, 99.9%; obtained from ChemPur® Karlsruhe, Germany) and graphite felt (GF) electrodes (10 mm × 15 mm × 6 mm, SIGRACELL® GFA 6 EA, obtained from SGL Carbon, Wiesbaden, Germany) were connected using stainless steel adapters. CV studies were performed using a Metrohm Autolab PGSTAT204 workstation and Nova 2.1 software. Unless otherwise noted, some materials obtained from commercial suppliers were used directly without further purification. Indole **1** and *N*-alkylsulfonamide **2** were prepared according to the literature.[1, 2]

Table S-1. Scopes of indoles **1** and sulfonamides **2.**

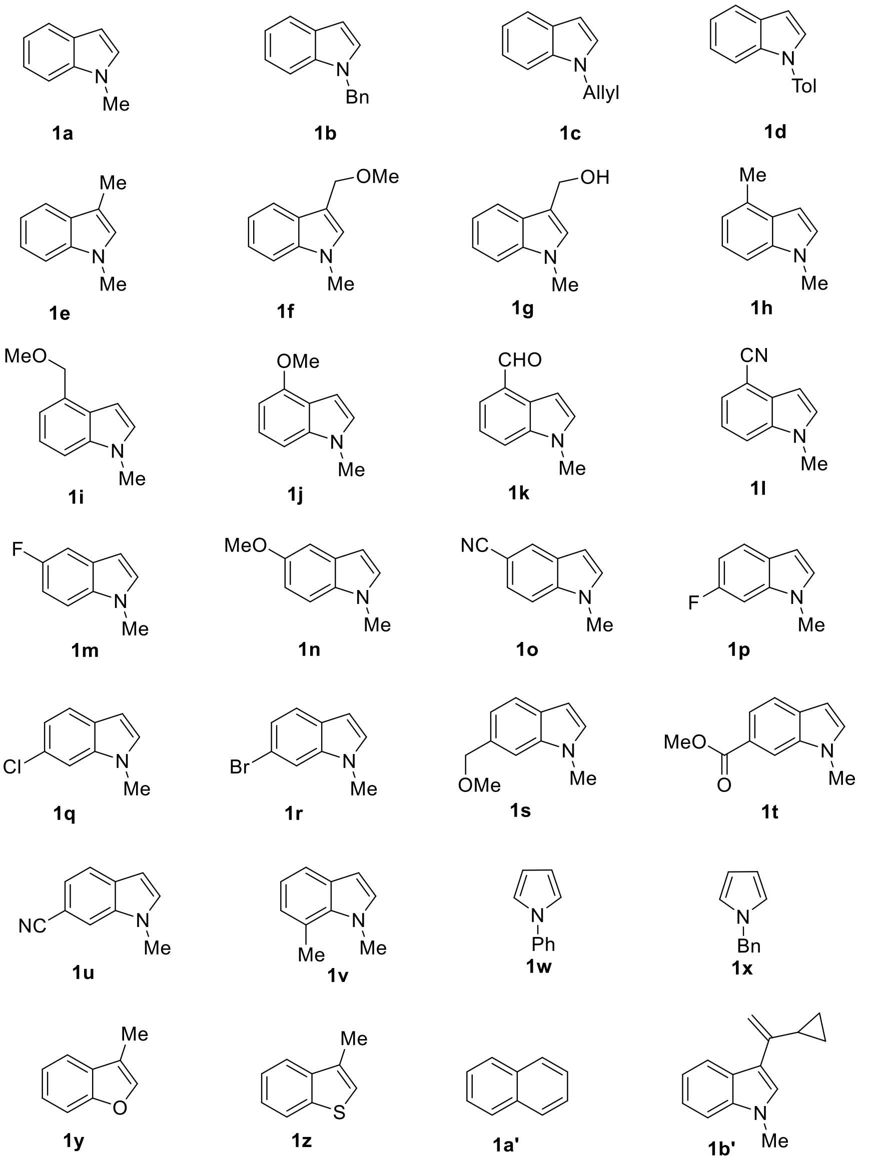

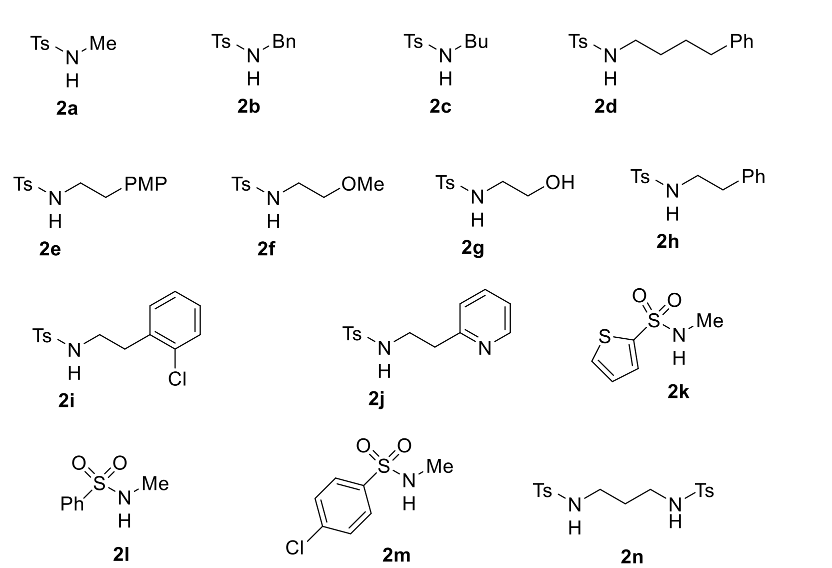

Table S-2. Electrochemical C–H Amidation of Heterorenes with Sulfonamides.[a]

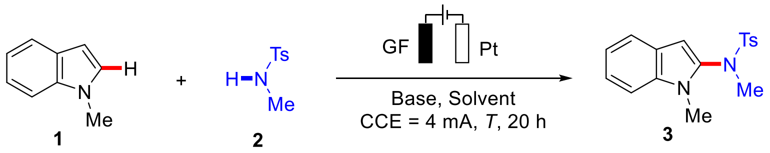

Entry	Solvent	Base	T / °C	Yield / %
1	MeCN	Na_2CO_3	50	n.r.
2	MeCN/H_2O (3:1)	Na_2CO_3	50	15
3	*t*-AmylOH/H_2O (3:1)	Na_2CO_3	50	n.r.
4	*i*-PrOH/H_2O (1:1)	Na_2CO_3	50	n.r.
5	DMSO/H_2O (2:1)	Na_2CO_3	50	13
6	1,4-Dioxane/H_2O (1:1)	Na_2CO_3	50	27
7	1,4-Dioxane/H_2O (1:1)	–	50	n.r.
8	1,4-Dioxane/H_2O (1:1)	K_2CO_3	50	24
9	1,4-Dioxane/H_2O (1:1)	NaOH	50	37
10	1,4-Dioxane/H_2O (1:1)	NaOPiv	50	20
11	1,4-Dioxane/H_2O (1:1)	NaOAc	50	32
12	1,4-Dioxane/H_2O (1:1)	K_3PO_4	50	55
13	1,4-Dioxane/H_2O (2:1)	K_3PO_4	50	50
14[b]	1,4-Dioxane/H_2O (1:1)	K_3PO_4	50	34
15	1,4-Dioxane/H_2O (1:1)	K_3PO_4	30	21
16	1,4-Dioxane/H_2O (1:1)	K_3PO_4	80	68
17	1,4-Dioxane/H_2O (1:1)	K_3PO_4	90	57
18[c]	1,4-Dioxane/H_2O (1:1)	K_3PO_4	80	60
19[d]	1,4-Dioxane/H_2O (1:1)	K_3PO_4	80	54
20[e]	1,4-Dioxane/H_2O (1:1)	K_3PO_4	80	64
21[f]	1,4-Dioxane/H_2O (1:1)	K_3PO_4	80	50
22[g]	1,4-Dioxane/H_2O (1:1)	K_3PO_4	80	n.r.
23[h]	1,4-Dioxane/H_2O (1:1)	K_3PO_4	80	n.r.

[a] Undivided cell, GF anode, Pt cathode, constant current = 4 mA, **1** (0.50 mmol), **2a** (1.0 mmol, 2.0 equiv), base (1.0 mmol, 2.0 equiv), solvent (4 mL), under air, 20 h. Yield of isolated products. [b] CCE = 8 mA. [c] **2a** (0.75 mmol, 1.5 equiv). [d] With K_3PO_4 (1.0 equiv). [e] GF(+)|Ni(−) instead of GF(+)|Pt(−). [f] GF(+)|Fe(−) instead of GF(+)|Pt(−). [g] Pt(+)|Pt(−) instead of GF(+)|Pt(−). [h] No electricity.

2. Synthesis and Characterization of Sulfonamides 2

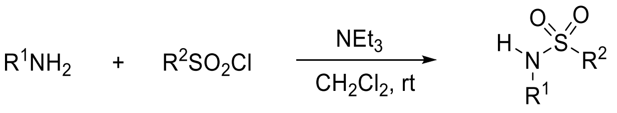

In a 50 mL flask with a stir-bar was charged with primary amine (5 mmol) and dichloromethane (25 mL). The solution was stirred at 0 °C and triethylamine (0.8 mL, 6 mmol) was added. Then sulfochlorides (5 mmol) was added dropwise to the solution. After stirring for 8 h at room temperature, the reaction mixture was quenched with water, extracted with CH_2Cl_2 (2 × 20 mL). The combined organic layer was dried over anhydrous Na_2SO_4, concentrated to afford the residue. The residue was subject to flash column chromatography on silica gel using *n*-hexane/EtOAc (v/v, 1:1) as eluent to give the pure product in good yield.

Specially, the synthesis of alcohol **2g** (the 2nd step in the follow scheme) was according to a protocol reported in the literature.[3]

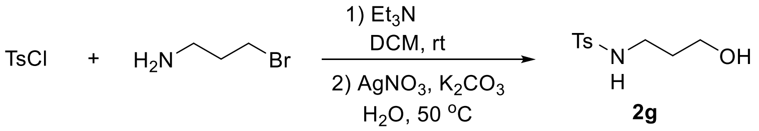

For selected characterization of substrates **2**, see:

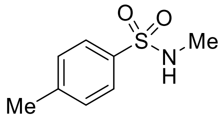

2a

***N*,4-dimethylbenzenesulfonamide (2a)**.

White solid; **M.p.**: 82–83 °C; **^{1}H NMR** (400 MHz, $CDCl_3$): δ = 7.72 (d, *J* = 8.4 Hz, 2H), 7.26 (d, *J* = 8.4 Hz, 2H), 5.24 (q, *J* = 4.8 Hz, 1H), 2.55 (d, *J* = 5.2 Hz, 3H), 2.38 (s, 3H). **^{13}C NMR** (100 MHz, $CDCl_3$): δ = 143.4 (C_q), 135.5 (C_q), 129.6 (CH), 127.1 (CH), 29.1 (CH_3), 21.4 (CH_3). **IR** (ATR): 3271, 1595, 1494, 1452, 1408, 1306, 1154, 820 cm^{-1}. **MS** (ESI) *m/z* (relative intensity): 186 $[M+H]^+$ (30), 208 $[M+Na]^+$ (100). **HR-MS** (ESI) *m/z* calc. for $C_8H_{12}NO_2S$ $[M+H]^+$: 186.0583, found: 186.0581. The characterization data corresponds with those reported in the literature.[2]

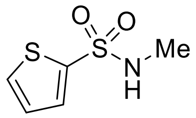

2k

***N*-methylthiophene-2-sulfonamide (2k)**.

Oil; **^{1}H NMR** (400 MHz, $CDCl_3$): δ = 7.57–7.53 (m, 2H), 7.04–7.02 (m, 1H), 5.33 (q, J = 4.8 Hz, 1H), 2.60 (d, J = 5.6 Hz, 3H). **^{13}C NMR** (100 MHz, $CDCl_3$): δ = 139.0 (C_q), 132.1 (CH), 132.0 (CH), 127.4 (CH), 29.2 (CH_3), 21.4 (CH_3). **IR** (ATR): 3290, 1508, 1469, 1403, 1316, 1226, 1151, 1014, 832 cm^{-1}. **MS** (ESI) *m/z* (relative intensity): 178 $[M+H]^+$ (20), 200 $[M+Na]^+$ (100). **HR-MS** (ESI) *m/z* calc. for $C_5H_8NO_2S_2$ $[M+H]^+$: 177.9991, found: 177.9992.

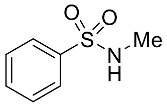

2l

***N*-methylbenzenesulfonamide (2l)**.

Oil; **^{1}H NMR** (400 MHz, $CDCl_3$): δ = 7.80 (d, J = 7.2 Hz, 2H), 7.53-7.52 (m, 1H), 7.45–7.41 (m, 2H), 5.40 (q, J = 5.2 Hz, 1H), 2.52 (d, J = 5.2 Hz, 3H). **^{13}C NMR** (100 MHz, $CDCl_3$): δ = 138.3 (C_q), 132.6 (CH), 129.0 (CH), 126.9 (CH), 29.0 (CH_3). **IR** (ATR): 3292, 1474, 1446, 1409, 1309, 1154, 1090, 753, 688 cm^{-1}. **MS** (ESI) *m/z* (relative intensity): 172 $[M+H]^+$ (30), 194 $[M+Na]^+$ (100). **HR-MS** (ESI) *m/z* calc. for $C_7H_{10}NO_2S$ $[M+H]^+$: 172.0427, found: 172.0427.

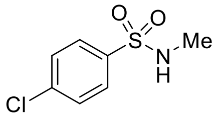

2m

4-chloro-*N*-methylbenzenesulfonamide (2m).

White solid; **M.p.**: 69–70 °C; **^{1}H NMR** (400 MHz, $CDCl_3$): δ = 7.79 (d, J = 8.4 Hz, 2H), 7.47 (d, J = 8.4 Hz, 2H), 5.22 (d, J = 4.0 Hz, 1H), 2.62 (d, J = 5.2 Hz, 3H). **^{13}C NMR** (100 MHz, $CDCl_3$): δ = 139.2 (C_q), 137.2 (C_q), 129.4 (CH), 128.7 (CH), 29.2 (CH_3). **IR** (ATR): 3302, 1585, 1476, 1406, 1318, 1157, 1087, 826 cm^{-1}. **MS** (ESI) *m/z* (relative intensity): 206 $[M+H]^+$ (20), 228 $[M+Na]^+$ (100). **HR-MS** (ESI) *m/z* calc. for $C_7H_9ClNO_2S$ $[M+H]^+$: 206.0037, found: 206.0038.

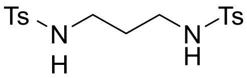

2n

***N,N′*-(propane-1,3-diyl)bis(4-methylbenzenesulfonamide) (2n)**.

White solid; **M.p.**: 141–143 °C; **^{1}H NMR** (400 MHz, $CDCl_3$): δ = 7.72 (d, J = 8.4 Hz, 4H), 7.29 (d, J = 8.4 Hz, 4H), 3.00 (q, J = 6.4 Hz, 4H), 2.42 (s, 6H), 1.69–1.62 (m, 2H).

^{13}C NMR (100 MHz, $CDCl_3$): δ = 143.7 (C_q), 136.9 (C_q), 129.9 (CH), 127.2 (CH), 40.0 (CH_2), 29.9 (CH_2), 21.6 (CH_3). **IR** (ATR): 3269, 2930, 1595, 1493, 1462, 1432, 1320, 1152, 820 cm^{-1}. **MS** (ESI) *m/z* (relative intensity): 383 $[M+H]^+$ (70), 405 $[M+Na]^+$ (100). **HR-MS** (ESI) *m/z* calc. for $C_{17}H_{23}N_2O_4S_2$ $[M+H]^+$: 383.1094, found: 383.1098.

3. General Procedure for Electrochemical C–H Amidation of Heteroarenes

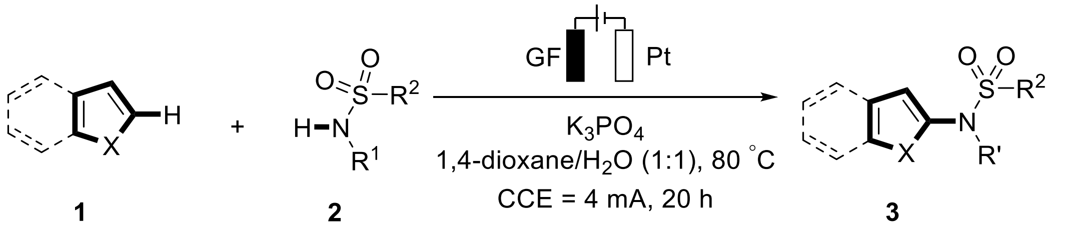

The electrocatalysis was carried out in an undivided cell under air with a graphite felt (GF) anode (10 mm × 15 mm × 6 mm) and a platinum cathode (10 mm × 15 mm × 0.25 mm). Heteroarene **1** (0.4 mmol, 1.0 equiv), sulfonamide **2** (0.8 mmol, 2.0 equiv) and K_3PO_4 (212 mg, 2.0 equiv, 0.25 M) were dissolved in a mixture of 1,4-dioxane/H_2O (1:1, 4 mL). Electrocatalysis was performed at 80 °C with a constant current of 4.0 mA maintained for 20 h. The GF anode was washed with ethyl acetate (3 × 5 mL) in an ultrasonic bath and the solvent was transfered to a round bottom flask. Silica was added to the flask and all volatiles were evaporated under vacuum. Purification was performed by flash column chromatography on silica gel using *n*-hexane/EtOAc as the eluent to give the corresponding products **3**.

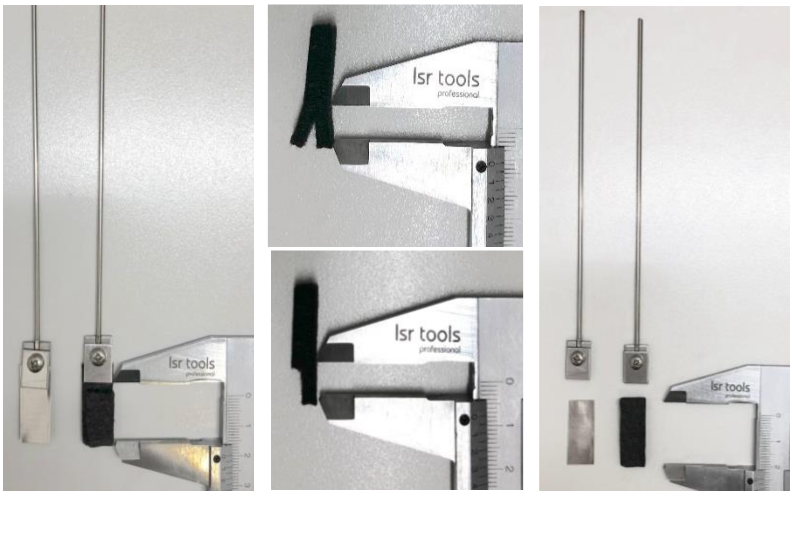

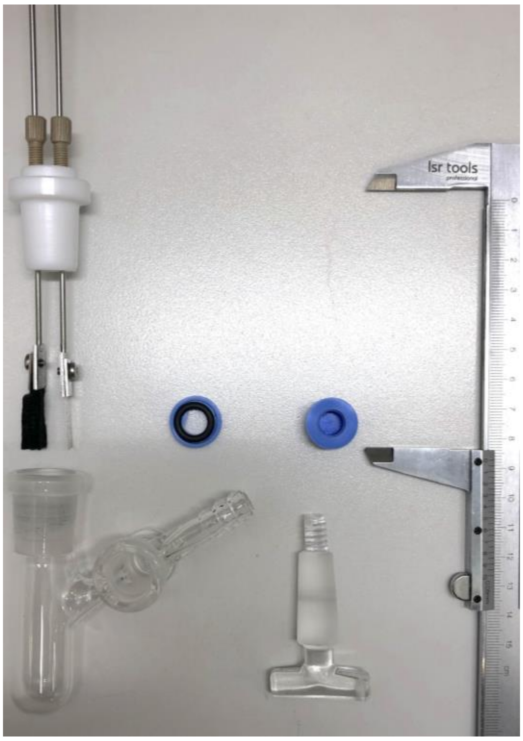

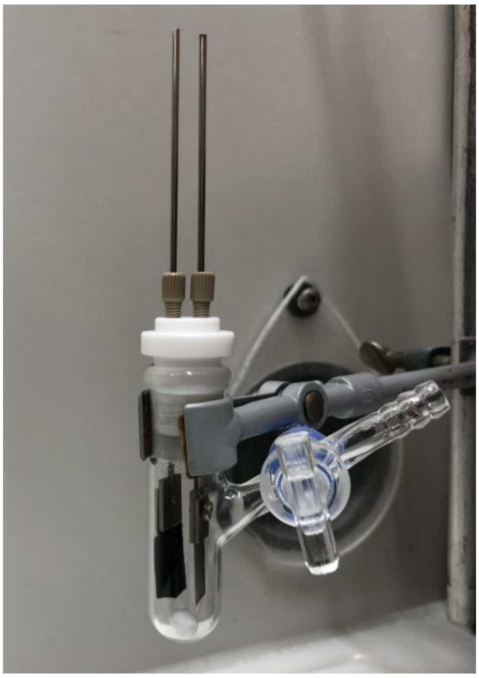

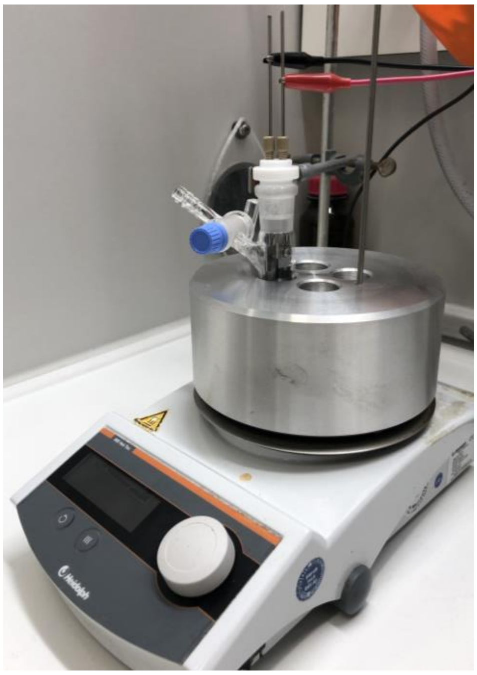

4. Characterization Data of Products 3

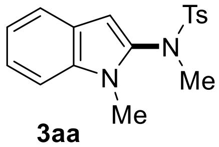

3aa

***N*,4-dimethyl-*N*-(1-methyl-1*H*-indol-2-yl)benzenesulfonamide (3aa)**

The general procedure was followed using **1a** (65.5 mg, 0.50 mmol) and **2a** (185 mg, 1.0 mmol). Purification by column chromatography on silica gel (*n*-hexane/EtOAc =

3:1) yielded **3aa** (107 mg, 68%) as a white solid. **M.p.**: 99–101 °C. **^{1}H NMR** (400 MHz, $CDCl_3$): δ = 7.67 (d, J = 8.0 Hz, 2H), 7.54 (d, J = 8.0 Hz, 1H), 7.40–7.30 (m, 4H), 7.16 (t, J = 7.2 Hz, 1H), 5.87 (s, 1H), 3.86 (s, 3H), 3.23 (s, 3H), 2.52 (s, 3H). **^{13}C NMR** (100 MHz, $CDCl_3$): δ = 144.2 (C_q), 136.9 (C_q), 135.1 (C_q), 132.8 (C_q), 129.5 (CH), 128.7 (CH), 125.9 (C_q), 122.5 (CH), 120.8 (CH), 119.9 (CH), 110.0 (CH), 96.5 (CH), 40.4 (CH_3), 29.5 (CH_3), 21.7 (CH_3). **IR** (ATR): 1596, 1540, 1468, 1391, 1317, 1159, 1087, 872, 812 cm^{-1}. **MS** (ESI) *m/z* (relative intensity): 315 $[M+H]^+$ (80), 337 $[M+Na]^+$ (100). **HR-MS** (ESI) *m/z* calc. for $C_{17}H_{19}N_2O_2S$ $[M+H]^+$: 315.1162, found: 315.1162.

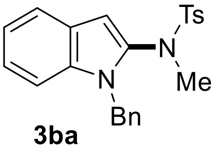

3ba

***N*-(1-benzyl-1*H*-indol-2-yl)-*N*,4-dimethylbenzenesulfonamide (3ba)**

The general procedure was followed using **1b** (104 mg, 0.50 mmol) and **2a** (185 mg, 1.0 mmol). Purification by column chromatography on silica gel (*n*-hexane/EtOAc = 3:1) yielded **3ba** (101 mg, 52%) as a white solid. **M.p.**: 171–173 °C. **^{1}H NMR** (400 MHz, $CDCl_3$): δ = 7.71 (d, J = 8.0 Hz, 2H), 7.57 (d, J = 8.0 Hz, 1H), 7.39–7.24 (m, 7H), 7.18–7.15 (m, 3H), 5.92 (s, 1H), 5.62 (s, 2H), 3.01 (s, 3H), 2.53 (s, 3H). **^{13}C NMR** (100 MHz, $CDCl_3$): δ = 144.3 (C_q), 138.0 (C_q), 136.8 (C_q), 134.8 (C_q), 133.0 (C_q), 129.5 (CH), 128.7 (CH), 127.3 (CH), 126.8 (CH), 126.1 (C_q), 122.8 (CH), 121.0 (CH), 120.1 (CH), 110.7 (CH), 97.7 (CH), 46.4 (CH_2), 40.4 (CH_3), 21.7 (CH_3). **IR** (ATR): 1594, 1531, 1460, 1397, 1162, 810, 767, 724 cm^{-1}. **MS** (ESI) *m/z* (relative intensity): 391 $[M+H]^+$ (40), 413 $[M+Na]^+$ (100). **HR-MS** (ESI) *m/z* calc. for $C_{23}H_{23}N_2O_2S$ $[M+H]^+$: 391.1475, found: 391.1479.

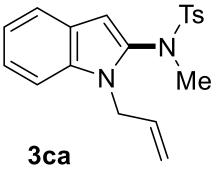

3ca

***N*-(1-allyl-1*H*-indol-2-yl)-*N*,4-dimethylbenzenesulfonamide (3ca)**

The general procedure was followed using **1c** (78.5 mg, 0.50 mmol) and **2a** (185 mg, 1.0 mmol). Purification by column chromatography on silica gel (*n*-hexane/EtOAc = 3:1) yielded **3ca** (85 mg, 50%) as a yellow oil. **^{1}H NMR** (400 MHz, $CDCl_3$): δ = 7.63

(d, J = 8.8 Hz, 2H), 7.47 (d, J = 8.0 Hz, 1H), 7.34–7.31 (m, 3H), 7.22 (t, J = 7.2 Hz, 1H), 7.08 (t, J = 8.0 Hz, 1H), 6.02–5.96 (m, 1H), 5.80 (s, 1H), 5.15 (dd, J = 10.4, 1.6 Hz, 1H), 5.01 (dd, J = 17.2, 1.2 Hz, 1H), 4.91 (s, 2H), 3.15 (s, 3H), 2.47 (s, 3H). **^{13}C NMR** (100 MHz, $CDCl_3$): δ = 144.3 (C_q), 136.5 (C_q), 134.6 (C_q), 134.0 (CH), 133.0 (C_q), 129.6 (CH), 128.8 (CH), 126.1 (C_q), 122.7 (CH), 121.0 (CH), 120.1 (CH), 116.6 (C_q), 110.8 (CH), 97.4 (CH), 45.3 (CH_2), 40.8 (CH_3), 21.8 (CH_3). **IR** (ATR): 1595, 1420, 1357, 1220, 1165, 1090, 873, 769, 736 cm^{-1}. **MS** (ESI) *m/z* (relative intensity): 341 $[M+H]^+$ (97), 363 $[M+Na]^+$ (100). **HR-MS** (ESI) *m/z* calc. for $C_{19}H_{21}N_2O_2S$ $[M+H]^+$: 341.1318, found: 341.1321.

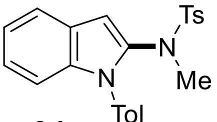

3da

N,4-dimethyl-N-(1-(p-tolyl)-1H-indol-2-yl)benzenesulfonamide (3da)

The general procedure was followed using **1d** (103.5 mg, 0.50 mmol) and **2a** (185 mg, 1.0 mmol). Purification by column chromatography on silica gel (*n*-hexane/EtOAc = 3:1) yielded **3da** (89.7 mg, 46%) as a white solid. **M.p.**: 143–145 °C. **^{1}H NMR** (400 MHz, $CDCl_3$): δ = 7.67 (d, J = 8.8 Hz, 2H), 7.57 (d, J = 7.6 Hz, 1H), 7.36–7.33 (m, 6H), 7.21–7.13 (m, 3H), 6.12 (s, 1H), 3.08 (s, 3H), 2.49 (s, 6H, 2Me). **^{13}C NMR** (100 MHz, $CDCl_3$): δ = 144.0 (C_q), 137.9 (C_q), 136.8 (C_q), 136.0 (C_q), 134.6 (C_q), 133.9 (C_q), 130.3 (CH), 129.6 (CH), 128.4 (CH), 128.1 (CH), 126.1 (C_q), 123.1 (CH), 120.9 (CH), 120.6 (CH), 111.1 (CH), 98.7 (CH), 40.3 (CH_3), 21.7 (CH_3), 21.4 (CH_3). **IR** (ATR): 1596, 1547, 1514, 1452, 1375, 1342, 1156, 1084, 876, 752 cm^{-1}. **MS** (ESI) *m/z* (relative intensity): 391 $[M+H]^+$ (100), 413 $[M+Na]^+$ (75). **HR-MS** (ESI) *m/z* calc. for $C_{23}H_{23}N_2O_2S$ $[M+H]^+$: 391.1475, found: 391.1478.

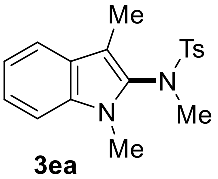

3ea

N-(1,3-dimethyl-1H-indol-2-yl)-N,4-dimethylbenzenesulfonamide (3ea)

The general procedure was followed using **1e** (72.5 mg, 0.50 mmol) and **2a** (185 mg, 1.0 mmol). Purification by column chromatography on silica gel (*n*-hexane/EtOAc =

3:1) yielded **3ea** (131 mg, 80%) as a white solid. **M.p.**: 131–132 °C. **^{1}H NMR** (400 MHz, $CDCl_3$): δ = 7.74 (d, J = 8.8 Hz, 2H), 7.53 (d, J = 8.0 Hz, 1H), 7.37–7.34 (m, 4H), 7.19–7.15 (m, 1H), 3.77 (s, 3H), 3.67 (s, 3H), 2.51 (s, 3H), 1.65 (s, 3H). **^{13}C NMR** (100 MHz, $CDCl_3$): δ = 144.0 (C_q), 135.7 (C_q), 134.9 (C_q), 132.2 (C_q), 129.8 (CH), 127.8 (CH), 126.6 (C_q), 122.7 (CH), 119.09 (CH), 119.05 (CH), 109.7 (CH), 106.7 (C_q), 38.9 (CH_3), 29.4 (CH_3), 21.6 (CH_3), 8.0 (CH_3). **IR** (ATR): 2919, 1595, 1470, 1340, 1153, 1089, 903, 773, 739 cm^{-1}. **MS** (ESI) *m/z* (relative intensity): 329 $[M+H]^+$ (100), 351 $[M+Na]^+$ (97). **HR-MS** (ESI) *m/z* calc. for $C_{18}H_{21}N_2O_2S$ $[M+H]^+$: 329.1318, found: 329.1321.

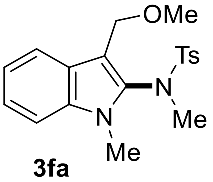

3fa

***N*-[3-(methoxymethyl)-1-methyl-1*H*-indol-2-yl]-*N*,4-dimethylbenzenesulfonamide (3fa)**

The general procedure was followed using **1f** (87.5 mg, 0.50 mmol) and **2a** (185 mg, 1.0 mmol). Purification by column chromatography on silica gel (*n*-hexane/EtOAc = 3:1) yielded **3fa** (98 mg, 55%) as a white solid. **M.p.**: 105–107 °C. **^{1}H NMR** (400 MHz, $CDCl_3$): δ = 7.68 (d, J = 8.8 Hz, 2H), 7.64 (d, J = 8.0 Hz, 1H), 7.35–7.27 (m, 4H), 7.17–7.13 (m, 1H), 4.05 (d, J = 11.6 Hz, 1H), 3.72 (s, 3H), 3.56 (d, J = 11.6 Hz, 1H), 3.36 (s, 3H), 3.14 (s, 3H), 2.48 (s, 3H). **^{13}C NMR** (100 MHz, $CDCl_3$): δ = 144.2 (C_q), 135.4 (C_q), 135.0 (C_q), 133.9 (C_q), 129.9 (CH), 127.9 (CH), 126.1 (C_q), 123.1 (CH), 120.1 (CH), 119.8 (CH), 109.9 (CH), 108.1 (C_q), 64.3 (CH_2), 57.8 (CH_3), 40.0 (CH_3), 29.6 (CH_3), 21.7 (CH_3). **IR** (ATR): 1594, 1568, 1471, 1399, 1345, 1162, 1088, 910, 777 cm^{-1}. **MS** (ESI) *m/z* (relative intensity): 381 $[M+Na]^+$ (100). **HR-MS** (ESI) *m/z* calc. for $C_{19}H_{23}N_2O_3S$ $[M+H]^+$: 359.1424, found: 359.1420.

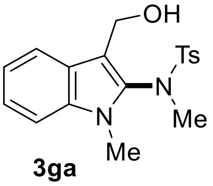

3ga

***N*-[3-(hydroxymethyl)-1-methyl-1*H*-indol-2-yl]-*N*,4-dimethylbenzenesulfonamide (3ga)**

The general procedure was followed using **1g** (80.5 mg, 0.50 mmol) and **2a** (185 mg, 1.0 mmol). Purification by column chromatography on silica gel (*n*-hexane/EtOAc =

2:1) yielded **3ga** (77mg, 45%) as a colorless oil. **^{1}H NMR** (400 MHz, $CDCl_3$): δ = 7.67 (d, J = 8.0 Hz, 2H), 7.32–7.28 (m, 5H), 7.19 (t, J = 6.4 Hz, 1H), 5.07 (s, 1H), 4.45 (d, J = 12.4 Hz, 1H), 4.13 (d, J = 12.4 Hz, 1H), 3.49 (s, 3H), 3.36 (s, 3H), 2.48 (s, 3H). **^{13}C NMR** (100 MHz, $CDCl_3$): δ = 144.5 (C_q), 135.4 (C_q), 135.0 (C_q), 132.9 (C_q), 129.9 (CH), 127.6 (CH), 125.4 (C_q), 123.2 (CH), 120.2 (CH), 119.6 (CH), 111.5 (C_q), 109.8 (CH), 54.8 (CH_2), 39.7 (CH_3), 29.2 (CH_3), 21.7 (CH_3). **IR** (ATR): 2916, 1597, 1567, 1471, 1344, 1160, 1060, 905, 816, 773 cm^{-1}. **MS** (ESI) *m/z* (relative intensity): 367 $[M+Na]^+$ (100). **HR-MS** (ESI) *m/z* calc. for $C_{18}H_{21}N_2O_3S$ $[M+Na]^+$: 367.1087, found: 367.1080.

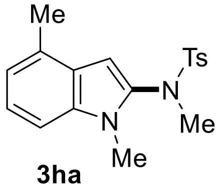

3ha

***N*-(1,4-dimethyl-1*H*-indol-2-yl)-*N*,4-dimethylbenzenesulfonamide (3ha)**

The general procedure was followed using **1h** (72.5 mg, 0.50 mmol) and **2a** (185 mg, 1.0 mmol). Purification by column chromatography on silica gel (*n*-hexane/EtOAc = 3:1) yielded **3ha** (128 mg, 78%) as a colorless oil. **^{1}H NMR** (400 MHz, $CDCl_3$): δ = 7.70 (d, J = 8.0 Hz, 2H), 7.38 (d, J = 7.6 Hz, 2H), 7.25 (d, J = 4.0 Hz, 2H), 5.98 (t, J = 4.4 Hz, 1H), 5.90 (s, 1H), 3.85 (s, 3H), 3.25 (s, 3H), 2.54 (s, 3H), 2.49 (s, 3H). **^{13}C NMR** (100 MHz, $CDCl_3$): δ = 144.2 (C_q), 136.4 (C_q), 134.8 (C_q), 132.8 (C_q), 130.2 (C_q), 129.4 (CH), 128.8 (CH), 125.8 (C_q), 122.6 (CH), 120.1 (CH), 107.6 (CH), 95.2 (CH), 40.4 (CH_3), 29.6 (CH_3), 21.7 (CH_3), 18.7 (CH_3). **IR** (ATR): 1597, 1537, 1495, 1454, 1347, 1156, 1046, 861, 781 cm^{-1}. **MS** (ESI) *m/z* (relative intensity): 329 $[M+H]^+$ (100), 351 $[M+Na]^+$ (85). **HR-MS** (ESI) *m/z* calc. for $C_{18}H_{21}N_2O_2S$ $[M+H]^+$: 329.1318, found: 329.1321.

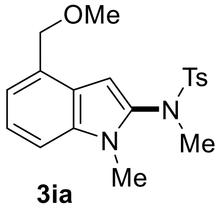

3ia

***N*-[4-(methoxymethyl)-1-methyl-1*H*-indol-2-yl]-*N*,4-dimethylbenzenesulfonamide (3ia)**

The general procedure was followed using **1i** (87.5 mg, 0.50 mmol) and **2a** (185 mg, 1.0 mmol). Purification by column chromatography on silica gel (*n*-hexane/EtOAc = 3:1) yielded **3ia** (116 mg, 65%) as a yellow solid. **M.p.**: 73–75 °C. **^{1}H NMR** (400 MHz, $CDCl_3$): δ = 7.64 (d, J = 8.4 Hz, 2H), 7.34 (d, J = 8.8 Hz, 3H), 7.27 (t, J = 8.0 Hz, 1H), 7.10 (d, J = 7.2 Hz, 1H), 5.98 (s, 1H), 4.67 (s, 2H), 3.84 (s, 3H), 3.35 (s, 3H), 3.22 (s, 3H), 2.50 (s, 3H). **^{13}C NMR** (100 MHz, $CDCl_3$): δ = 144.3 (C_q), 136.9 (C_q), 135.2 (C_q), 132.6 (C_q), 129.9 (C_q), 129.4 (CH), 128.8 (CH), 124.8 (C_q), 122.3 (CH), 119.7 (CH), 109.8 (CH), 95.3 (CH), 73.2 (CH_2), 57.8 (CH_3), 40.4 (CH_3), 29.6 (CH_3), 21.7 (CH_3). **IR** (ATR): 2929, 1597, 1541, 1495, 1453, 1347, 1088, 881, 776 cm^{-1}. **MS** (ESI) *m/z* (relative intensity): 359 $[M+H]^+$ (75), 381 $[M+Na]^+$ (100). **HR-MS** (ESI) *m/z* calc. for $C_{19}H_{23}N_2O_3S$ $[M+H]^+$: 359.1424, found: 359.1427.

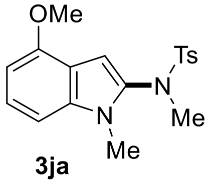

3ja

***N*-(4-methoxy-1-methyl-1*H*-indol-2-yl)-*N*,4-dimethylbenzenesulfonamide (3ja)**

The general procedure was followed using **1j** (80.5 mg, 0.50 mmol) and **2a** (185 mg, 1.0 mmol). Purification by column chromatography on silica gel (*n*-hexane/EtOAc = 3:1) yielded **3ja** (111 mg, 64%) as a yellow solid. **M.p.**: 122–123 °C. **^{1}H NMR** (400 MHz, $CDCl_3$): δ = 7.66 (d, J = 7.6 Hz, 2H), 7.35 (d, J = 7.6 Hz, 2H), 7.22 (dt, J = 8.8, 0.8 Hz, 1H), 7.00 (d, J = 8.4 Hz, 1H), 6.56 (d, J = 8.0 Hz, 1H), 5.99 (s, 1H), 3.93 (s, 3H), 3.82 (s, 3H), 3.19 (s, 3H), 2.50 (s, 3H). **^{13}C NMR** (100 MHz, $CDCl_3$): δ = 153.3 (C_q), 144.2 (C_q), 136.4 (C_q), 135.5 (C_q), 132.7 (C_q), 129.5 (CH), 128.6 (CH), 123.3 (CH), 116.6 (C_q), 103.4 (CH), 99.7 (CH), 93.9 (CH), 55.4 (CH_3), 40.4 (CH_3), 29.8 (CH_3), 21.7 (CH_3). **IR** (ATR): 1580, 1535, 1499, 1454, 1353, 1252, 1160, 872, 760 cm^{-1}. **MS** (ESI) *m/z* (relative intensity): 345 $[M+H]^+$ (95), 367 $[M+Na]^+$ (100). **HR-MS** (ESI) *m/z* calc. for $C_{18}H_{21}N_2O_3S$ $[M+H]^+$: 345.1267, found: 345.1271.

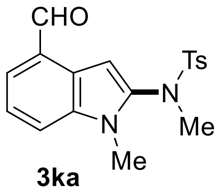

3ka

***N*-(4-formyl-1-methyl-1*H*-indol-2-yl)-*N*,4-dimethylbenzenesulfonamide (3ka)**

The general procedure was followed using **1k** (79.5 mg, 0.50 mmol) and **2a** (185 mg, 1.0 mmol). Purification by column chromatography on silica gel (*n*-hexane/EtOAc = 3:1) yielded **3ka** (95.8 mg, 56%) as a yellow solid. **M.p.**: 144–146 °C. **^{1}H NMR** (400 MHz, $CDCl_3$): δ = 10.15 (s, 1H), 7.66–7.60 (m, 4H), 7.42 (t, *J* = 8.0 Hz, 1H), 7.34 (d, *J* = 8.0 Hz, 2H), 6.70 (s, 1H), 3.91 (s, 3H), 3.20 (s, 3H), 2.49 (s, 3H). **^{13}C NMR** (100 MHz, $CDCl_3$): δ = 193.1 (CH), 144.7 (C_q), 140.4 (C_q), 135.7 (C_q), 132.2 (C_q), 129.6 (CH), 128.7 (CH), 128.3 (C_q), 128.2 (CH), 123.6 (C_q), 121.8 (CH), 116.4 (CH), 97.2 (CH), 40.2 (CH_3), 29.9 (CH_3), 21.7 (CH_3). **IR** (ATR): 1679, 1595, 1570, 1524, 1346, 1158, 1010, 870, 729 cm^{-1}. **MS** (ESI) *m/z* (relative intensity): 343 $[M+H]^+$ (60), 365 $[M+Na]^+$ (100). **HR-MS** (ESI) *m/z* calc. for $C_{18}H_{19}N_2O_3S$ $[M+H]^+$: 343.1111, found: 343.1114.

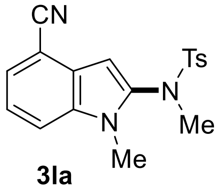

3la

***N*-(4-cyano-1-methyl-1*H*-indol-2-yl)-*N*,4-dimethylbenzenesulfonamide (3la)**

The general procedure was followed using **1l** (78 mg, 0.50 mmol) and **2a** (185 mg, 1.0 mmol). Purification by column chromatography on silica gel (*n*-hexane/EtOAc = 2:1) yielded **3la** (98 mg, 58%) as a yellow solid. **M.p.**: 128–129 °C. **^{1}H NMR** (400 MHz, $CDCl_3$): δ = 7.57–7.54 (m, 3H), 7.43 (dd, *J* = 7.6, 0.8 Hz, 1H), 7.33 (dd, *J* = 8.8, 0.8 Hz, 2H), 7.27–7.24 (m, 1H), 6.01 (d, *J* = 0.8 Hz, 1H), 3.84 (s, 3H), 3.15 (s, 3H), 2.46 (s, 3H). **^{13}C NMR** (100 MHz, $CDCl_3$): δ = 144.9 (C_q), 139.7 (C_q), 134.7 (C_q), 132.0 (C_q), 129.7 (CH), 128.6 (CH), 127.3 (C_q), 125.5 (CH), 122.0 (CH), 118.6 (C_q), 114.8 (CH), 103.0 (C_q), 95.3 (CH), 40.2 (CH_3), 30.0 (CH_3), 21.7 (CH_3). **IR** (ATR): 2222, 1597, 1532, 1456, 1420, 1350, 1162, 857, 772, 728 cm^{-1}. **MS** (ESI) *m/z* (relative intensity): 340 $[M+H]^+$ (100), 362 $[M+Na]^+$ (40). **HR-MS** (ESI) *m/z* calc. for $C_{18}H_{18}N_3O_2S$ $[M+H]^+$: 340.1114, found: 340.1114.

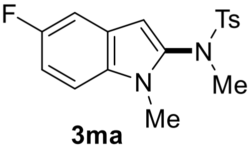

3ma

N-(5-fluoro-1-methyl-1H-indol-2-yl)-N,4-dimethylbenzenesulfonamide (3ma)

The general procedure was followed using **1m** (74.5 mg, 0.50 mmol) and **2a** (185 mg, 1.0 mmol). Purification by column chromatography on silica gel (n-hexane/EtOAc = 2:1) yielded **3ma** (120 mg, 72%) as a white solid. **M.p.**: 116–118 °C. **^{1}H NMR** (400 MHz, $CDCl_3$): δ = 7.59 (d, J = 8.4 Hz, 2H), 7.32 (dd, J = 9.2, 2.4 Hz, 2H), 7.23 (m, 1H), 7.12 (dd, J = 9.6, 2.4 Hz, 1H), 6.99 (dt, J = 9.2, 2.4 Hz, 1H), 5.77 (d, J = 0.8 Hz, 1H), 3.78 (s, 3H), 3.15 (s, 3H), 2.46 (s, 3H). **^{13}C NMR** (100 MHz, $CDCl_3$): δ = 158.3 (d, $^1J_{C–F}$ = 234.0 Hz, C_q), 144.4 (C_q), 138.2 (C_q), 132.7 (C_q), 131.7 (C_q), 129.5 (CH), 128.7 (CH), 125.9 (d, $^3J_{C–F}$ = 10.0 Hz, C_q), 110.8 (CH), 109.9 (d, $^2J_{C–F}$ = 26.0 Hz, CH), 105.5 (d, $^2J_{C–F}$ = 24.0 Hz, CH), 96.5 (d, $^3J_{C–F}$ = 5.0 Hz, CH), 40.2 (CH_3), 29.7 (CH_3), 21.7 (CH_3). **^{19}F NMR** (376 MHz, $CDCl_3$): δ = –124.1. **IR** (ATR): 1597, 1577, 1541, 1484, 1348, 1157, 1055, 864, 770 cm^{-1}. **MS** (ESI) m/z (relative intensity): 333 $[M+H]^+$ (100), 355 $[M+Na]^+$ (98). **HR-MS** (ESI) m/z calc. for $C_{17}H_{18}FN_2O_2S$ $[M+H]^+$: 333.1068, found: 333.1063.

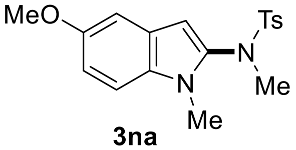

3na

N-(5-methoxy-1-methyl-1H-indol-2-yl)-N,4-dimethylbenzenesulfonamide (3na)

The general procedure was followed using **1n** (80.5 mg, 0.50 mmol) and **2a** (185 mg, 1.0 mmol). Purification by column chromatography on silica gel (n-hexane/EtOAc = 3:1) yielded **3na** (129 mg, 75%) as a white solid. **M.p.**: 122–124 °C. **^{1}H NMR** (400 MHz, $CDCl_3$): δ = 7.63 (d, J = 8.0 Hz, 2H), 7.33 (d, J = 8.0 Hz, 2H), 7.24 (d, J = 8.8 Hz, 1H), 6.96–6.93 (m, 2H), 5.74 (s, 1H), 3.83 (s, 3H), 3.77 (s, 3H), 3.17 (s, 3H), 2.48 (s, 3H). **^{13}C NMR** (100 MHz, $CDCl_3$): δ = 154.4 (C_q), 144.2 (C_q), 137.1 (C_q), 132.9 (C_q), 130.4 (C_q), 129.5 (CH), 128.7 (CH), 126.1 (C_q), 112.9 (CH), 110.8 (CH), 102.5 (CH), 96.2 (CH), 56.0 (CH_3), 40.4 (CH_3), 29.6 (CH_3), 21.7 (CH_3). **IR** (ATR): 1596, 1577, 1532, 1486, 1434, 1396, 1151, 1087, 896, 800 cm^{-1}. **MS** (ESI) m/z (relative intensity): 345 $[M+H]^+$ (80), 367 $[M+Na]^+$ (100). **HR-MS** (ESI) m/z calc. for $C_{18}H_{21}N_2O_3S$ $[M+H]^+$: 345.1267, found: 345.1261.

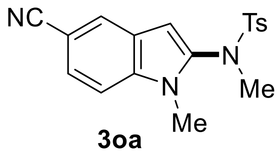

3oa

***N*-(5-cyano-1-methyl-1*H*-indol-2-yl)-*N*,4-dimethylbenzenesulfonamide (3oa)**

The general procedure was followed using **1o** (78.0 mg, 0.50 mmol) and **2a** (185 mg, 1.0 mmol). Purification by column chromatography on silica gel (*n*-hexane/EtOAc = 2:1) yielded **3oa** (115 mg, 68%) as a white solid. **M.p.**: 153–154 °C. **^{1}H NMR** (400 MHz, $CDCl_3$): δ = 7.28 (s, 1H), 7.58 (d, J = 8.0 Hz, 2H), 7.47 (d, J = 8.4 Hz, 1H), 7.40–7.33 (m, 3H), 5.89 (s, 1H), 3.84 (s, 3H), 3.17 (s, 3H), 2.48 (s, 3H). **^{13}C NMR** (100 MHz, $CDCl_3$): δ = 144.8 (C_q), 139.4 (C_q), 136.6 (C_q), 132.3 (C_q), 129.7 (CH), 128.7 (CH), 126.4 (CH), 125.6 (C_q), 125.3 (CH), 120.6 (C_q), 111.0 (CH), 103.0 (C_q), 97.4 (CH), 40.3 (CH_3), 29.9 (CH_3), 21.8 (CH_3). **IR** (ATR): 2230, 1616, 1595, 1541, 1467, 1349, 1155, 1055, 898, 786 cm^{-1}. **MS** (ESI) *m/z* (relative intensity): 362 $[M+Na]^+$ (100). **HR-MS** (ESI) *m/z* calc. for $C_{18}H_{18}N_3O_2S$ $[M+H]^+$: 340.1114, found: 340.1110.

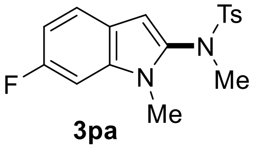

3pa

***N*-(6-fluoro-1-methyl-1*H*-indol-2-yl)-*N*,4-dimethylbenzenesulfonamide (3pa)**

The general procedure was followed using **1p** (74.5 mg, 0.50 mmol) and **2a** (185 mg, 1.0 mmol). Purification by column chromatography on silica gel (*n*-hexane/EtOAc = 2:1) yielded **3pa** (120 mg, 72%) as a white solid. **M.p.**: 141–143 °C. **^{1}H NMR** (400 MHz, $CDCl_3$): δ = 7.60 (d, J = 8.0 Hz, 2H), 7.38 (dd, J = 8.8, 5.2 Hz, 1H), 7.31 (d, J = 8.4 Hz, 2H), 6.99 (dd, J = 10.0, 2.4 Hz, 1H), 6.85 (m, 1H), 5.80 (s, 1H), 3.73 (s, 3H), 3.15 (s, 3H), 2.46 (s, 3H). **^{13}C NMR** (100 MHz, $CDCl_3$): δ = 160.2 (d, $^{1}J_{C–F}$ = 237.1 Hz, C_q), 144.3 (C_q), 137.2 (d, $^{4}J_{C–F}$ = 3.6 Hz, C_q), 135.1 (d, $^{3}J_{C–F}$ = 12.2 Hz, C_q), 132.8 (C_q), 129.5 (CH), 128.6 (CH), 122.2 (C_q), 121.7 (d, $^{3}J_{C–F}$ = 10.0 Hz, CH), 108.7 (d, $^{2}J_{C-F}$ = 24.4 Hz, CH), 96.8 (CH), 96.3 (d, $^{2}J_{C–F}$ = 26.1 Hz, CH), 40.3 (CH_3), 29.6 (CH_3), 21.7 (CH_3). **^{19}F NMR** (376 MHz, $CDCl_3$): δ = –119.1. **IR** (ATR): 1620, 1545, 1474, 1452, 1350, 1160, 1047, 835, 809 cm^{-1}. **MS** (ESI) *m/z* (relative intensity): 333 $[M+H]^+$ (100), 355 $[M+Na]^+$ (97). **HR-MS** (ESI) *m/z* calc. for $C_{17}H_{18}FN_2O_2S$ $[M+H]^+$: 333.1068, found: 333.1066.

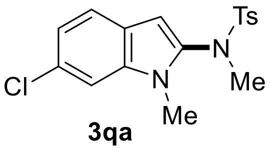

3qa

_N_-(6-chloro-1-methyl-1_H_-indol-2-yl)-_N_,4-dimethylbenzenesulfonamide (3qa)

The general procedure was followed using **1q** (82.5 mg, 0.50 mmol) and **2a** (185 mg, 1.0 mmol). Purification by column chromatography on silica gel (*n*-hexane/EtOAc = 3:1) yielded **3qa** (101 mg, 58%) as a white solid. **M.p.**: 128–129 °C. **^{1}H NMR** (400 MHz, $CDCl_3$): δ = 7.58 (d, *J* = 8.4 Hz, 2H), 7.37 (dd, *J* = 8.4, 0.4 Hz, 1H), 7.32–7.30 (m, 3H), 7.04 (dd, *J* = 8.4, 2.0 Hz, 1H), 5.79 (s, 1H), 3.74 (s, 3H), 3.15 (s, 3H), 2.46 (s, 3H). **^{13}C NMR** (100 MHz, $CDCl_3$): δ = 144.4 (C_q), 137.6 (C_q), 135.5 (C_q), 132.7 (C_q), 129.5 (CH), 128.7 (CH), 128.4 (C_q), 124.4 (C_q), 121.8 (CH), 120.7 (CH), 110.0 (CH), 96.8 (CH), 40.3 (CH_3), 29.6 (CH_3), 21.7 (CH_3). **IR** (ATR): 1594, 1537, 1454, 1351, 1160, 865, 799, 744 cm^{-1}. **MS** (ESI) *m/z* (relative intensity): 349 $[M+H]^+$ (100), 371 $[M+Na]^+$ (75). **HR-MS** (ESI) *m/z* calc. for $C_{17}H_{18}{}^{35}ClN_2O_2S$ $[M+H]^+$: 349.0772, found: 349.0769.

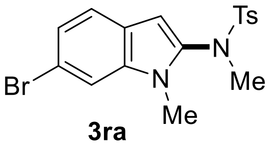

3ra

_N_-(6-bromo-1-methyl-1_H_-indol-2-yl)-_N_,4-dimethylbenzenesulfonamide (3ra)

The general procedure was followed using **1r** (104 mg, 0.50 mmol) and **2a** (185 mg, 1.0 mmol). Purification by column chromatography on silica gel (*n*-hexane/EtOAc = 3:1) yielded **3ra** (98 mg, 50%) as a white solid. **M.p.**: 146–148 °C. **^{1}H NMR** (400 MHz, $CDCl_3$): δ = 7.59–7.56 (m, 3H), 7.33–7.30 (m, 3H), 7.18 (d, *J* = 8.8 Hz, 1H), 5.72 (d, *J* = 0.8 Hz, 1H), 3.77 (s, 3H), 3.14 (s, 3H), 2.46 (s, 3H). **^{13}C NMR** (100 MHz, $CDCl_3$): δ = 144.5 (C_q), 138.0 (C_q), 133.7 (C_q), 132.5 (C_q), 129.6 (CH), 128.7 (CH), 127.4 (C_q), 125.4 (CH), 123.2 (CH), 113.2 (C_q), 111.5 (CH), 96.1 (CH), 40.3 (CH_3), 29.7 (CH_3), 21.7 (CH_3). **IR** (ATR): 1597, 1538, 1467, 1431, 1351, 1161, 1052, 804 cm^{-1}. **MS** (ESI) *m/z* (relative intensity): 395 $[M+H]^+$ (90), 417 $[M+Na]^+$ (100). **HR-MS** (ESI) *m/z* calc. for $C_{17}H_{18}{}^{79}BrN_2O_2S$ $[M+H]^+$: 393.0267, found: 393.0249.

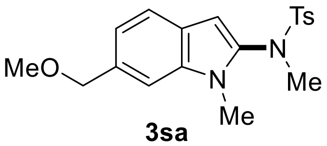

3sa

N-[6-(methoxymethyl)-1-methyl-1H-indol-2-yl]-N,4-dimethylbenzenesulfonamide (3sa)

The general procedure was followed using **1s** (87.5 mg, 0.50 mmol) and **2a** (185 mg, 1.0 mmol). Purification by column chromatography on silica gel (*n*-hexane/EtOAc = 3:1) yielded **3sa** (118 mg, 66%) as a yellow oil. **^{1}H NMR** (400 MHz, $CDCl_3$): δ = 7.60 (d, J = 7.0 Hz, 2H), 7.45 (d, J = 7.0 Hz, 1H), 7.34 (s, 1H), 7.30 (d, J = 7.0 Hz, 2H), 7.07 (dd, J = 7.0, 1.5 Hz, 1H), 5.79 (s, 1H), 4.59 (s, 2H), 3.79 (s, 3H), 3.41 (s, 3H), 3.16 (s, 3H), 2.45 (s, 3H). **^{13}C NMR** (100 MHz, $CDCl_3$): δ = 144.2 (C_q), 137.2 (C_q), 135.1 (C_q), 132.8 (C_q), 132.7 (C_q), 129.4 (CH), 128.7 (CH), 125.4 (C_q), 120.7 (CH), 120.2 (CH), 109.4 (CH), 96.5 (CH), 75.4 (CH_2), 57.9 (CH_3), 40.3 (CH_3), 29.5 (CH_3), 21.7 (CH_3). **IR** (ATR): 2932, 1597, 1537, 1468, 1347, 1086, 909, 811, 725 cm^{-1}. **MS** (ESI) *m/z* (relative intensity): 359 $[M+H]^+$ (100), 381 $[M+Na]^+$ (80). **HR-MS** (ESI) *m/z* calc. for $C_{19}H_{23}N_2O_3S$ $[M+H]^+$: 359.1424, found: 359.1422.

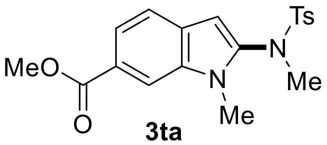

3ta

Methyl 2-[(N,4-dimethylphenyl)sulfonamido]-1-methyl-1H-indole-6-carboxylate (3ta)

The general procedure was followed using **1t** (94.5 mg, 0.50 mmol) and **2a** (185 mg, 1.0 mmol). Purification by column chromatography on silica gel (*n*-hexane/EtOAc = 1:1) yielded **3ta** (93 mg, 50%) as a white solid. **M.p.**: 167–168 °C. **^{1}H NMR** (400 MHz, $CDCl_3$): δ = 8.14 (s, 1H), 7.81 (d, J = 8.0 Hz, 1H), 7.62 (d, J = 8.0 Hz, 2H), 7.52 (d, J = 8.4 Hz, 1H), 7.35 (d, J = 8.4 Hz, 2H), 5.88 (s, 1H), 3.98 (s, 3H), 3.88 (s, 3H), 3.20 (s, 3H), 2.50 (s, 3H). **^{13}C NMR** (100 MHz, $CDCl_3$): δ = 168.1 (C=O), 144.6 (C_q), 139.9 (C_q), 134.5 (C_q), 132.6 (C_q), 129.62 (CH), 129.58 (C_q), 128.7 (CH), 124.1 (C_q), 121.0 (CH), 120.4 (CH), 112.5 (CH), 96.9 (CH), 52.1 (CH_3), 40.2 (CH_3), 29.8 (CH_3), 21.8 (CH_3). **IR** (ATR): 1702, 1619, 1594, 1529, 1468, 1436, 1346, 1259, 1156, 880, 746 cm^{-1}. **MS** (ESI) *m/z* (relative intensity): 373 $[M+H]^+$ (73), 395 $[M+Na]^+$ (100). **HR-MS** (ESI) *m/z* calc. for $C_{19}H_{21}N_2O_4S$ $[M+H]^+$: 373.1217, found: 373.1216.

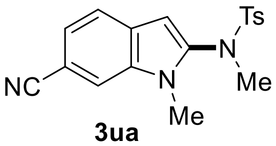

3ua

***N*-(6-cyano-1-methyl-1*H*-indol-2-yl)-*N*,4-dimethylbenzenesulfonamide (3ua)**

The general procedure was followed using **1u** (78 mg, 0.50 mmol) and **2a** (185 mg, 1.0 mmol). Purification by column chromatography on silica gel (*n*-hexane/EtOAc = 2:1) yielded **3ua** (102 mg, 60%) as a white solid. **M.p.**: 144–145 °C. **^{1}H NMR** (400 MHz, $CDCl_3$): δ = 7.66 (s, 1H), 7.58 (d, J = 8.0 Hz, 2H), 7.53 (d, J = 8.4 Hz, 1H), 7.35–7.30 (m, 3H), 5.89 (d, J = 0.8 Hz, 1H), 3.84 (s, 3H), 3.17 (s, 3H), 2.48 (s, 3H). **^{13}C NMR** (100 MHz, $CDCl_3$): δ = 144.7 (C_q), 140.6 (C_q), 134.0 (C_q), 132.3 (C_q), 129.7 (CH), 129.1 (C_q), 128.7 (CH), 122.8 (CH), 121.6 (CH), 120.6 (C_q), 114.9 (CH), 104.9 (C_q), 97.3 (CH), 40.1 (CH_3), 29.9 (CH_3), 21.8 (CH_3). **IR** (ATR): 2213, 1595, 1532, 1471, 1331, 1158, 1087, 873, 815, 757 cm^{-1}. **MS** (ESI) *m/z* (relative intensity): 340 $[M+H]^+$ (100), 362 $[M+Na]^+$ (90). **HR-MS** (ESI) *m/z* calc. for $C_{18}H_{18}N_3O_2S$ $[M+H]^+$: 340.1114, found: 340.1110.

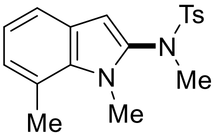

3va

***N*-(1,7-dimethyl-1*H*-indol-2-yl)-*N*,4-dimethylbenzenesulfonamide (3va)**

The general procedure was followed using **1v** (72.5 mg, 0.50 mmol) and **2a** (185 mg, 1.0 mmol). Purification by column chromatography on silica gel (*n*-hexane/EtOAc = 3:1) yielded **3va** (100 mg, 61%) as a white solid. **M.p.**: 190–192 °C. **^{1}H NMR** (400 MHz, $CDCl_3$): δ = 7.61 (d, J = 8.4 Hz, 2H), 7.32–7.29 (m, 3H), 6.94 (d, J = 8.4 Hz, 2H), 5.75 (s, 1H), 4.04 (s, 3H), 3.15 (s, 3H), 2.79 (s, 3H), 2.46 (s, 3H). **^{13}C NMR** (100 MHz, $CDCl_3$): δ = 144.2 (C_q), 137.1 (C_q), 134.2 (C_q), 132.9 (C_q), 129.5 (CH), 128.8 (CH), 126.6 (C_q), 125.3 (CH), 121.8 (C_q), 120.0 (CH), 118.9 (CH), 97.0 (CH), 40.5 (CH_3), 32.4 (CH_3), 21.8 (CH_3), 20.2 (CH_3). **IR** (ATR): 2921, 1595, 1547, 1491, 1450, 1339, 1153, 1086, 819, 737 cm^{-1}. **MS** (ESI) *m/z* (relative intensity): 329 $[M+H]^+$ (97), 351 $[M+Na]^+$ (100). **HR-MS** (ESI) *m/z* calc. for $C_{18}H_{21}N_2O_2S$ $[M+H]^+$: 329.1318, found: 329.1316.

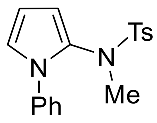

3wa

***N*,4-dimethyl-*N*-(1-phenyl-1*H*-pyrrol-2-yl)benzenesulfonamide (3wa)**

The general procedure was followed using **1w** (71.5 mg, 0.50 mmol) and **2a** (185 mg, 1.0 mmol). Purification by column chromatography on silica gel (*n*-hexane/EtOAc = 3:1) yielded **3wa** (84.8 mg, 52%) as a yellow solid. **M.p.**: 123–125 °C. **^{1}H NMR** (400 MHz, $CDCl_3$): δ = 7.59 (d, J = 8.4 Hz, 2H), 7.44–7.33 (m, 5H), 7.27 (d, J = 8.0 Hz, 2H), 6.77 (dd, J = 3.1, 1.9 Hz, 1H), 6.14 (t, J = 3.6 Hz, 1H), 5.70 (dd, J = 3.8, 1.8 Hz, 1H), 3.02 (s, 3H), 2.43 (s, 3H). **^{13}C NMR** (100 MHz, $CDCl_3$): δ = 143.8 (C_q), 138.8 (C_q), 134.4 (C_q), 129.5 (C_q), 129.4 (CH), 129.1 (CH), 128.4 (CH), 127.3 (CH), 125.8 (CH), 121.0 (CH), 107.6 (CH), 105.7 (CH), 40.3 (CH_3), 21.7 (CH_3). **IR** (ATR): 1594, 1548, 1455, 1342, 1174, 1072, 850, 819, 771 cm^{-1}. **MS** (ESI) *m/z* (relative intensity): 327 $[M+H]^+$ (95), 349 $[M+Na]^+$ (100). **HR-MS** (ESI) *m/z* calc. for $C_{18}H_{19}N_2O_2S$ $[M+H]^+$: 327.1162, found: 327.1160.

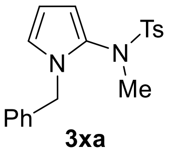

3xa

***N*-(1-benzyl-1*H*-pyrrol-2-yl)-*N*,4-dimethylbenzenesulfonamide (3xa)**

The general procedure was followed using **1x** (78.5 mg, 0.50 mmol) and **2a** (185 mg, 1.0 mmol). Purification by column chromatography on silica gel (*n*-hexane/EtOAc = 3:1) yielded **3xa** (80 mg, 47%) as a white solid. **M.p.**: 117–119 °C. **^{1}H NMR** (400 MHz, $CDCl_3$): δ = 7.65 (d, J = 8.4 Hz, 2H), 7.35–7.27 (m, 5H), 7.20 (d, J = 7.2 Hz, 2H), 6.63 (m, 1H), 6.04 (t, J = 3.6 Hz, 1H), 5.48-5.46 (m, 1H), 5.09 (s, 2H), 2.85 (s, 3H), 2.48 (s, 3H). **^{13}C NMR** (100 MHz, $CDCl_3$): δ = 143.9 (C_q), 138.2 (C_q), 134.4 (C_q), 129.5 (C_q), 129.4 (CH), 128.7 (CH), 128.6 (CH), 127.6 (CH), 127.5 (CH), 120.1 (CH), 106.7 (CH), 104.3 (CH), 49.9 (CH_2), 40.1 (CH_3), 21.7 (CH_3). **IR** (ATR): 2922, 1598, 1541, 1477, 1452, 1342, 1164, 1062, 833, 808 cm^{-1}. **MS** (ESI) *m/z* (relative intensity): 341 $[M+H]^+$ (97), 363 $[M+Na]^+$ (100). **HR-MS** (ESI) *m/z* calc. for $C_{19}H_{21}N_2O_2S$ $[M+H]^+$: 341.1318, found: 341.1318.

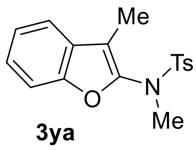

3ya

***N*,4-dimethyl-*N*-(3-methylbenzofuran-2-yl)benzenesulfonamide (3ya)**

The general procedure was followed using **1y** (66.0 mg, 0.50 mmol) and **2a** (185 mg, 1.0 mmol). Purification by column chromatography on silica gel (*n*-hexane/EtOAc = 3:1) yielded **3ya** (95 mg, 60%) as a yellow oil. **^{1}H NMR** (400 MHz, $CDCl_3$): δ = 7.65 (d, J = 8.4 Hz, 2H), 7.50 (d, J = 7.2 Hz, 1H), 7.30–7.21 (m, 5H), 3.19 (s, 3H), 2.44 (s, 3H), 2.24 (s, 3H). **^{13}C NMR** (100 MHz, $CDCl_3$): δ = 151.8 (C_q), 145.3 (C_q), 144.2 (C_q), 134.9 (C_q), 129.7 (CH), 129.4 (C_q), 128.1 (CH), 125.0 (CH), 122.7 (CH), 120.1 (CH), 112.2 (C_q), 111.0 (CH), 37.2 (CH_3), 21.7 (CH_3), 8.2 (CH_3). **IR** (ATR): 2923, 1645, 1597, 1453, 1357, 1172, 1087, 897, 765 cm^{-1}. **MS** (ESI) *m/z* (relative intensity): 316 $[M+H]^+$ (30), 338 $[M+Na]^+$ (100). **HR-MS** (ESI) *m/z* calc. for $C_{17}H_{18}NO_3S$ $[M+H]^+$: 316.1002, found: 316.1001.

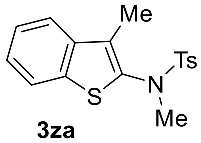

3za

***N*,4-dimethyl-*N*-(3-methylbenzo[b]thiophen-2-yl)benzenesulfonamide (3za)**

The general procedure was followed using **1z** (74.0 mg, 0.50 mmol) and **2a** (185 mg, 1.0 mmol). Purification by column chromatography on silica gel (*n*-hexane/EtOAc = 3:1) yielded **3za** (66 mg, 40%) as a yellow solid. **M.p.**: 102–103 °C. **^{1}H NMR** (400 MHz, $CDCl_3$): δ = 7.70–7.68 (m, 4H), 7.41–7.28 (m, 4H), 3.25 (s, 3H), 2.48 (s, 3H), 2.39 (s, 3H). **^{13}C NMR** (100 MHz, $CDCl_3$): δ = 144.3 (C_q), 138.3 (C_q), 137.43 (C_q), 137.39 (C_q), 134.0 (C_q), 132.1 (C_q), 129.7 (CH), 128.6 (CH), 125.4 (CH), 124.4 (CH), 122.9 (CH), 122.6 (CH), 40.2 (CH_3), 21.8 (CH_3), 12.0 (CH_3). **IR** (ATR): 1597, 1458, 1434, 1350, 1170, 1089, 978, 842, 770 cm^{-1}. **MS** (ESI) *m/z* (relative intensity): 354 $[M+Na]^+$ (100). **HR-MS** (ESI) *m/z* calc. for $C_{17}H_{18}NO_2S_2$ $[M+H]^+$: 332.0773, found: 332.0762.

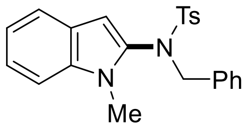

3ab

N-benzyl-4-methyl-N-(1-methyl-1H-indol-2-yl)benzenesulfonamide (3ab)

The general procedure was followed using **1a** (65.5 mg, 0.50 mmol) and **2b** (261 mg, 1.0 mmol). Purification by column chromatography on silica gel (*n*-hexane/EtOAc = 3:1) yielded **3ab** (88 mg, 45%) as a white solid. **M.p.**: 155–157 °C. **^{1}H NMR** (400 MHz, $CDCl_3$): δ = 7.71 (d, J = 7.6 Hz, 2H), 7.52 (d, J = 8.0 Hz, 1H), 7.37 (d, J = 8.0 Hz, 2H), 7.23 (m, 7H), 7.13–7.11 (m, 1H), 5.94 (s, 1H), 5.19 (s, 1H), 4.12 (s, 1H), 3.39 (s, 3H), 2.52 (s, 3H). **^{13}C NMR** (100 MHz, $CDCl_3$): δ = 144.2 (C_q), 135.4 (C_q), 135.2 (C_q), 135.0 (C_q), 134.7 (C_q), 129.6 (CH), 129.4 (CH), 128.6 (CH), 128.5 (CH), 128.3 (CH), 126.0 (C_q), 122.3 (CH), 120.9 (CH), 119.8 (CH), 110.0 (CH), 98.0 (CH), 57.6 (CH_2), 29.0 (CH_3), 21.8 (CH_3). **IR** (ATR): 2962, 1351, 1259, 1161, 1085, 1014, 797 cm^{-1}. **MS** (ESI) *m/z* (relative intensity): 391 $[M+H]^+$ (100), 413 $[M+Na]^+$ (98). **HR-MS** (ESI) *m/z* calc. for $C_{23}H_{23}N_2O_2S$ $[M+H]^+$: 391.1475, found: 391.1476.

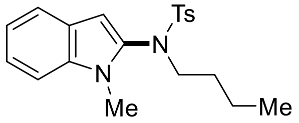

3ac

N-butyl-4-methyl-N-(1-methyl-1H-indol-2-yl)benzenesulfonamide (3ac)

The general procedure was followed using **1a** (65.5 mg, 0.50 mmol) and **2c** (227 mg, 1.0 mmol). Purification by column chromatography on silica gel (*n*-hexane/EtOAc = 3:1) yielded **3ac** (94 mg, 53%) as a white solid. **M.p.**: 81–83 °C. **^{1}H NMR** (400 MHz, $CDCl_3$): δ = 7.62 (d, J = 8.4 Hz, 2H), 7.51 (d, J = 7.6 Hz, 1H), 7.37–7.24 (m, 4H), 7.12 (dt, J = 7.6, 0.8 Hz, 1H), 5.94 (s, 1H), 3.84 (s, 1H), 3.80 (s, 3H), 3.11 (s, 1H), 2.46 (s, 3H), 1.49–1.28 (m, 4H), 0.86 (t, J = 7.2 Hz, 3H). **^{13}C NMR** (100 MHz, $CDCl_3$): δ = 144.0 (C_q), 135.33 (C_q), 135.28 (C_q), 134.2 (C_q), 129.5 (CH), 128.5 (CH), 126.0 (C_q), 122.4 (CH), 120.8 (CH), 119.9 (CH), 110.0 (CH), 97.6 (CH), 57.1 (CH_2), 30.3 (CH_2), 29.4 (CH_3), 21.7 (CH_3), 19.9 (CH_2), 13.7 (CH_3). **IR** (ATR): 2957, 1599, 1538, 1460, 1342, 1162, 1162, 1048, 810 cm^{-1}. **MS** (ESI) *m/z* (relative intensity): 357 $[M+H]^+$ (97), 379 $[M+Na]^+$ (100). **HR-MS** (ESI) *m/z* calc. for $C_{20}H_{25}N_2O_2S$ $[M+H]^+$: 357.1631, found: 357.1635.

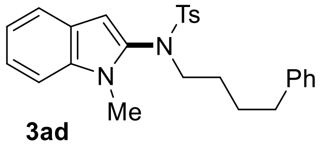

3ad

4-methyl-*N*-(1-methyl-1*H*-indol-2-yl)-*N*-(4-phenylbutyl)benzenesulfonamide (3ad)

The general procedure was followed using **1a** (65.5 mg, 0.50 mmol) and **2d** (303 mg, 1.0 mmol). Purification by column chromatography on silica gel (*n*-hexane/EtOAc = 3:1) yielded **3ad** (91 mg, 42%) as a colorless oil. **^{1}H NMR** (400 MHz, $CDCl_3$): δ = 7.64 (d, J = 8.0 Hz, 2H), 7.55 (d, J = 8.0 Hz, 1H), 7.40–7.25 (m, 6H), 7.22–7.11 (m, 4H), 5.86 (s, 1H), 3.88 (s, 1H), 3.79 (s, 3H), 3.19 (s, 1H), 2.58 (m, 2H), 2.50 (s, 3H), 1.64–1.45 (m, 4H). **^{13}C NMR** (100 MHz, $CDCl_3$): δ = 144.1 (C_q), 142.0 (C_q), 135.4 (C_q), 135.2 (C_q), 134.2 (C_q), 129.5 (CH), 128.5 (CH), 128.4 (CH), 126.0 (C_q), 125.95 (CH), 122.5 (CH), 120.9 (CH), 120.0 (CH), 110.0 (CH), 97.7 (CH), 53.1 (CH_2), 35.4 (CH_2), 29.5 (CH_3), 28.5 (CH_2), 27.9 (CH_2), 21.8 (CH_3). **IR** (ATR): 2926, 1599, 1537, 1461, 1337, 1164, 1080, 875, 813, 735 cm^{-1}. **MS** (ESI) *m/z* (relative intensity): 433 $[M+H]^+$ (100), 455 $[M+Na]^+$ (50). **HR-MS** (ESI) *m/z* calc. for $C_{26}H_{29}N_2O_2S$ $[M+H]^+$: 433.1944, found: 433.1947.

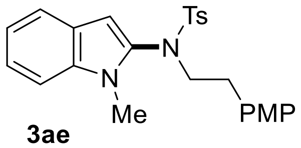

3ae

***N*-(4-methoxyphenethyl)-4-methyl-*N*-(1-methyl-1*H*-indol-2-yl)benzenesulfon amide (3ae)**

The general procedure was followed using **1a** (65.5 mg, 0.50 mmol) and **2e** (305 mg, 1.0 mmol). Purification by column chromatography on silica gel (*n*-hexane/EtOAc = 3:1) yielded **3ae** (139 mg, 64%) as a colorless oil. **^{1}H NMR** (400 MHz, $CDCl_3$): δ = 7.63 (d, J = 8.2 Hz, 2H), 7.56 (d, J = 8.0 Hz, 1H), 7.39–7.29 (m, 4H), 7.16 (dt, J = 7.6, 0.8 Hz, 1H), 7.02 (d, J = 8.4 Hz, 2H), 6.81 (d, J = 8.4 Hz, 2H), 5.91 (s, 1H), 4.13 (s, 1H), 3.79 (s, 3H), 3.68 (s, 3H), 3.33 (s, 1H), 2.80 (s, 1H), 2.66 (s, 1H), 2.48 (s, 3H). **^{13}C NMR** (100 MHz, $CDCl_3$): δ = 158.4 (C_q), 144.1 (C_q), 135.4 (C_q), 134.9 (C_q), 134.0 (C_q), 129.8 (CH), 129.7 (C_q), 129.5 (CH), 128.5 (CH), 126.0 (C_q), 122.5 (CH), 120.9 (CH), 119.9 (CH), 114.0 (CH), 110.1 (CH), 97.6 (CH), 55.3 (CH_3), 54.3 (CH_2), 33.7 (CH_2), 29.4 (CH_3), 21.7 (CH_3). **IR** (ATR): 2928, 1611, 1598, 1583, 1538, 1511, 1462, 1346, 1245, 1160, 1089, 912, 813 cm^{-1}. **MS** (ESI) *m/z* (relative intensity): 435 $[M+H]^+$ (100), 457 $[M+Na]^+$ (98). **HR-MS** (ESI) *m/z* calc. for $C_{25}H_{27}N_2O_3S$ $[M+H]^+$: 435.1737, found: 435.1741.

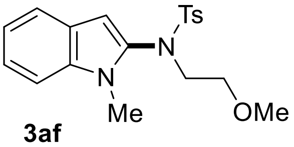

3af

N-(2-methoxyethyl)-4-methyl-N-(1-methyl-1H-indol-2-yl)benzenesulfonamide (3af)

The general procedure was followed using **1a** (65.5 mg, 0.50 mmol) and **2f** (229 mg, 1.0 mmol). Purification by column chromatography on silica gel (*n*-hexane/EtOAc = 3:1) yielded **3af** (107 mg, 60%) as a yellow solid. **M.p.**: 114–116 °C. **^{1}H NMR** (400 MHz, $CDCl_3$): δ = 7.63 (d, J = 8.4 Hz, 2H), 7.50 (d, J = 8.4 Hz, 1H), 7.36–7.24 (m, 4H), 7.12-7.08 (m, 1H), 5.82 (d, J = 0.8 Hz, 1H), 4.06 (m, 1H), 3.79 (s, 3H), 3.39–3.29 (m, 3H), 3.24 (s, 3H), 2.46 (s, 3H). **^{13}C NMR** (100 MHz, $CDCl_3$): δ = 144.1 (C_q), 135.4 (C_q), 135.0 (C_q), 134.4 (C_q), 129.5 (CH), 128.5 (CH), 125.9 (C_q), 122.5 (CH), 120.9 (CH), 119.9 (CH), 110.1 (CH), 97.9 (CH), 69.7 (CH_2), 58.5 (CH_3), 52.4 (CH_2), 29.3 (CH_3), 21.7 (CH_3). **IR** (ATR): 1596, 1537, 1432, 1350, 1286, 1164, 1098, 914, 735 cm^{-1}. **MS** (ESI) *m/z* (relative intensity): 359 $[M+H]^+$ (60), 381 $[M+Na]^+$ (100). **HR-MS** (ESI) *m/z* calc. for $C_{19}H_{23}N_2O_3S$ $[M+H]^+$: 359.1424, found: 359.1427.

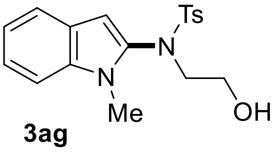

3ag

N-(2-hydroxyethyl)-4-methyl-N-(1-methyl-1H-indol-2-yl)benzenesulfonamide (3ag)

The general procedure was followed using **1a** (65.5 mg, 0.50 mmol) and **2g** (215 mg, 1.0 mmol). Purification by column chromatography on silica gel (*n*-hexane/EtOAc = 3:1) yielded **3ag** (64 mg, 37%) as a yellow solid. **M.p.**: 129–130 °C. **^{1}H NMR** (400 MHz, $CDCl_3$): δ = 7.64 (d, J = 8.4 Hz, 2H), 7.51 (d, J = 8.0 Hz, 1H), 7.36–7.28 (m, 4H), 7.11 (dt, J = 8.8, 0.8 Hz, 1H), 5.88 (s, 1H), 3.99 (s, 1H), 3.81 (s, 3H), 3.66 (m, 2H), 3.35 (m, 1H), 2.47 (s, 3H). **^{13}C NMR** (100 MHz, $CDCl_3$): δ = 144.4 (C_q), 135.4 (C_q), 135.1 (C_q), 134.3 (C_q), 129.7 (CH), 128.5 (CH), 125.9 (C_q), 122.8 (CH), 121.0 (CH), 120.1 (CH), 110.2 (CH), 98.0 (CH), 60.5 (CH_2), 55.8 (CH_2), 29.5 (CH_3), 21.8 (CH_3). **IR** (ATR): 2921, 1697, 1598, 1539, 1469, 1338, 1158, 1040, 922, 802 cm^{-1}. **MS** (ESI) *m/z* (relative intensity): 345 $[M+H]^+$ (80), 367 $[M+Na]^+$ (100). **HR-MS** (ESI) *m/z* calc. for $C_{18}H_{21}N_2O_3S$ $[M+H]^+$: 345.1267, found: 345.1269.

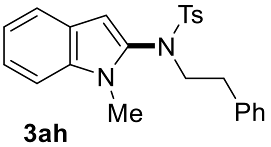

3ah

4-methyl-*N*-(1-methyl-1*H*-indol-2-yl)-*N*-phenethylbenzenesulfonamide (3ah)

The general procedure was followed using **1a** (65.5 mg, 0.50 mmol) and **2h** (275 mg, 1.0 mmol). Purification by column chromatography on silica gel (*n*-hexane/EtOAc = 3:1) yielded **3ah** (113 mg, 56%) as a white solid. **M.p.**: 149–151 °C. **^{1}H NMR** (400 MHz, $CDCl_3$): δ = 7.63 (d, J = 8.4 Hz, 2H), 7.56 (d, J = 8.0 Hz, 1H), 7.37 (d, J = 8.0 Hz, 1H), 7.33–7.20 (m, 6H), 7.18–7.14 (m, 1H), 7.12–7.10 (m, 2H), 5.91 (s, 1H), 4.80 (m, 1H), 3.67 (s, 3H), 3.37 (m, 1H), 2.86–2.74 (m, 2H), 2.47 (s, 3H). **^{13}C NMR** (100 MHz, $CDCl_3$): δ = 144.2 (C_q), 137.7 (C_q), 135.4 (C_q), 134.9 (C_q), 133.9 (C_q), 129.5 (CH), 128.8 (CH), 128.6 (CH), 128.5 (CH), 126.7 (CH), 126.0 (C_q), 122.5 (CH), 120.9 (CH), 120.0 (CH), 110.1 (CH), 97.7 (CH), 54.0 (CH_2), 34.6 (CH_2), 29.4 (CH_3), 21.7 (CH_3). **IR** (ATR): 2918, 1596, 1543, 1453, 1429, 1350, 1165, 1094, 754, 683 cm^{-1}. **MS** (ESI) *m/z* (relative intensity): 405 $[M+H]^+$ (60), 427 $[M+Na]^+$ (100). **HR-MS** (ESI) *m/z* calc. for $C_{24}H_{25}N_2O_2S$ $[M+H]^+$: 405.1631, found: 405.1635.

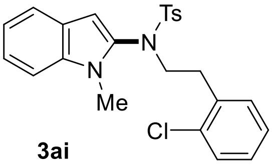

3ai

***N*-(2-chlorophenethyl)-4-methyl-*N*-(1-methyl-1*H*-indol-2-yl)benzenesulfonamide (3ai)**

The general procedure was followed using **1a** (65.5 mg, 0.50 mmol) and **2i** (309 mg, 1.0 mmol). Purification by column chromatography on silica gel (*n*-hexane/EtOAc = 3:1) yielded **3ai** (136 mg, 62%) as a white solid. **M.p.**: 142–144 °C. **^{1}H NMR** (400 MHz, $CDCl_3$): δ = 7.66 (d, J = 8.4 Hz, 2H), 7.59 (d, J = 7.6 Hz, 1H), 7.41 (d, J = 8.0 Hz, 1H), 7.36–7.32 (m, 4H), 7.20–7.17 (m, 4H), 5.98 (s, 1H), 4.15 (m, 1H), 3.78 (s, 3H), 3.45 (m, 1H), 3.06–2.87 (m, 2H), 2.50 (s, 3H). **^{13}C NMR** (100 MHz, $CDCl_3$): δ = 144.2 (C_q), 135.46 (C_q), 135.45 (C_q), 134.8 (C_q), 134.3 (C_q), 134.0 (C_q), 131.0 (CH), 129.7 (CH), 129.5 (CH), 128.6 (CH), 128.3 (CH), 127.1 (CH), 126.0 (C_q), 122.5 (CH), 121.0 (CH), 119.9 (CH), 110.1 (CH), 97.9 (CH), 52.2 (CH_2), 32.8 (CH_2), 29.5 (CH_3), 21.7 (CH_3). **IR** (ATR): 1598, 1534, 1471, 1350, 1164, 1072, 918, 885, 734 cm^{-1}. **MS** (ESI) *m/z*

(relative intensity): 439 $[M+H]^+$ (100), 461 $[M+Na]^+$ (70). **HR-MS** (ESI) *m/z* calc. for $C_{24}H_{24}{}^{35}ClN_2O_2S$ $[M+H]^+$: 439.1242, found: 439.1243.

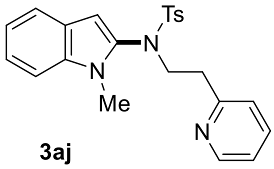

3aj

4-methyl-*N*-(1-methyl-1*H*-indol-2-yl)-*N*-(2-(pyridin-2-yl)ethyl)benzenesulfonamide (3aj)

The general procedure was followed using **1a** (65.5 mg, 0.50 mmol) and **2j** (276 mg, 1.0 mmol). Purification by column chromatography on silica gel (*n*-hexane/EtOAc = 3:1) yielded **3aj** (101 mg, 50%) as a white solid. **M.p.**: 148–150 °C. **^{1}H NMR** (400 MHz, $CDCl_3$): δ = 8.48 (d, *J* = 4.4 Hz, 1H), 7.65 (d, *J* = 8.4 Hz, 2H), 7.56–7.51 (m, 2H), 7.36–7.27 (m, 4H), 7.15–7.09 (m, 3H), 5.90 (s, 1H), 4.33 (m, 1H), 3.67 (s, 3H), 3.59 (m, 1H), 3.02–2.95 (m, 2H), 2.48 (s, 3H). **^{13}C NMR** (100 MHz, $CDCl_3$): δ = 158.0 (C_q), 149.4 (CH), 144.2 (C_q), 136.6 (CH), 135.4 (C_q), 134.8 (C_q), 134.0 (C_q), 129.5 (CH), 128.6 (CH), 126.0 (C_q), 123.4 (CH), 122.5 (CH), 121.7 (CH), 121.0 (CH), 119.9 (CH), 110.0 (CH), 98.0 (CH), 52.5 (CH_2), 36.8 (CH_2), 29.4 (CH_3), 21.8 (CH_3). **IR** (ATR): 2923, 1588, 1473, 1455, 1429, 1393, 1340, 1160, 907, 871, 764 cm^{-1}. **MS** (ESI) *m/z* (relative intensity): 406 $[M+H]^+$ (100), 428 $[M+Na]^+$ (10). **HR-MS** (ESI) *m/z* calc. for $C_{23}H_{24}N_3O_2S$ $[M+H]^+$: 406.1584, found: 406.1589.

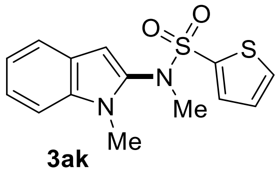

3ak

***N*-methyl-*N*-(1-methyl-1*H*-indol-2-yl)thiophene-2-sulfonamide (3ak)**

The general procedure was followed using **1a** (65.5 mg, 0.50 mmol) and **2k** (176 mg, 1.0 mmol). Purification by column chromatography on silica gel (*n*-hexane/EtOAc = 3:1) yielded **3ak** (107 mg, 70%) as a yellow solid. **M.p.**: 199–200 °C. **^{1}H NMR** (400 MHz, $CDCl_3$): δ = 7.72 (dd, *J* = 5.2, 1.2 Hz, 1H), 7.55–7.53 (m, 2H), 7.37 (d, *J* = 8.0 Hz, 1H), 7.30 (dt, *J* = 8.0, 0.8 Hz, 1H), 7.21–7.12 (m, 2H), 5.97 (s, 1H), 3.82 (s, 3H), 3.29 (s, 3H). **^{13}C NMR** (100 MHz, $CDCl_3$): δ = 136.3 (C_q), 135.8 (C_q), 135.2 (C_q), 134.2 (CH), 133.0 (CH), 127.6 (CH), 125.9 (C_q), 122.7 (CH), 121.0 (CH), 120.1 (CH), 110.1 (CH), 96.6 (CH), 40.6 (CH_3), 29.5 (CH_3). **IR** (ATR): 2920, 1537, 1452, 1350,

1160, 1012, 798, 730, 665 cm^{-1}. **MS** (ESI) *m/z* (relative intensity): 307 [M+H]$^+$ (35), 329 [M+Na]$^+$ (100). **HR-MS** (ESI) *m/z* calc. for $C_{14}H_{15}N_2O_2S_2$ [M+H]$^+$: 307.0569, found: 307.0572.

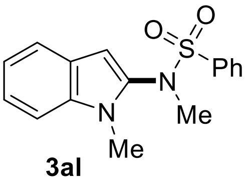

3al

***N*-methyl-*N*-(1-methyl-1*H*-indol-2-yl)benzenesulfonamide (3al)**

The general procedure was followed using **1a** (65.5 mg, 0.50 mmol) and **2l** (171 mg, 1.0 mmol). Purification by column chromatography on silica gel (*n*-hexane/EtOAc = 3:1) yielded **3al** (107 mg, 71%) as a white solid. **M.p.**: 109–111 °C. **^{1}H NMR** (400 MHz, $CDCl_3$): δ = 7.78 (d, J = 8.4 Hz, 2H), 7.70 (t, J = 7.2 Hz, 1H), 7.59–7.51 (m, 3H), 7.38 (d, J = 8.0 Hz, 1H), 7.32–7.30 (m, 1H), 7.16–7.12 (m, 1H), 5.82 (s, 1H), 3.84 (s, 3H), 3.23 (s, 3H). **^{13}C NMR** (100 MHz, $CDCl_3$): δ = 136.8 (C_q), 135.8 (C_q), 135.2 (C_q), 133.4 (CH), 128.9 (CH), 128.7 (CH), 125.9 (C_q), 122.6 (CH), 120.9 (CH), 120.0 (CH), 110.0 (CH), 96.7 (CH), 40.6 (CH_3), 29.5 (CH_3). **IR** (ATR): 2922, 1580, 1539, 1471, 1392, 1352, 1162, 1090, 872, 777 cm^{-1}. **MS** (ESI) *m/z* (relative intensity): 301 [M+H]$^+$ (50), 323 [M+Na]$^+$ (100). **HR-MS** (ESI) *m/z* calc. for $C_{16}H_{17}N_2O_2S$ [M+H]$^+$: 301.1005, found: 301.1009.

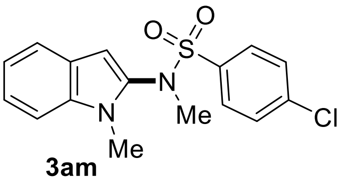

3am

4-chloro-*N*-methyl-*N*-(1-methyl-1*H*-indol-2-yl)benzenesulfonamide (3am)

The general procedure was followed using **1a** (65.5 mg, 0.50 mmol) and **2m** (204 mg, 1.0 mmol). Purification by column chromatography on silica gel (*n*-hexane/EtOAc = 3:1) yielded **3am** (109 mg, 65%) as a white solid. **M.p.**: 110–112 °C. **^{1}H NMR** (400 MHz, $CDCl_3$): δ = 7.67 (d, J = 8.8 Hz, 2H), 7.52–7.50 (m, 3H), 7.35 (d, J = 8.4 Hz, 1H), 7.31–7.27 (m, 1H), 7.15–7.11 (m, 1H), 5.82 (s, 1H), 3.81 (s, 3H), 3.20 (s, 3H). **^{13}C NMR** (100 MHz, $CDCl_3$): δ = 140.0 (C_q), 136.4 (C_q), 135.1 (C_q), 134.2 (C_q), 130.1 (CH), 129.2 (CH), 125.8 (C_q), 122.7 (CH), 120.9 (CH), 120.1 (CH), 110.0 (CH), 96.6 (CH), 40.5 (CH_3), 29.5 (CH_3). **IR** (ATR): 1542, 1462, 1447, 1337, 1165, 871, 777, 734

cm^{-1}. **MS** (ESI) *m/z* (relative intensity): 335 [M+H]$^+$ (40), 357 [M+Na]$^+$ (100). **HR-MS** (ESI) *m/z* calc. for $C_{16}H_{16}{}^{35}ClN_2O_2S$ [M+H]$^+$: 335.0616, found: 335.0614.

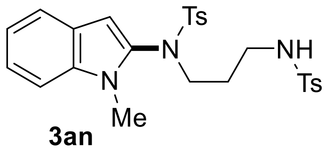

3an

4-methyl-*N*-(1-methyl-1*H*-indol-2-yl)-*N*-{3-[(4-methylphenyl)sulfonamido]propyl} benzenesulfonamide (3an)

The general procedure was followed using **1a** (65.5 mg, 0.50 mmol) and **2n** (382 mg, 1.0 mmol). Purification by column chromatography on silica gel (*n*-hexane/EtOAc = 3:1) yielded **3an** (82 mg, 32%) as a colorless oil. **^{1}H NMR** (400 MHz, $CDCl_3$): δ = 7.71 (d, J = 8.4 Hz, 2H), 7.60 (d, J = 8.4 Hz, 2H), 7.51 (dd, J = 8.0, 0.8 Hz, 1H), 7.34–7.26 (m, 6H), 7.14 (dt, J = 8.0, 1.2 Hz, 1H), 5.85 (s, 1H), 5.07 (t, J = 6.4 Hz, 1H), 3.91 (br s, 3H), 3.67 (s, 3H), 3.17 (br s, 1H), 3.03 (q, J = 6.4 Hz, 2H), 2.49 (s, 3H), 2.44 (s, 3H), 1.63 (t, J = 6.4 Hz, 2H). **^{13}C NMR** (100 MHz, $CDCl_3$): δ = 144.5 (C_q), 143.5 (C_q), 137.0 (C_q), 135.4 (C_q), 134.9 (C_q), 133.6 (C_q), 129.8 (CH), 129.6 (CH), 128.5 (CH), 127.1 (CH), 125.9 (C_q), 122.7 (CH), 120.9 (CH), 120.1 (CH), 110.0 (CH), 97.7 (CH), 50.5 (CH_2), 40.1 (CH_2), 29.5 (CH_3), 28.4 (CH_2), 21.7 (CH_3), 21.6 (CH_3). **IR** (ATR): 3292, 2924, 1597, 1538, 1470, 1429, 1336, 1155, 1088, 812, 726 cm^{-1}. **MS** (ESI) *m/z* (relative intensity): 512 [M+H]$^+$ (90), 534 [M+Na]$^+$ (100). **HR-MS** (ESI) *m/z* calc. for $C_{26}H_{30}N_3O_4S_2$ [M+H]$^+$: 512.1672, found: 512.1673.

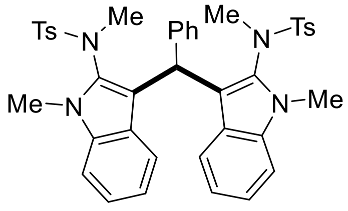

4

***N*,*N*'-[(phenylmethylene)bis(1-methyl-1*H*-indole-3,2-diyl)]bis(*N*,4-dimethyl benzenesulfonamide) (4)**

In a 15 mL flask with a stir-bar was charged with benzaldehyde (16 mg, 0.15 mmol) and **3aa** (94.2 mg, 0.3 mmol). EtOAc (5.0 mL) and H_2SO_4 (10.0 mg) were added and the solution was stirred at room temperature for 10 hours. Purification by filtration and crystallisation (*n*-hexane/EtOAc) yielded **4** (91.0 mg, 85%) as a white solid; **M.p.**: 272–274 °C; **^{1}H NMR** (400 MHz, $CDCl_3$): δ = 7.43 (d, J = 8.0 Hz, 4H), 7.28–7.26 (m, 4H),

7.18–7.10 (m, 9H), 6.76 (t, J = 7.6 Hz, 2H), 6.59 (t, J = 8.4 Hz, 2H), 6.10 (s, 1H), 3.41 (s, 6H), 3.08 (s, 6H), 2.40 (s, 6H). **^{13}C NMR** (100 MHz, $CDCl_3$): δ = 143.8 (C_q), 143.7 (C_q), 136.6 (C_q), 135.2 (C_q), 132.6 (C_q), 129.8 (CH), 129.6 (CH), 128.0 (CH), 127.6 (CH), 126.2 (C_q), 122.3 (CH), 122.0 (CH), 119.6 (CH), 114.8 (C_q), 109.4 (CH), 38.3 (CH_3), 38.0 (CH), 29.7 (CH_3), 21.7 (CH_3). **IR** (ATR): 1599, 1557, 1492, 1463, 1355, 1335, 1159, 943, 811, 744, 672 cm^{-1}. **MS** (ESI) *m/z* (relative intensity): 717 $[M+H]^+$ (2), 739 $[M+Na]^+$ (100). **HR-MS** (ESI) *m/z* calc. for $C_{41}H_{41}N_4O_4S_2$ $[M+H]^+$: 717.2564, found: 717.2535.

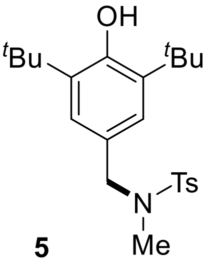

5

***N*-(3,5-di-tert-butyl-4-hydroxybenzyl)-*N*,4-dimethylbenzenesulfonamide (5)**

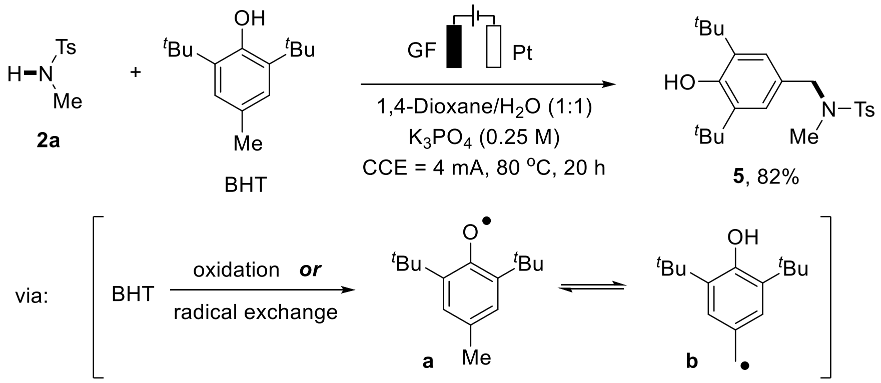

Under the standard conditions as shown in the text (Scheme 5a), **5** can be obtained as a white solid. The oxygen-centered radical **a** is most likely generated by electrooxidation and will form **b** through an intramolecular radical transfer.[5] **M.p.**: 157–159 °C. **^{1}H NMR** (400 MHz, $CDCl_3$): δ = 7.75 (d, J = 8.4 Hz, 2H), 7.38 (d, J = 8.0 Hz, 2H), 7.08 (s, 2H), 5.26 (s, 1H), 4.08 (s, 2H), 2.63 (s, 3H), 2.46 (s, 3H), 1.46 (s, 18H). **^{13}C NMR** (100 MHz, $CDCl_3$): δ = 153.5 (C_q), 143.3 (C_q), 136.1 (C_q), 134.5 (C_q), 129.7 (CH), 127.5 (CH), 126.0 (C_q), 125.2 (CH), 54.4 (CH_2), 34.3 (CH_3), 30.2 (CH_3), 29.5 (CH_3), 21.5 (CH_3). **IR** (ATR): 3626, 2947, 1596, 1433, 1355, 1334, 1161, 924, 747

cm^{-1}. **MS** (ESI) *m/z* (relative intensity): 404 $[M+H]^+$ (10), 426 $[M+Na]^+$ (100). **HR-MS** (ESI) *m/z* calc. for $C_{23}H_{34}NO_3S$ $[M+H]^+$: 404.2254, found: 404.2246.

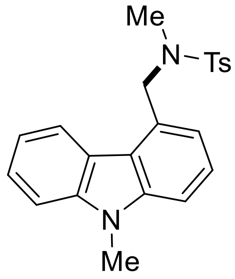

6

N,4-dimethyl-N-[(9-methyl-9H-carbazol-4-yl)methyl]benzenesulfonamide (6)

Under the standard conditions as shown in the text (Scheme 5b), **6** can be obtained as a colorless oil. **^{1}H NMR** (400 MHz, $CDCl_3$): δ = 8.36 (d, *J* = 8.0 Hz, 1H), 7.84 (d, *J* = 8.4 Hz, 2H), 7.51 (t, *J* = 8.0 Hz, 1H), 7.44–7.29 (m, 6H), 7.10 (d, *J* = 6.4 Hz, 1H), 4.76 (s, 2H), 3.87 (s, 3H), 2.56 (s, 3H), 2.50 (s, 3H). **^{13}C NMR** (100 MHz, $CDCl_3$): δ = 143.8 (C_q), 141.5 (C_q), 141.4 (C_q), 133.6 (C_q), 130.2 (C_q), 129.9 (CH), 128.1 (CH), 125.9 (CH), 125.5 (CH), 123.8 (CH), 122.1 (C_q), 121.2 (C_q), 120.4 (CH), 119.5 (CH), 108.5 (CH), 108.4 (CH), 52.9 (CH_2), 34.7 (CH_3), 29.3 (CH_3), 21.8 (CH_3). **IR** (ATR): 2921, 2851, 1595, 1470, 1443, 1425, 1336, 1159, 739, 722 cm^{-1}. **MS** (ESI) *m/z* (relative intensity): 379 $[M+H]^+$ (75), 401 $[M+Na]^+$ (100). **HR-MS** (ESI) *m/z* calc. for $C_{22}H_{23}N_2O_2S$ $[M+H]^+$: 379.1475, found: 379.1481.

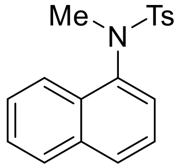

3a'a

N,4-dimethyl-N-(naphthalen-1-yl)benzenesulfonamide (3a'a)

The general procedure was followed using **1a'** (64 mg, 0.50 mmol) and **2a** (185 mg, 1.0 mmol). Purification by column chromatography on silica gel (*n*-hexane/EtOAc = 3:1) yielded **3a'a** (23 mg, 15%) as a colorless oil. **^{1}H NMR** (400 MHz, $CDCl_3$): δ = 8.27 (d, *J* = 8.0 Hz, 1H), 7.86–7.81 (m, 2H), 7.64 (d, *J* = 8.4 Hz, 2H), 7.58–7.47 (m, 2H), 7.34–7.30 (m, 3H), 6.87 (dd, *J* = 7.6, 1.2 Hz, 1H), 3.30 (s, 3H), 2.46 (s, 3H). **^{13}C NMR** (100 MHz, $CDCl_3$): δ = 143.7 (C_q), 138.7 (C_q), 134.84 (C_q), 134.82 (C_q), 132.4 (C_q), 129.6 (CH), 128.9 (CH), 128.3 (CH), 128.1 (CH), 127.0 (CH), 126.7 (CH), 125.1 (CH), 124.9

(CH), 124.2 (CH), 39.9 (CH_3), 21.7 (CH_3). **IR** (ATR): 1596, 1507, 1493, 1451, 1341, 1152, 1087, 1001, 887, 804, 771 cm^{-1}. **MS** (ESI) *m/z* (relative intensity): 312 $[M+H]^+$ (40), 334 $[M+Na]^+$ (100). **HR-MS** (ESI) *m/z* calc. for $C_{18}H_{18}NO_2S$ $[M+H]^+$: 312.1053, found: 312.1056.

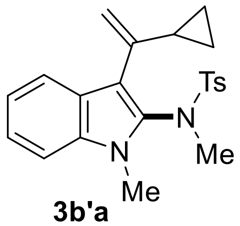

3b'a

_N_-[3-(1-cyclopropylvinyl)-1-methyl-1_H_-indol-2-yl]-_N_,4-dimethylbenzenesulfon amide (3b'a)

The general procedure was followed using **1b'** (98.5 mg, 0.50 mmol) and **2a** (185 mg, 1.0 mmol) at 80 °C for 20 h. Purification by column chromatography on silica gel (*n*-hexane/EtOAc = 3:1) yielded **3b'a** (38 mg, 20%) as a yellow oil. **^{1}H NMR** (400 MHz, $CDCl_3$): δ = 7.73 (d, *J* = 8.4 Hz, 2H), 7.67 (d, *J* = 8.0 Hz, 1H), 7.28–7.24 (m, 4H), 7.12–7.08 (m, 1H), 4.73 (t, *J* = 1.2 Hz, 1H), 4.71 (d, *J* = 1.6 Hz, 1H), 3.64 (s, 3H), 3.32 (s, 3H), 2.43 (s, 3H), 1.40–1.33 (m, 1H), 0.72–0.66 (m, 1H), 0.60–0.43 (m, 3H). **^{13}C NMR** (100 MHz, $CDCl_3$): δ = 143.8 (C_q), 143.1 (C_q), 136.8 (C_q), 134.9 (C_q), 131.4 (C_q), 129.7 (CH), 128.1 (CH), 125.8 (C_q), 123.0 (CH), 121.1 (CH), 119.9 (CH), 115.7 (C_q), 110.4 (C_q), 109.7 (CH), 39.7 (CH_3), 29.7 (CH_3), 21.7 (CH_3), 16.8 (CH), 9.7 (CH_2), 7.5 (CH_2). **IR** (ATR): 2925, 1598, 1553, 1467, 1434, 1342, 1155, 1088, 932, 812 , 780, 742, 665 cm^{-1}. **MS** (ESI) *m/z* (relative intensity): 381 $[M+H]^+$ (100), 403 $[M+Na]^+$ (75). **HR-MS** (ESI) *m/z* calc. for $C_{22}H_{25}N_2O_2S$ $[M+H]^+$: 381.1631, found: 381.1629.

5. Gram-Scale Reaction

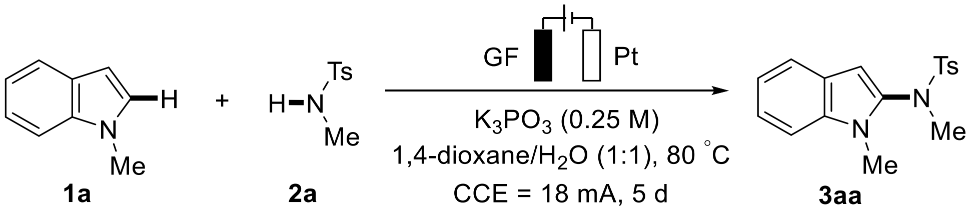

The electrocatalysis was carried out in an undivided cell under air with a graphite felt (GF) anode (25 mm × 50 mm × 6 mm) and a platinum cathode (25 mm × 50 mm × 0.25 mm). Indole **1a** (1.31 g, 10 mmol, 1.0 equiv), *N*-methyl-*para*-toluenesulfonamide **2a** (1.85 g, 20 mmol, 2.0 equiv) and K_3PO_4 (2.12 g, 1.0 equiv) were dissolved in solvent mixture of 1,4-dioxane/H_2O (1:1, 40 mL). Electrocatalysis was performed at 80 °C with a constant current of 18 mA maintained for 5 days. The GF anode was washed with EtOAc (3 × 20 mL) in an ultrasonic bath. The solvent was transfered to a round bottom flask. Silica was added to the flask and all volatiles were evaporated under vacuum. Purification was performed by flash column chromatography on silica gel using *n*-hexane/EtOAc (1:1) as the eluent to yield product **3aa** (1.8 g, 57%).

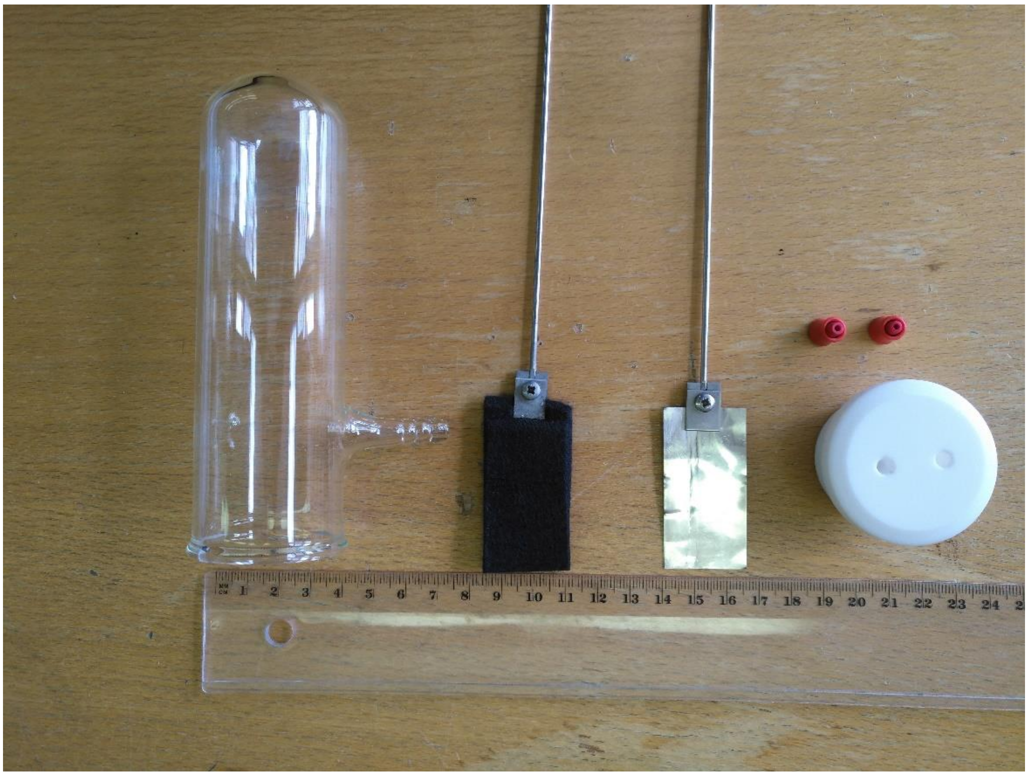

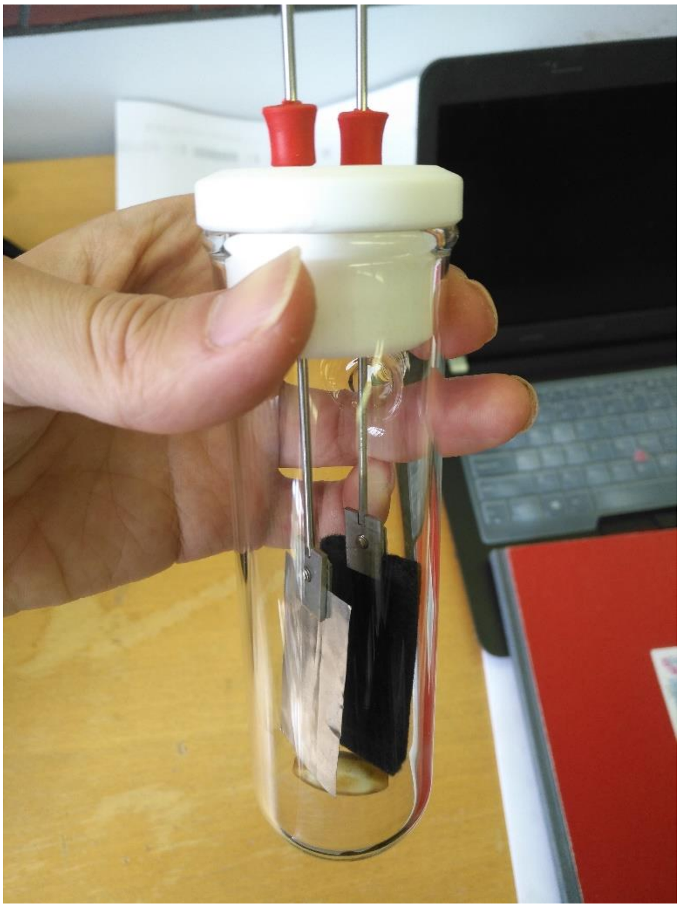

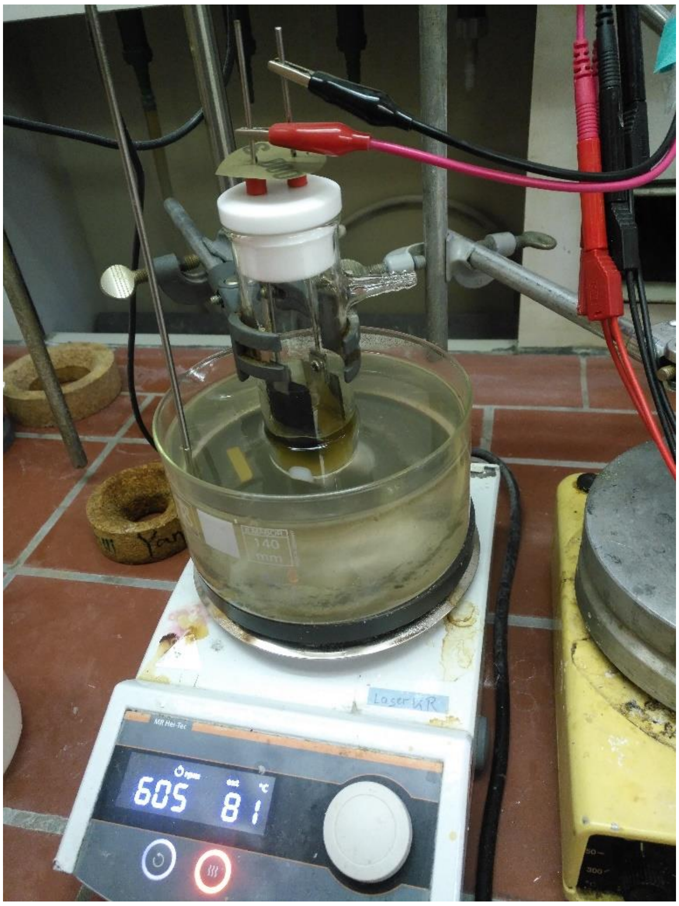
605
81

6. Mechanistic Studies

6.1 Preparation of [D$_1$]-1a

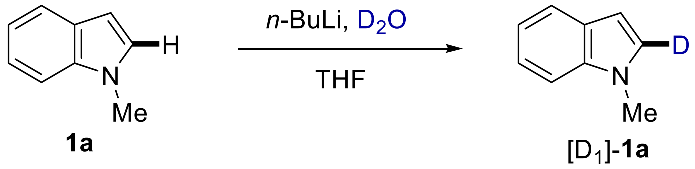

[D$_1$]-**1a** was prepared using a previously published method. [4]

A flame dried three-necked flask was charged with 1-methylindole (0.70 g, 5.1 mmol) and then flushed with N_2. Anhydrous THF (20 mL) was added, and the reaction was allowed to stir until all the 1-methylindole is dissolved. The solution was cooled to 0 °C, and *n*-BuLi (2.5 M, 3.7 mL, 9.2 mmol) was added drop wise. The resulting solution was allowed to warm to ambient temperature, and allowed to stir for an additional 60 minutes. Then 1.0 mL of D_2O was slowly added drop wise. After the solution was fully quenched with D_2O, it was extracted with ethyl acetate (2×20 mL). The resulting organic solution was dried over Na_2SO_4 and the solvent was removed. The final product was purified by chromatography on silica gel to afford the [D$_1$]-**1a** with a 85% yield.

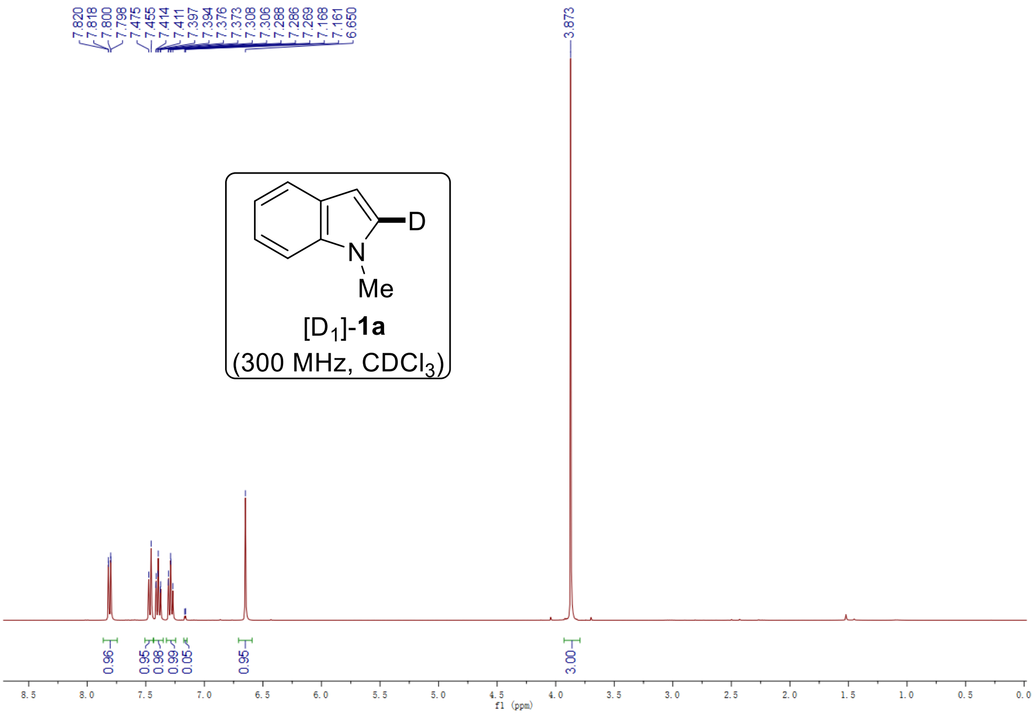

6.2 KIE studies

Parallel experiments

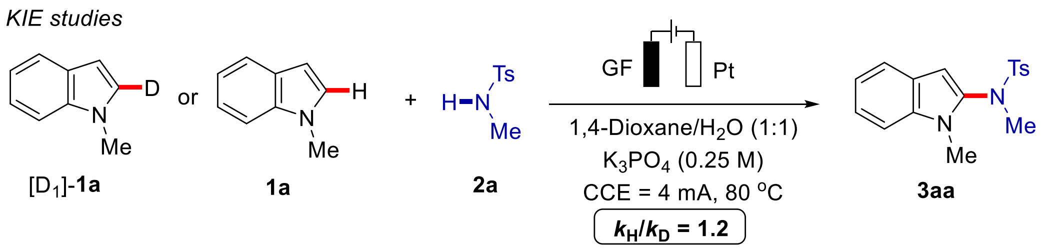

Following the general procedure, an undivided cell was equipped with a GF anode (10 mm × 15 mm × 6 mm) and a platinum cathode (10 mm × 15 mm × 0.25 mm). **1a** (65.5 mg, 0.50 mmol) or [D_5]-**1a** (66.0 mg, 0.50 mmol), sulfonamides **2a** (185 mg, 1.0 mmol) and K_3PO_4 (212 mg, 2.0 equiv) were dissolved in mixture of 1,4-dioxane/H_2O (1:1, 4.0 mL). Electrocatalysis was performed at 80 °C with a constant current of 4.0 mA maintained for 90 min. The GF anode was washed with ethyl acetate (3 × 10 mL) in an ultrasonic bath. The solvent was transfered to a round bottom flask. Silica was added to the flask and all volatiles were evaporated under vacuum. The product **3aa** (17.0 mg and 14.0 mg respectively) was independently isolated by flash chromatography using ethyl acetate/*n*-hexane (1:1) as the eluent. The value of k_H/k_D was determined based on the isolated yields.

6.3 Cyclic voltammetry

CV measurements were conducted with a Metrohm Autolab PGSTAT204 potentiostat and Nova 2.1 software. For all experiments, a glassy carbon working electrode (disk, diameter: 3 mm), a platinum wire counter electrode, and an Ag/AgCl reference electrode were employed. All cyclic voltammograms were obtained at room temperature with a scan rate of 100 mV/s. The CV of sulfonamide (5 mM) was performed in acetonitrile containing 0.1 M nBu$_4$NPF$_6$ (black). K_3PO_4 (0.2 M) (red), the mixture of sulfonamide (0.2 M) and K_3PO_4 (0.2 M) (blue) in acetonitrile/H_2O (10:1), and the mixture of sulfonamide and K_3PO_4 (green) in water were stirred for 10 hours at room temperature before the CV measurement. Deviations from the general experimental setup were indicated in the respective figures and descriptions.

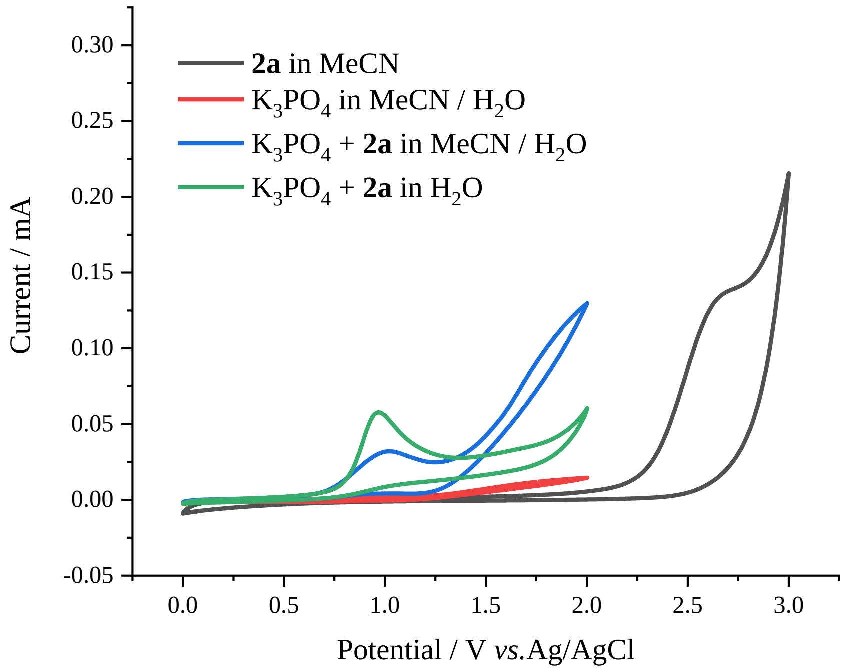

Figure S-1 Cyclic voltammograms.

7. References

[1] X. Dong, R. Sang, Q. Wang, X.-Y . Tang, M. Shi, *Chem. Eur. J.* **2013**, *19*, 16910–16915. .

[2] Y. Yang, X. Meng, B. Zhu, Y. Jia, X. Cao, S. Huang, *Eur. J. Org. Chem*. **2019**, *2019*, 1166–1169.

[3] Y.-S. Bao, C.-Y. Chen, Z.-Z. Huang, *J. Org. Chem*. **2012**, *77*, 8344–8349.

[4] X. Pu, M. Zhang, J. Lan, S. Chen, Z. Liu, W. Liang, Y. Yang, M. Zhang, J. You, *Org. Lett*. **2019**, *21*, 1139–1143.

[5] a) Y. Gao, G. Lu, P. Zhang, L. Zhang, G. Tang, Y. Zhao, *Org. Lett*. **2016**, *18*, 1242–1245; b) H. Egami, T. Ide, Y. Kawato, Y. Hamashima, *Chem. Commun*. **2015**, *51*, 16675–16678.

8. NMR Spectra

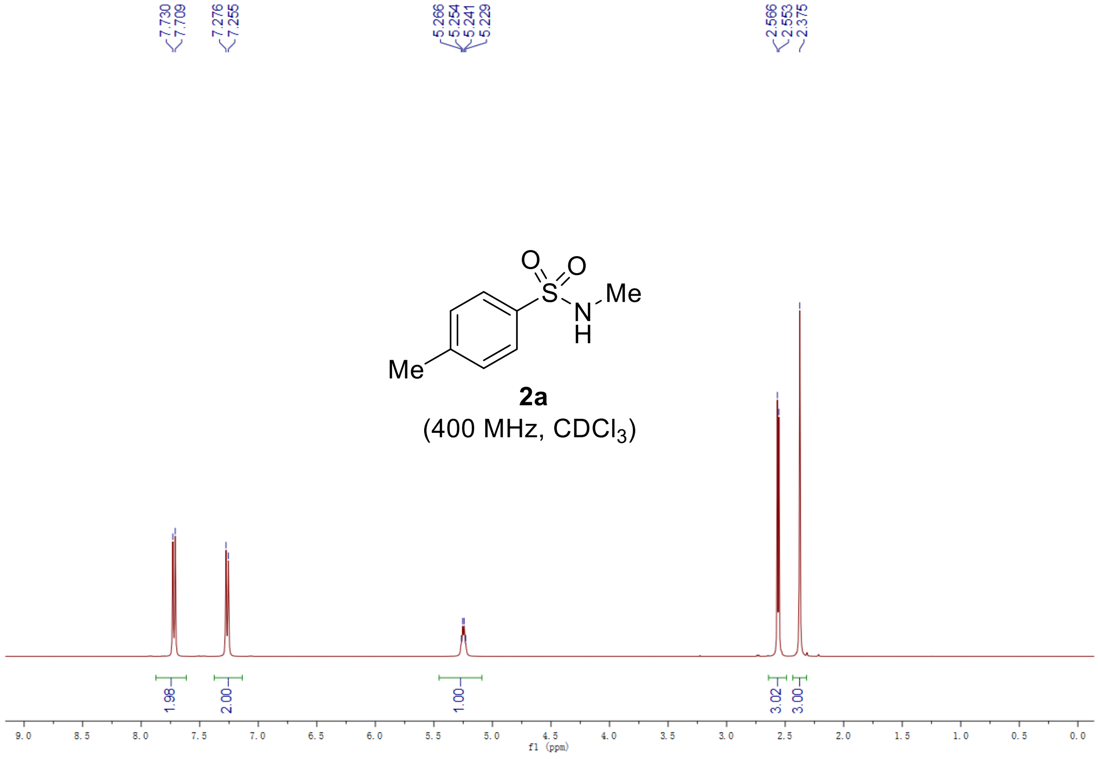

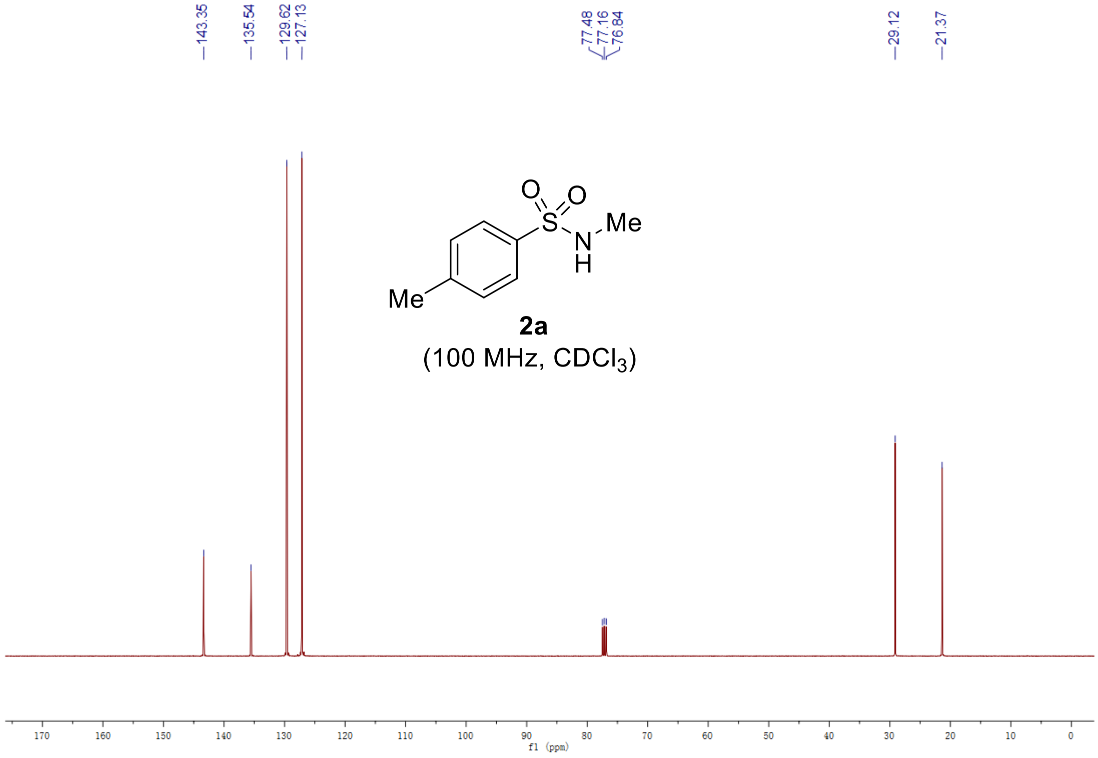

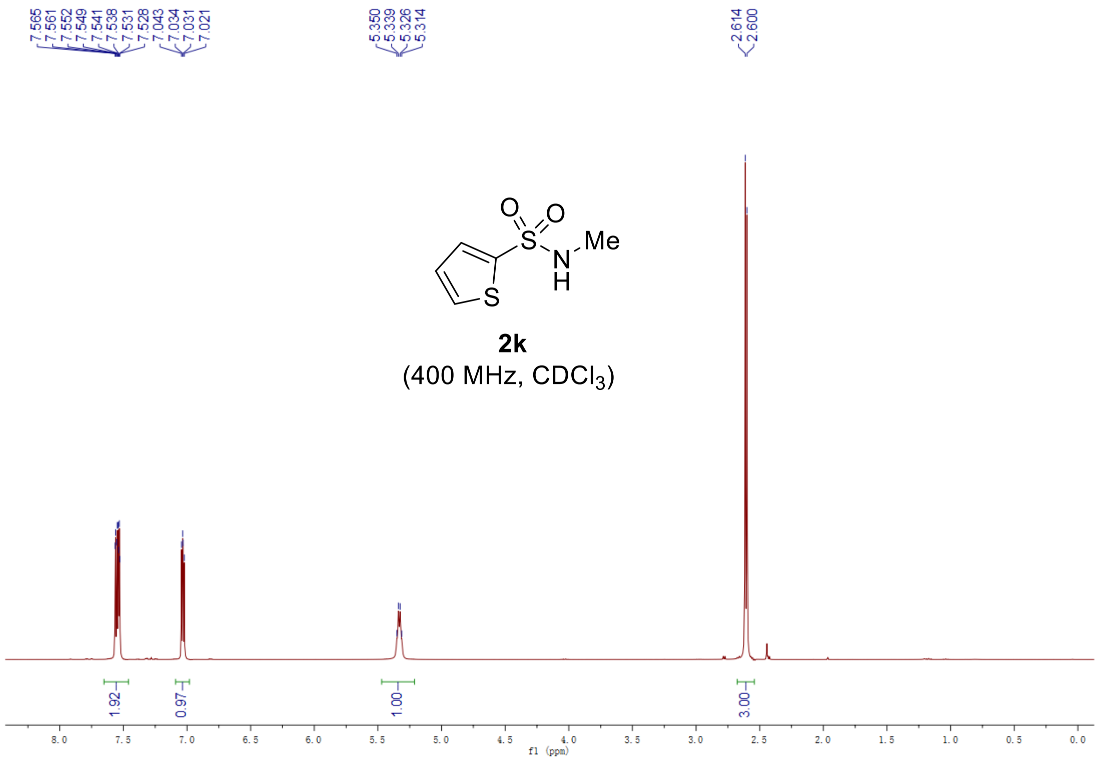
7.565
7.561
7.552
7.549
7.541
7.538
7.531
7.528
7.043
7.034
7.031
7.021
5.350
5.339
5.326
5.314
2.614
2.600
Me
2k
(400 MHz, CDCl3)
1.92
0.97
1.00
3.00
f1 (ppm)

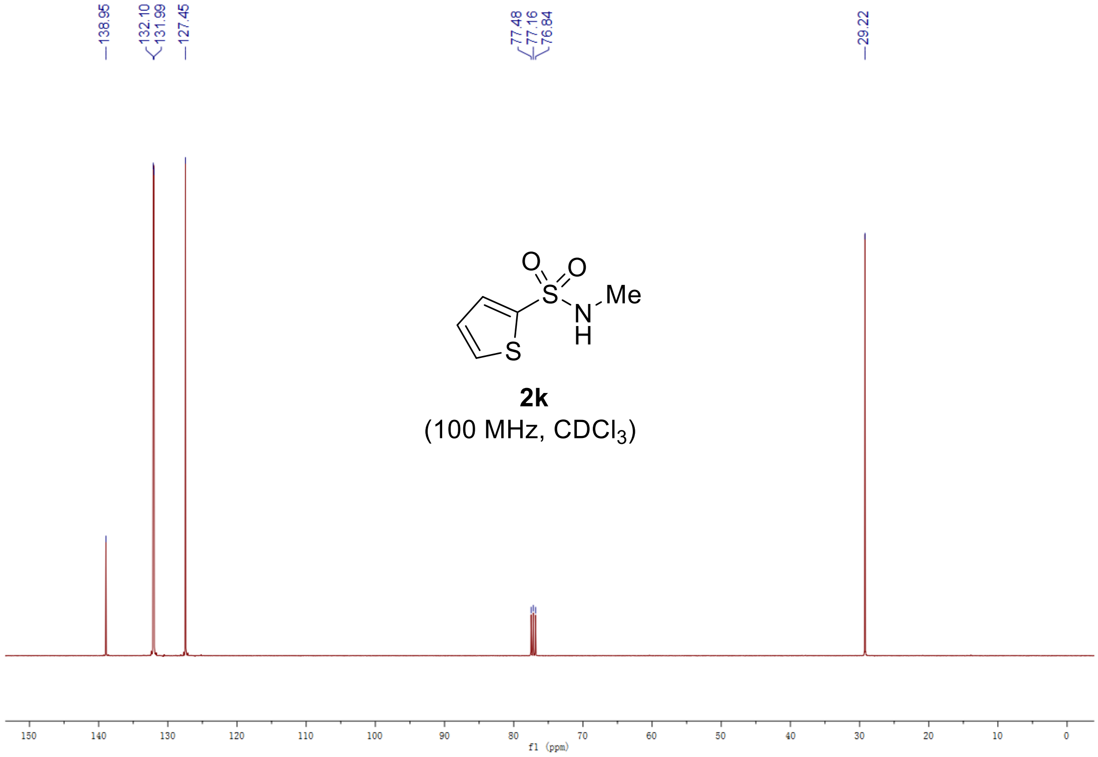
138.95
132.10
131.99
127.45
77.48
77.16
76.84
29.22
Me
2k
(100 MHz, CDCl3)
f1 (ppm)

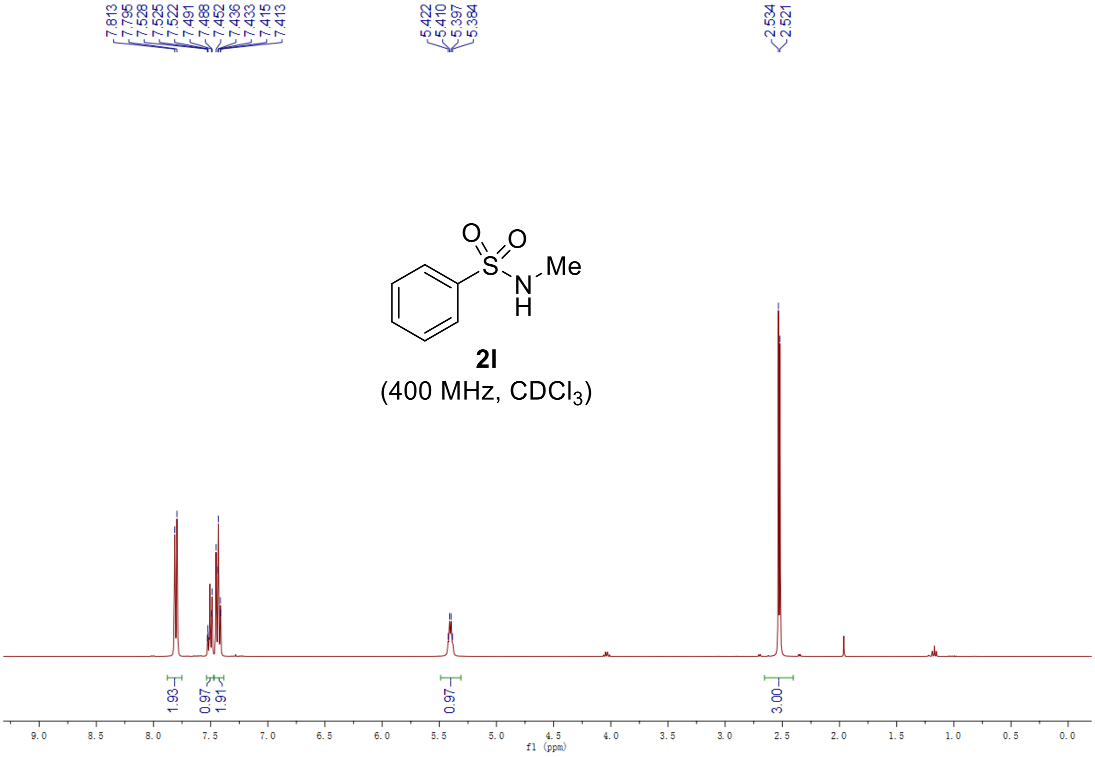
7.813
7.795
7.528
7.525
7.522
7.491
7.488
7.452
7.436
7.433
7.415
7.413
5.422
5.410
5.397
5.384
2.534
2.521
2l
(400 MHz, CDCl3)
1.93
0.97
1.91
0.97
3.00
f1 (ppm)

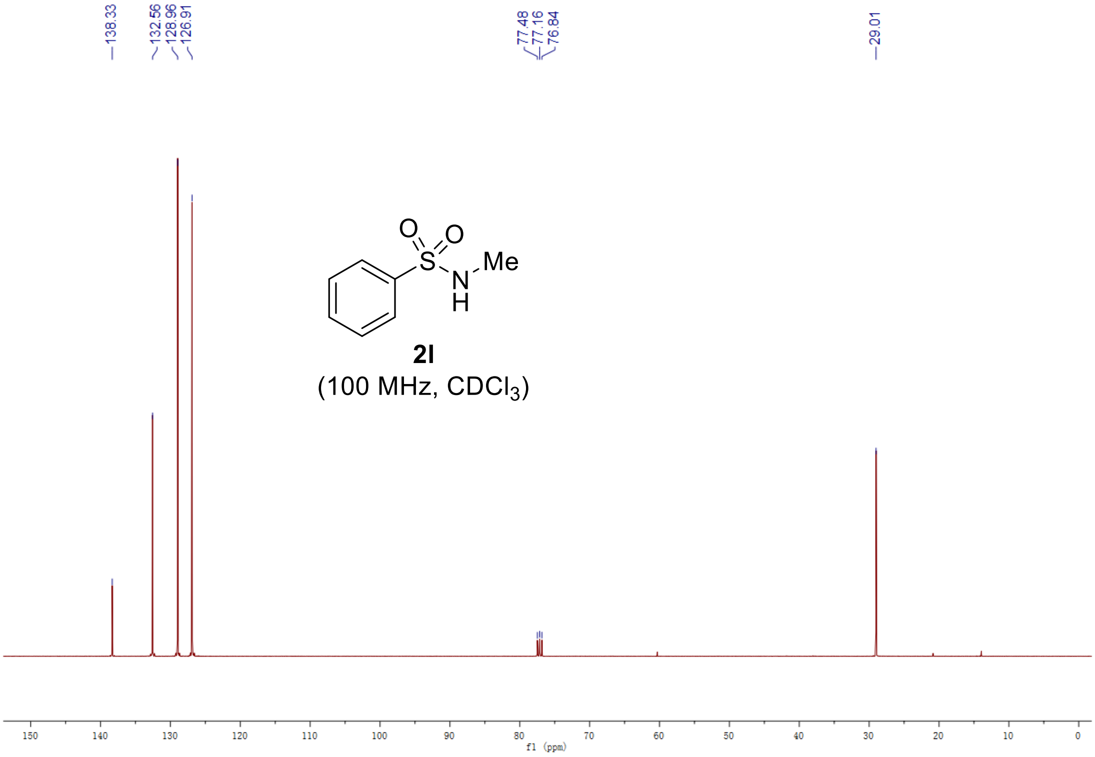
138.33
132.56
128.96
126.91
77.48
77.16
76.84
29.01
2l
(100 MHz, CDCl3)
f1 (ppm)

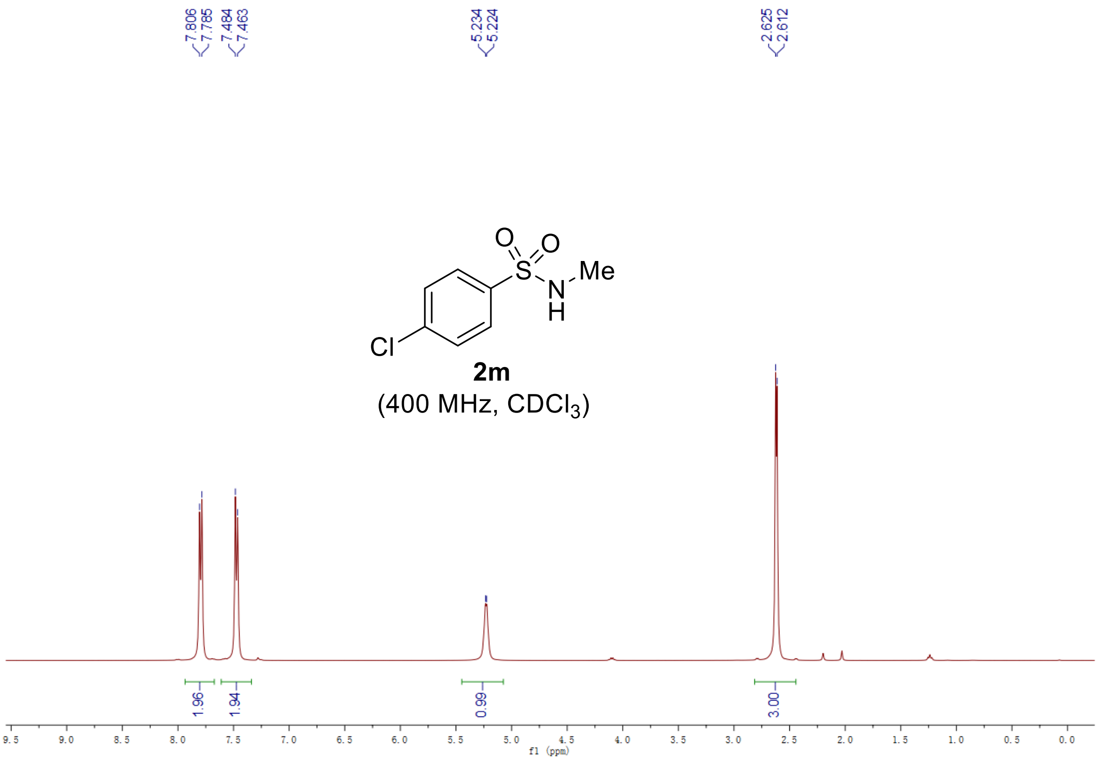
7.806
7.785
7.484
7.463
5.234
5.224
2.625
2.612
2m
(400 MHz, CDCl3)
1.96
1.94
0.99
3.00
f1 (ppm)

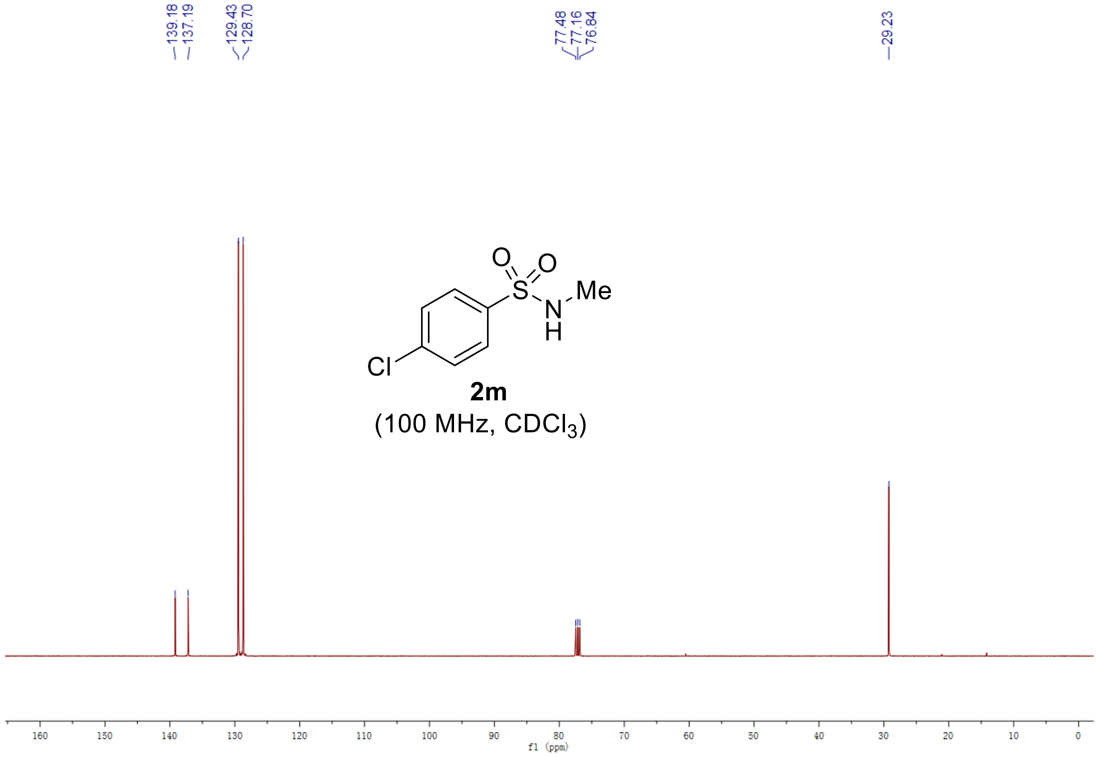
139.18
137.19
129.43
128.70
77.48
77.16
76.84
29.23
2m
(100 MHz, CDCl3)
f1 (ppm)

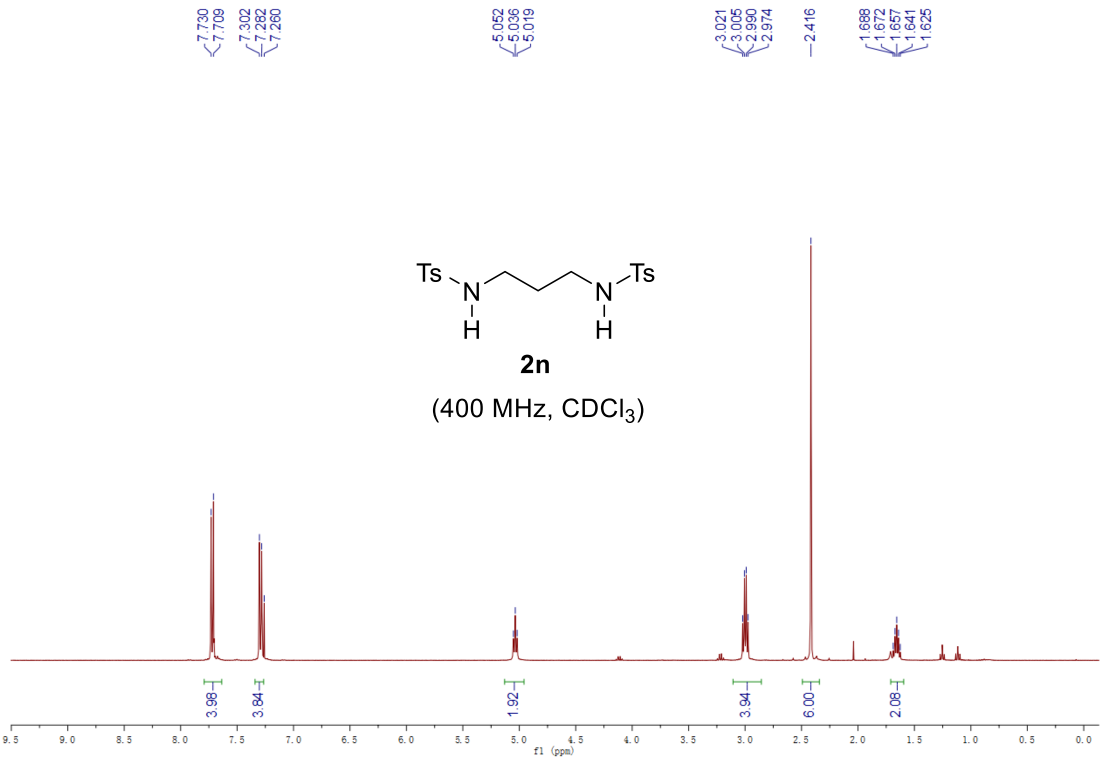
7.730
7.709
7.302
7.282
7.260
5.052
5.036
5.019
3.021
3.005
2.990
2.974
2.416
1.688
1.672
1.657
1.641
1.625
Ts
N
H
2n
(400 MHz, CDCl3)
3.98
3.84
1.92
3.94
6.00
2.08
f1 (ppm)

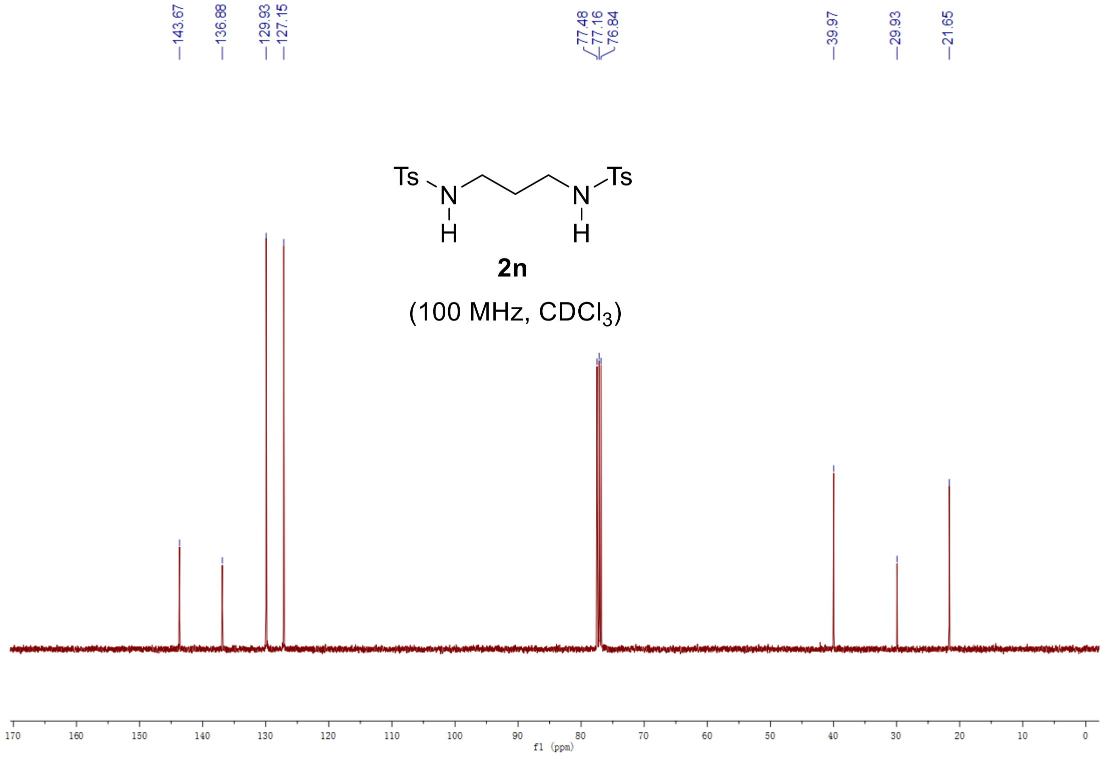
143.67
136.88
129.93
127.15
77.48
77.16
76.84
39.97
29.93
21.65
Ts
N
H
2n
(100 MHz, CDCl3)
f1 (ppm)

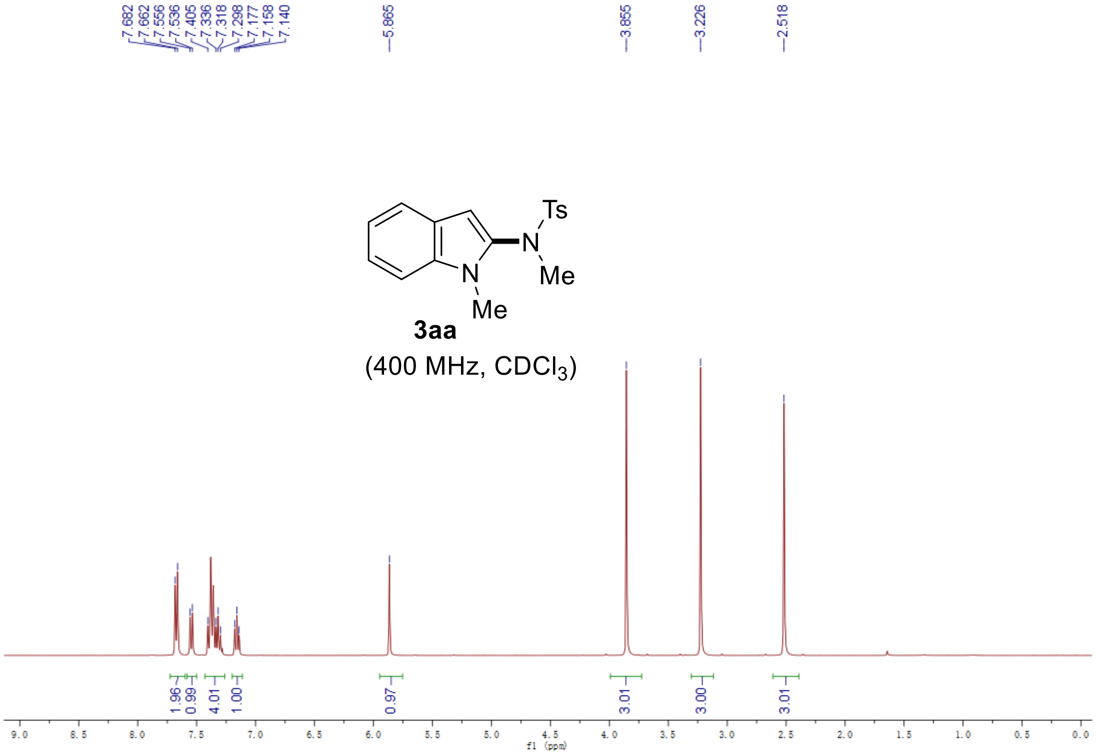
7.682
7.662
7.556
7.536
7.405
7.336
7.318
7.298
7.177
7.158
7.140
5.865
3.855
3.226
2.518
Ts
N
Me
N
Me
3aa
(400 MHz, CDCl3)
1.96
0.99
4.01
1.00
0.97
3.01
3.00
3.01
f1 (ppm)

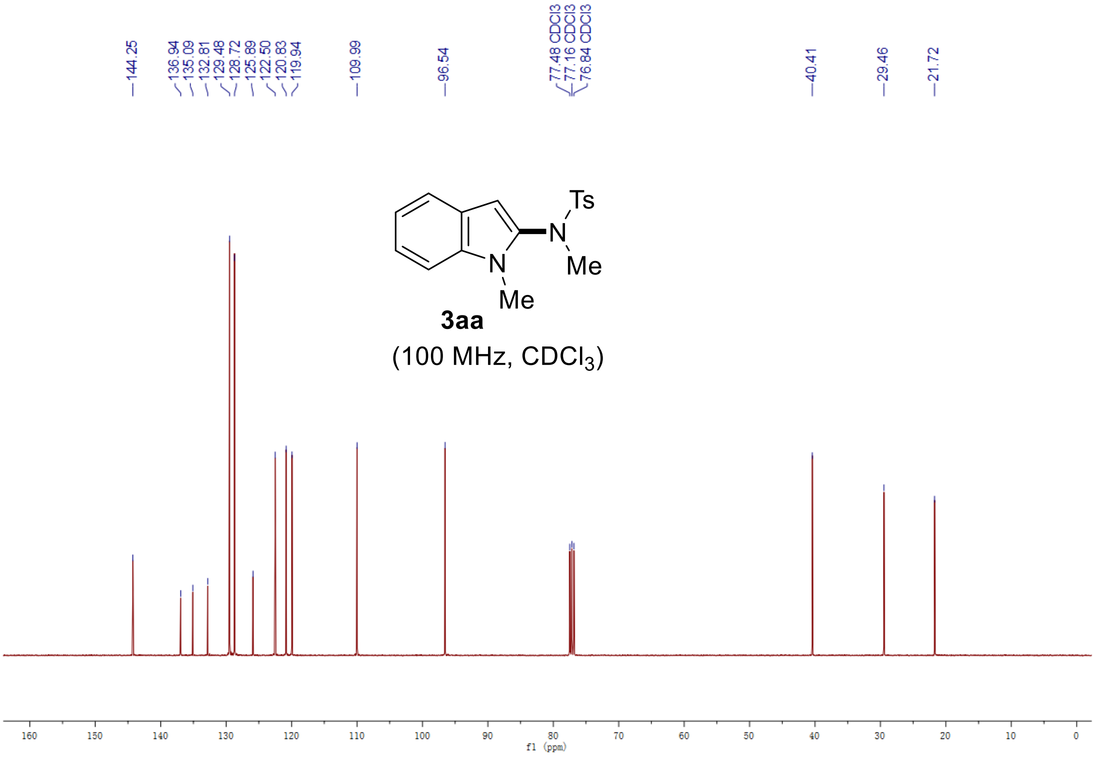
144.25
136.94
135.09
132.81
129.48
128.72
125.89
122.50
120.83
119.94
109.99
96.54
77.48 CDCl3
77.16 CDCl3
76.84 CDCl3
40.41
29.46
21.72
Ts
N
Me
N
Me
3aa
(100 MHz, CDCl3)
f1 (ppm)

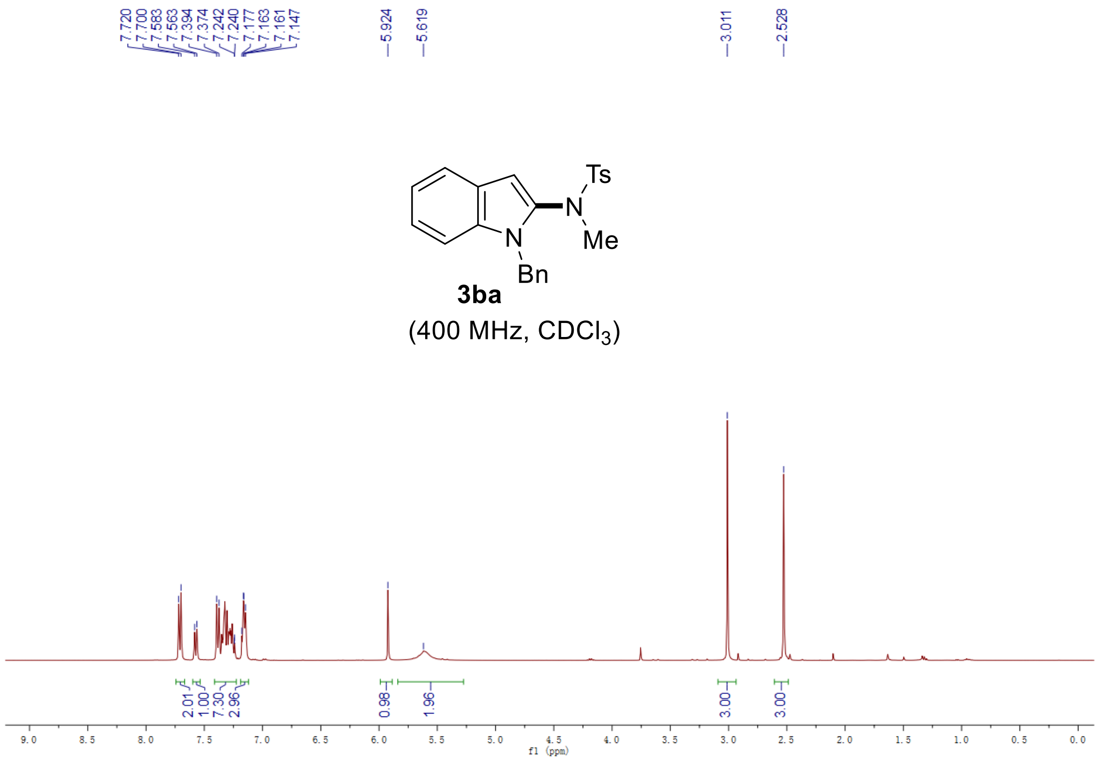
7.720
7.700
7.583
7.563
7.394
7.374
7.242
7.240
7.177
7.163
7.161
7.147
5.924
5.619
3.011
2.528
Ts
N
Me
N
Bn
3ba
(400 MHz, CDCl3)
2.01
1.00
7.30
2.96
0.98
1.96
3.00
3.00
9.0 8.5 8.0 7.5 7.0 6.5 6.0 5.5 5.0 4.5 4.0 3.5 3.0 2.5 2.0 1.5 1.0 0.5 0.0
f1 (ppm)

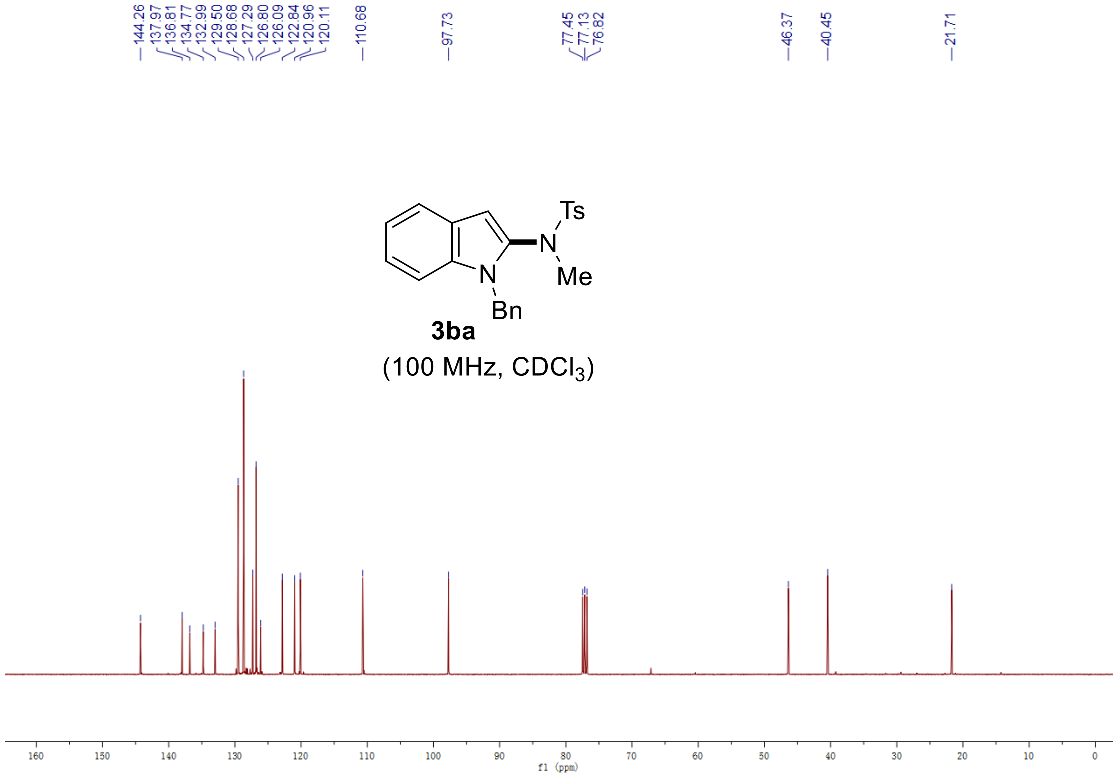
144.26
137.97
136.81
134.77
132.99
129.50
128.68
127.29
126.80
126.09
122.84
120.96
120.11
110.68
97.73
77.45
77.13
76.82
46.37
40.45
21.71
Ts
N
Me
N
Bn
3ba
(100 MHz, CDCl3)
160 150 140 130 120 110 100 90 80 70 60 50 40 30 20 10 0
f1 (ppm)

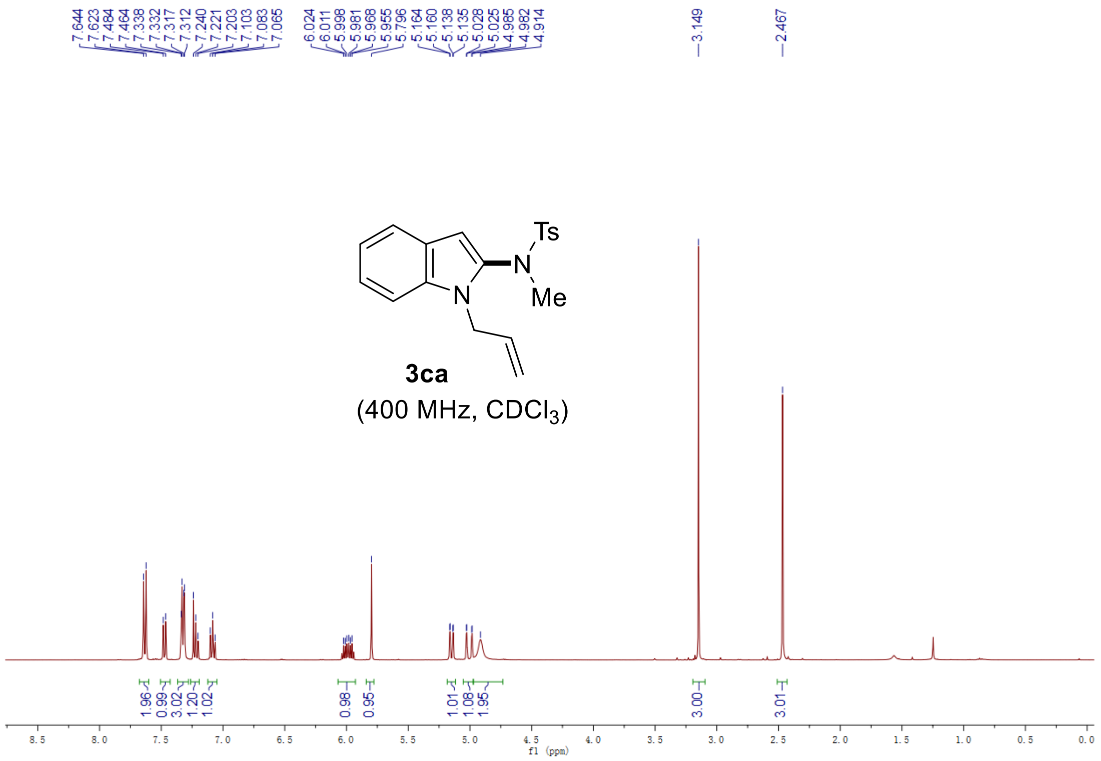
7.644
7.623
7.484
7.464
7.338
7.332
7.317
7.312
7.240
7.221
7.203
7.103
7.083
7.065
6.024
6.011
5.998
5.981
5.968
5.955
5.796
5.164
5.160
5.138
5.135
5.028
5.025
4.985
4.982
4.914
3.149
2.467
Ts
N
Me
N
3ca
(400 MHz, CDCl3)
1.96
0.99
3.02
1.20
1.02
0.98
0.95
1.01
1.08
1.95
3.00
3.01
f1 (ppm)

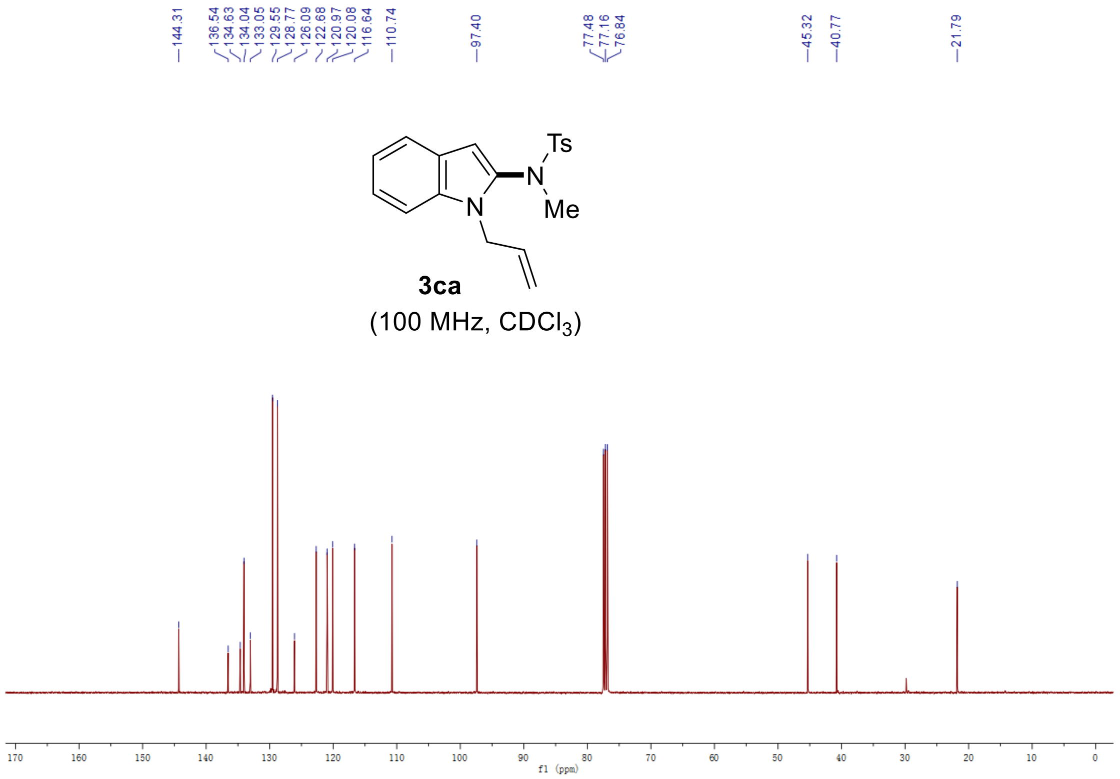
144.31
136.54
134.63
134.04
133.05
129.55
128.77
126.09
122.68
120.97
120.08
116.64
110.74
97.40
77.48
77.16
76.84
45.32
40.77
21.79
Ts
N
Me
N
3ca
(100 MHz, CDCl3)
f1 (ppm)

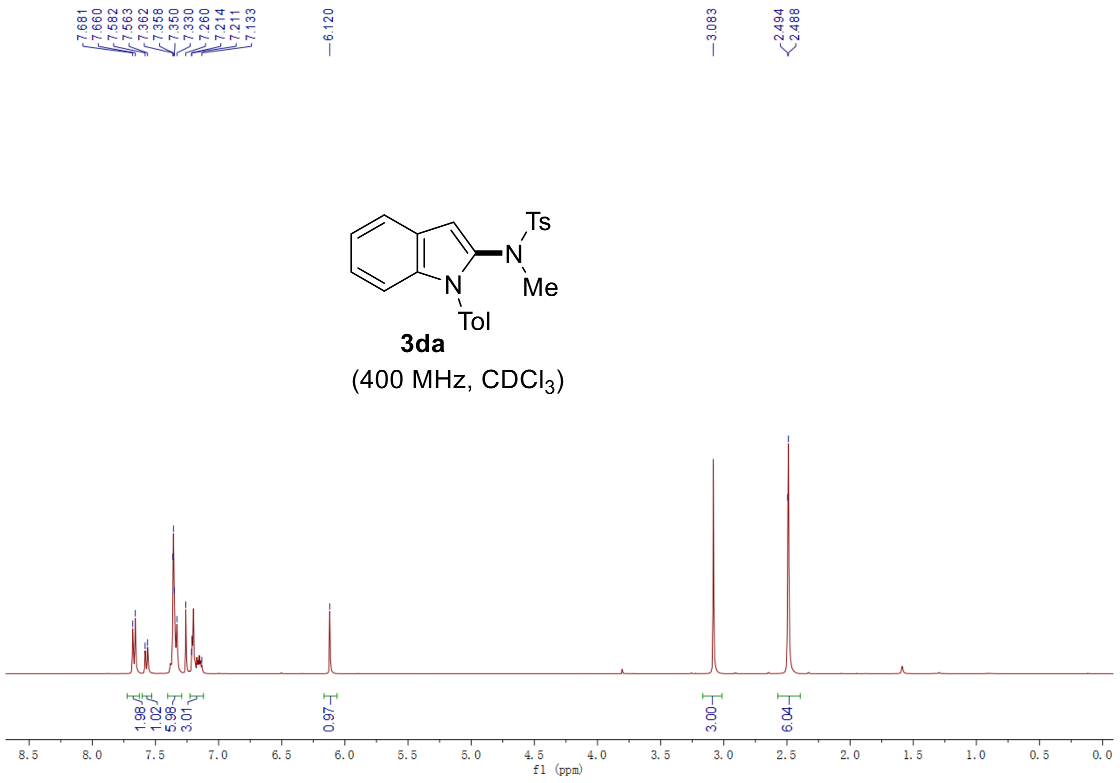
7.681
7.660
7.582
7.563
7.362
7.358
7.350
7.330
7.260
7.214
7.211
7.133
6.120
3.083
2.494
2.488
Ts
N
Me
N
Tol
3da
(400 MHz, CDCl3)
1.98
1.02
5.98
3.01
0.97
3.00
6.04
f1 (ppm)

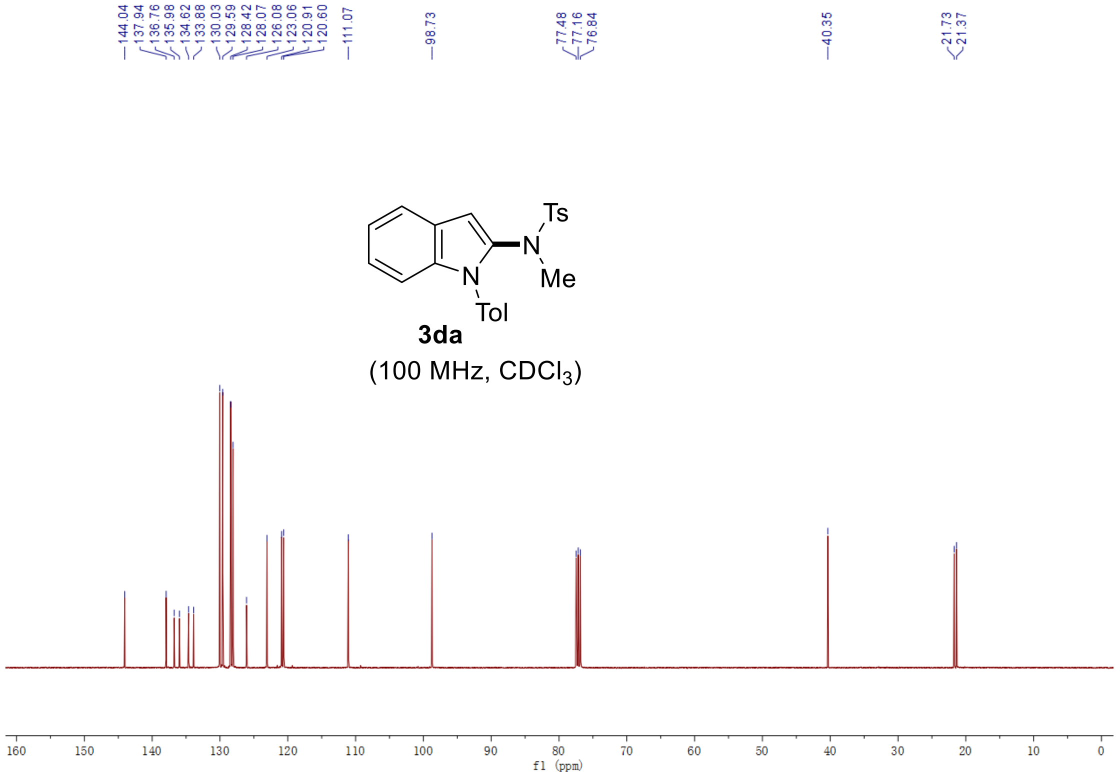
144.04
137.94
136.76
135.98
134.62
133.88
130.03
129.59
128.42
128.07
126.08
123.06
120.91
120.60
111.07
98.73
77.48
77.16
76.84
40.35
21.73
21.37
Ts
N
Me
N
Tol
3da
(100 MHz, CDCl3)
f1 (ppm)

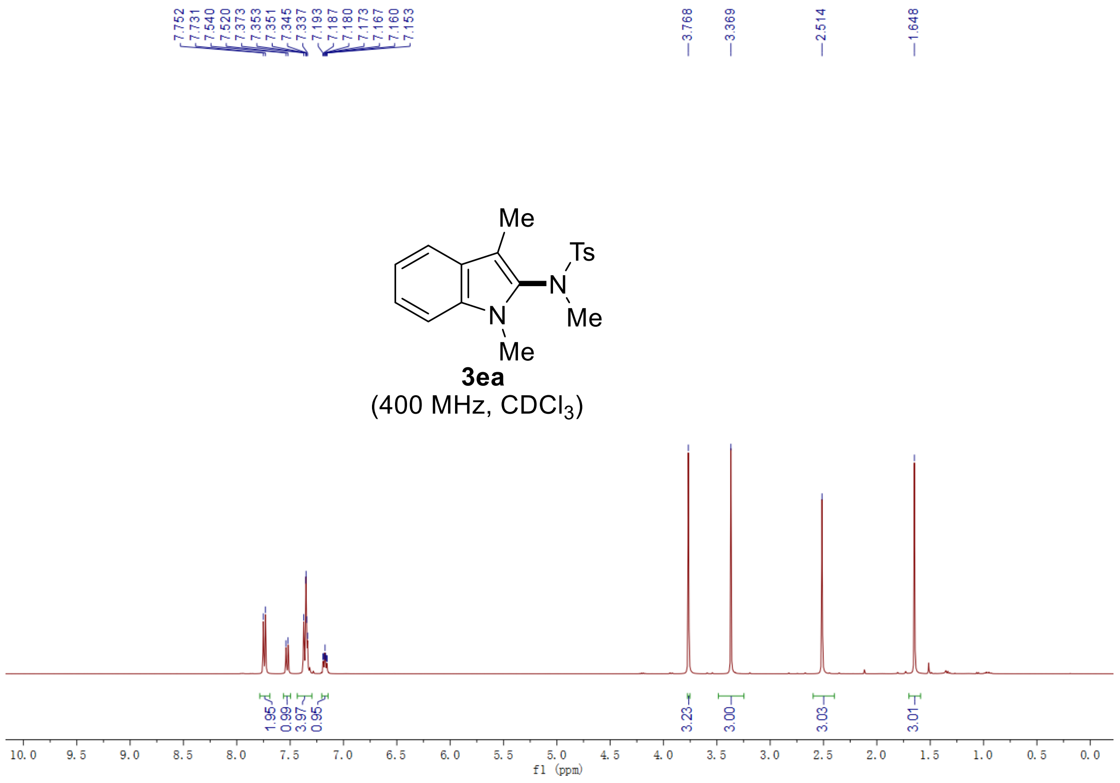
Me
Ts
N
Me
N
Me
3ea
(400 MHz, CDCl3)
f1 (ppm)

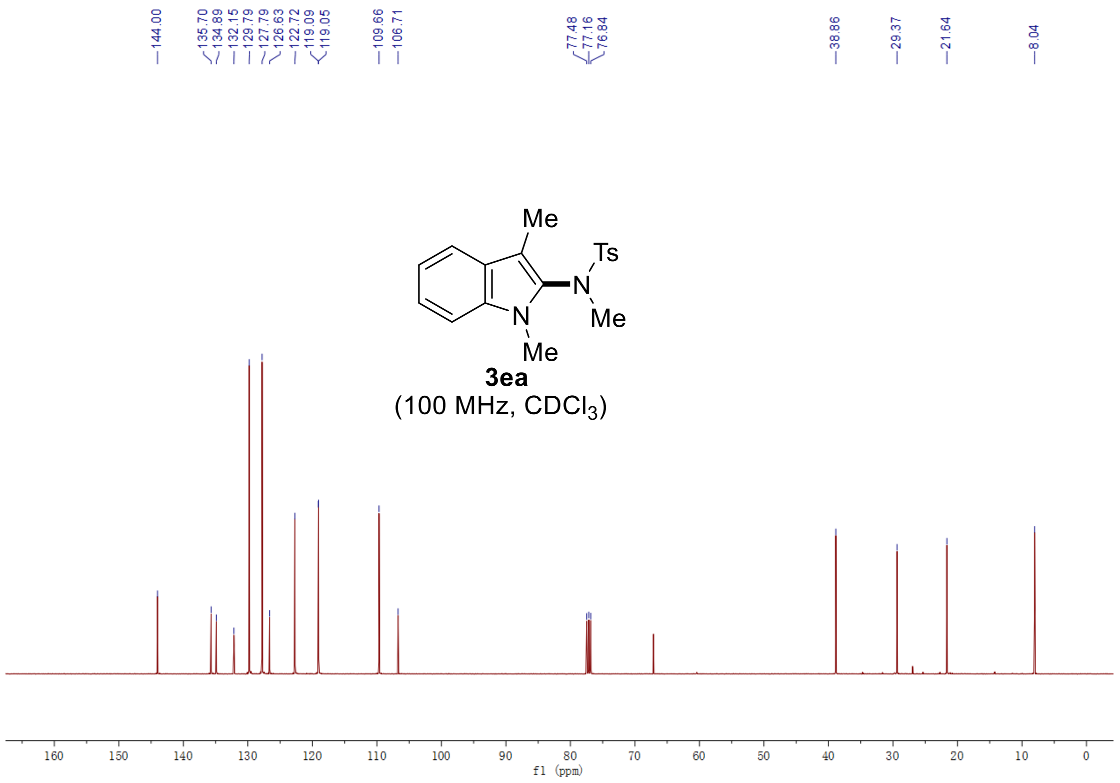
Me
Ts
N
Me
N
Me
3ea
(100 MHz, CDCl3)
f1 (ppm)

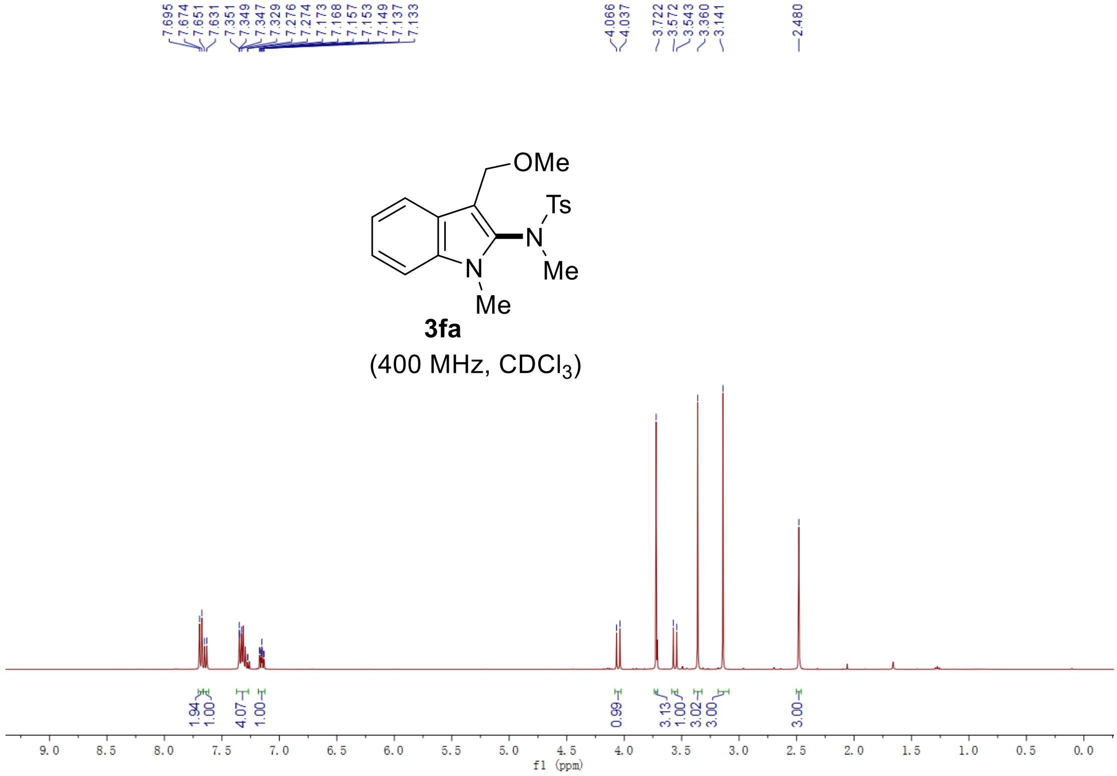
7.695
7.674
7.651
7.631
7.351
7.349
7.347
7.329
7.276
7.274
7.173
7.168
7.157
7.153
7.149
7.137
7.133
4.066
4.037
3.722
3.572
3.543
3.360
3.141
2.480
OMe
Ts
N
N
Me
Me
3fa
(400 MHz, CDCl3)
1.94
1.00
4.07
1.00
0.99
3.13
1.00
3.02
3.00
3.00
f1 (ppm)

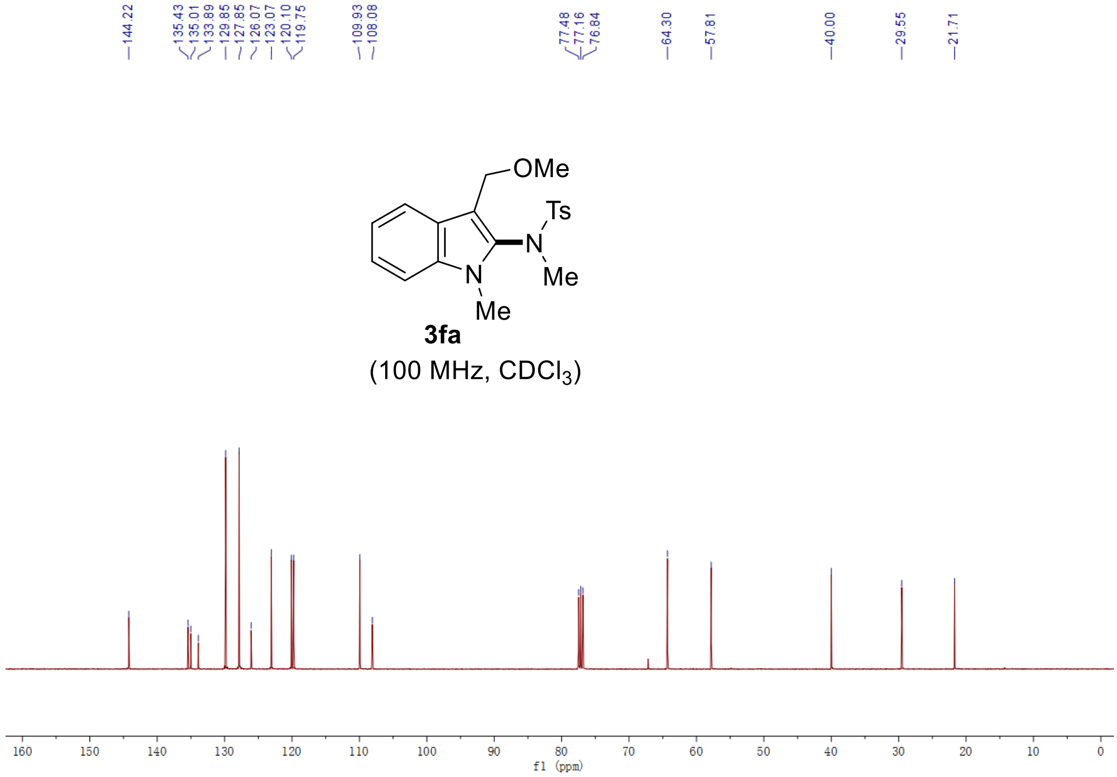
144.22
135.43
135.01
133.89
129.85
127.85
126.07
123.07
120.10
119.75
109.93
108.08
77.48
77.16
76.84
64.30
57.81
40.00
29.55
21.71
OMe
Ts
N
N
Me
Me
3fa
(100 MHz, CDCl3)
f1 (ppm)

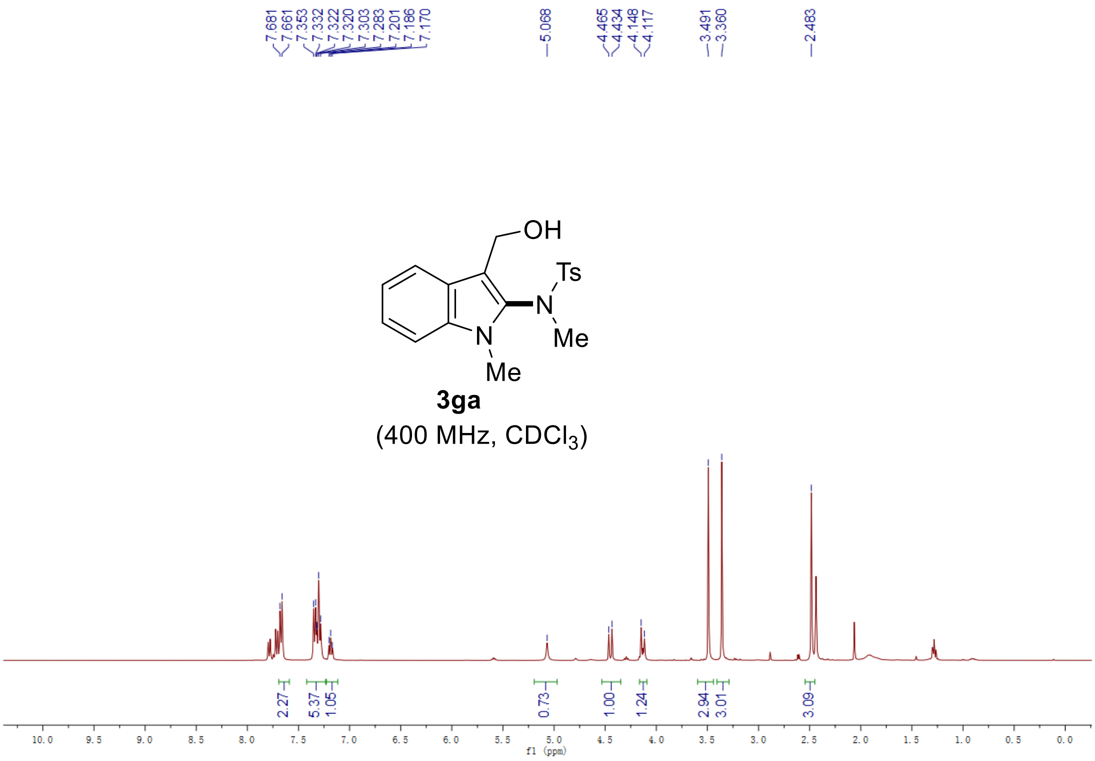
7.681
7.661
7.353
7.332
7.322
7.320
7.303
7.283
7.201
7.186
7.170
5.068
4.465
4.434
4.148
4.117
3.491
3.360
2.483
OH
Ts
N
N
Me
Me
3ga
(400 MHz, CDCl3)
2.27
5.37
1.05
0.73
1.00
1.24
2.94
3.01
3.09
f1 (ppm)

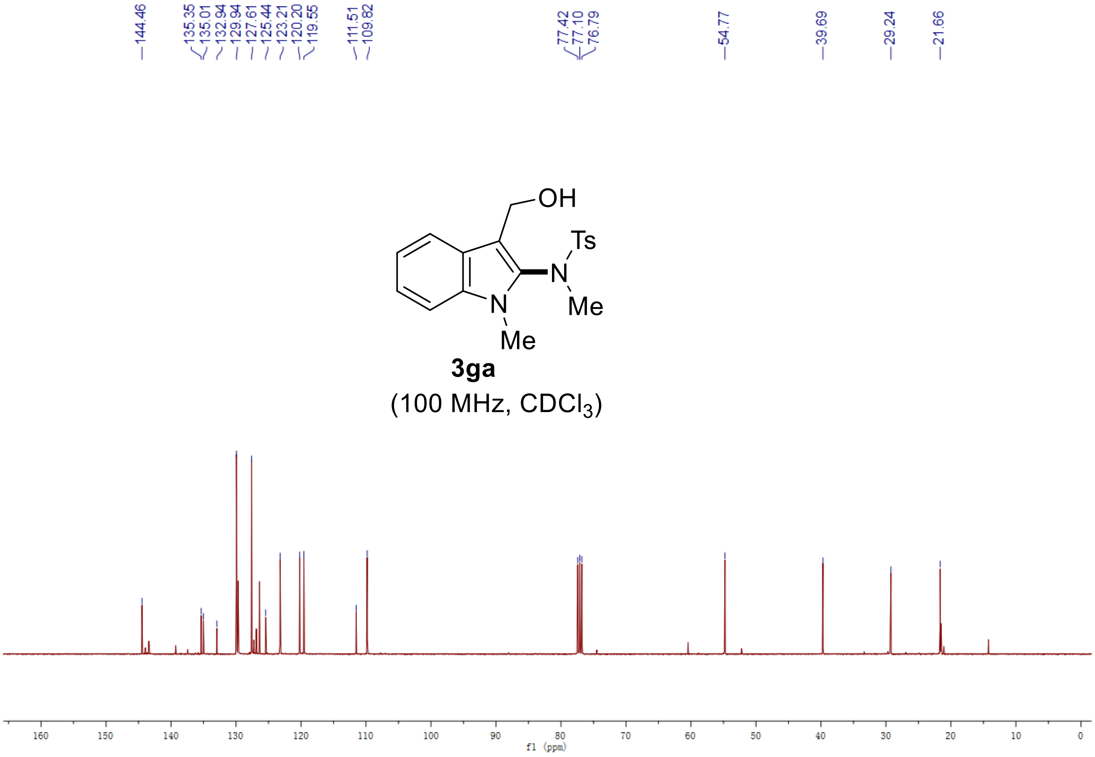
144.46
135.35
135.01
132.94
129.94
127.61
125.44
123.21
120.20
119.55
111.51
109.82
77.42
77.10
76.79
54.77
39.69
29.24
21.66
OH
Ts
N
N
Me
Me
3ga
(100 MHz, CDCl3)
f1 (ppm)

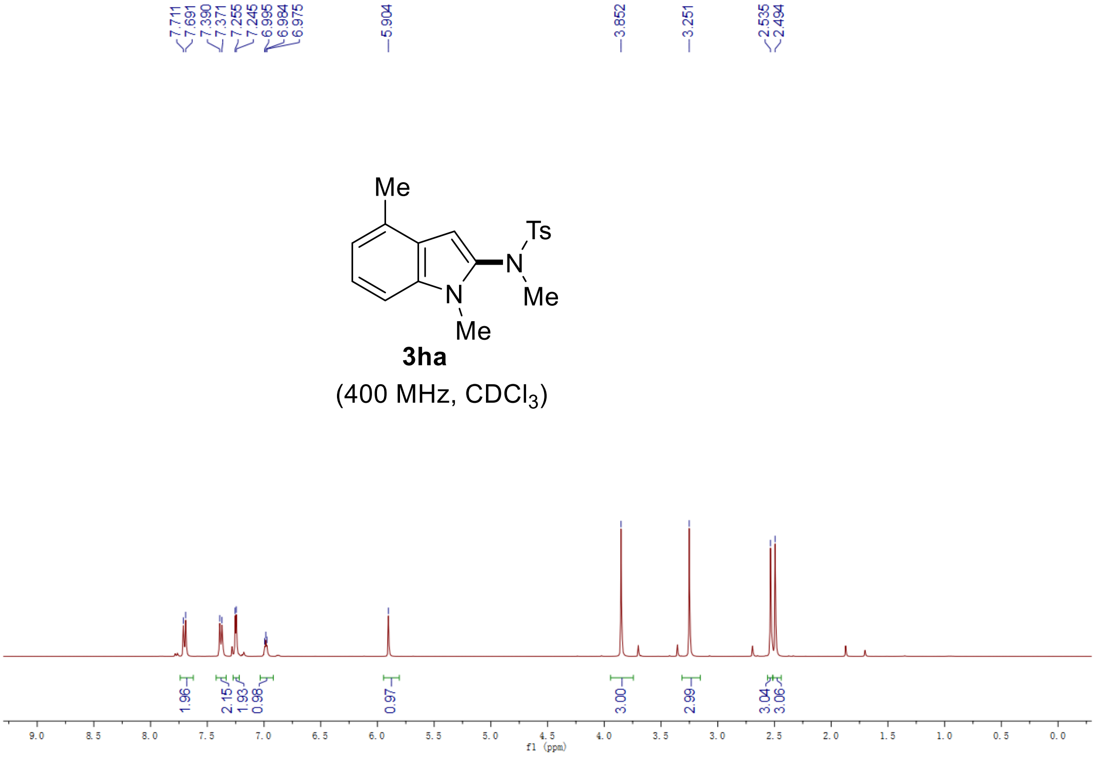

3ha
(400 MHz, $CDCl_3$)

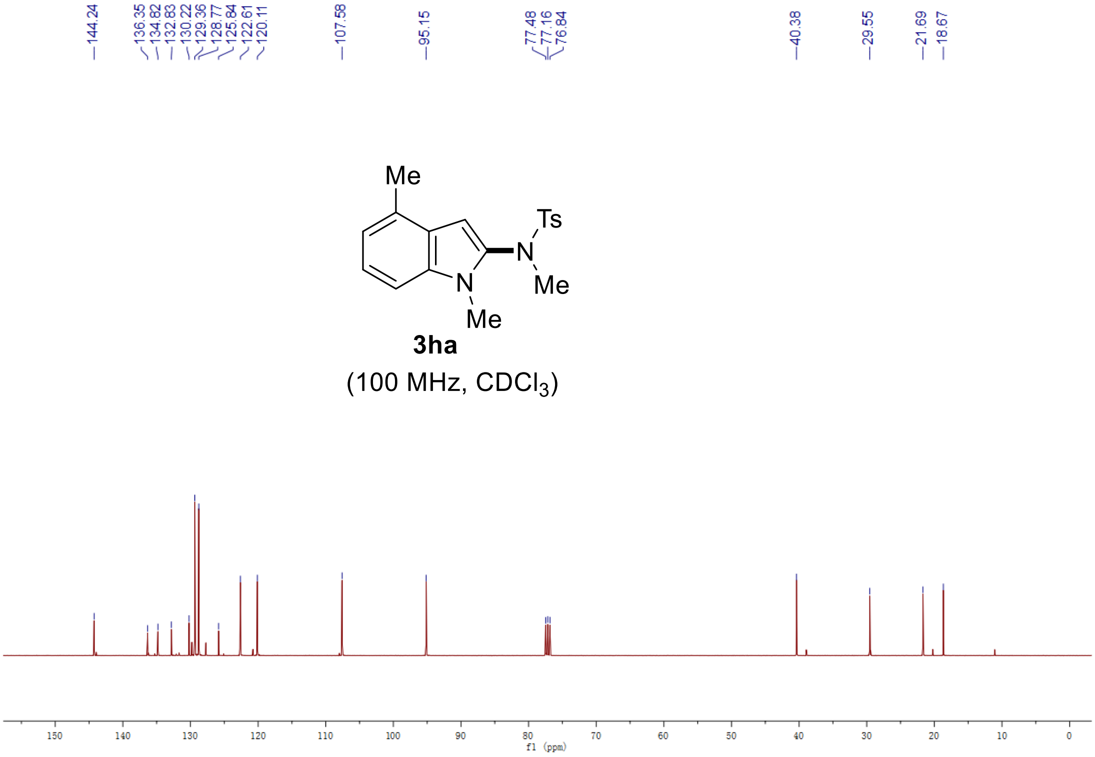

3ha
(100 MHz, $CDCl_3$)

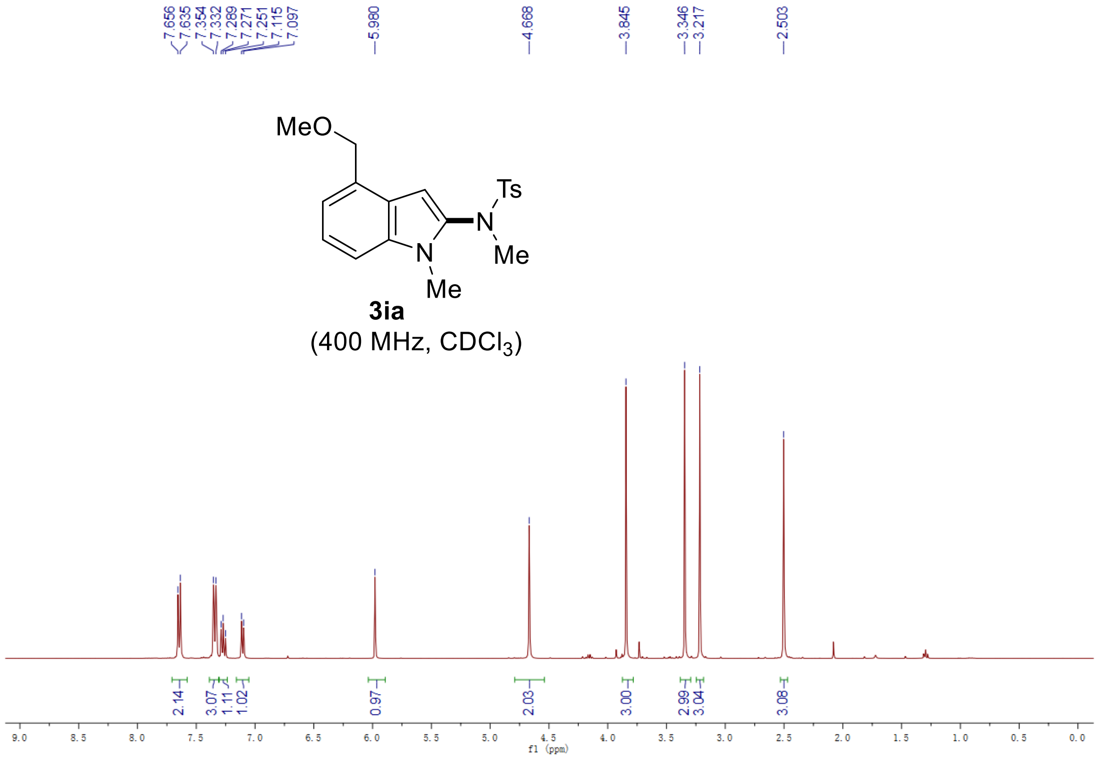
7.656
7.635
7.354
7.332
7.289
7.271
7.251
7.115
7.097
5.980
4.668
3.845
3.346
3.217
2.503
MeO
Ts
N
N
Me
Me
3ia
(400 MHz, CDCl3)
2.14
3.07
1.11
1.02
0.97
2.03
3.00
2.99
3.04
3.08
f1 (ppm)

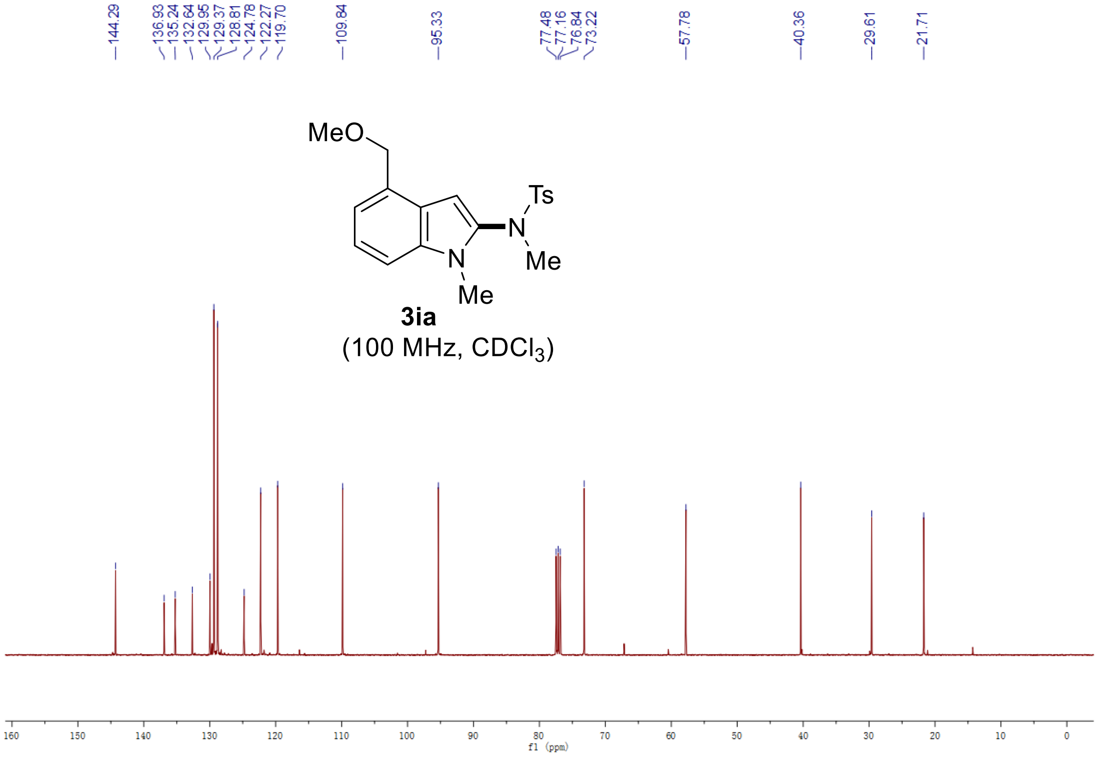
144.29
136.93
135.24
132.64
129.95
129.37
128.81
124.78
122.27
119.70
109.84
95.33
77.48
77.16
76.84
73.22
57.78
40.36
29.61
21.71
MeO
Ts
N
N
Me
Me
3ia
(100 MHz, CDCl3)
f1 (ppm)

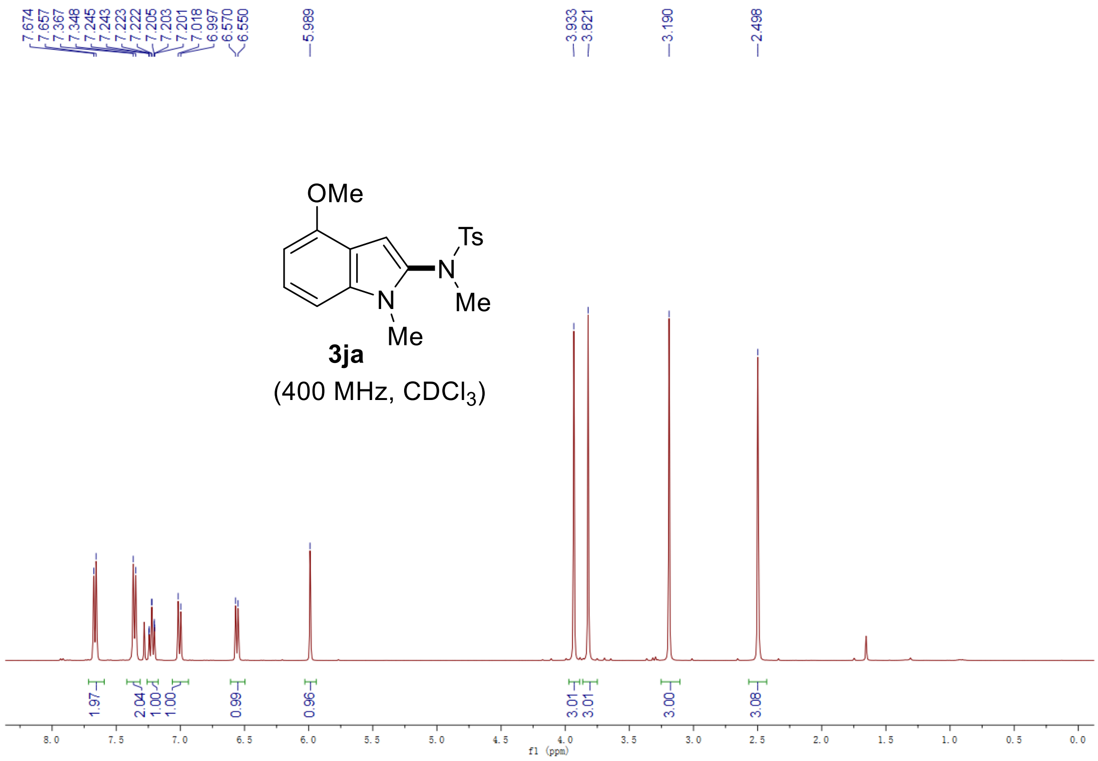
OMe
Ts
N
Me
N
Me
3ja
(400 MHz, CDCl3)
7.674
7.657
7.367
7.348
7.245
7.243
7.223
7.222
7.205
7.203
7.201
7.018
6.997
6.570
6.550
5.989
3.933
3.821
3.190
2.498
1.97
2.04
1.00
1.00
0.99
0.96
3.01
3.01
3.00
3.08
f1 (ppm)

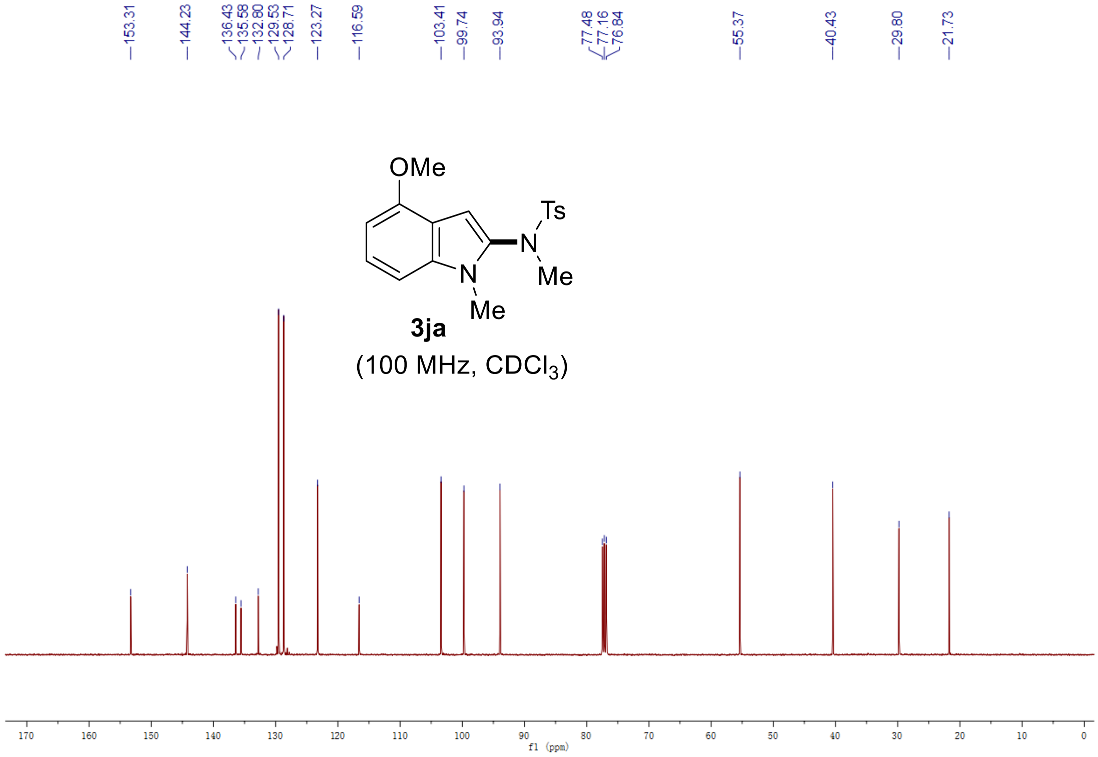
OMe
Ts
N
Me
N
Me
3ja
(100 MHz, CDCl3)
153.31
144.23
136.43
135.58
132.80
129.53
128.71
123.27
116.59
103.41
99.74
93.94
77.48
77.16
76.84
55.37
40.43
29.80
21.73
f1 (ppm)

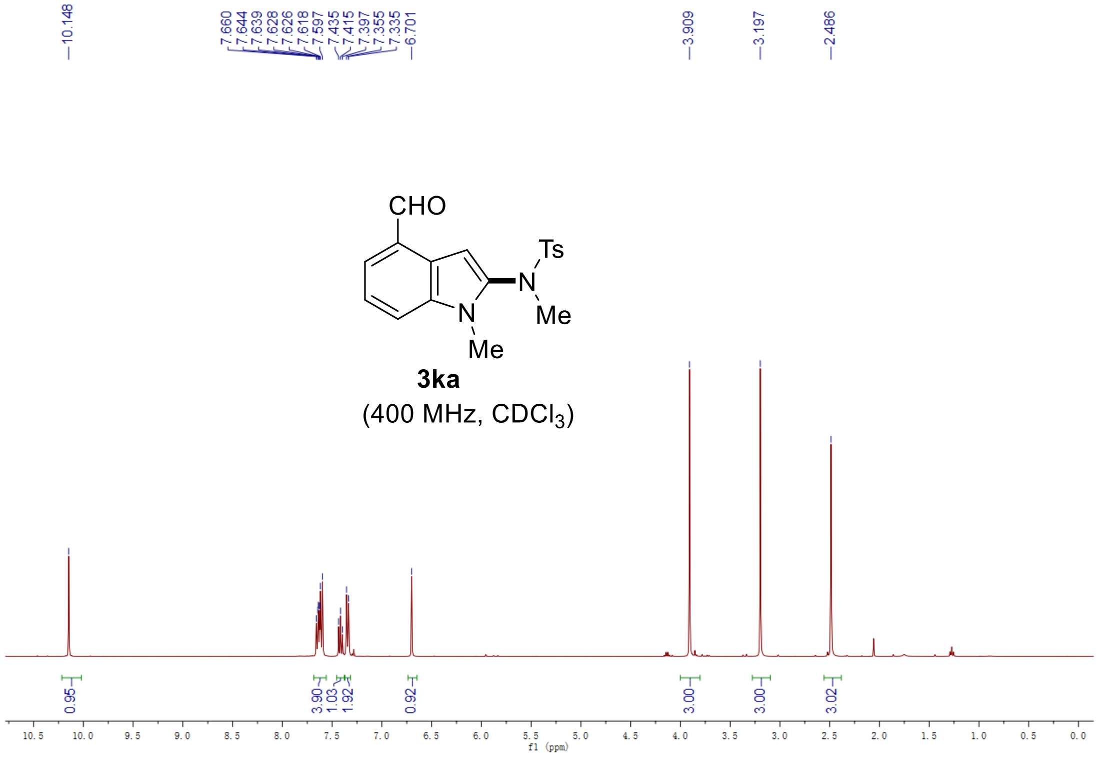

3ka
(400 MHz, $CDCl_3$)

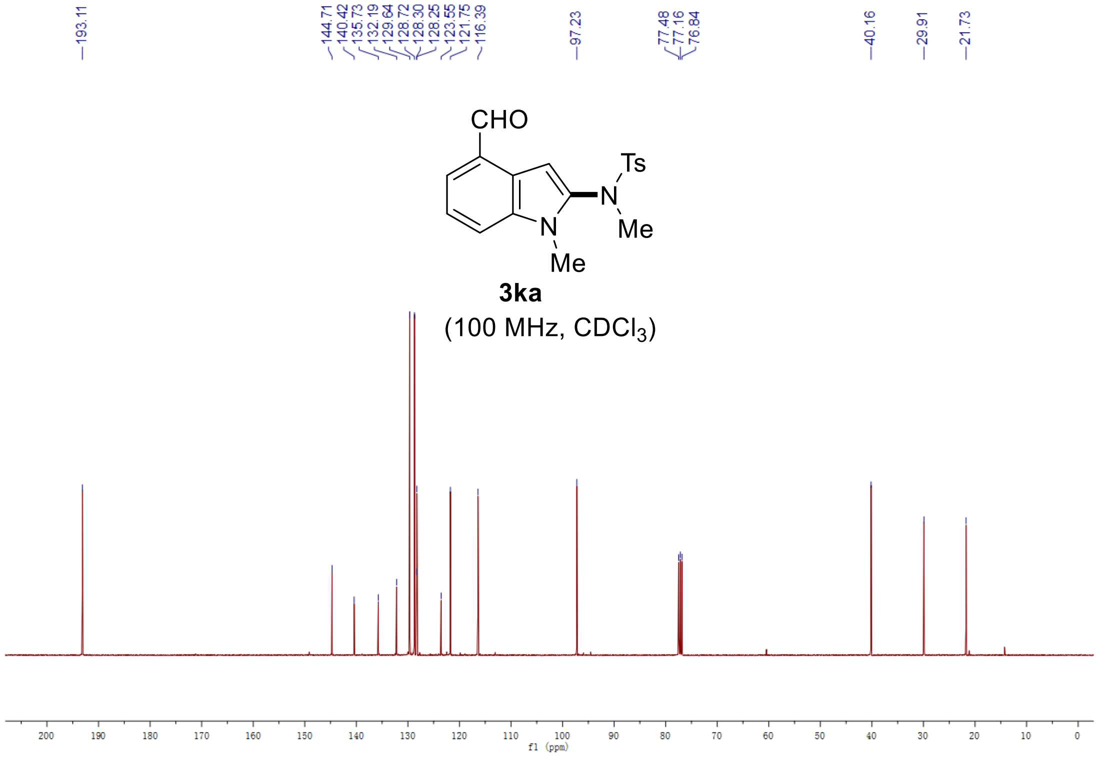

3ka
(100 MHz, $CDCl_3$)

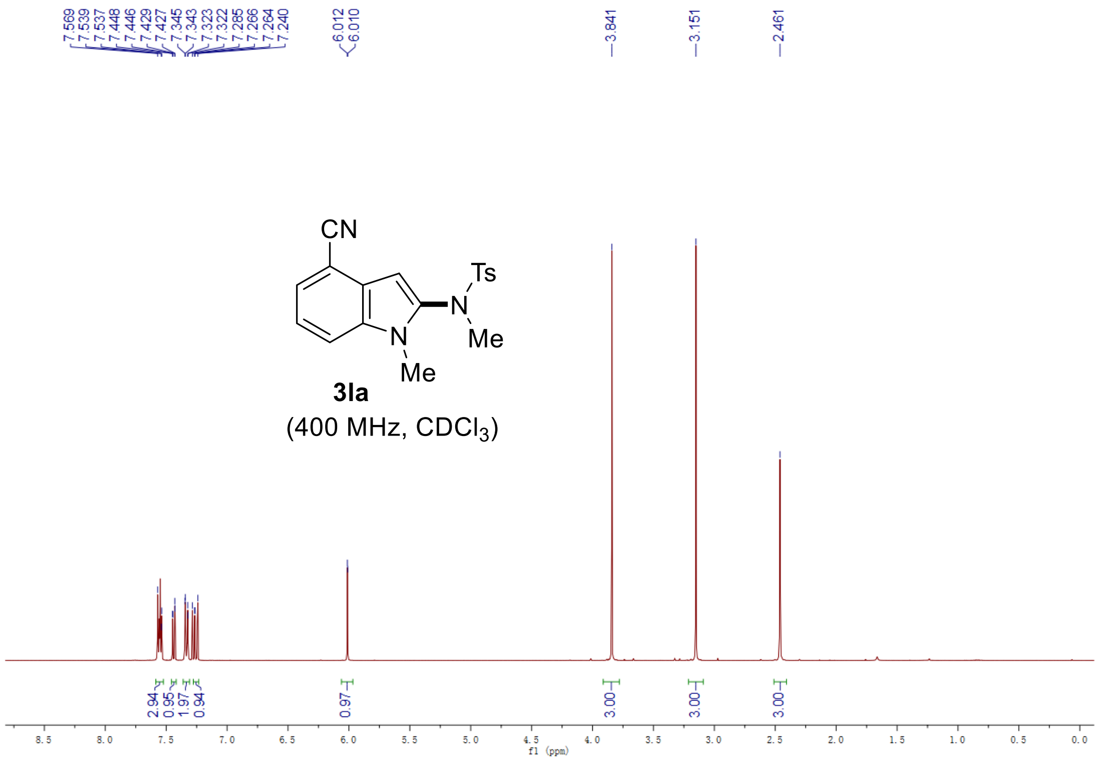
7.569
7.539
7.537
7.448
7.446
7.429
7.427
7.345
7.343
7.323
7.322
7.285
7.266
7.264
7.240
6.012
6.010
3.841
3.151
2.461
CN
Ts
N
N
Me
Me
3la
(400 MHz, CDCl3)
2.94
0.95
1.97
0.94
0.97
3.00
3.00
3.00
f1 (ppm)

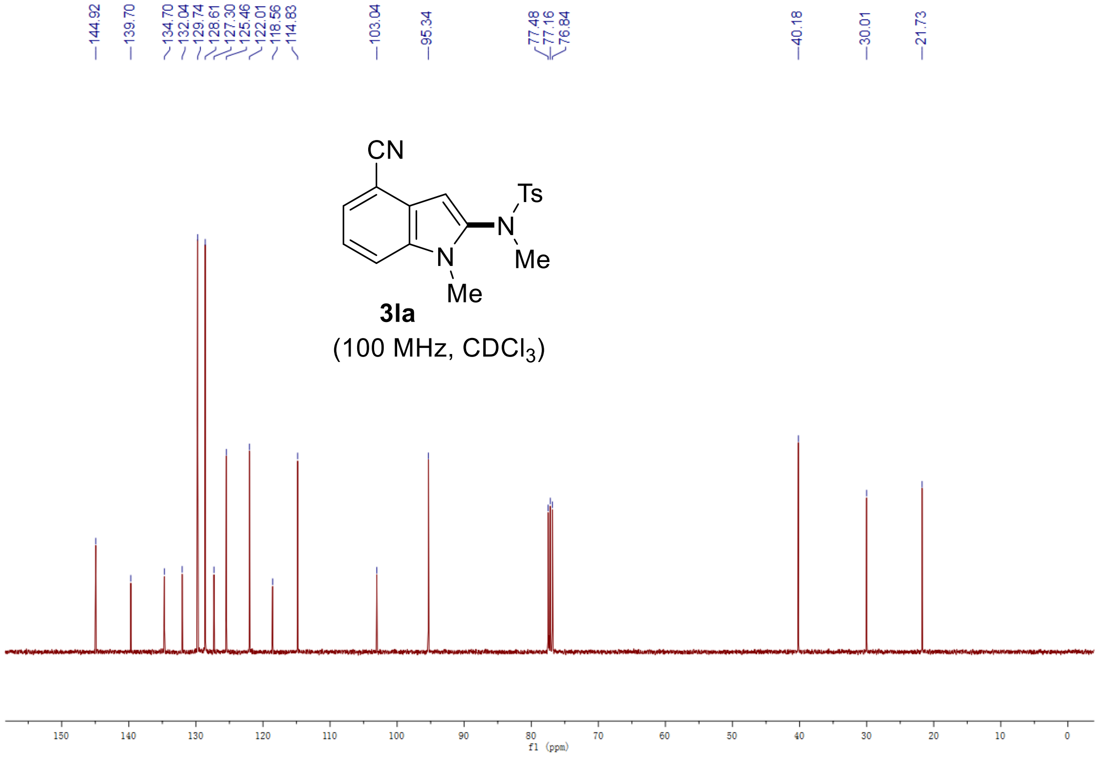
144.92
139.70
134.70
132.04
129.74
128.61
127.30
125.46
122.01
118.56
114.83
103.04
95.34
77.48
77.16
76.84
40.18
30.01
21.73
CN
Ts
N
N
Me
Me
3la
(100 MHz, CDCl3)
f1 (ppm)

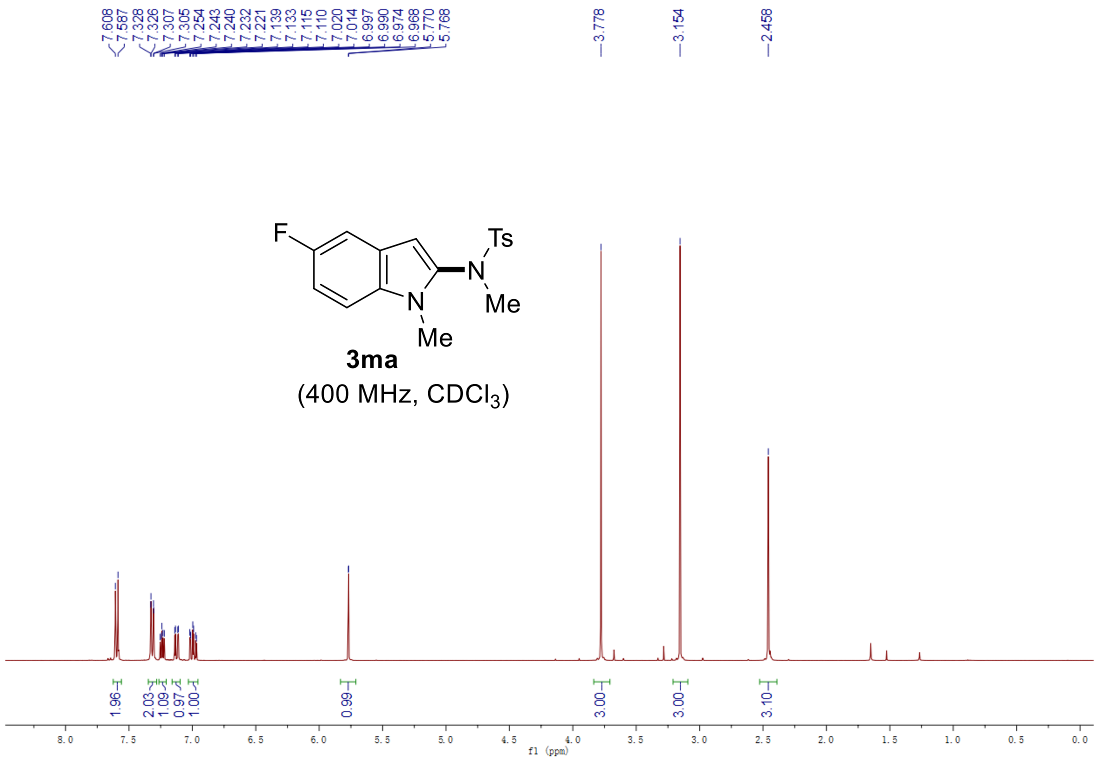
7.608
7.587
7.328
7.326
7.307
7.305
7.254
7.243
7.240
7.232
7.221
7.139
7.133
7.115
7.110
7.020
7.014
6.997
6.990
6.974
6.968
5.770
5.768
3.778
3.154
2.458
F
Ts
N
N
Me
Me
3ma
(400 MHz, CDCl3)
1.96
2.03
1.09
0.97
1.00
0.99
3.00
3.00
3.10
f1 (ppm)

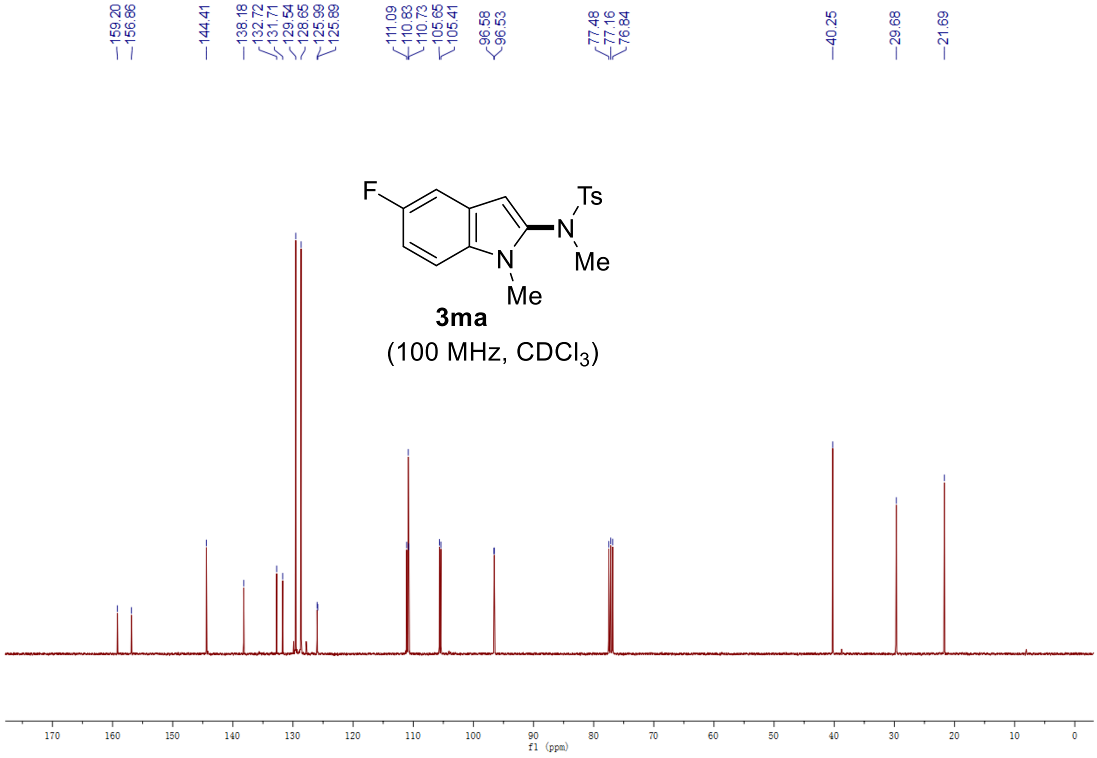
159.20
156.86
144.41
138.18
132.72
131.71
129.54
128.65
125.99
125.89
111.09
110.83
110.73
105.65
105.41
96.58
96.53
77.48
77.16
76.84
40.25
29.68
21.69
F
Ts
N
N
Me
Me
3ma
(100 MHz, CDCl3)
f1 (ppm)

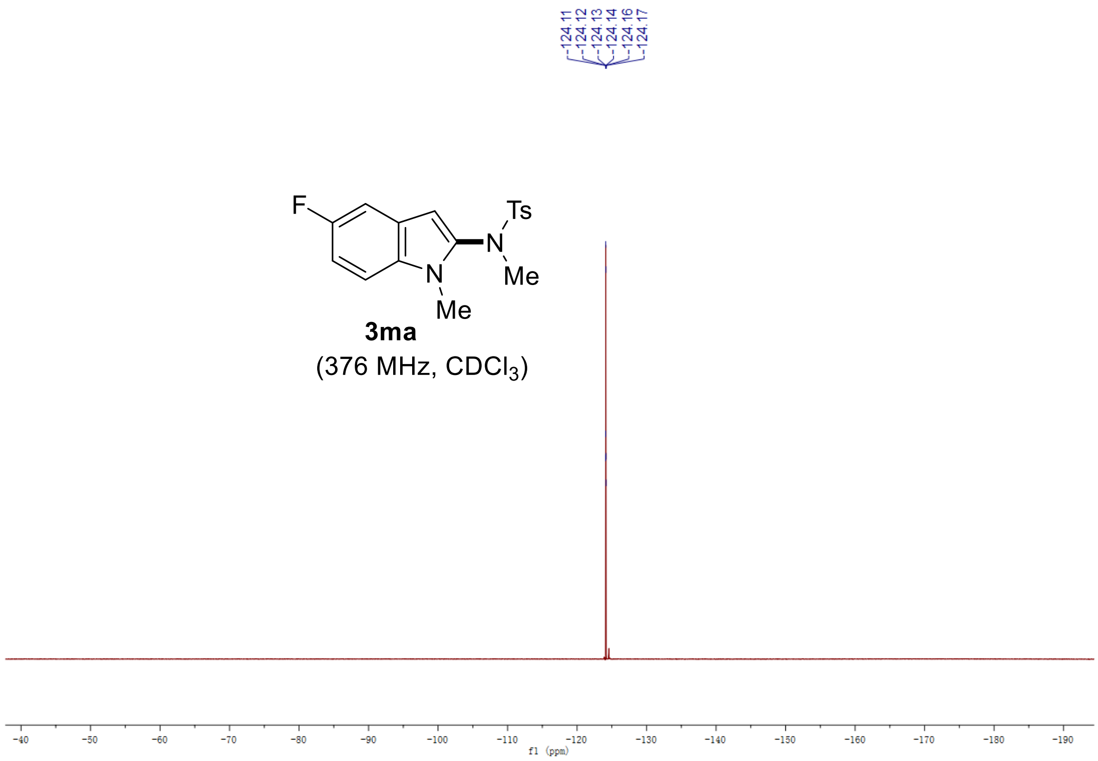

-124.11
-124.12
-124.13
-124.14
-124.16
-124.17
F
Ts
N
Me
N
Me
3ma
(376 MHz, CDCl3)
f1 (ppm)

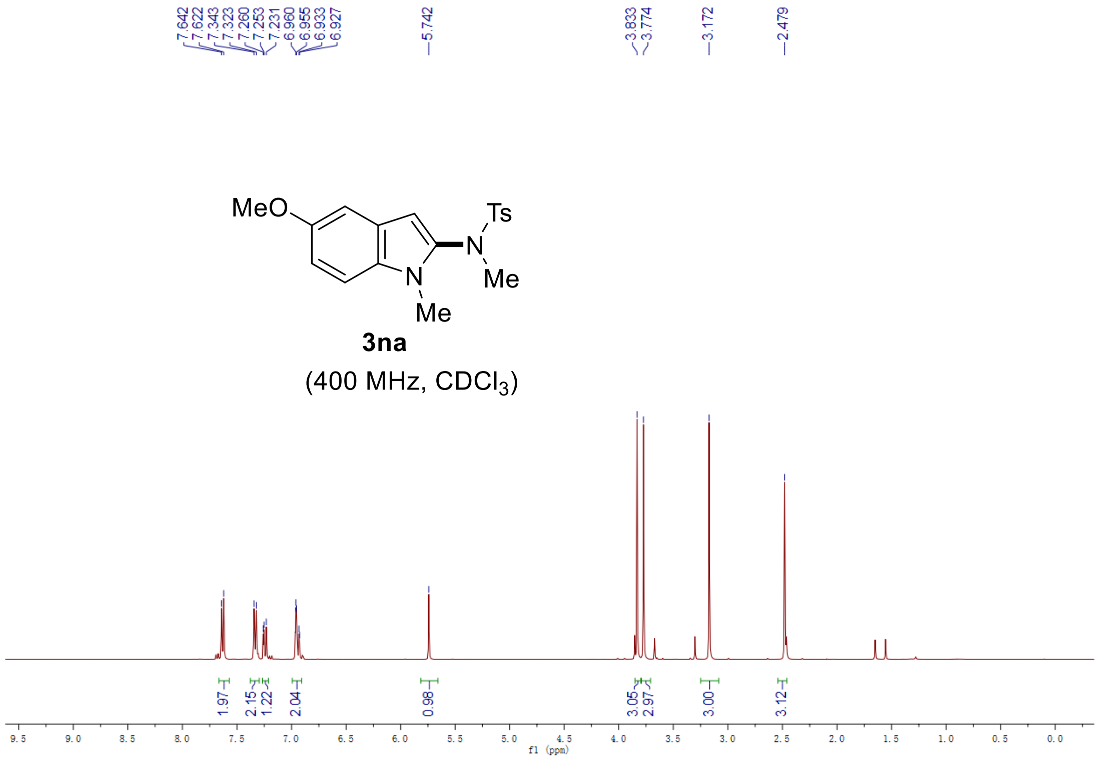

7.642
7.622
7.343
7.323
7.260
7.253
7.231
6.960
6.955
6.933
6.927
5.742
3.833
3.774
3.172
2.479
MeO
Ts
N
Me
N
Me
3na
(400 MHz, CDCl3)
1.97
2.15
1.22
2.04
0.98
3.05
2.97
3.00
3.12
f1 (ppm)

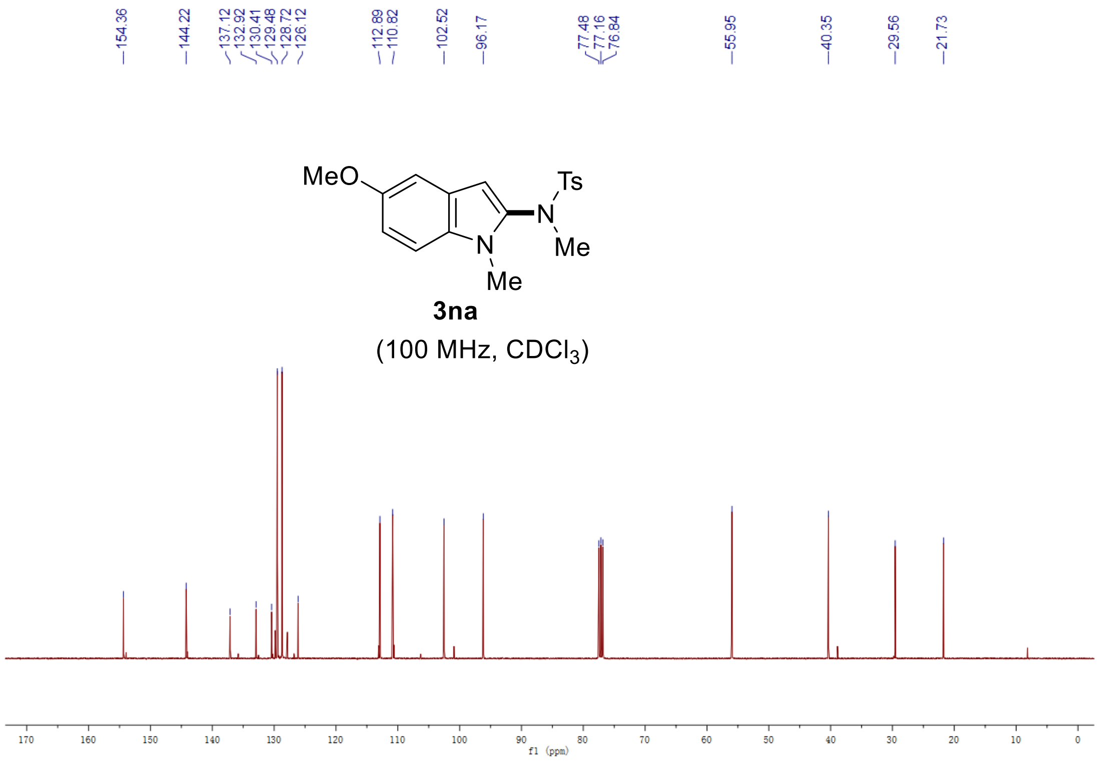
154.36
144.22
137.12
132.92
130.41
129.48
128.72
126.12
112.89
110.82
102.52
96.17
77.48
77.16
76.84
55.95
40.35
29.56
21.73
MeO
Ts
N
Me
N
Me
3na
(100 MHz, CDCl$_3$)
f1 (ppm)

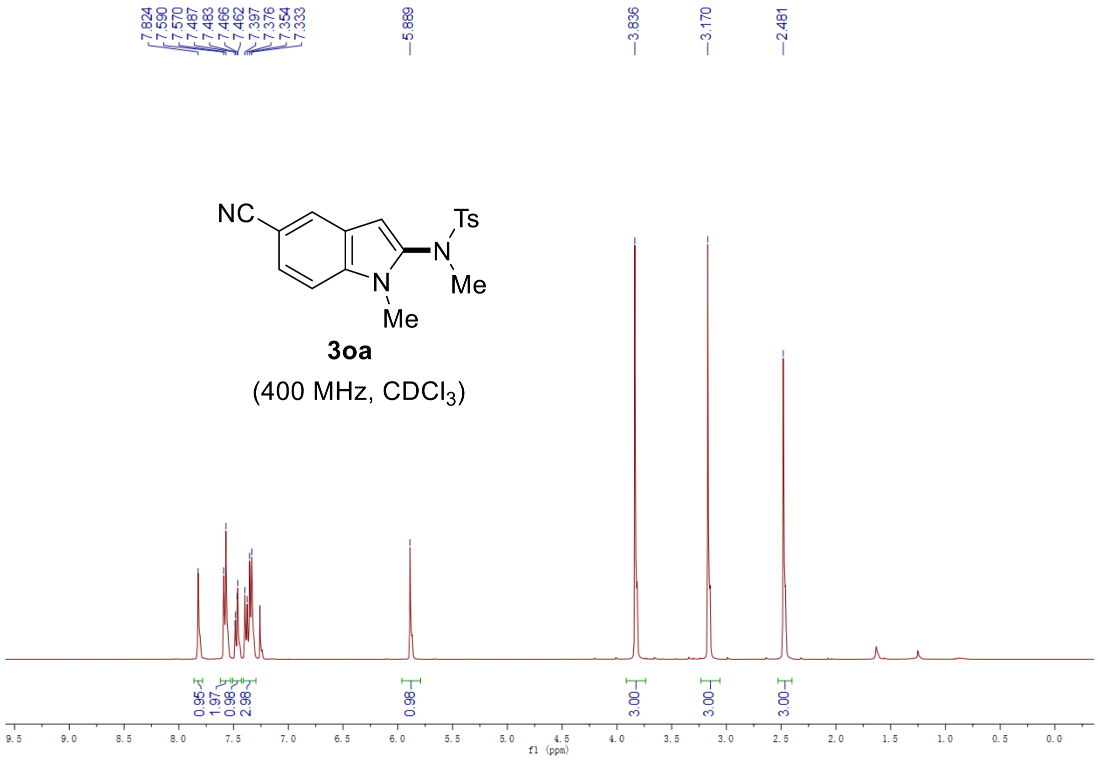
7.824
7.590
7.570
7.487
7.483
7.466
7.462
7.397
7.376
7.354
7.333
5.889
3.836
3.170
2.481
NC
Ts
N
Me
N
Me
3oa
(400 MHz, CDCl$_3$)
0.95
1.97
0.98
2.98
0.98
3.00
3.00
3.00
f1 (ppm)

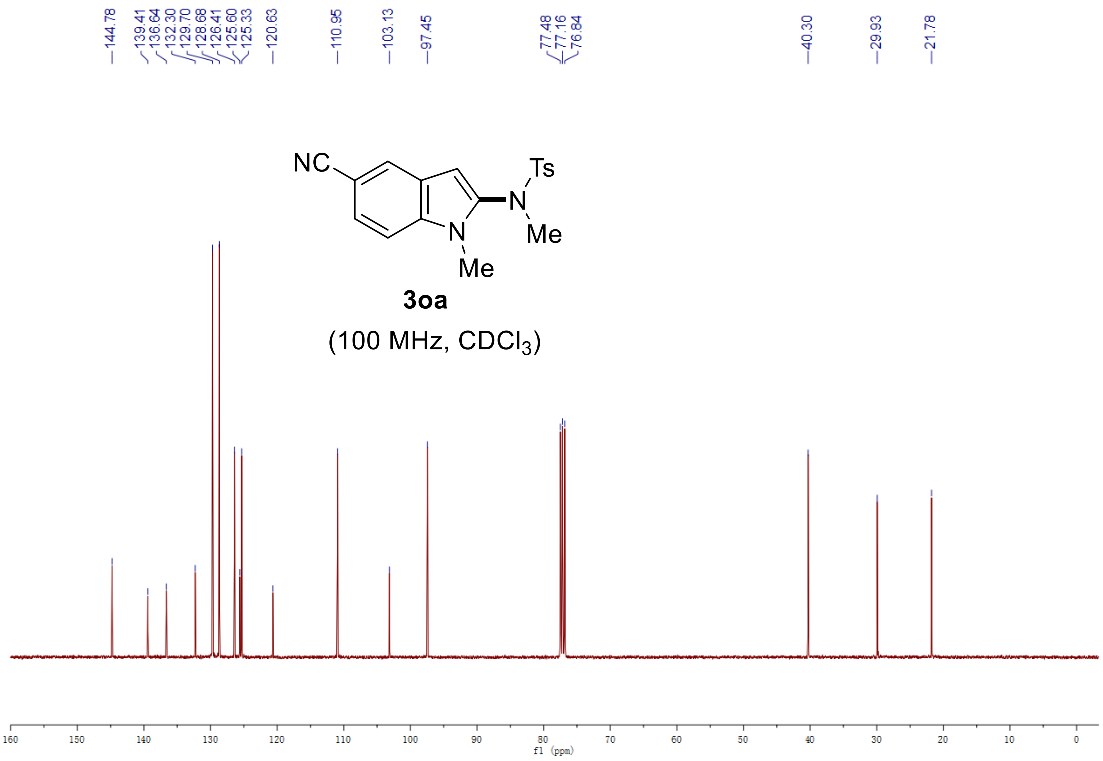

144.78
139.41
136.64
132.30
129.70
128.68
126.41
125.60
125.33
120.63
110.95
103.13
97.45
77.48
77.16
76.84
40.30
29.93
21.78
NC
Ts
N
Me
N
Me
3oa
(100 MHz, CDCl3)
f1 (ppm)

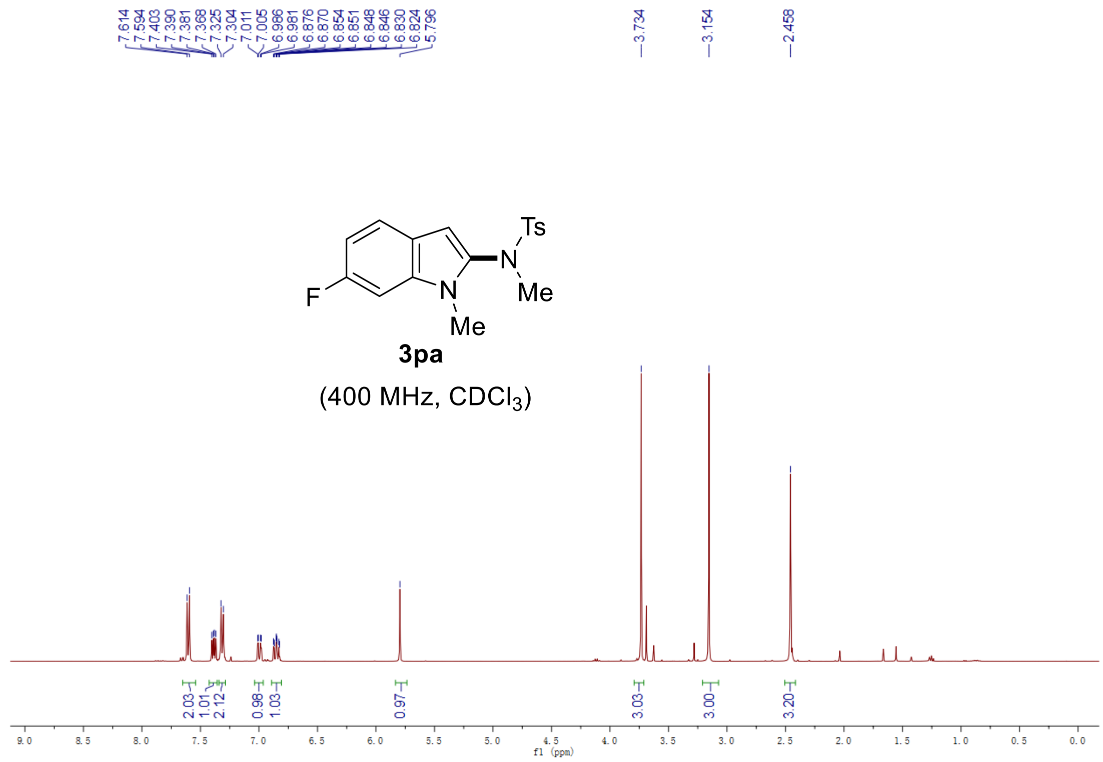

7.614
7.594
7.403
7.390
7.381
7.368
7.325
7.304
7.011
7.005
6.986
6.981
6.876
6.870
6.854
6.851
6.848
6.846
6.830
6.824
5.796
3.734
3.154
2.458
Ts
N
Me
F
N
Me
3pa
(400 MHz, CDCl3)
2.03
1.01
2.12
0.98
1.03
0.97
3.03
3.00
3.20
f1 (ppm)

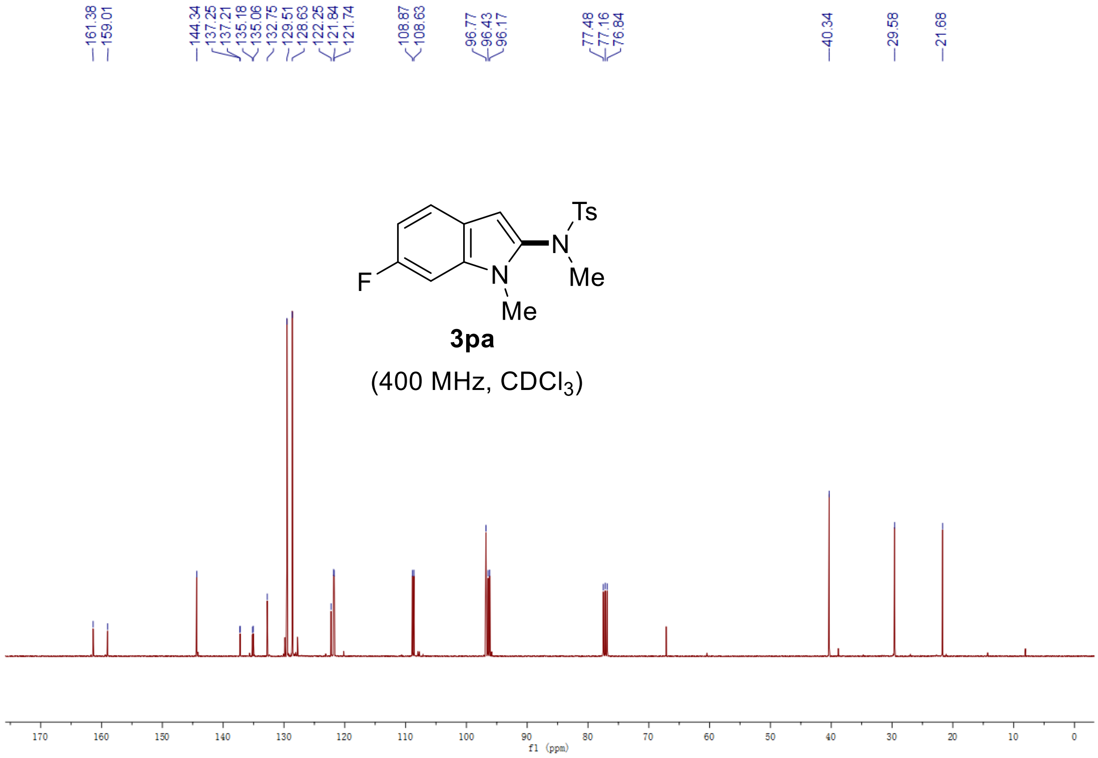
161.38
159.01
144.34
137.25
137.21
135.18
135.06
132.75
129.51
128.63
122.25
121.84
121.74
108.87
108.63
96.77
96.43
96.17
77.48
77.16
76.84
40.34
29.58
21.68
Ts
N
Me
F
N
Me
3pa
(400 MHz, CDCl3)
f1 (ppm)

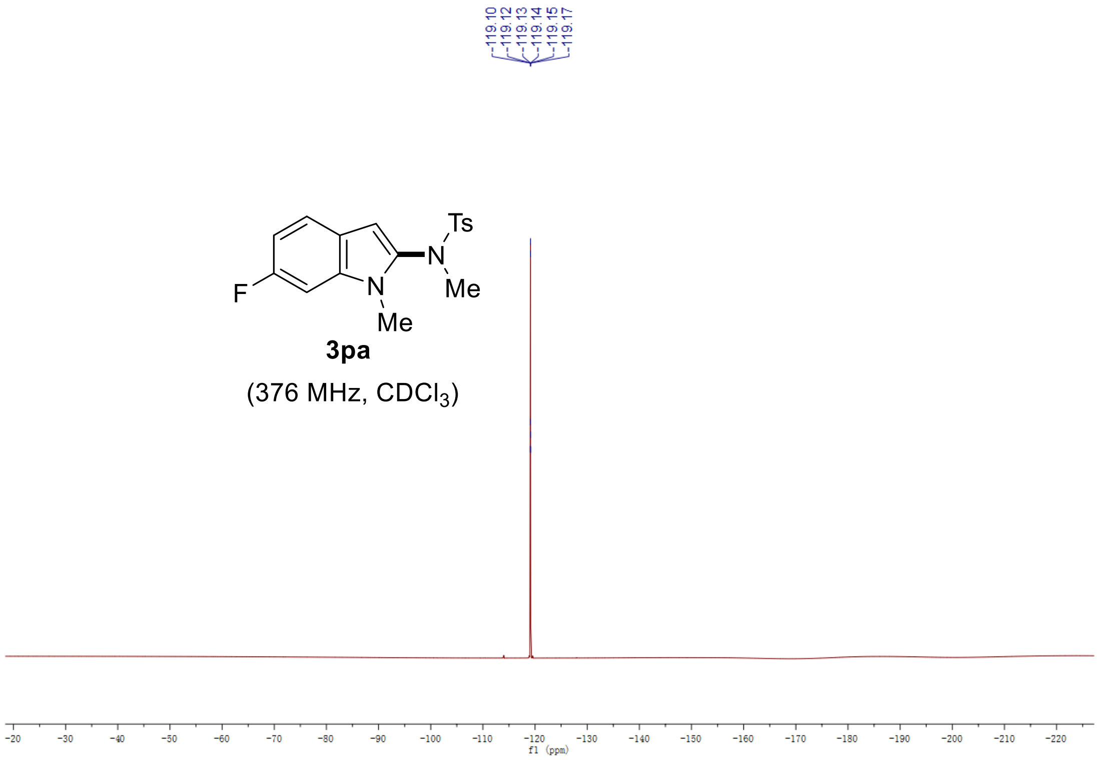
-119.10
-119.12
-119.13
-119.14
-119.15
-119.17
Ts
N
Me
F
N
Me
3pa
(376 MHz, CDCl3)
f1 (ppm)

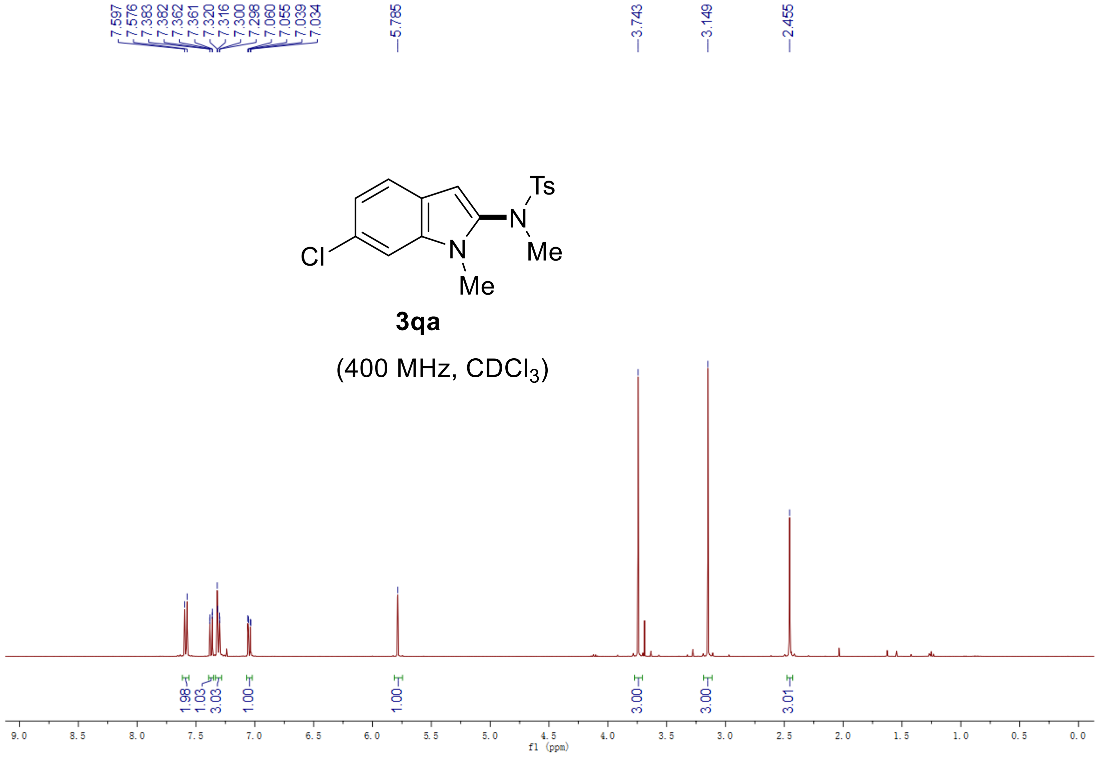

7.597
7.576
7.383
7.382
7.362
7.361
7.320
7.316
7.300
7.298
7.060
7.055
7.039
7.034
5.785
3.743
3.149
2.455
Ts
N
Me
Cl
N
Me
3qa
(400 MHz, CDCl3)
1.98
1.03
3.03
1.00
1.00
3.00
3.00
3.01
f1 (ppm)

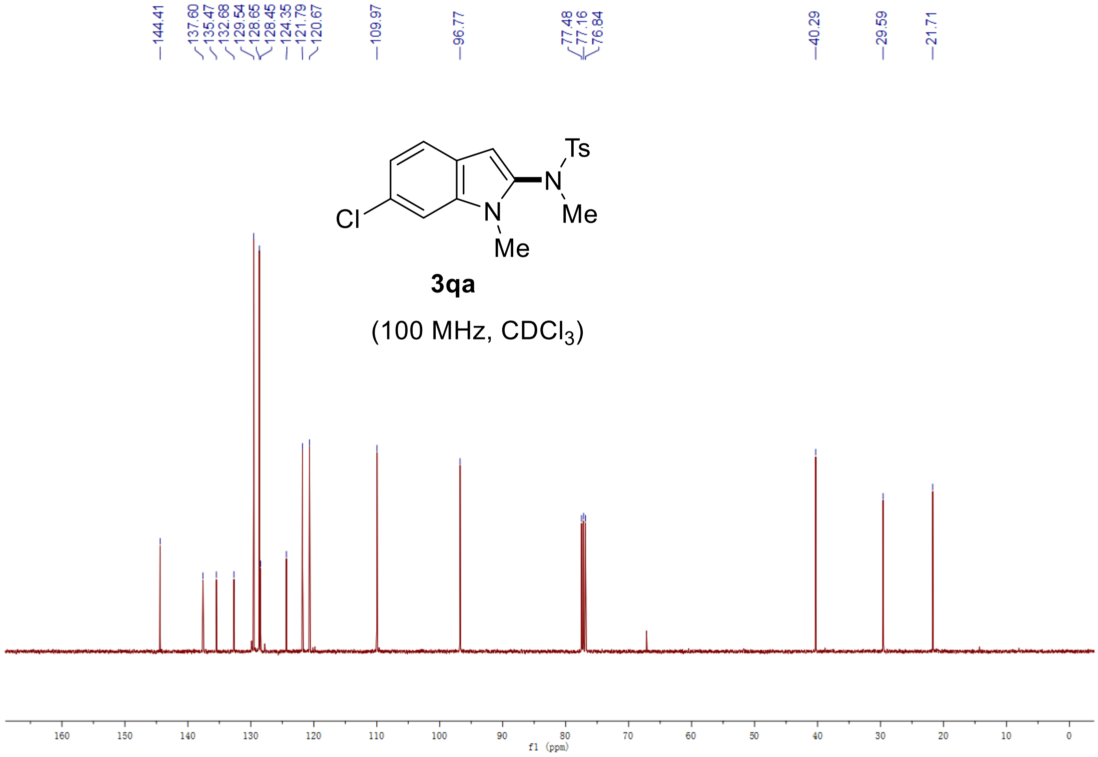

144.41
137.60
135.47
132.68
129.54
128.65
128.45
124.35
121.79
120.67
109.97
96.77
77.48
77.16
76.84
40.29
29.59
21.71
Ts
N
Me
Cl
N
Me
3qa
(100 MHz, CDCl3)
f1 (ppm)

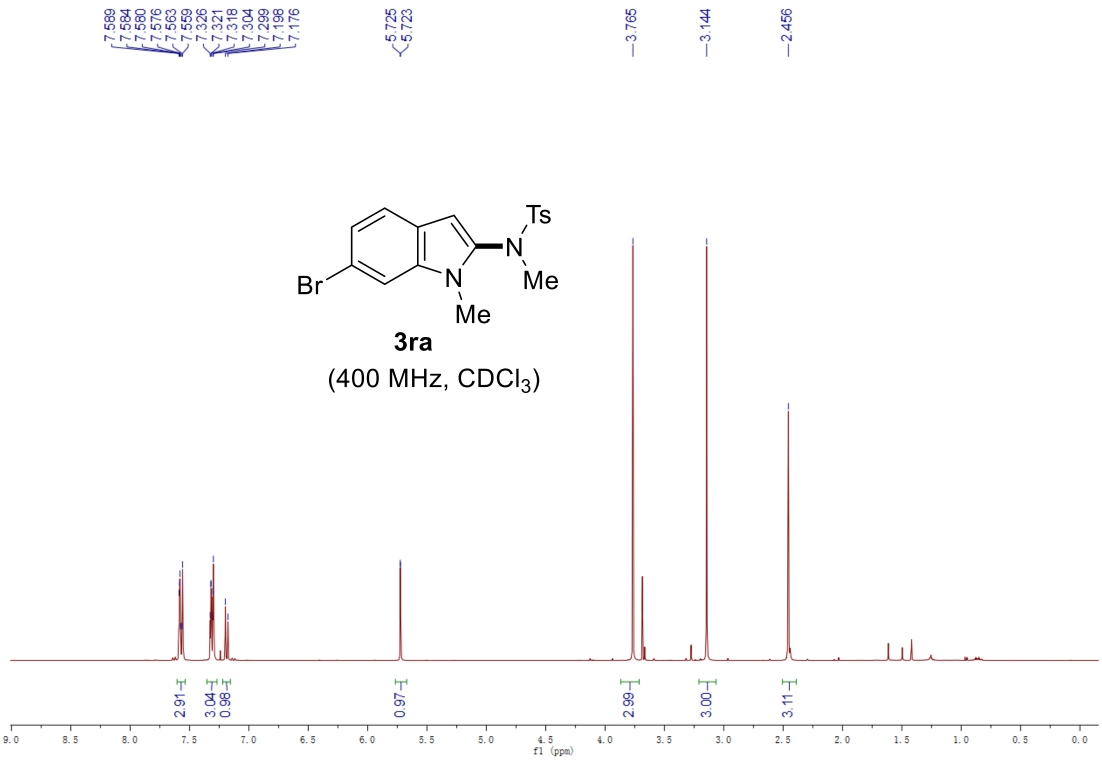
7.589
7.584
7.580
7.576
7.563
7.559
7.326
7.321
7.318
7.304
7.299
7.198
7.176
5.725
5.723
3.765
3.144
2.456
Ts
N
Br
N
Me
Me
3ra
(400 MHz, CDCl3)
2.91
3.04
0.98
0.97
2.99
3.00
3.11
f1 (ppm)

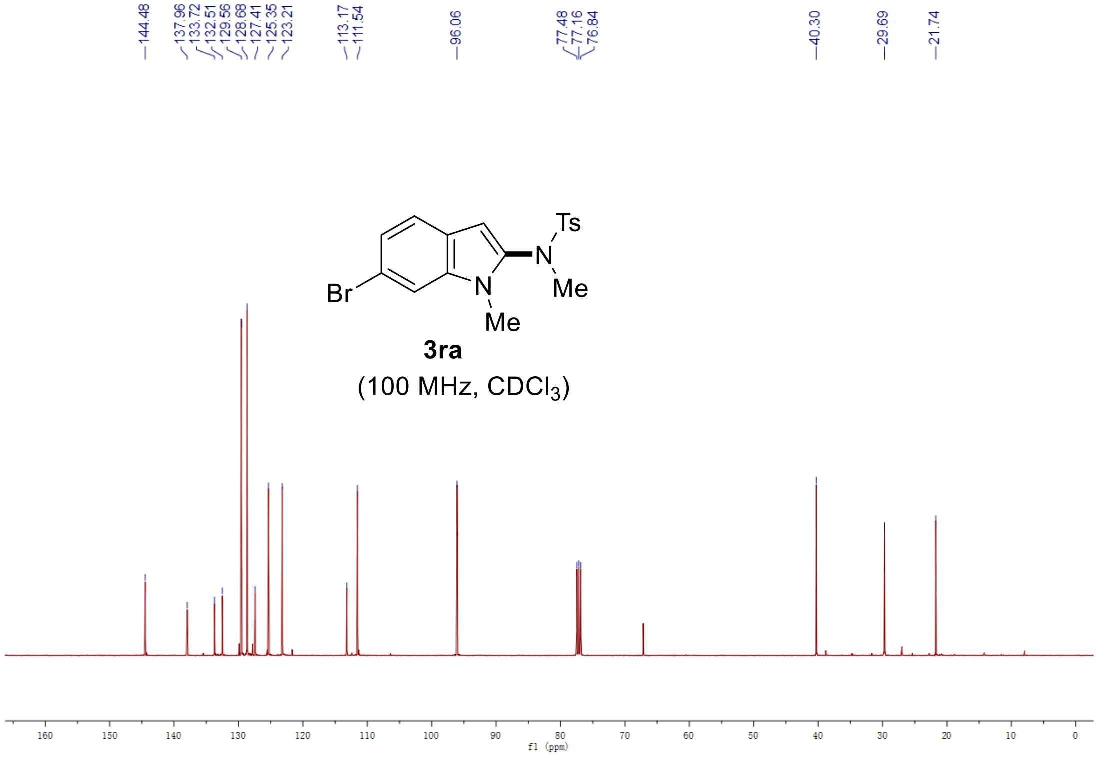
144.48
137.96
133.72
132.51
129.56
128.68
127.41
125.35
123.21
113.17
111.54
96.06
77.48
77.16
76.84
40.30
29.69
21.74
Ts
N
Br
N
Me
Me
3ra
(100 MHz, CDCl3)
f1 (ppm)

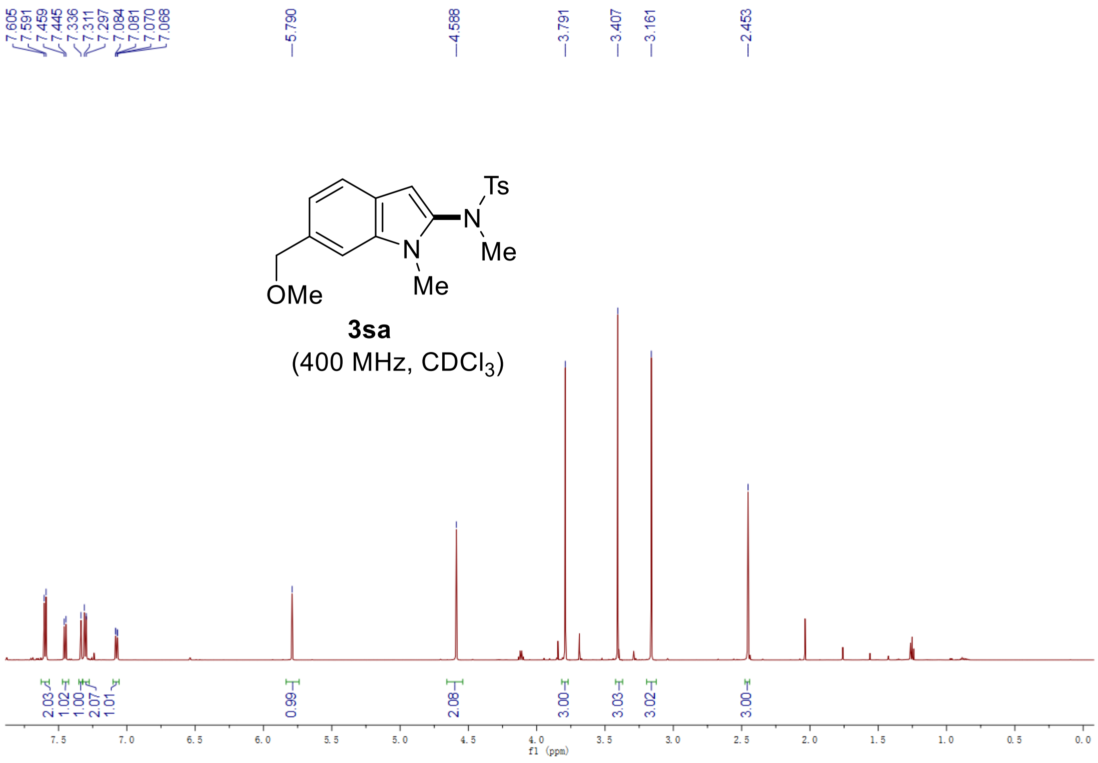
7.605
7.591
7.459
7.445
7.336
7.311
7.297
7.084
7.081
7.070
7.068
5.790
4.588
3.791
3.407
3.161
2.453
Ts
N
Me
N
Me
OMe
3sa
(400 MHz, CDCl$_3$)
2.03
1.02
1.00
2.07
1.01
0.99
2.08
3.00
3.03
3.02
3.00
f1 (ppm)

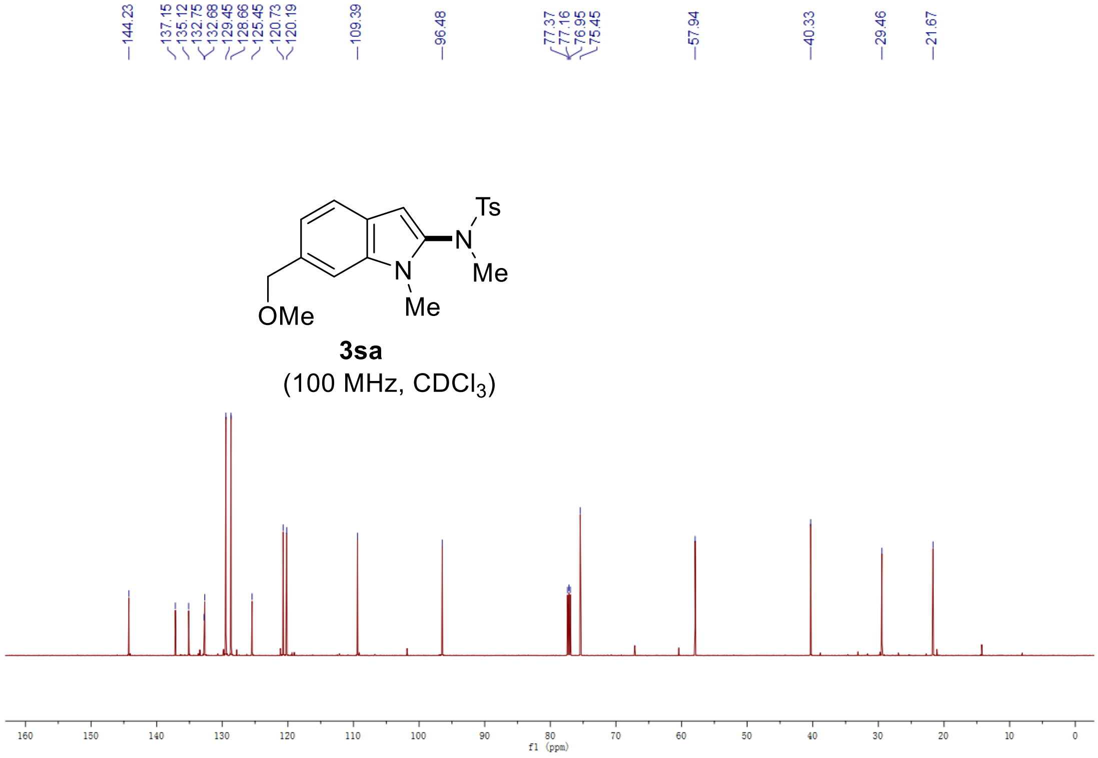
144.23
137.15
135.12
132.75
132.68
129.45
128.66
125.45
120.73
120.19
109.39
96.48
77.37
77.16
76.95
75.45
57.94
40.33
29.46
21.67
Ts
N
Me
N
Me
OMe
3sa
(100 MHz, CDCl$_3$)
f1 (ppm)

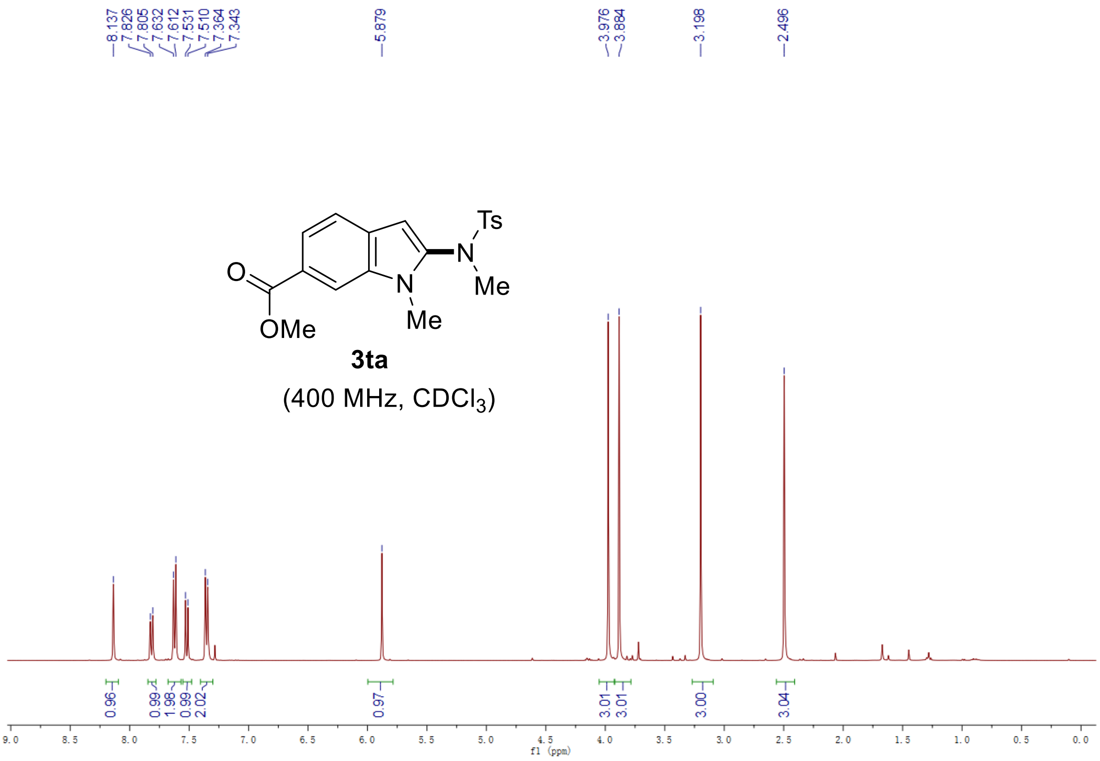
8.137
7.826
7.805
7.632
7.612
7.531
7.510
7.364
7.343
5.879
3.976
3.884
3.198
2.496
Ts
N
Me
O
OMe
N
Me
3ta
(400 MHz, CDCl3)
0.96
0.99
1.98
0.99
2.02
0.97
3.01
3.01
3.00
3.04
f1 (ppm)

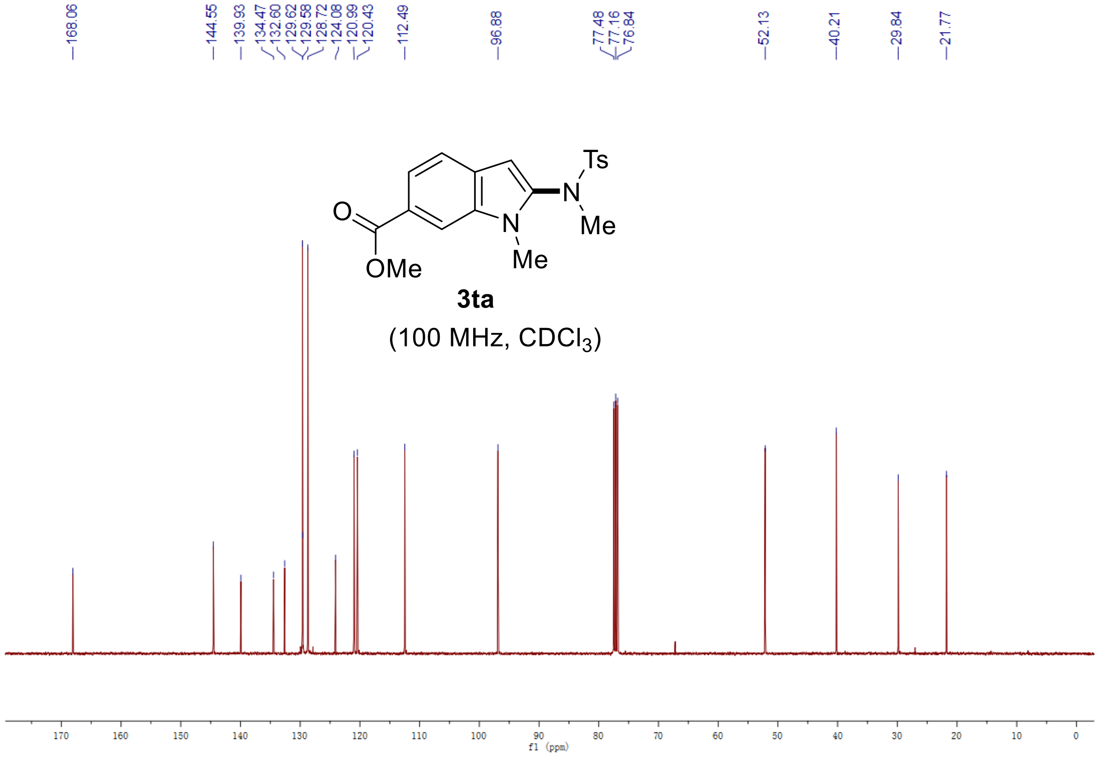
168.06
144.55
139.93
134.47
132.60
129.62
129.58
128.72
124.08
120.99
120.43
112.49
96.88
77.48
77.16
76.84
52.13
40.21
29.84
21.77
Ts
N
Me
O
OMe
N
Me
3ta
(100 MHz, CDCl3)
f1 (ppm)

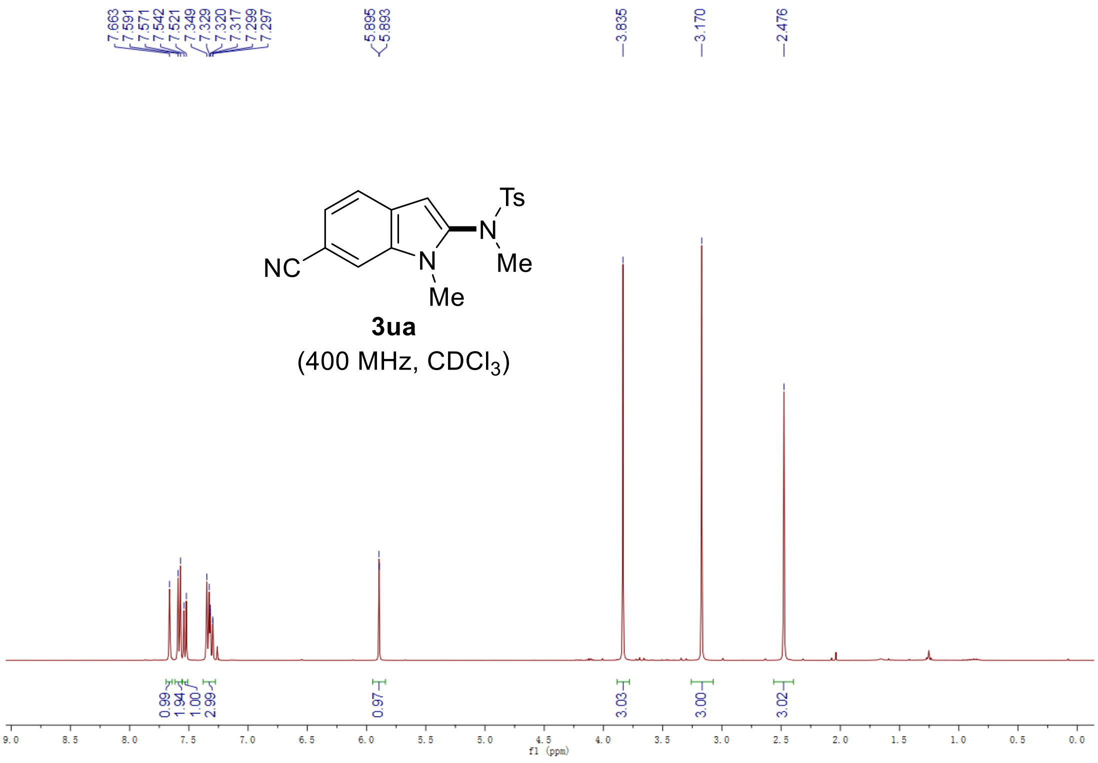
7.663
7.591
7.571
7.542
7.521
7.349
7.329
7.320
7.317
7.299
7.297
5.895
5.893
3.835
3.170
2.476
Ts
N
NC
N
Me
Me
3ua
(400 MHz, CDCl3)
0.99
1.94
1.00
2.99
0.97
3.03
3.00
3.02
f1 (ppm)

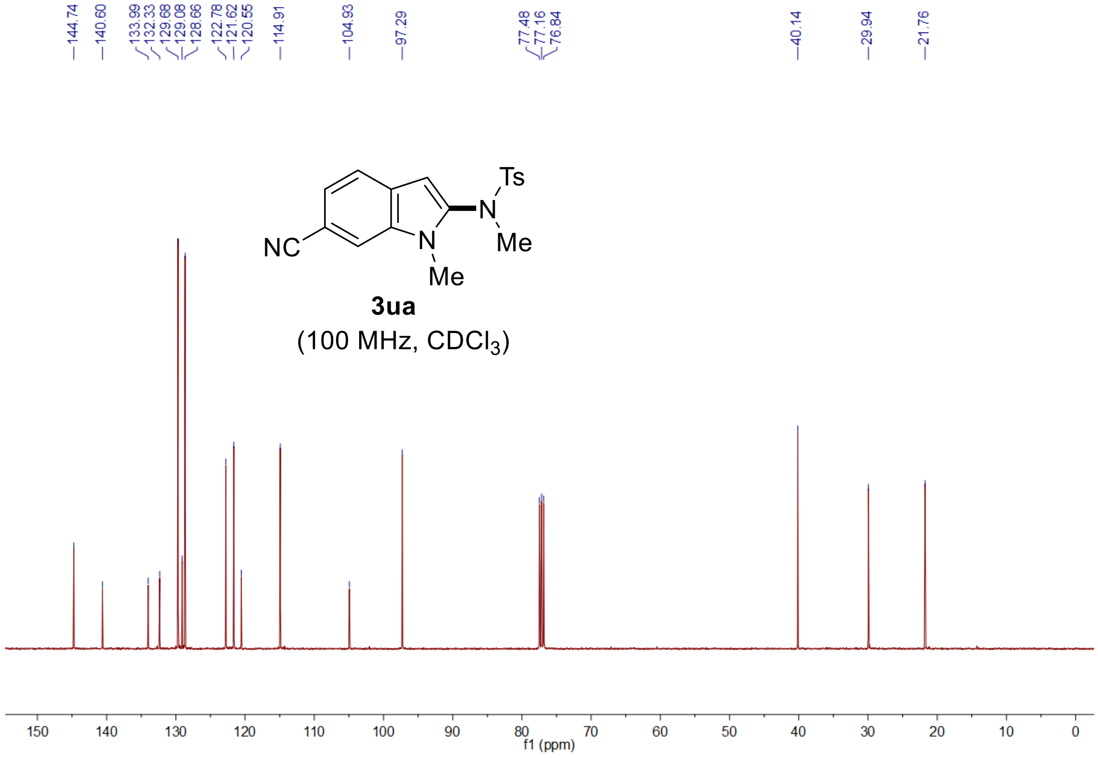
144.74
140.60
133.99
132.33
129.68
129.08
128.66
122.78
121.62
120.55
114.91
104.93
97.29
77.48
77.16
76.84
40.14
29.94
21.76
Ts
N
NC
N
Me
Me
3ua
(100 MHz, CDCl3)
f1 (ppm)

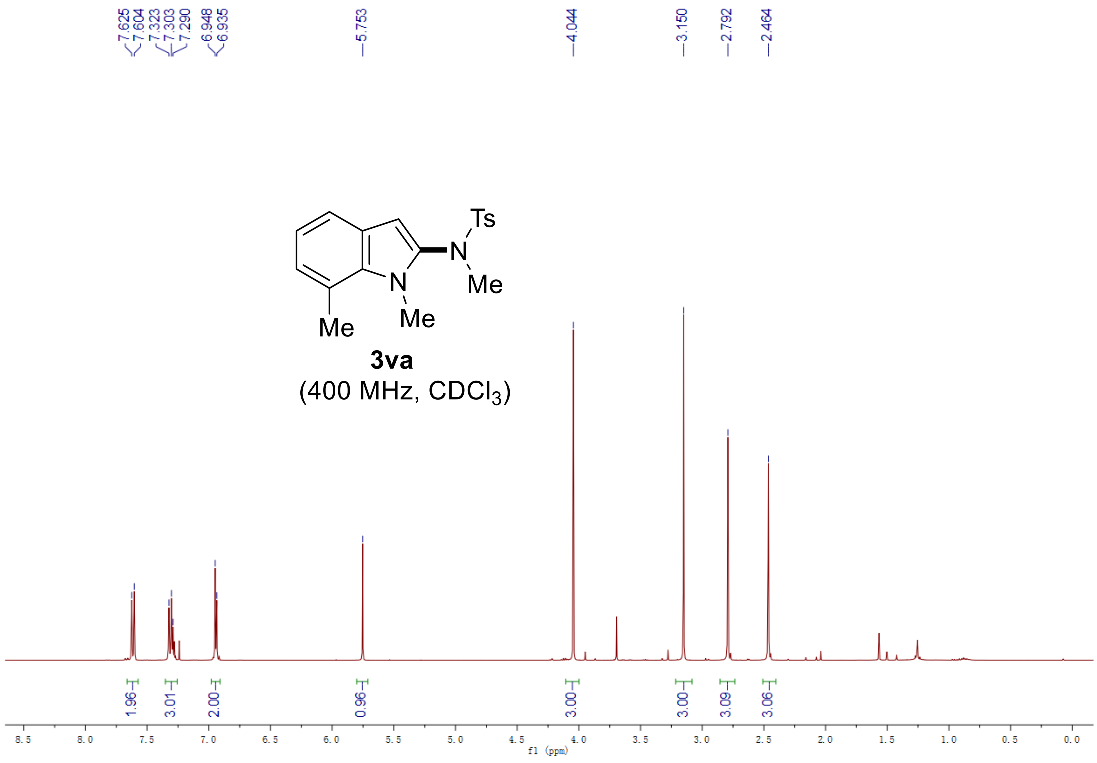
7.625
7.604
7.323
7.303
7.290
6.948
6.935
5.753
4.044
3.150
2.792
2.464
Ts
N
Me
N
Me
Me
3va
(400 MHz, CDCl3)
1.96
3.01
2.00
0.96
3.00
3.00
3.09
3.06
8.5
8.0
7.5
7.0
6.5
6.0
5.5
5.0
4.5
4.0
3.5
3.0
2.5
2.0
1.5
1.0
0.5
0.0
f1 (ppm)

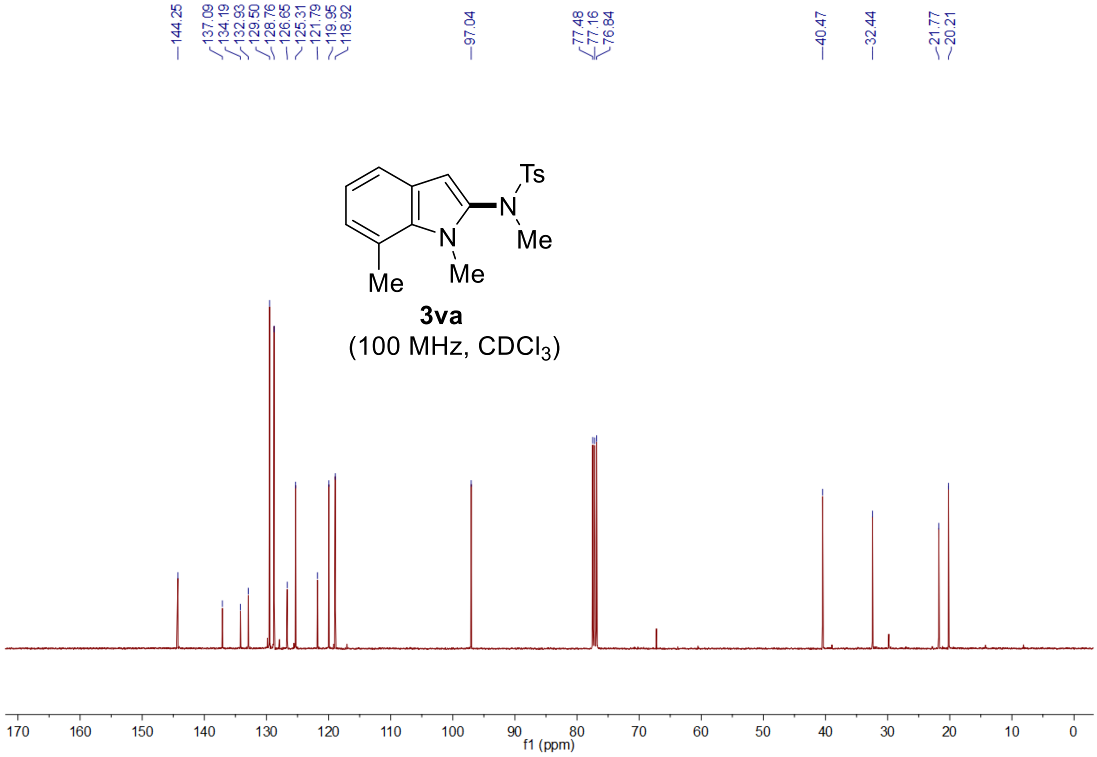
144.25
137.09
134.19
132.93
129.50
128.76
126.65
125.31
121.79
119.95
118.92
97.04
77.48
77.16
76.84
40.47
32.44
21.77
20.21
Ts
N
Me
N
Me
Me
3va
(100 MHz, CDCl3)
170
160
150
140
130
120
110
100
90
80
70
60
50
40
30
20
10
0
f1 (ppm)

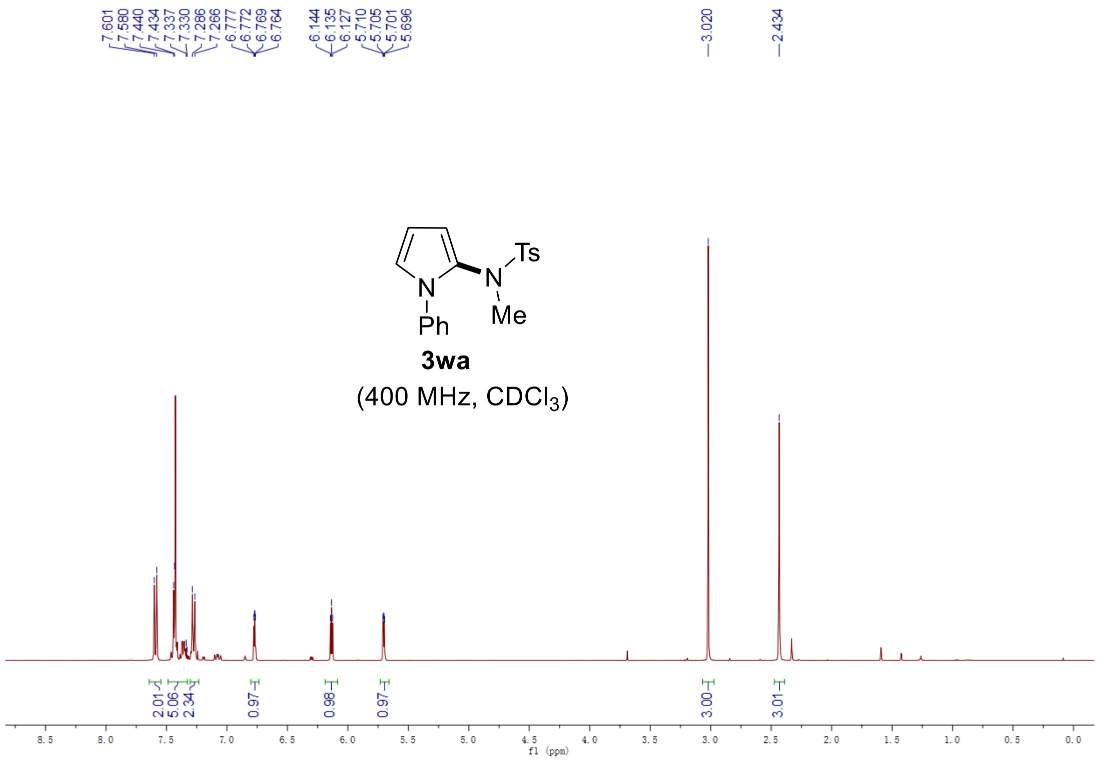

3wa

(400 MHz, $CDCl_3$)

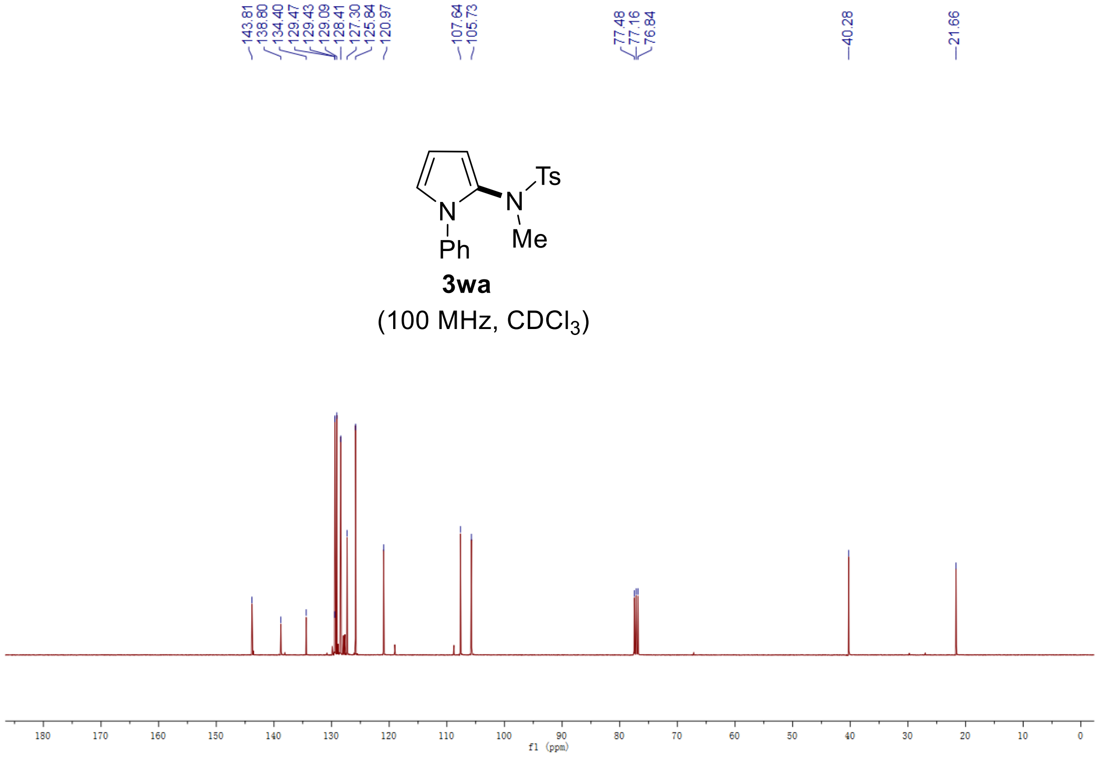

3wa

(100 MHz, $CDCl_3$)

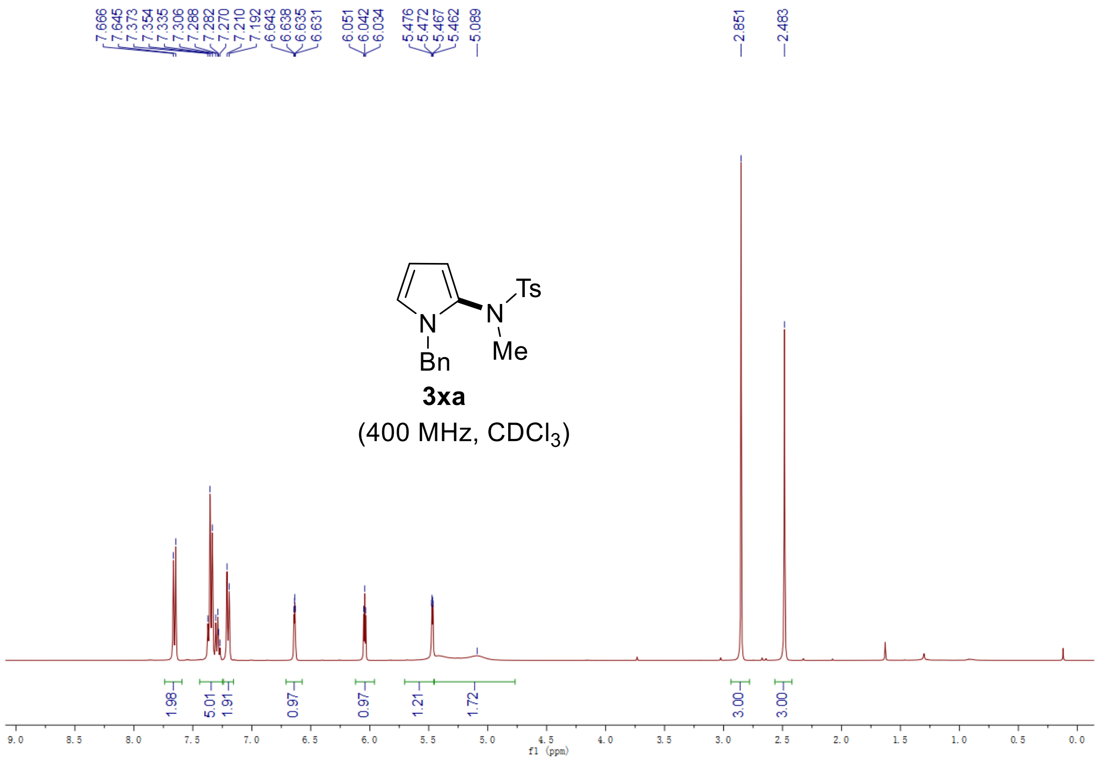
7.666
7.645
7.373
7.354
7.335
7.306
7.288
7.282
7.270
7.210
7.192
6.643
6.638
6.635
6.631
6.051
6.042
6.034
5.476
5.472
5.467
5.462
5.089
2.851
2.483
Ts
N
N
Bn
Me
3xa
(400 MHz, CDCl3)
1.98
5.01
1.91
0.97
0.97
1.21
1.72
3.00
3.00
f1 (ppm)

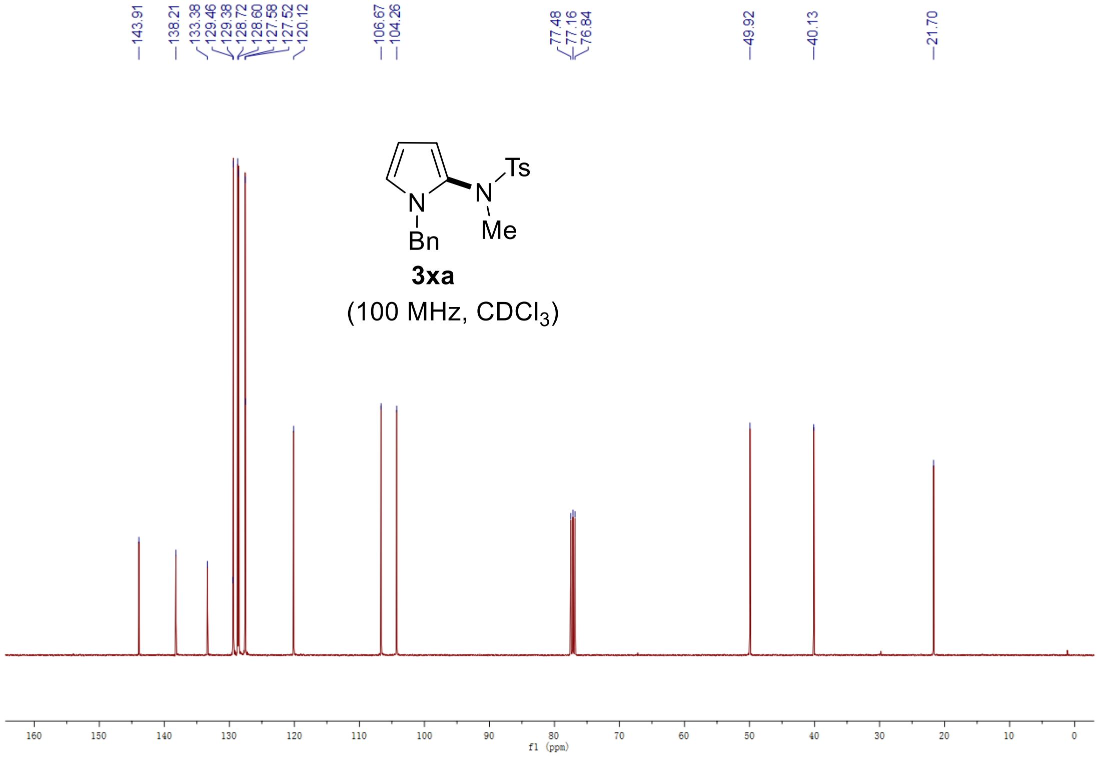
143.91
138.21
133.38
129.46
129.38
128.72
128.60
127.58
127.52
120.12
106.67
104.26
77.48
77.16
76.84
49.92
40.13
21.70
Ts
N
N
Bn
Me
3xa
(100 MHz, CDCl3)
f1 (ppm)

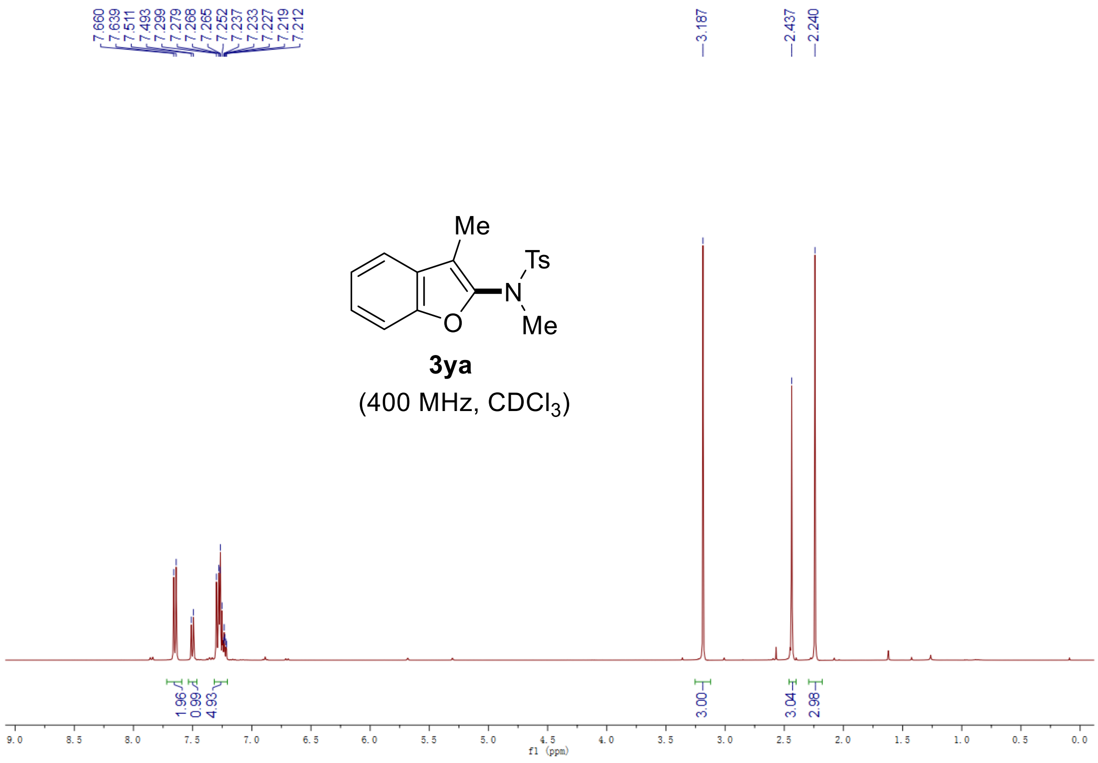
7.660
7.639
7.511
7.493
7.299
7.279
7.268
7.265
7.252
7.237
7.233
7.227
7.219
7.212
3.187
2.437
2.240
Me
Ts
N
O
Me
3ya
(400 MHz, CDCl3)
1.96
0.99
4.93
3.00
3.04
2.98
f1 (ppm)

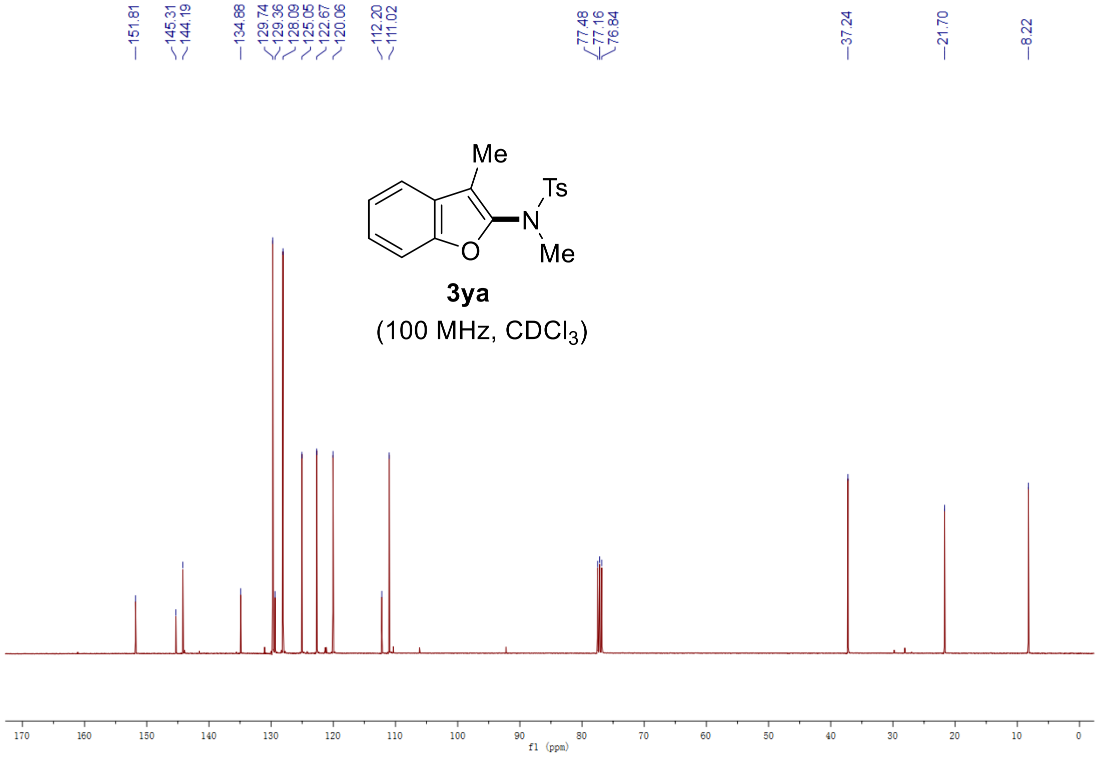
151.81
145.31
144.19
134.88
129.74
129.36
128.09
125.05
122.67
120.06
112.20
111.02
77.48
77.16
76.84
37.24
21.70
8.22
Me
Ts
N
O
Me
3ya
(100 MHz, CDCl3)
f1 (ppm)

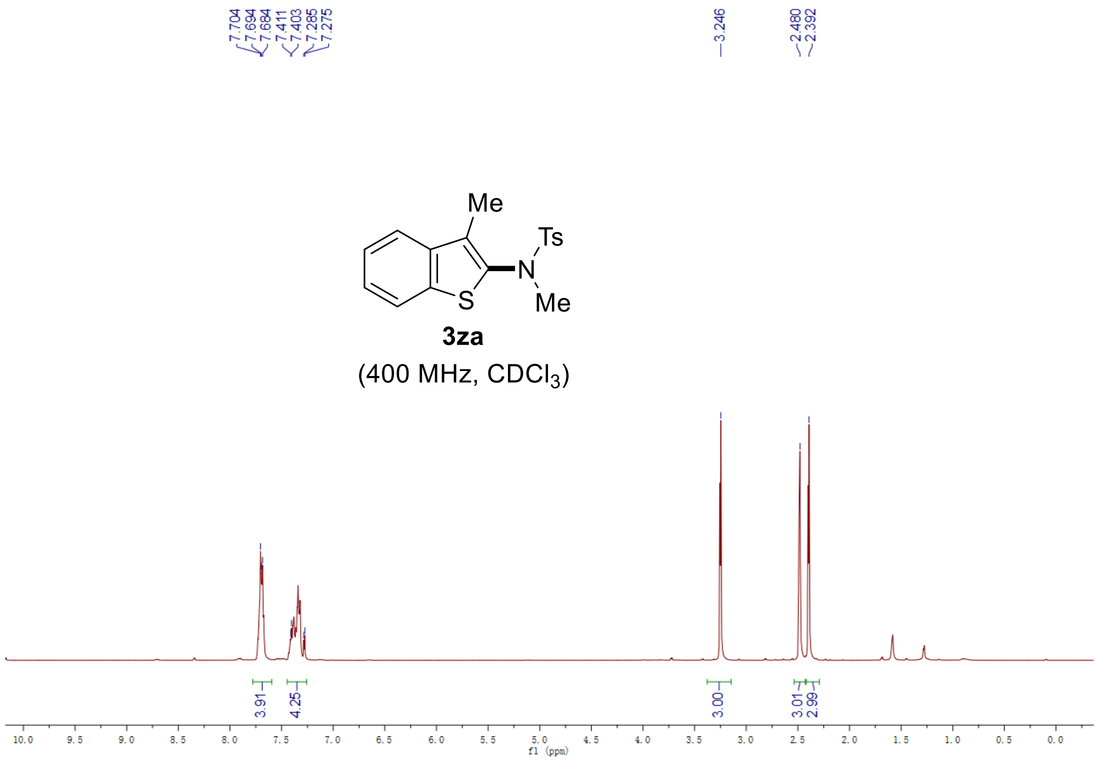
7.704
7.694
7.684
7.411
7.403
7.285
7.275
3.246
2.480
2.392
Me
Ts
N
S
Me
3za
(400 MHz, CDCl$_3$)
3.91
4.25
3.00
3.01
2.99
10.0 9.5 9.0 8.5 8.0 7.5 7.0 6.5 6.0 5.5 5.0 4.5 4.0 3.5 3.0 2.5 2.0 1.5 1.0 0.5 0.0
f1 (ppm)

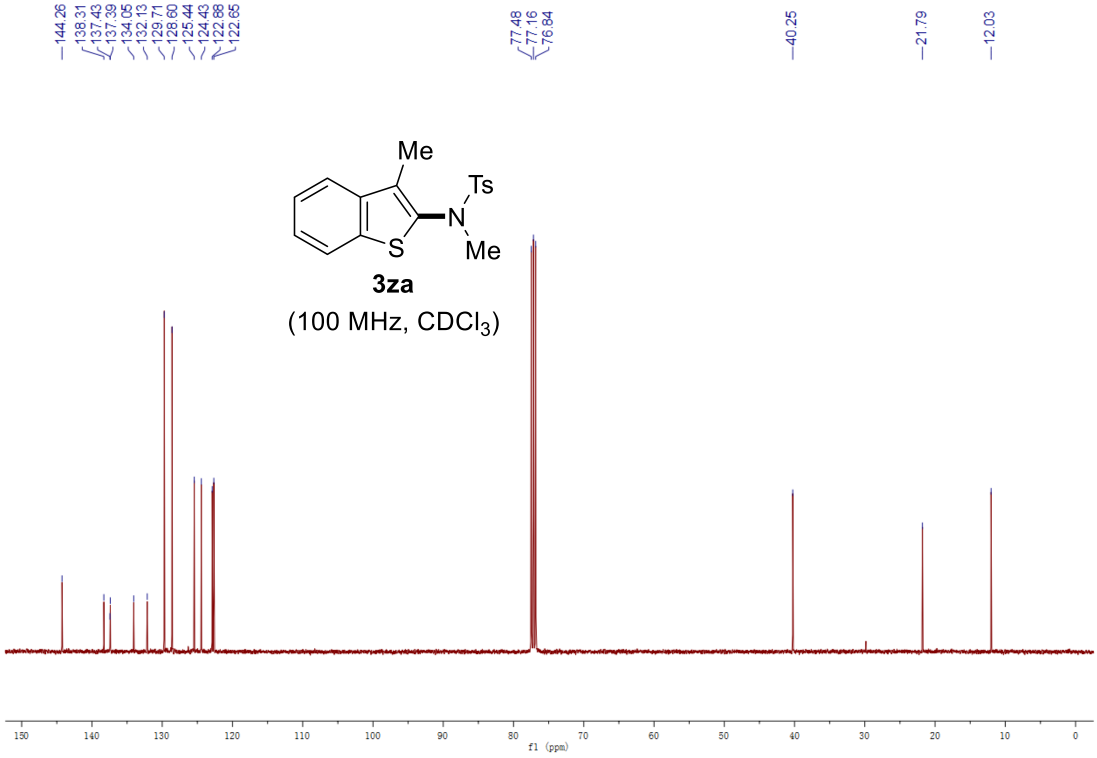
144.26
138.31
137.43
137.39
134.05
132.13
129.71
128.60
125.44
124.43
122.88
122.65
77.48
77.16
76.84
40.25
21.79
12.03
Me
Ts
N
S
Me
3za
(100 MHz, CDCl$_3$)
150 140 130 120 110 100 90 80 70 60 50 40 30 20 10 0
f1 (ppm)

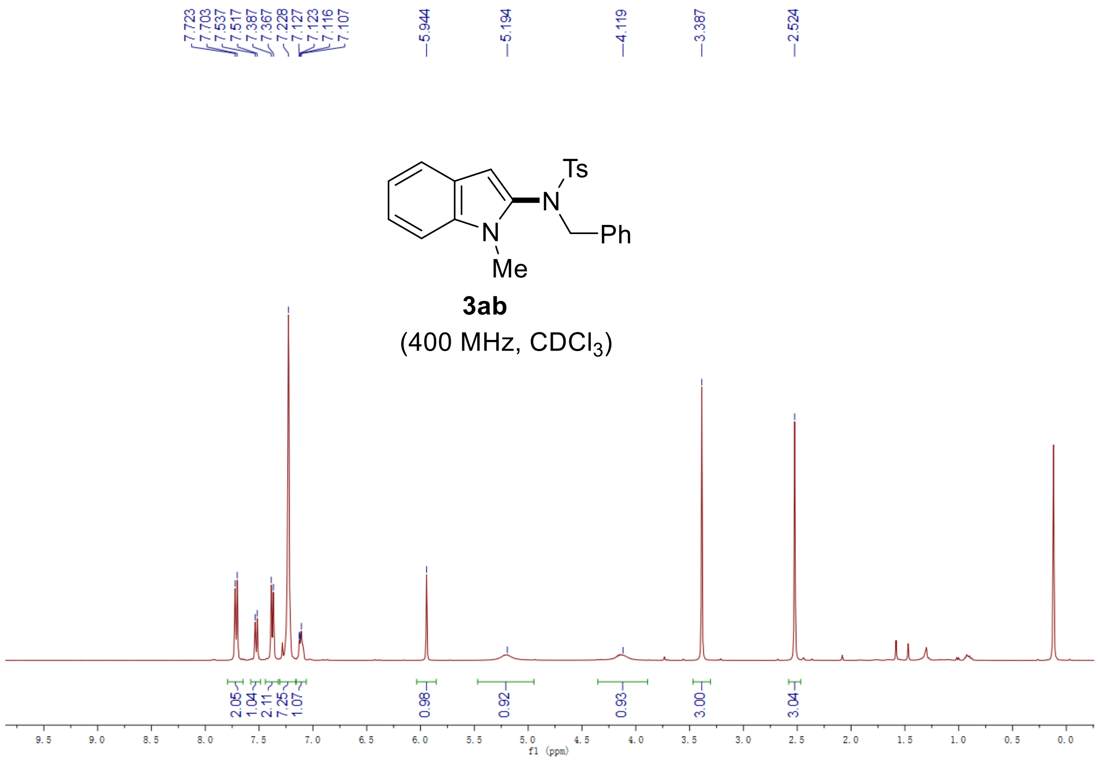
7.723
7.703
7.537
7.517
7.387
7.367
7.228
7.127
7.123
7.116
7.107
5.944
5.194
4.119
3.387
2.524
Ts
N
Ph
N
Me
3ab
(400 MHz, CDCl3)
2.05
1.04
2.11
7.25
1.07
0.98
0.92
0.93
3.00
3.04
f1 (ppm)

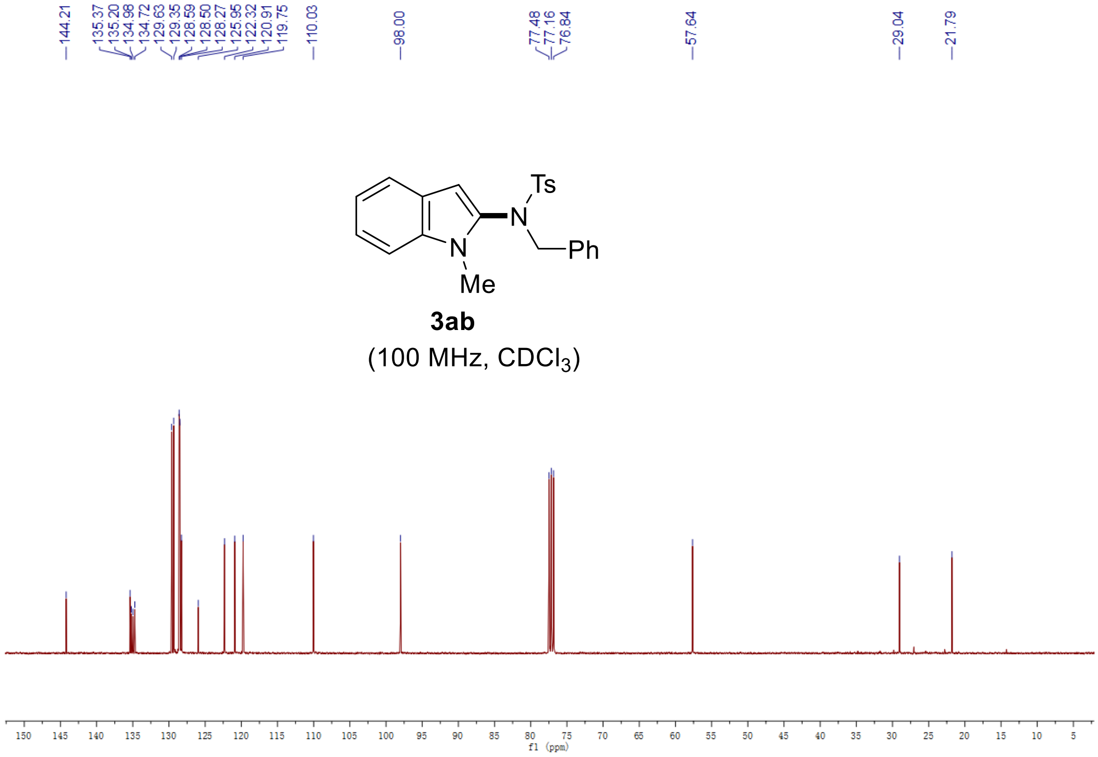
144.21
135.37
135.20
134.98
134.72
129.63
129.35
128.59
128.50
128.27
125.95
122.32
120.91
119.75
110.03
98.00
77.48
77.16
76.84
57.64
29.04
21.79
Ts
N
Ph
N
Me
3ab
(100 MHz, CDCl3)
f1 (ppm)

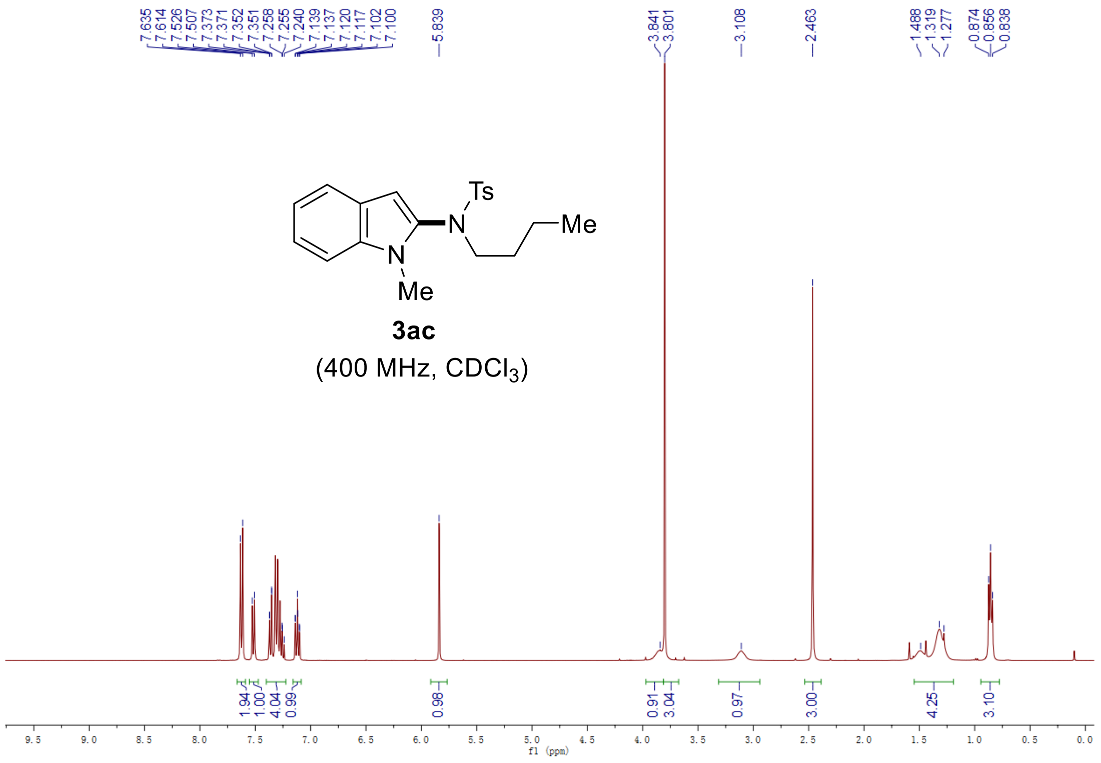
7.635
7.614
7.526
7.507
7.373
7.371
7.352
7.351
7.258
7.255
7.240
7.139
7.137
7.120
7.117
7.102
7.100
5.839
3.841
3.801
3.108
2.463
1.488
1.319
1.277
0.874
0.856
0.838
Ts
N
Me
N
Me
3ac
(400 MHz, CDCl3)
1.94
1.00
4.04
0.99
0.98
0.91
3.04
0.97
3.00
4.25
3.10
f1 (ppm)

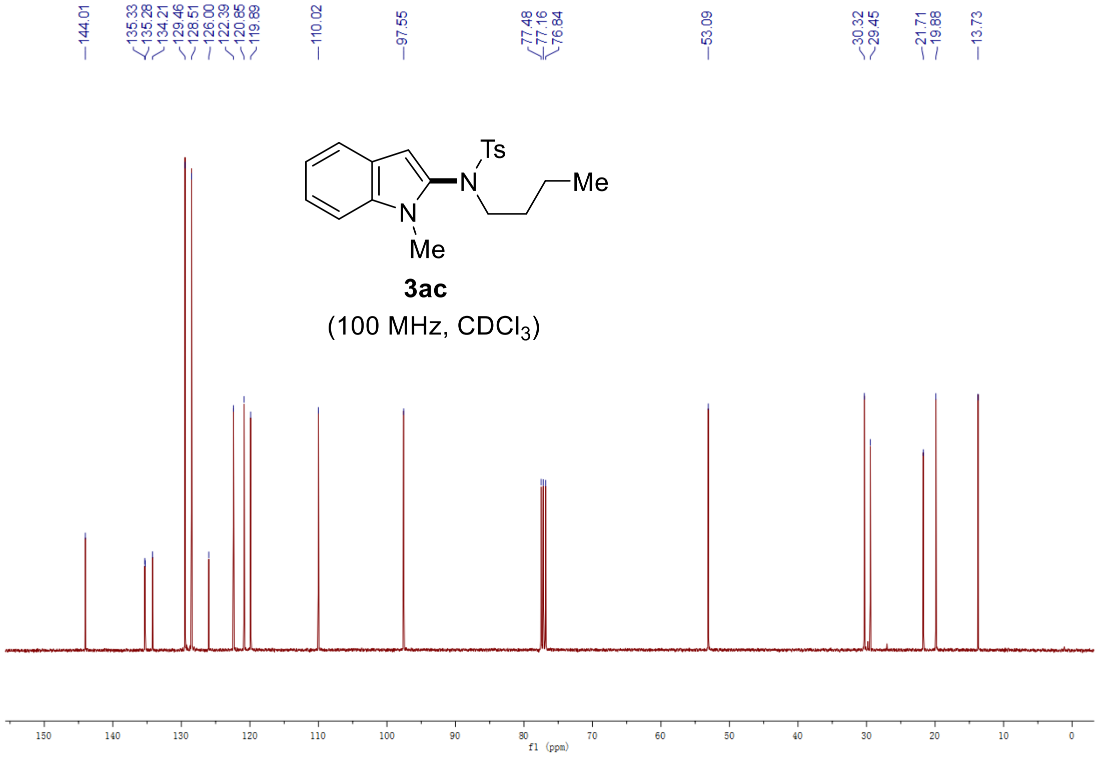
144.01
135.33
135.28
134.21
129.46
128.51
126.00
122.39
120.85
119.89
110.02
97.55
77.48
77.16
76.84
53.09
30.32
29.45
21.71
19.88
13.73
Ts
N
Me
N
Me
3ac
(100 MHz, CDCl3)
f1 (ppm)

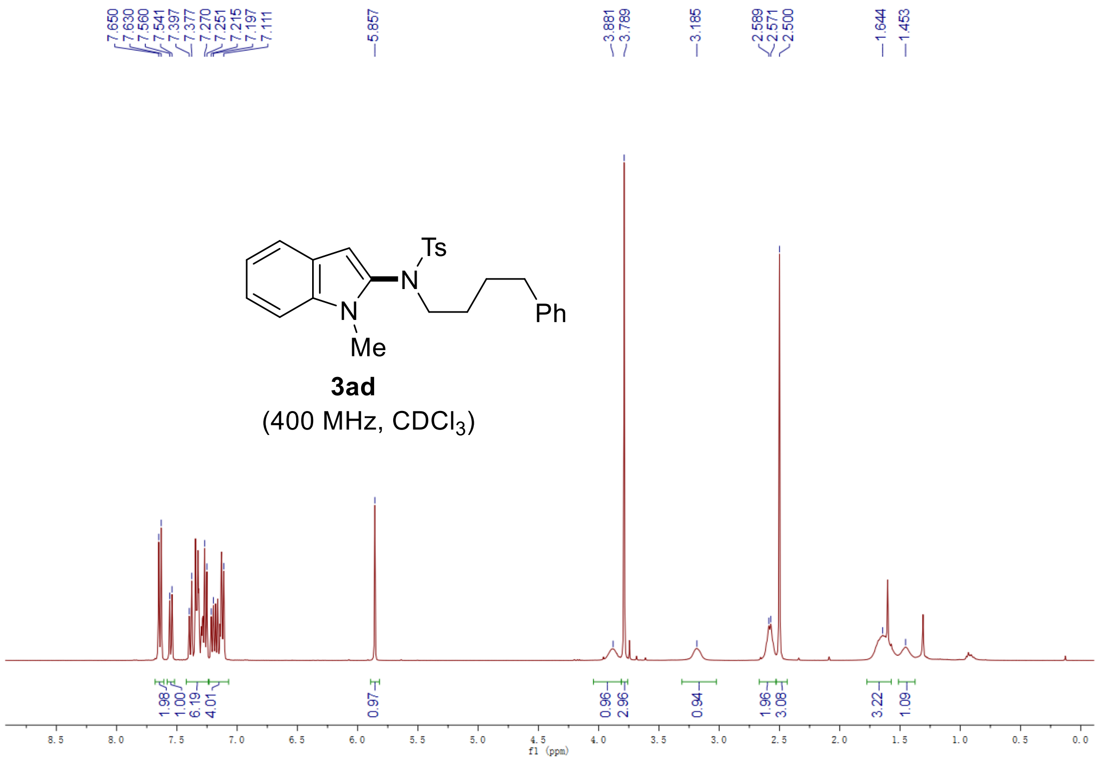
7.650
7.630
7.560
7.541
7.397
7.377
7.270
7.251
7.215
7.197
7.111
5.857
3.881
3.789
3.185
2.589
2.571
2.500
1.644
1.453
Ts
N
N
Me
Ph
3ad
(400 MHz, CDCl3)
1.98
1.00
6.19
4.01
0.97
0.96
2.96
0.94
1.96
3.08
3.22
1.09
f1 (ppm)

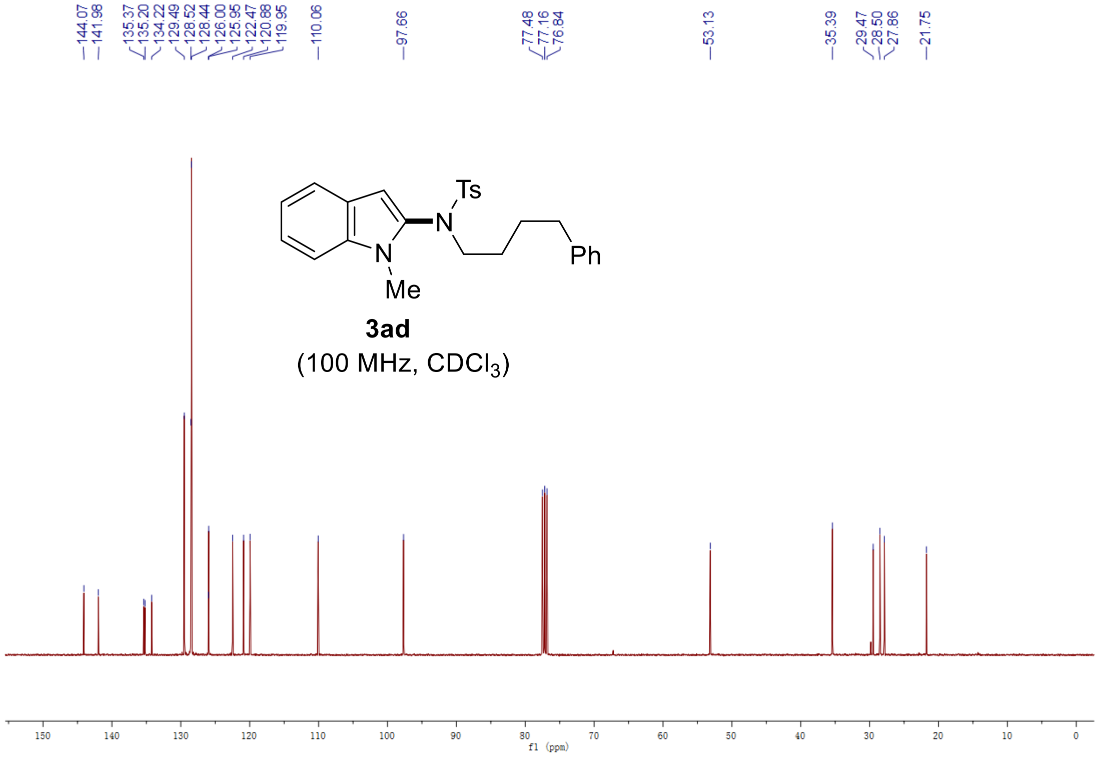
144.07
141.98
135.37
135.20
134.22
129.49
128.52
128.44
126.00
125.95
122.47
120.88
119.95
110.06
97.66
77.48
77.16
76.84
53.13
35.39
29.47
28.50
27.86
21.75
Ts
N
N
Me
Ph
3ad
(100 MHz, CDCl3)
f1 (ppm)

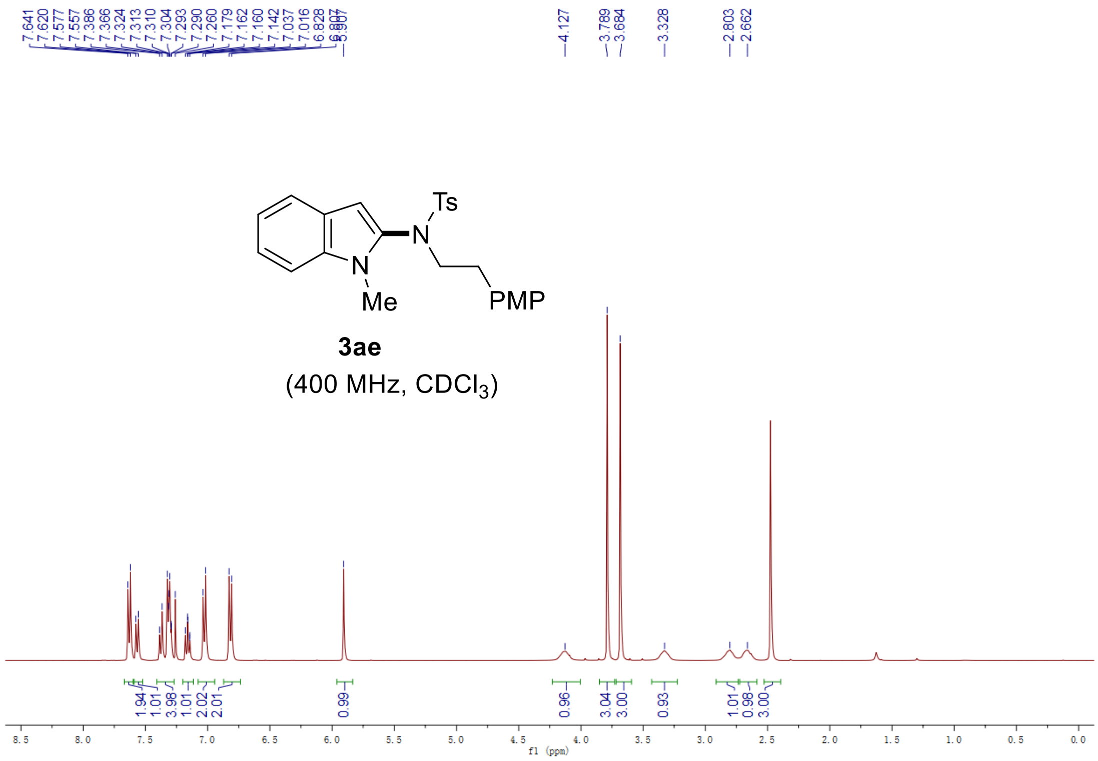

3ae
(400 MHz, CDCl3)

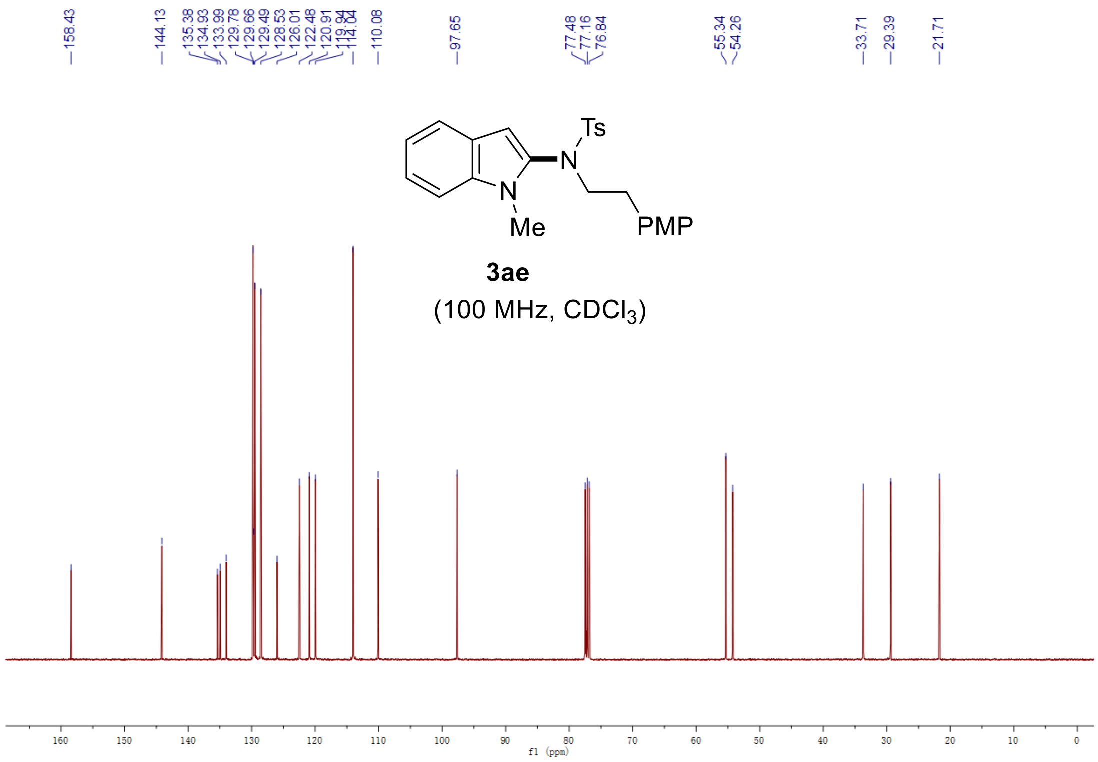

3ae
(100 MHz, CDCl3)

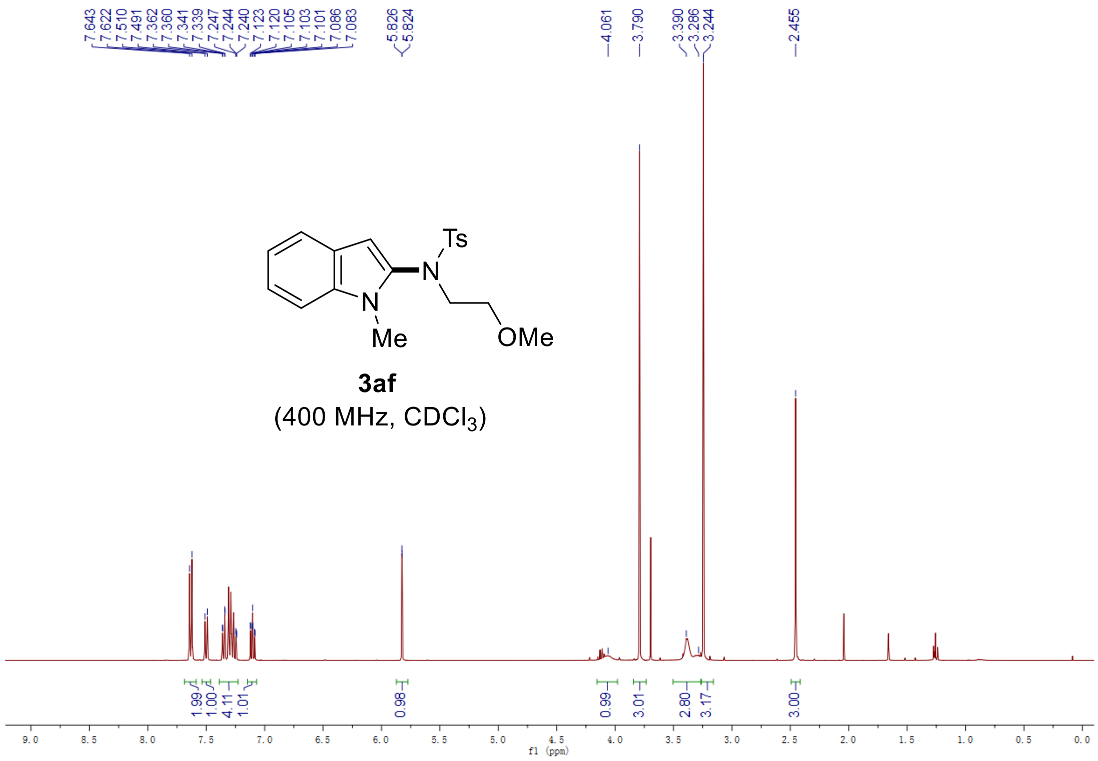

Ts
N
N
Me
OMe
3af
(400 MHz, CDCl3)
f1 (ppm)

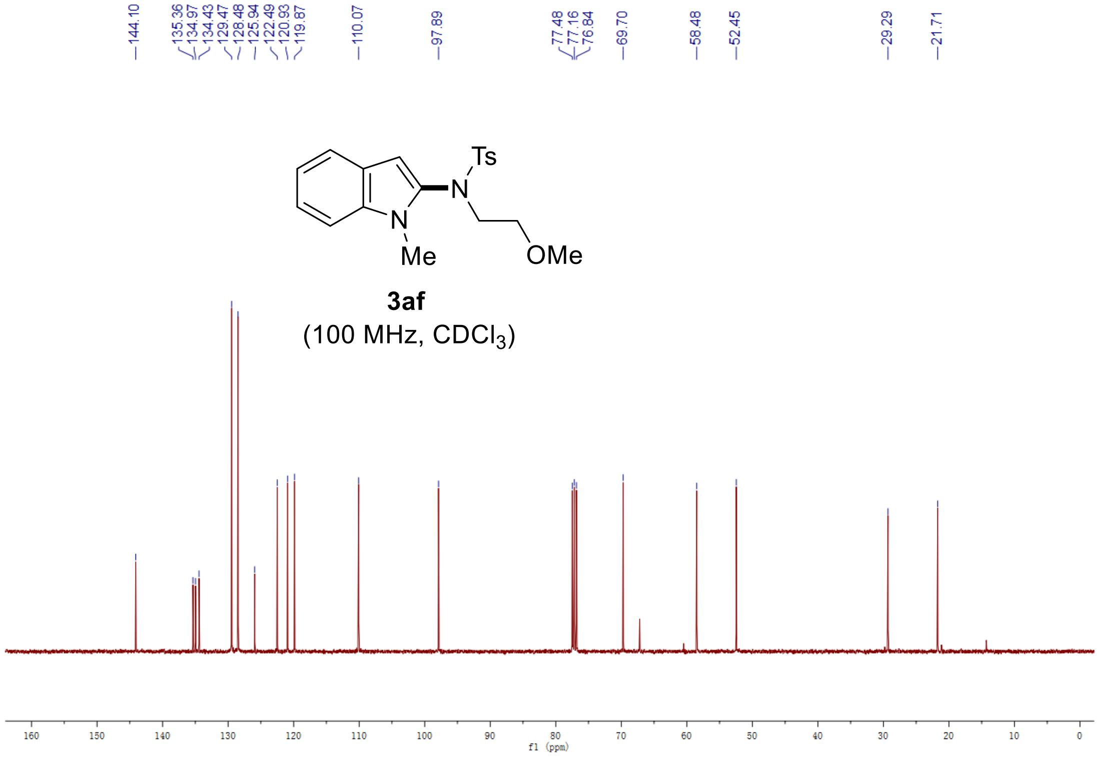

Ts
N
N
Me
OMe
3af
(100 MHz, CDCl3)
f1 (ppm)

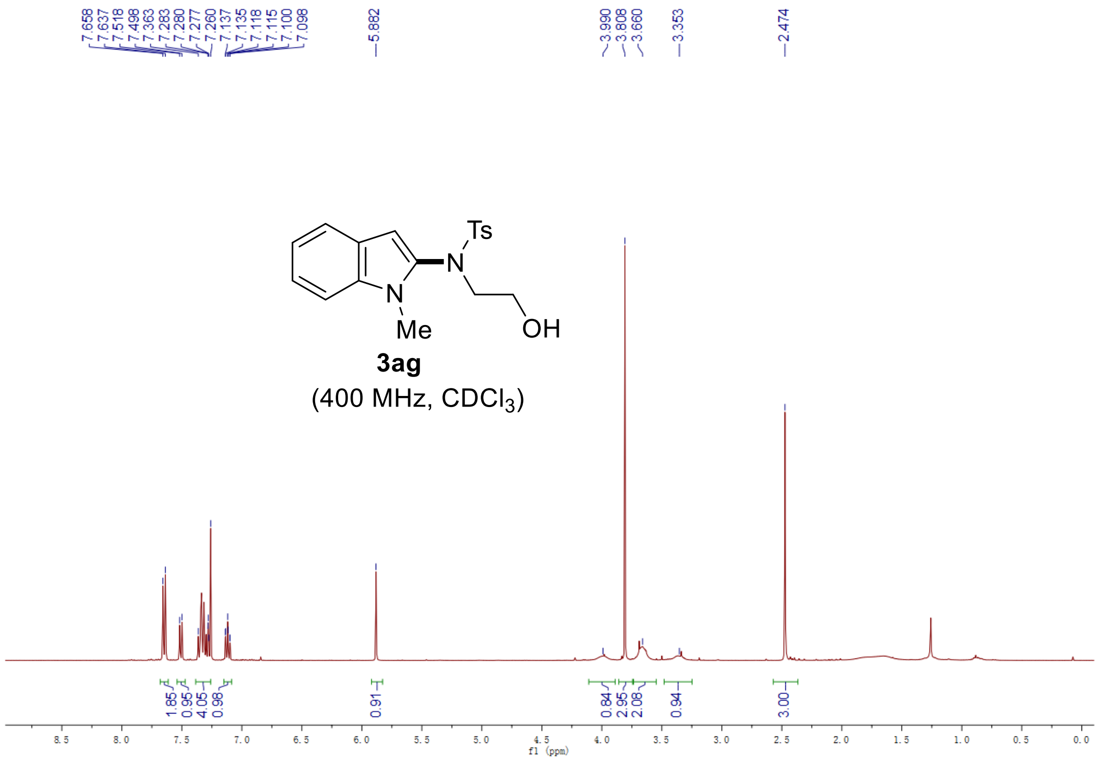
7.658
7.637
7.518
7.498
7.363
7.283
7.280
7.277
7.260
7.137
7.135
7.118
7.115
7.100
7.098
5.882
3.990
3.808
3.660
3.353
2.474
Ts
N
N
Me
OH
3ag
(400 MHz, CDCl3)
1.85
0.95
4.05
0.98
0.91
0.84
2.95
2.08
0.94
3.00
f1 (ppm)

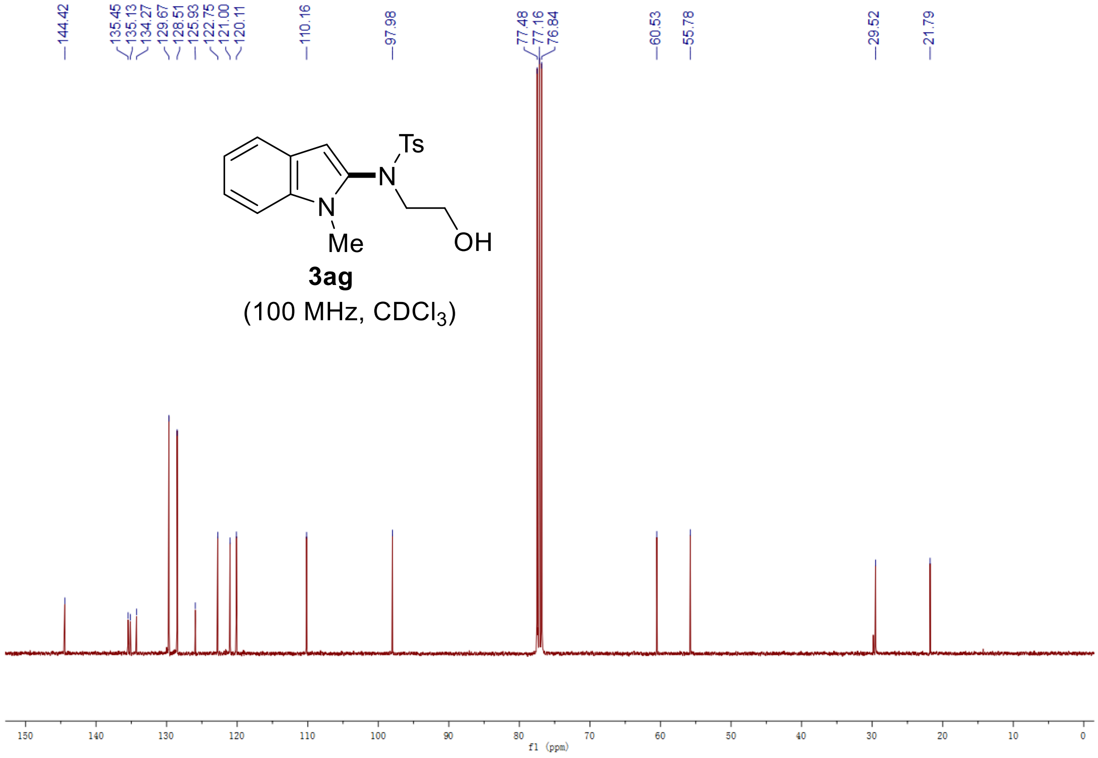
144.42
135.45
135.13
134.27
129.67
128.51
125.93
122.75
121.00
120.11
110.16
97.98
77.48
77.16
76.84
60.53
55.78
29.52
21.79
Ts
N
N
Me
OH
3ag
(100 MHz, CDCl3)
f1 (ppm)

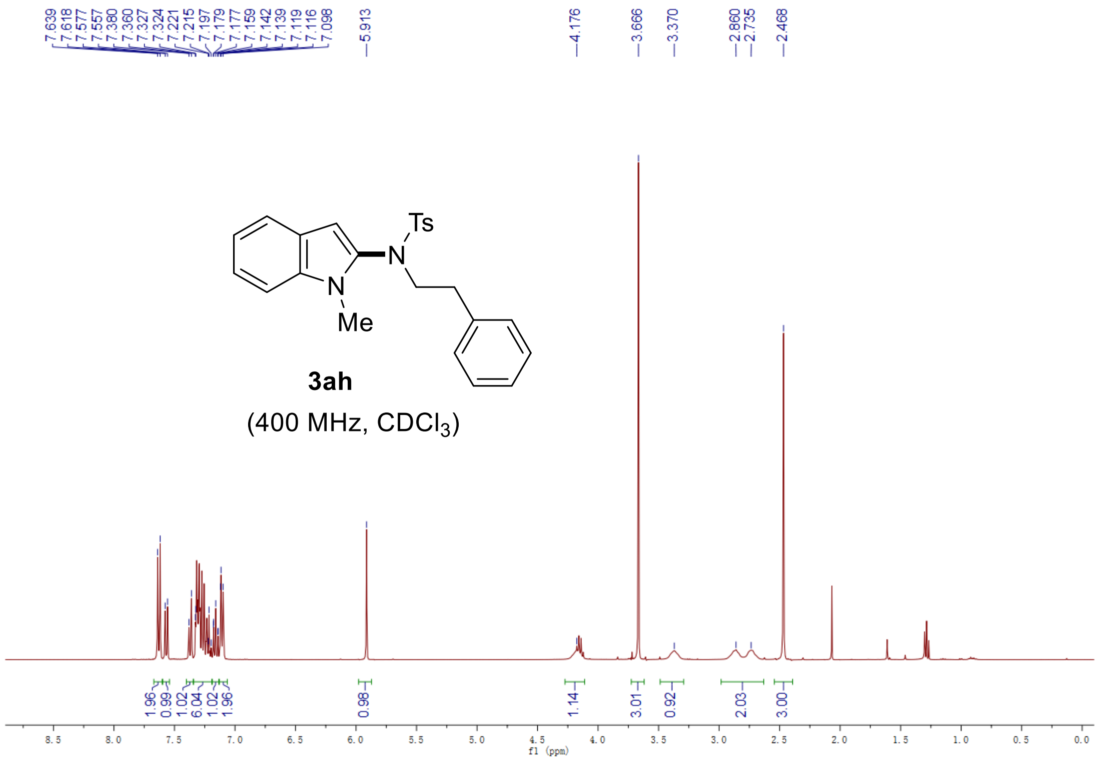
7.639
7.618
7.577
7.557
7.380
7.360
7.327
7.324
7.221
7.215
7.197
7.179
7.177
7.159
7.142
7.139
7.119
7.116
7.098
5.913
4.176
3.666
3.370
2.860
2.735
2.468
Ts
N
N
Me
3ah
(400 MHz, CDCl3)
1.96
0.99
1.02
6.04
1.02
1.96
0.98
1.14
3.01
0.92
2.03
3.00
f1 (ppm)

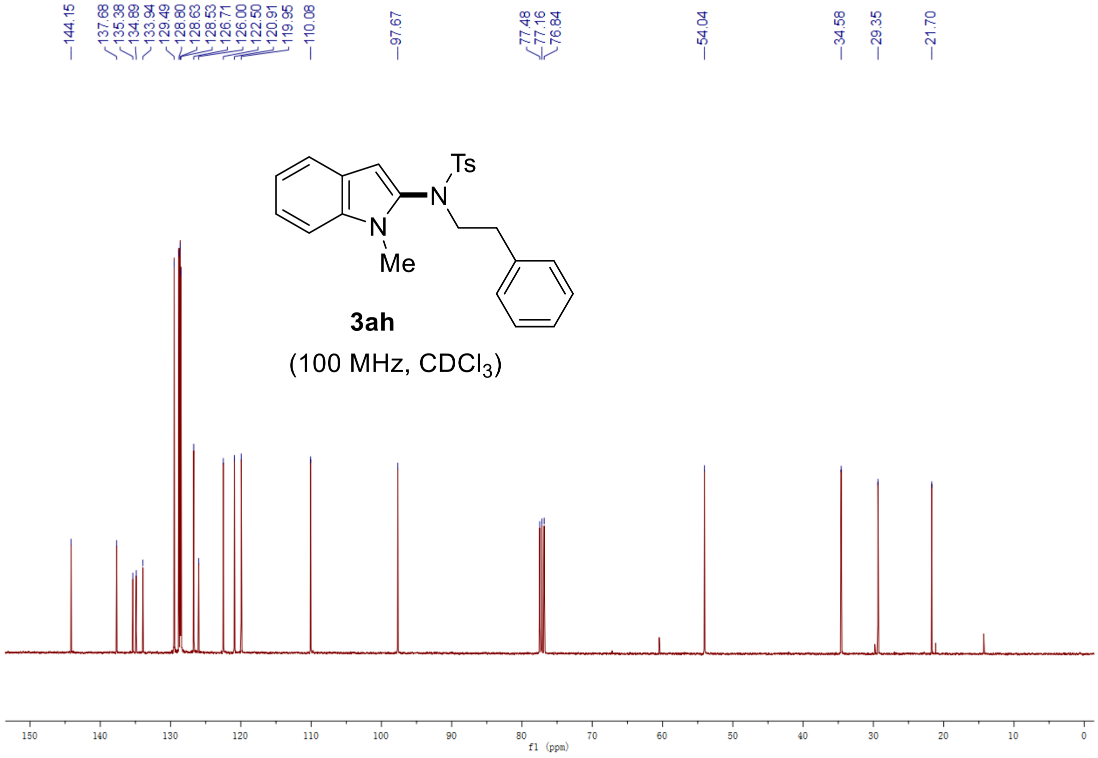
144.15
137.68
135.38
134.89
133.94
129.49
128.80
128.63
128.53
126.71
126.00
122.50
120.91
119.95
110.08
97.67
77.48
77.16
76.84
54.04
34.58
29.35
21.70
Ts
N
N
Me
3ah
(100 MHz, CDCl3)
f1 (ppm)

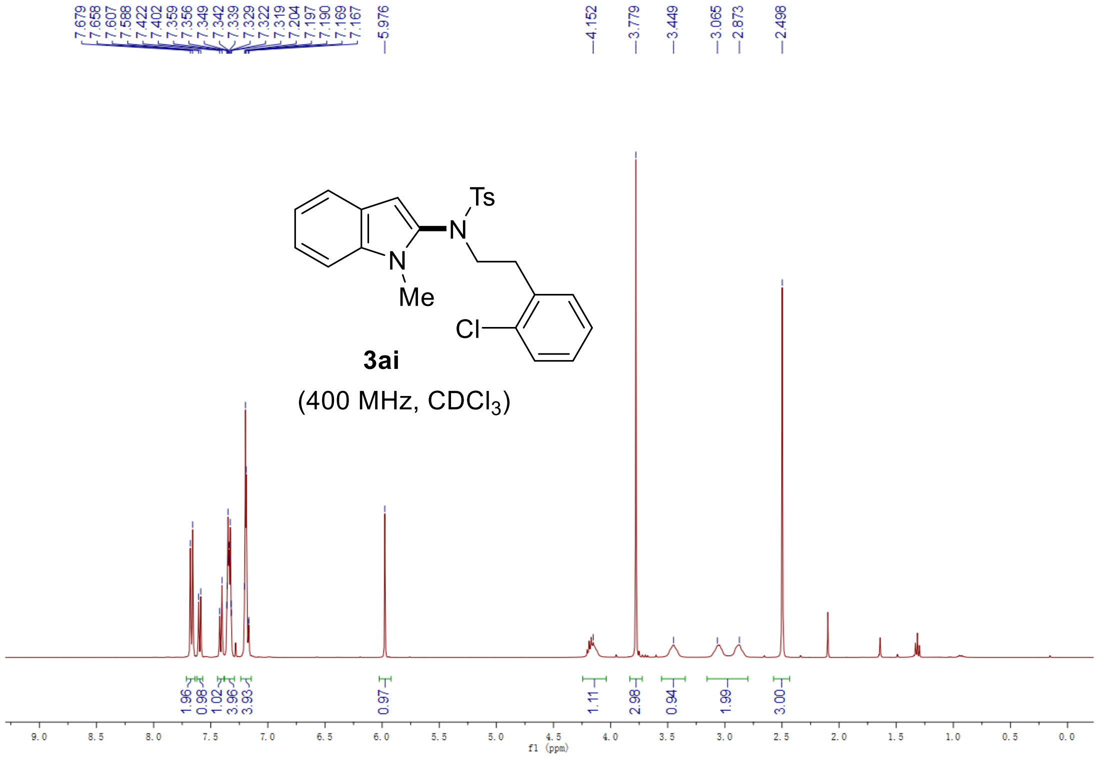

3ai

(400 MHz, $CDCl_3$)

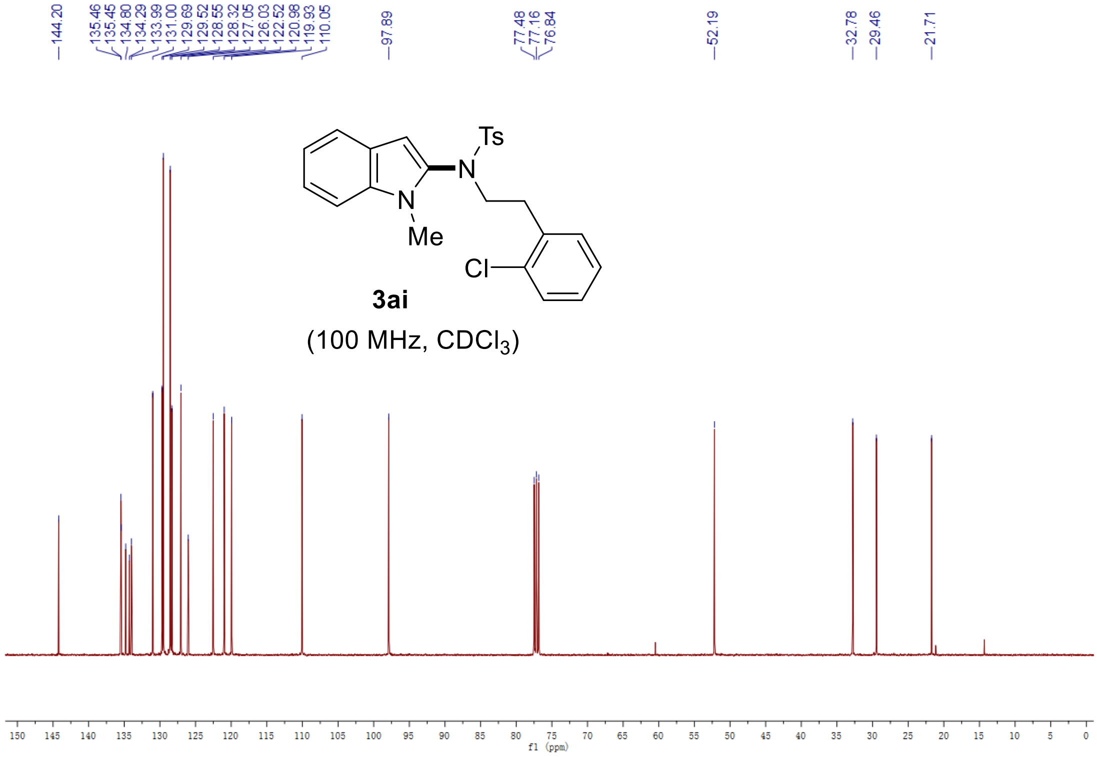

3ai

(100 MHz, $CDCl_3$)

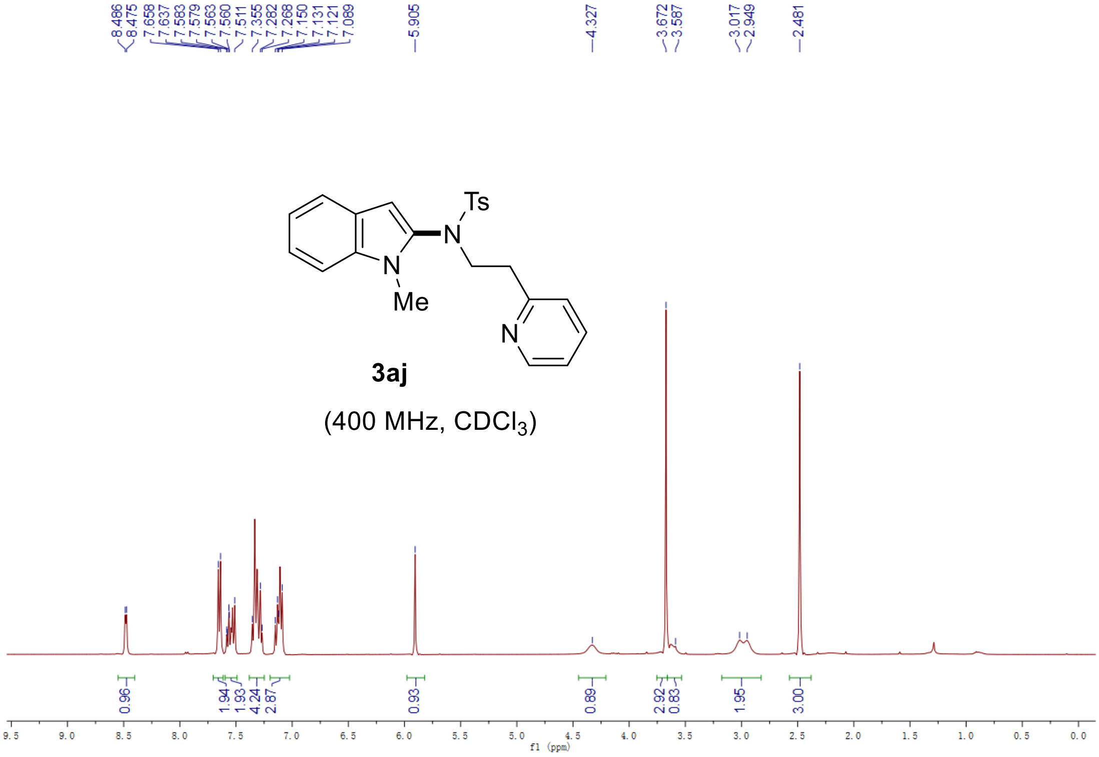
3aj
(400 MHz, CDCl3)
8.486
8.475
7.658
7.637
7.583
7.579
7.563
7.560
7.511
7.355
7.282
7.268
7.150
7.131
7.121
7.089
5.905
4.327
3.672
3.587
3.017
2.949
2.481
0.96
1.94
1.93
4.24
2.87
0.93
0.89
2.92
0.83
1.95
3.00
f1 (ppm)

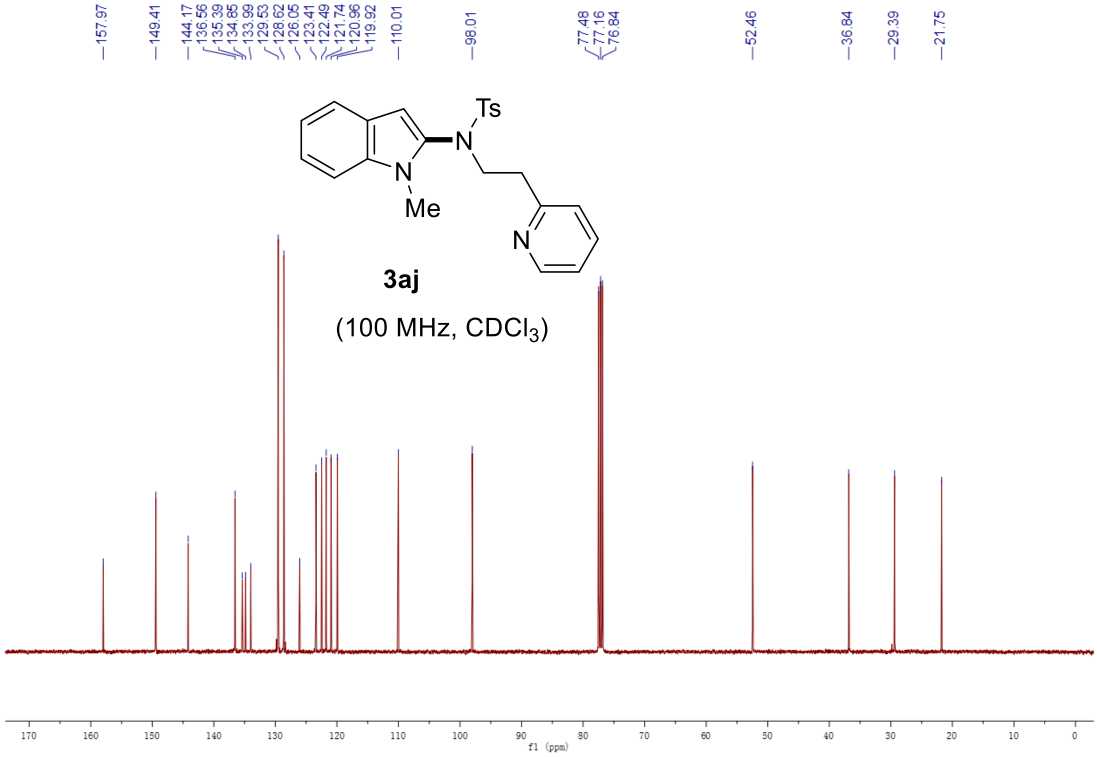
3aj
(100 MHz, CDCl3)
157.97
149.41
144.17
136.56
135.39
134.85
133.99
129.53
128.62
126.05
123.41
122.49
121.74
120.96
119.92
110.01
98.01
77.48
77.16
76.84
52.46
36.84
29.39
21.75
f1 (ppm)

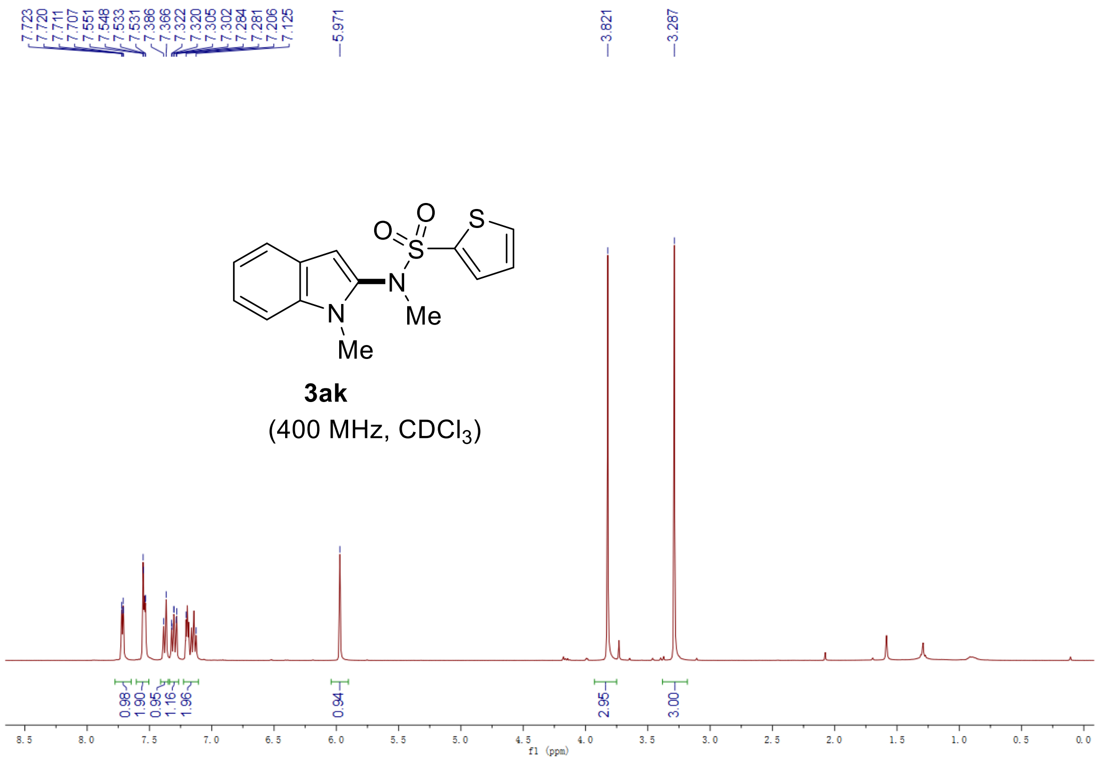
7.723
7.720
7.711
7.707
7.551
7.548
7.533
7.531
7.386
7.366
7.322
7.320
7.305
7.302
7.284
7.281
7.206
7.125
5.971
3.821
3.287
3ak
(400 MHz, CDCl3)
0.98
1.90
0.95
1.16
1.96
0.94
2.95
3.00
f1 (ppm)

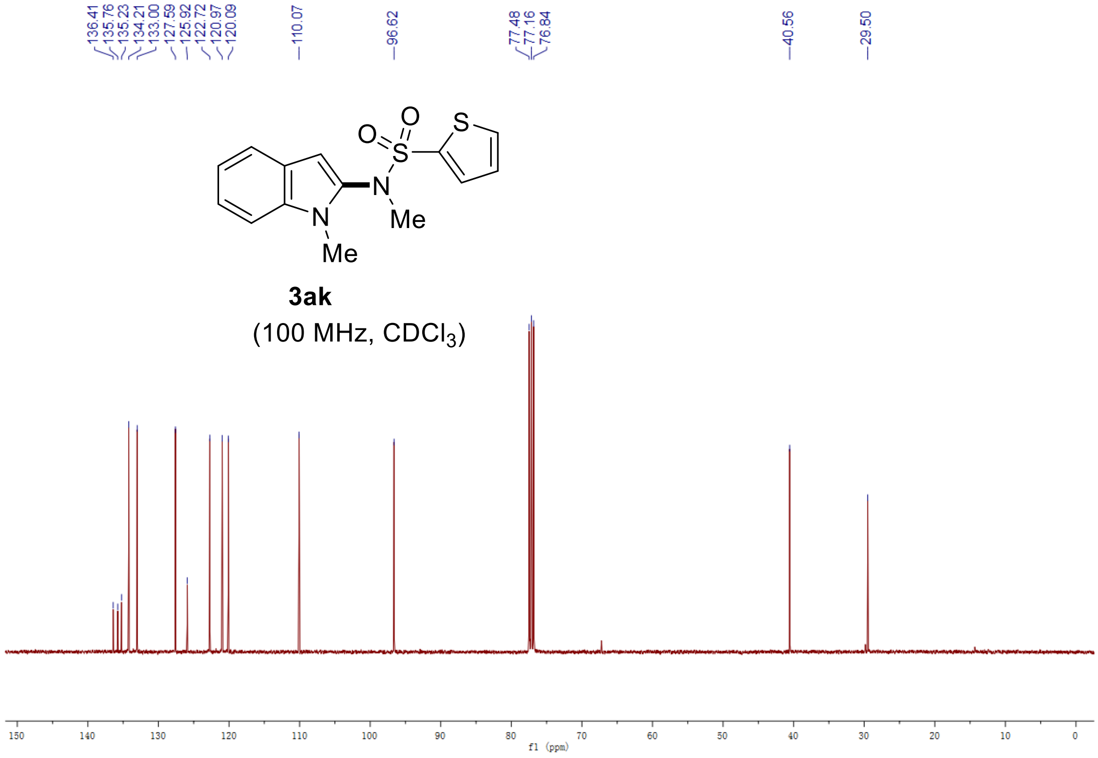
136.41
135.76
135.23
134.21
133.00
127.59
125.92
122.72
120.97
120.09
110.07
96.62
77.48
77.16
76.84
40.56
29.50
3ak
(100 MHz, CDCl3)
f1 (ppm)

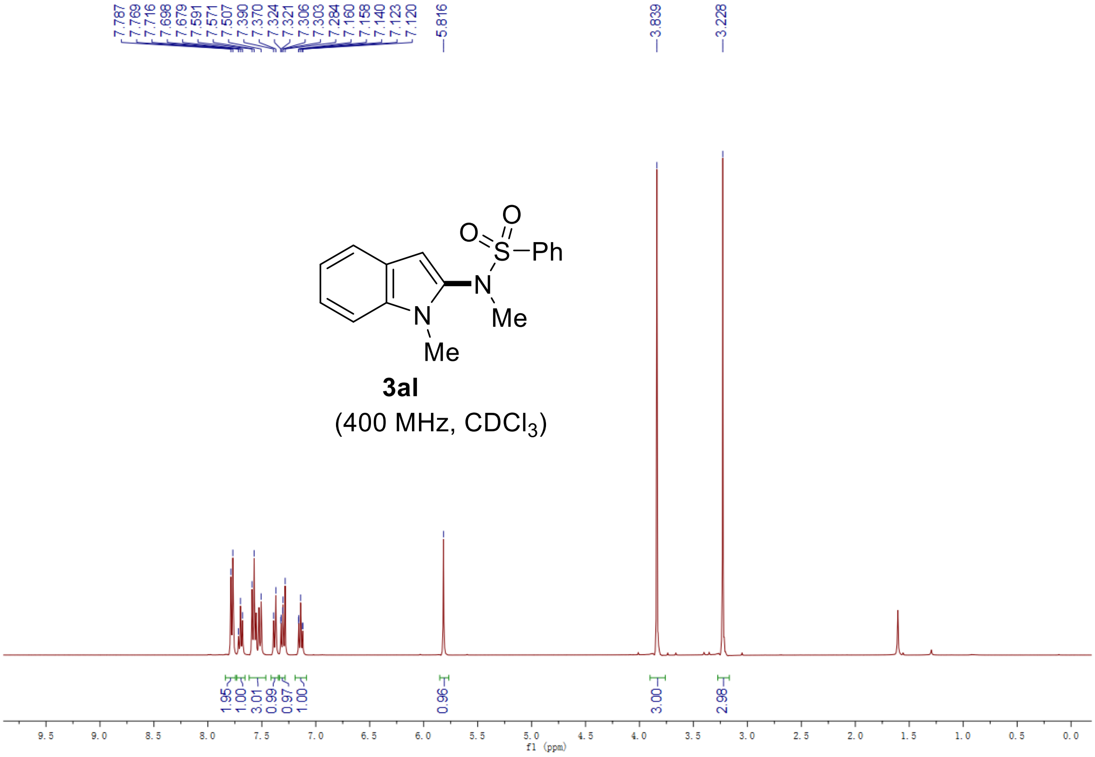
7.787
7.769
7.716
7.698
7.679
7.591
7.571
7.507
7.390
7.370
7.324
7.321
7.306
7.303
7.284
7.160
7.158
7.140
7.123
7.120
5.816
3.839
3.228
O
O
S
Ph
N
N
Me
Me
3al
(400 MHz, CDCl3)
1.95
1.00
3.01
0.99
0.97
1.00
0.96
3.00
2.98
f1 (ppm)

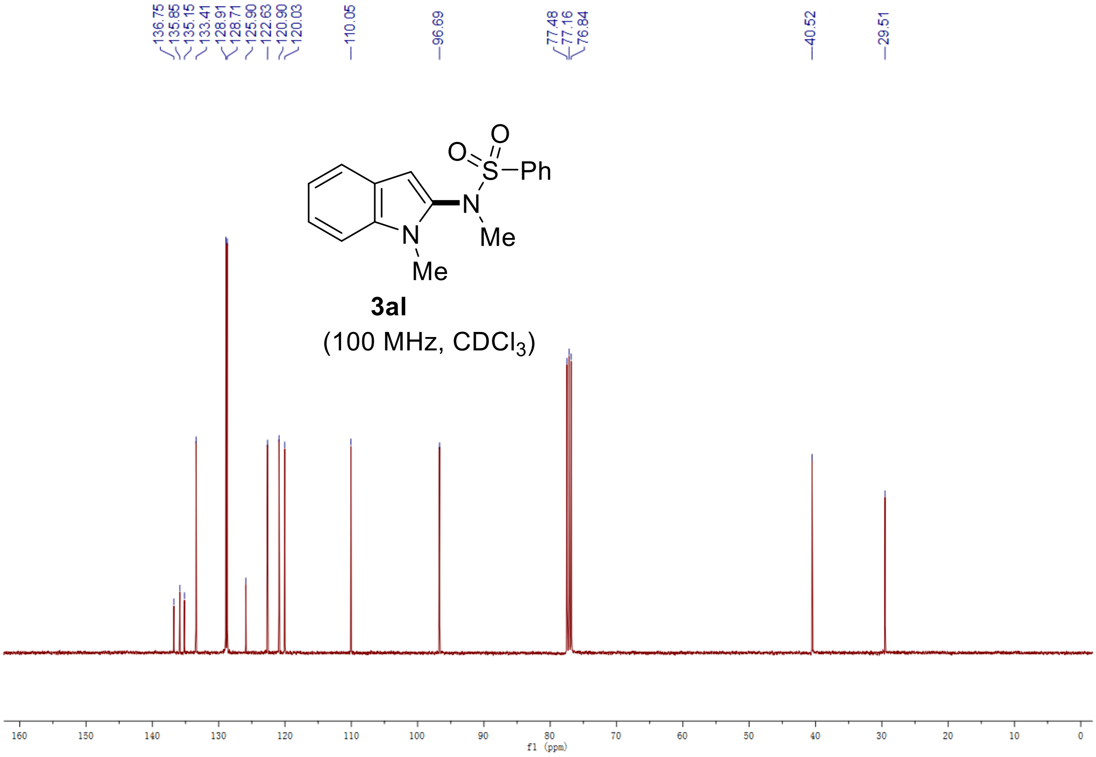
136.75
135.85
135.15
133.41
128.91
128.71
125.90
122.63
120.90
120.03
110.05
96.69
77.48
77.16
76.84
40.52
29.51
O
O
S
Ph
N
N
Me
Me
3al
(100 MHz, CDCl3)
f1 (ppm)

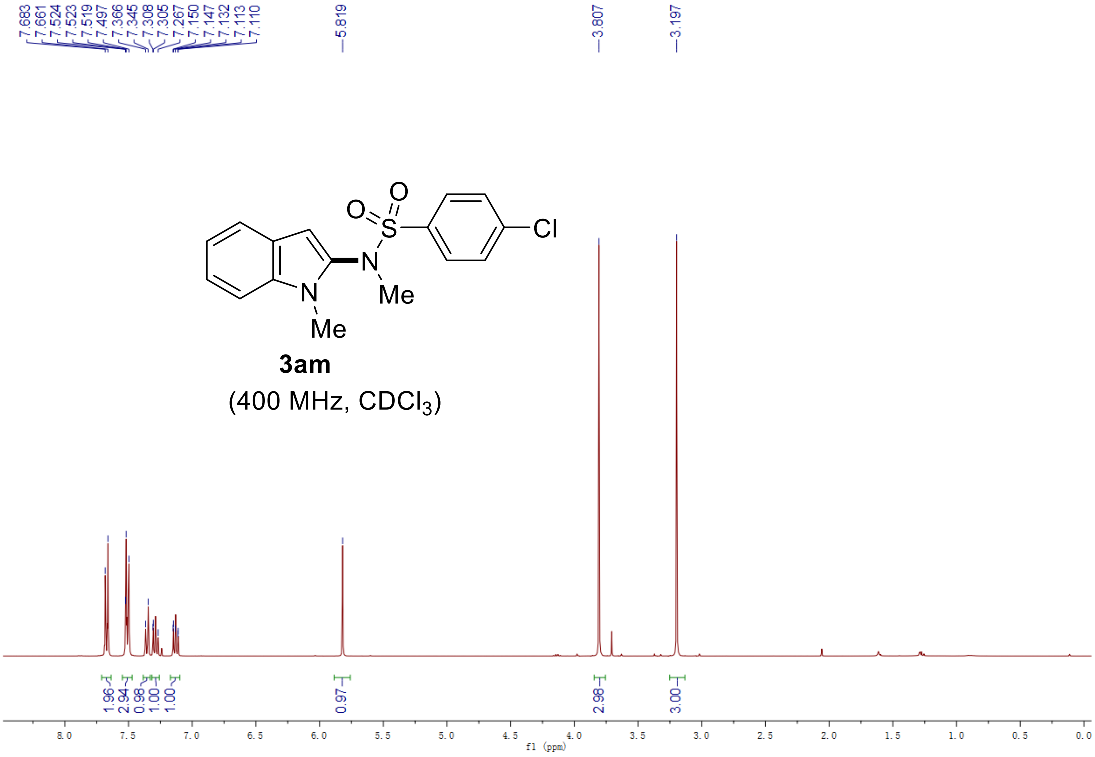
7.683
7.661
7.524
7.523
7.519
7.497
7.366
7.345
7.308
7.305
7.267
7.150
7.147
7.132
7.113
7.110
5.819
3.807
3.197
O
O
S
N
Cl
N
Me
Me
3am
(400 MHz, CDCl3)
1.96
2.94
0.98
1.00
1.00
0.97
2.98
3.00
8.0
7.5
7.0
6.5
6.0
5.5
5.0
4.5
4.0
3.5
3.0
2.5
2.0
1.5
1.0
0.5
0.0
f1 (ppm)

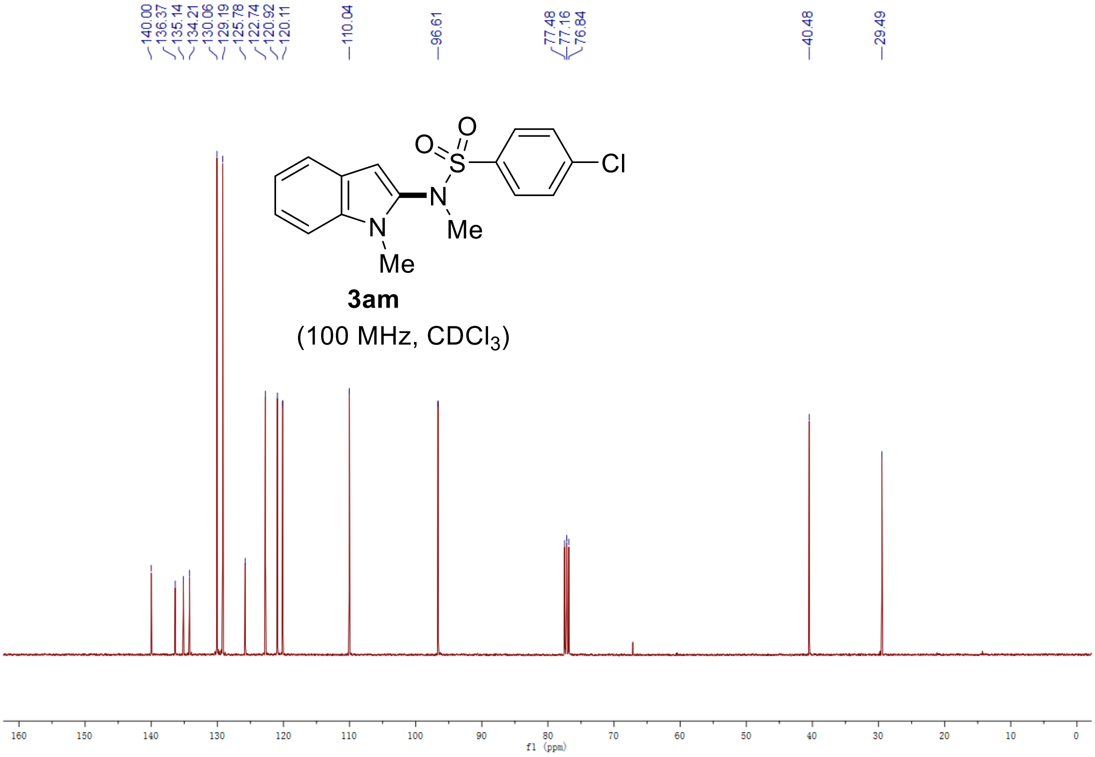
140.00
136.37
135.14
134.21
130.06
129.19
125.78
122.74
120.92
120.11
110.04
96.61
77.48
77.16
76.84
40.48
29.49
O
O
S
N
Cl
N
Me
Me
3am
(100 MHz, CDCl3)
160
150
140
130
120
110
100
90
80
70
60
50
40
30
20
10
0
f1 (ppm)

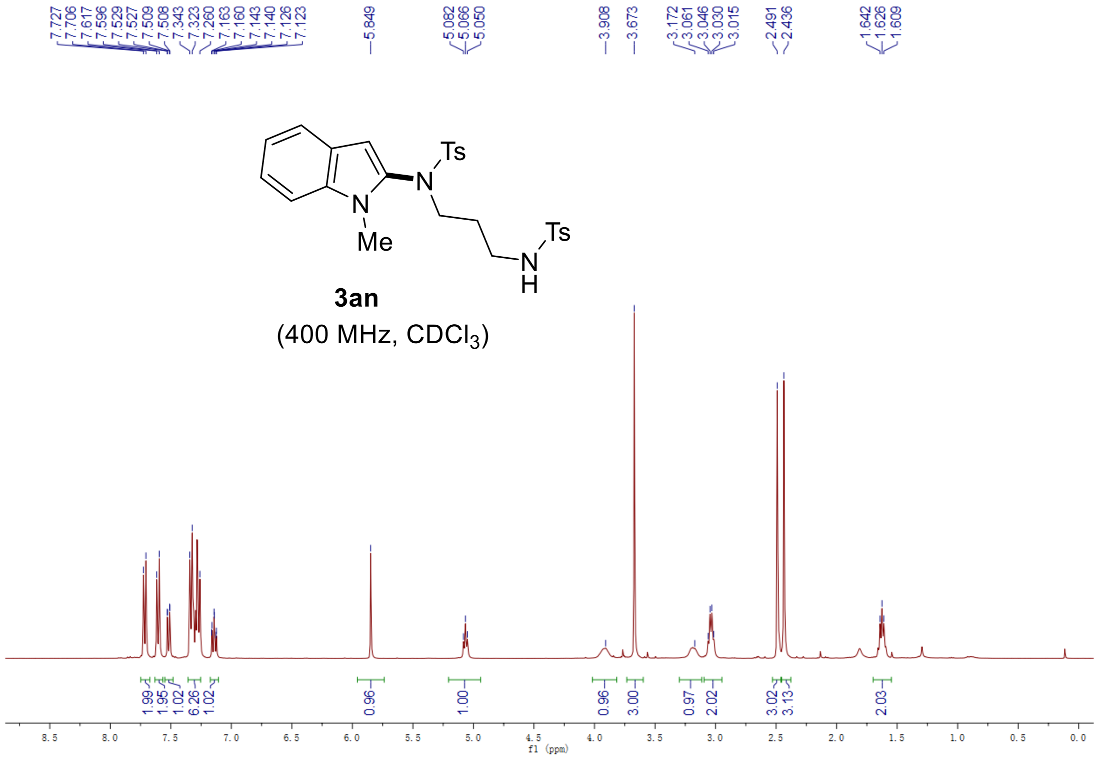

3an
(400 MHz, $CDCl_3$)

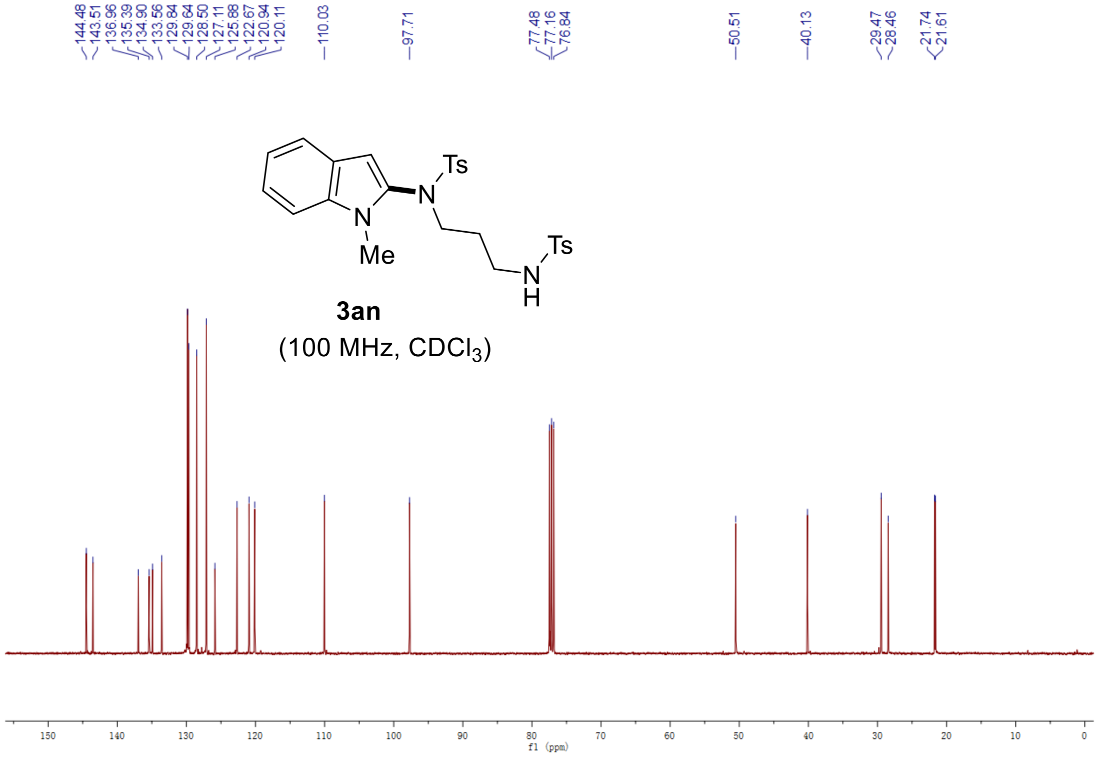

3an
(100 MHz, $CDCl_3$)

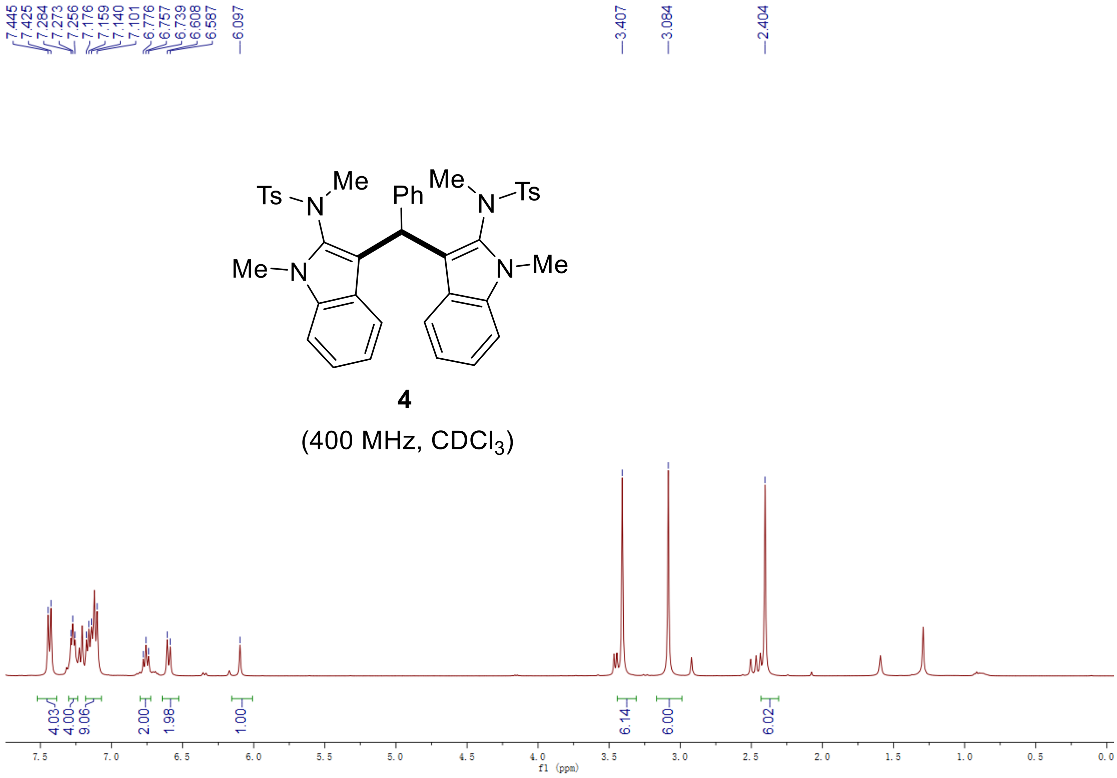
7.445
7.425
7.284
7.273
7.256
7.176
7.159
7.140
7.101
6.776
6.757
6.739
6.608
6.587
6.097
3.407
3.084
2.404
Ts
N
Me
Ph
Me
N
Ts
Me
N
N
Me
4
(400 MHz, $CDCl_3$)
4.03
4.00
9.06
2.00
1.98
1.00
6.14
6.00
6.02
7.5
7.0
6.5
6.0
5.5
5.0
4.5
4.0
3.5
3.0
2.5
2.0
1.5
1.0
0.5
0.0
f1 (ppm)

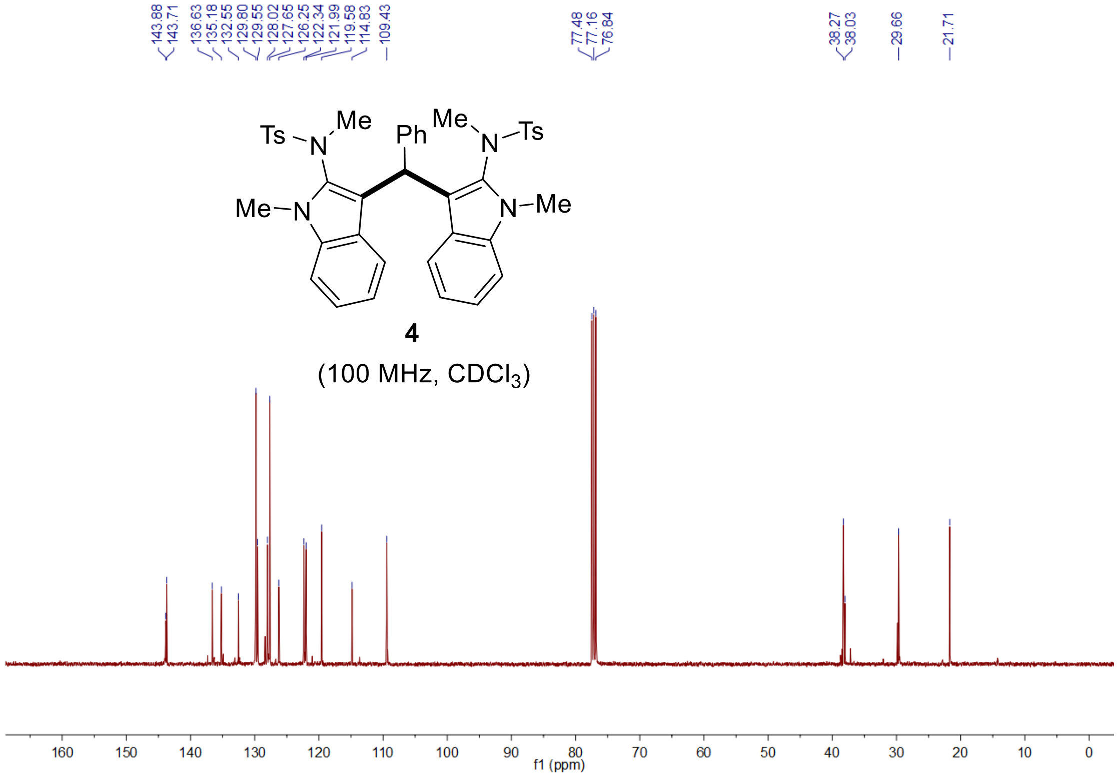
143.88
143.71
136.63
135.18
132.55
129.80
129.55
128.02
127.65
126.25
122.34
121.99
119.58
114.83
109.43
77.48
77.16
76.84
38.27
38.03
29.66
21.71
Ts
N
Me
Ph
Me
N
Ts
Me
N
N
Me
4
(100 MHz, $CDCl_3$)
160
150
140
130
120
110
100
90
80
70
60
50
40
30
20
10
0
f1 (ppm)

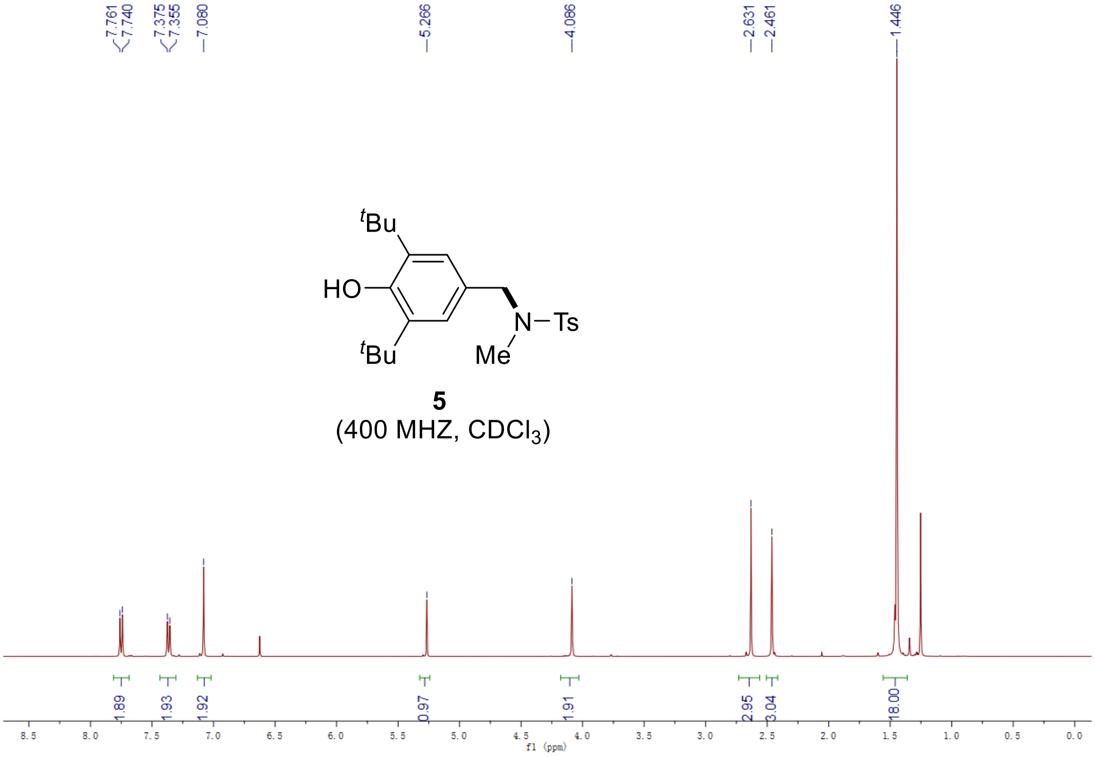
7.761
7.740
7.375
7.355
7.080
5.266
4.086
2.631
2.461
1.446
tBu
HO
N−Ts
tBu
Me
5
(400 MHZ, CDCl3)
1.89
1.93
1.92
0.97
1.91
2.95
3.04
18.00
8.5 8.0 7.5 7.0 6.5 6.0 5.5 5.0 4.5 4.0 3.5 3.0 2.5 2.0 1.5 1.0 0.5 0.0
f1 (ppm)

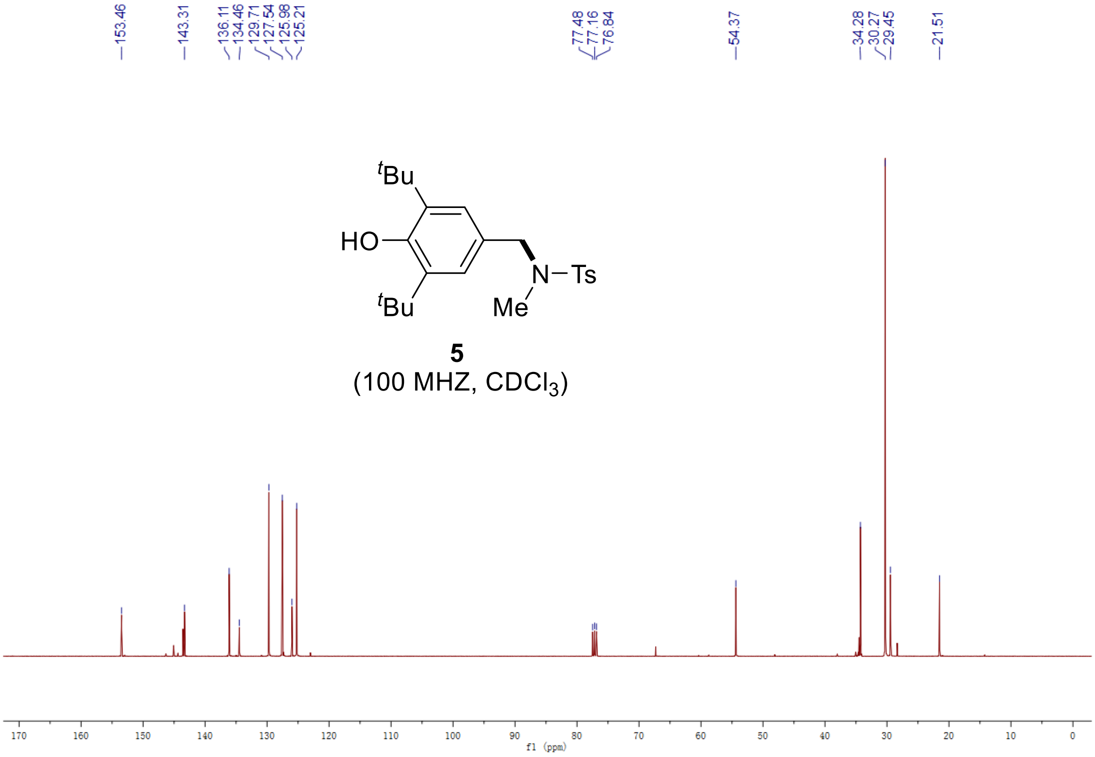
153.46
143.31
136.11
134.46
129.71
127.54
125.98
125.21
77.48
77.16
76.84
54.37
34.28
30.27
29.45
21.51
tBu
HO
N−Ts
tBu
Me
5
(100 MHZ, CDCl3)
170 160 150 140 130 120 110 100 90 80 70 60 50 40 30 20 10 0
f1 (ppm)

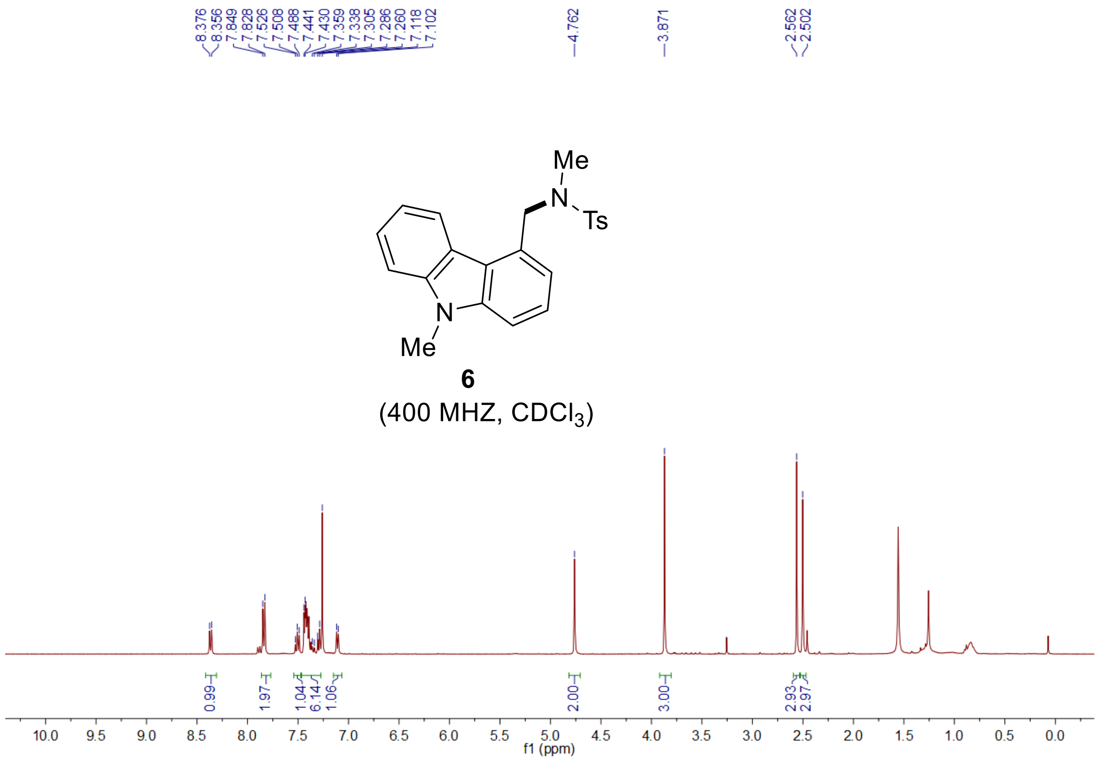
8.376
8.356
7.849
7.828
7.526
7.508
7.488
7.441
7.430
7.359
7.338
7.305
7.286
7.260
7.118
7.102
4.762
3.871
2.562
2.502
Me
N
Ts
N
Me
6
(400 MHZ, CDCl3)
0.99
1.97
1.04
6.14
1.06
2.00
3.00
2.93
2.97
10.0 9.5 9.0 8.5 8.0 7.5 7.0 6.5 6.0 5.5 5.0 4.5 4.0 3.5 3.0 2.5 2.0 1.5 1.0 0.5 0.0
f1 (ppm)

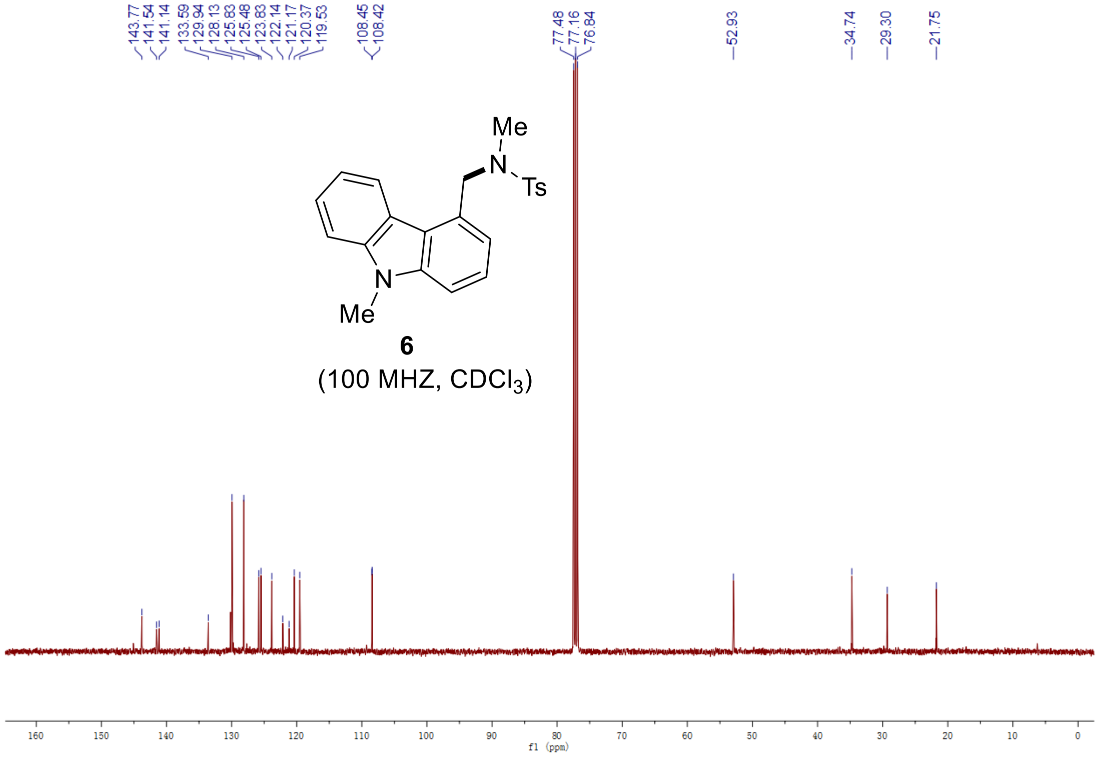
143.77
141.54
141.14
133.59
129.94
128.13
125.83
125.48
123.83
122.14
121.17
120.37
119.53
108.45
108.42
77.48
77.16
76.84
52.93
34.74
29.30
21.75
Me
N
Ts
N
Me
6
(100 MHZ, CDCl3)
160 150 140 130 120 110 100 90 80 70 60 50 40 30 20 10 0
f1 (ppm)

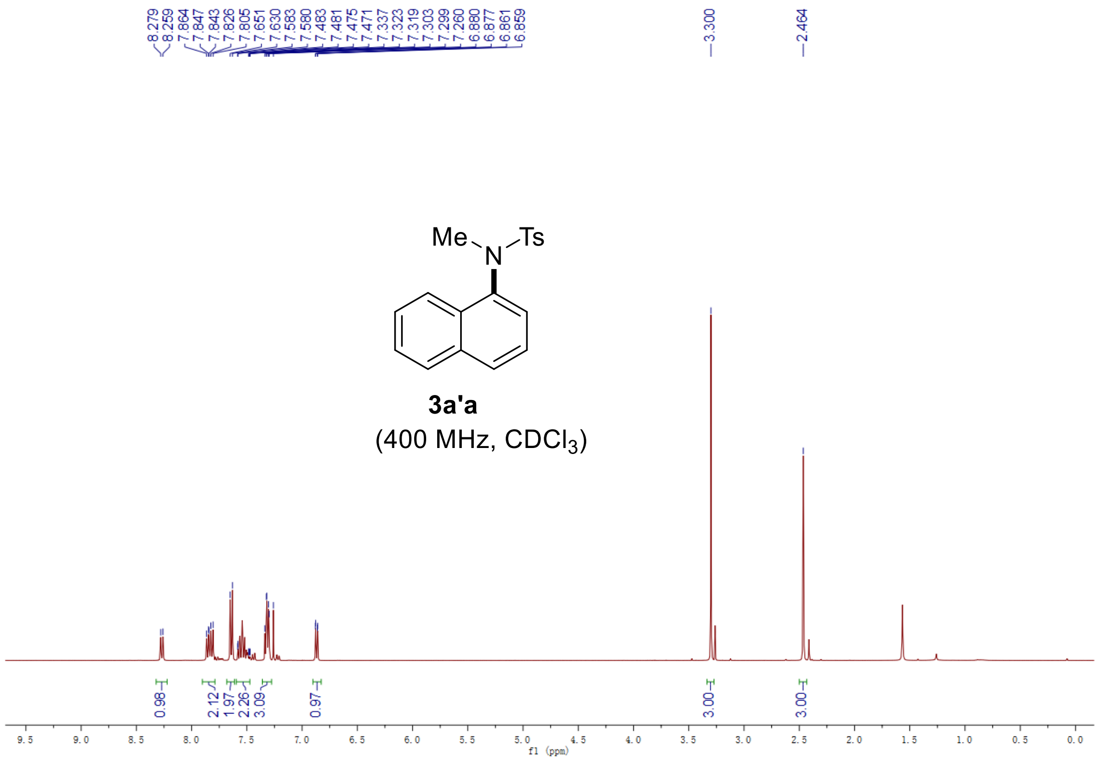
8.279
8.259
7.864
7.847
7.843
7.826
7.805
7.651
7.630
7.583
7.580
7.483
7.481
7.475
7.471
7.337
7.323
7.319
7.303
7.299
7.260
6.880
6.877
6.861
6.859
3.300
2.464
Me
N
Ts
3a'a
(400 MHz, CDCl3)
0.98
2.12
1.97
2.26
3.09
0.97
3.00
3.00
f1 (ppm)

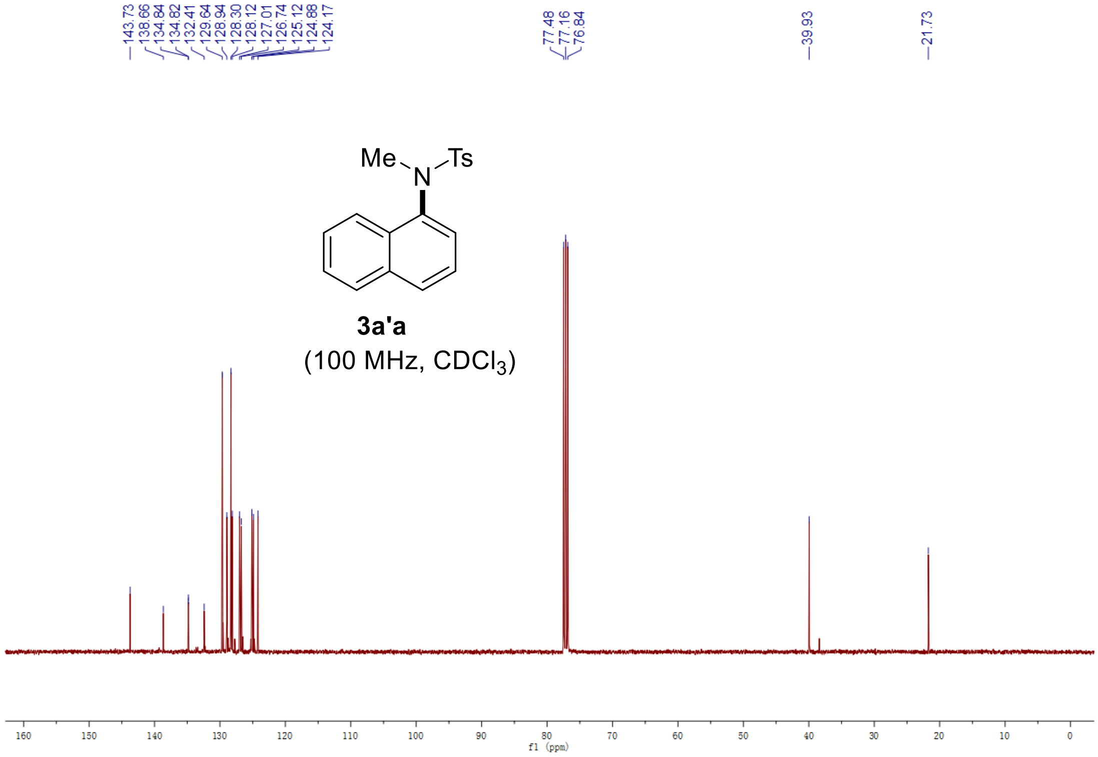
143.73
138.66
134.84
134.82
132.41
129.64
128.94
128.30
128.12
127.01
126.74
125.12
124.88
124.17
77.48
77.16
76.84
39.93
21.73
Me
N
Ts
3a'a
(100 MHz, CDCl3)
f1 (ppm)

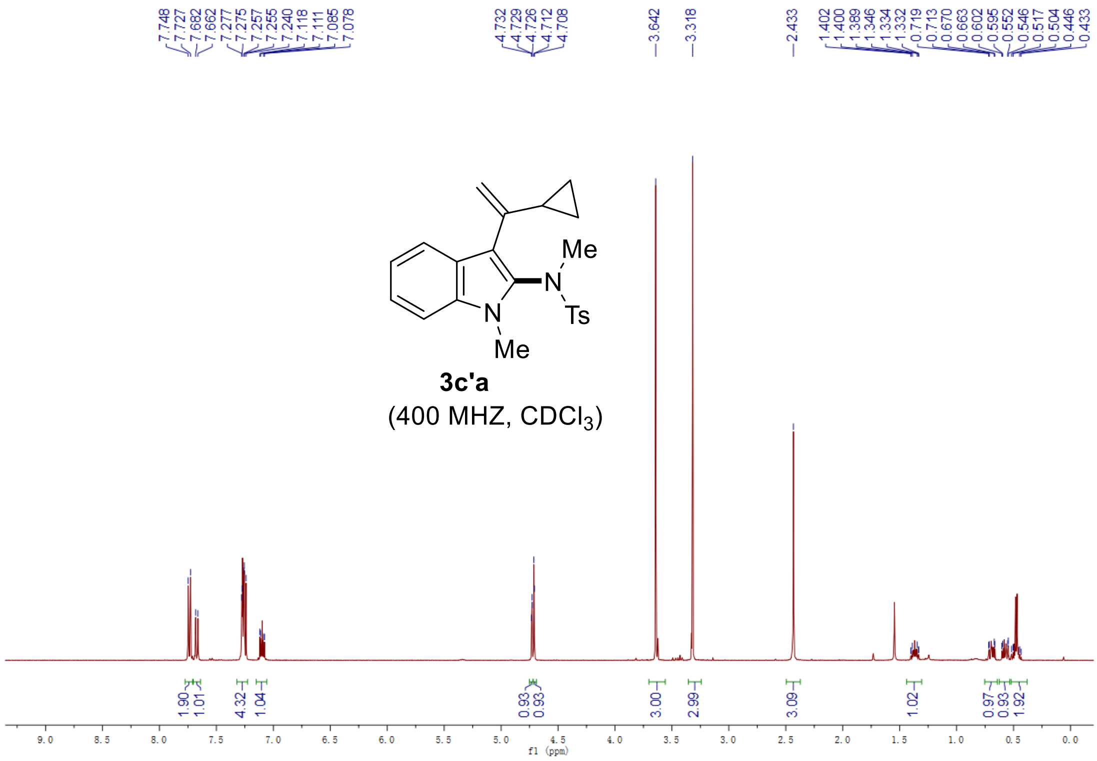

7.748
7.727
7.682
7.662
7.277
7.275
7.257
7.255
7.240
7.118
7.111
7.085
7.078
4.732
4.729
4.726
4.712
4.708
3.642
3.318
2.433
1.402
1.400
1.389
1.346
1.334
1.332
0.719
0.713
0.670
0.663
0.602
0.595
0.552
0.546
0.517
0.504
0.446
0.433
Me
N
Ts
N
Me
3c'a
(400 MHZ, CDCl3)
1.90
1.01
4.32
1.04
0.93
0.93
3.00
2.99
3.09
1.02
0.97
0.93
1.92
f1 (ppm)

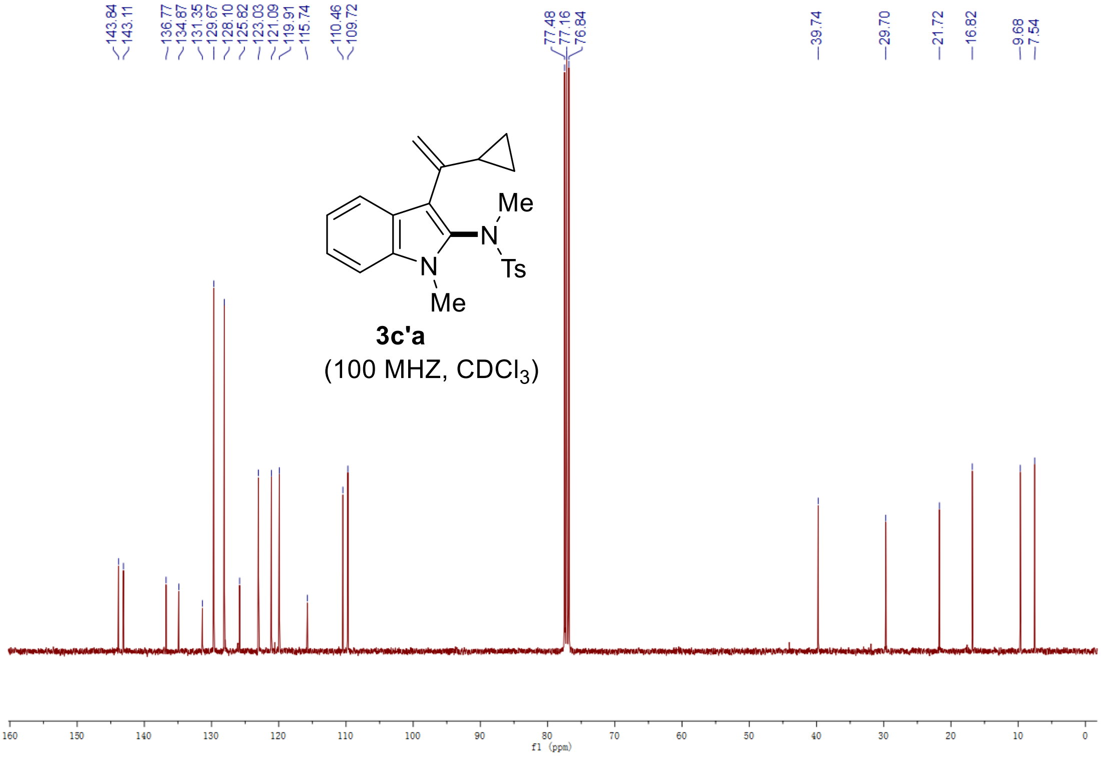

143.84
143.11
136.77
134.87
131.35
129.67
128.10
125.82
123.03
121.09
119.91
115.74
110.46
109.72
77.48
77.16
76.84
39.74
29.70
21.72
16.82
9.68
7.54
Me
N
Ts
N
Me
3c'a
(100 MHZ, CDCl3)
f1 (ppm)
